# From Functional Fatty Acids to Potent and Selective Natural-Product-Inspired Mimetics via Conformational Profiling

**DOI:** 10.1021/acscentsci.3c01155

**Published:** 2024-02-12

**Authors:** Lauren E. Markham, Thomas Koelblen, Harry R. Chobanian, Ariele Viacava Follis, Thomas P. Burris, Glenn C. Micalizio

**Affiliations:** † Department of Chemistry, Dartmouth College, 6128 Burke Laboratory, Hanover, New Hampshire 03755, United States; ‡ University of Florida Genetics Institute, P.O. Box 103610, 2033 Mowry Road, Gainesville, Florida 32610, United States; § ROME Therapeutics, 201 Brookline Avenue, Suite 1001, Boston, Massachusetts 02215, United States

## Abstract

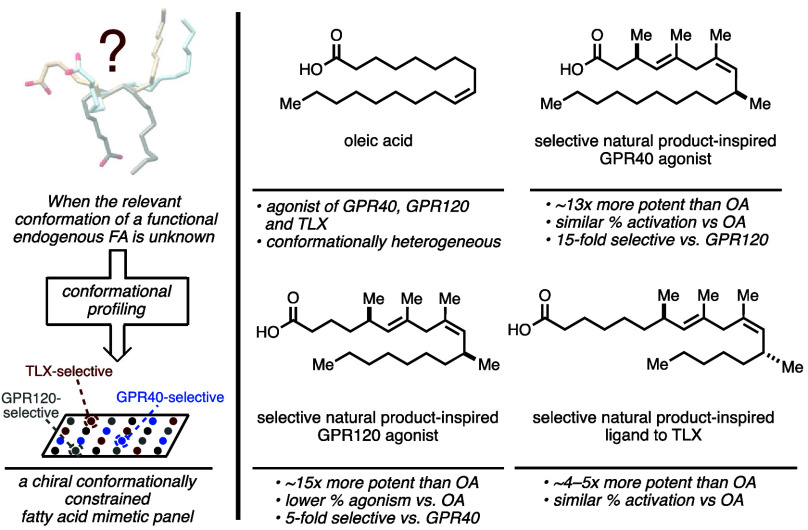

Fatty acids play important signaling roles in biology, albeit typically lacking potency or selectivity, due to their substantial conformational flexibility. While being recognized as having properties of potentially great value as therapeutics, it is often the case that the functionally relevant conformation of the natural fatty acid is not known, thereby complicating efforts to develop natural-product-inspired ligands that have similar functional properties along with enhanced potency and selectivity profiles. In other words, without structural information associated with a particular functional relationship and the hopelessly unbiased conformational preferences of the endogenous ligand, one is molecularly ill-informed regarding the precise ligand–receptor interactions that play a role in driving the biological activity of interest. To address this problem, a molecular strategy to query the relevance of distinct subpopulations of fatty acid conformers has been established through “conformational profiling”, a process whereby a unique collection of chiral and conformationally constrained fatty acids is employed to deconvolute beneficial structural features that impart natural-product-inspired function. Using oleic acid as an example because it is known to engage a variety of receptors, including GPR40, GPR120, and TLX, a 24-membered collection of mimetics was designed and synthesized. It was then demonstrated that this collection contained members that have enhanced potency and selectivity profiles, with some being clearly biased for engagement of the GPCRs GPR40 and GPR120 while others were identified as potent and selective modulators of the nuclear receptor TLX. A chemical synthesis strategy that exploited the power of modern technology for stereoselective synthesis was critical to achieving success, establishing a common sequence of bond-forming reactions to access a disparate collection of chiral mimetics, whose conformational preferences are impacted by the nature of stereodefined moieties differentially positioned about the C_18_ skeleton of the parent fatty acid. Overall, this study establishes a foundation to fuel future programs aimed at developing natural-product-inspired fatty acid mimetics as valuable tools in chemical biology and potential therapeutic leads.

## Abstract 2

A molecular strategy to query the relevance of distinct subpopulations of fatty acid conformers was used to deconvolute beneficial structural features that impart natural-product-inspired function.

## Introduction

Fatty acids are important natural products in biology, serving as energy sources and signaling agents that regulate many physiological processes of relevance to inflammation and metabolism.^1^ Therefore, the macromolecular receptors that are regulated by fatty acids, including nuclear receptors, G-protein-coupled receptors, and enzymes, are of great interest in chemical biology and medicine. Unfortunately, natural fatty acids typically have low binding affinities, due in part to the substantial loss in entropy incurred as they adopt a specific three-dimensional bound conformation. This structural deficiency, derived from the conformational flexibility of the natural ligand, is also a primary reason for their promiscuity (lack of selectivity). Molecules that mimic the activity of fatty acids (so-called fatty acid mimetics) have become of great interest as functionally relevant agents for chemical biology and leads for the development of therapeutics.^2^ While initially intended to orthosterically bind to fatty acid receptors, the vast majority of fatty acid mimetics surfacing from campaigns in medicinal chemistry have structures that are wildly different than their natural product ligand counterparts, typically boasting polysubstituted aromatics as a means to accomplish partial conformational rigidification and often lacking chiral conformational constraints that could reinforce functionally relevant chiral conformations related to those taken by the native fatty acid ligands.^2^ In contrast to these approaches, we have begun to develop fatty acid mimetics that have clearly defined natural-product-inspired chiral conformational constraints that aim to recapitulate the bound conformation of functionally relevant fatty acids and offer natural-product-inspired ligands that are substantially more potent and selective than their endogenous fatty acid counterparts.

When the structure of an endogenous fatty acid–receptor complex is known, hence revealing the precise and often chiral conformation that the achiral ligand takes when bound, the design of chiral conformationally constrained mimetics has proven to be relatively straightforward. For example, we recently employed the crystal structure of palmitoleic acid bound to ToxT, a transcription factor required for virulence in cholera, as a guide for developing a conformationally constrained chiral palmitoleic acid mimetic. These studies resulted in the identification of the most potent inhibitor of ToxT reported to date.^3^ Unfortunately, however, it is often the case that a functional relationship between a particular fatty acid and a receptor target is not supported by structural knowledge that clarifies how the natural fatty acid binds. In such cases, the conformational heterogeneity of the endogenous fatty acid does little to help inform the molecular design of mimetics that would offer enhanced potency and selectivity for the function of interest. To address this significant problem confronting the design of natural-product-inspired ligands of potential broad value in biology and medicine, we initiated a program aimed at accomplishing a synthetic means of “conformational profiling”, where a collection of chiral and conformationally constrained fatty acid mimetics could be used to guide hypothesis development regarding the preferred conformations that endogenous fatty acids take when bound to receptors of interest—doing so without structural data for the FA–receptor complex (e.g., from the use of X-ray crystallography or cryo-EM). Here we describe our initial foray into this area and report aspects of our molecular design, details of the synthetic chemistry required to prepare a collection of chiral and conformationally constrained mimetics, and an early glimpse of potent and selective fatty acid mimetics that target a variety of receptors of potential broad medicinal value.

**Figure 1:**
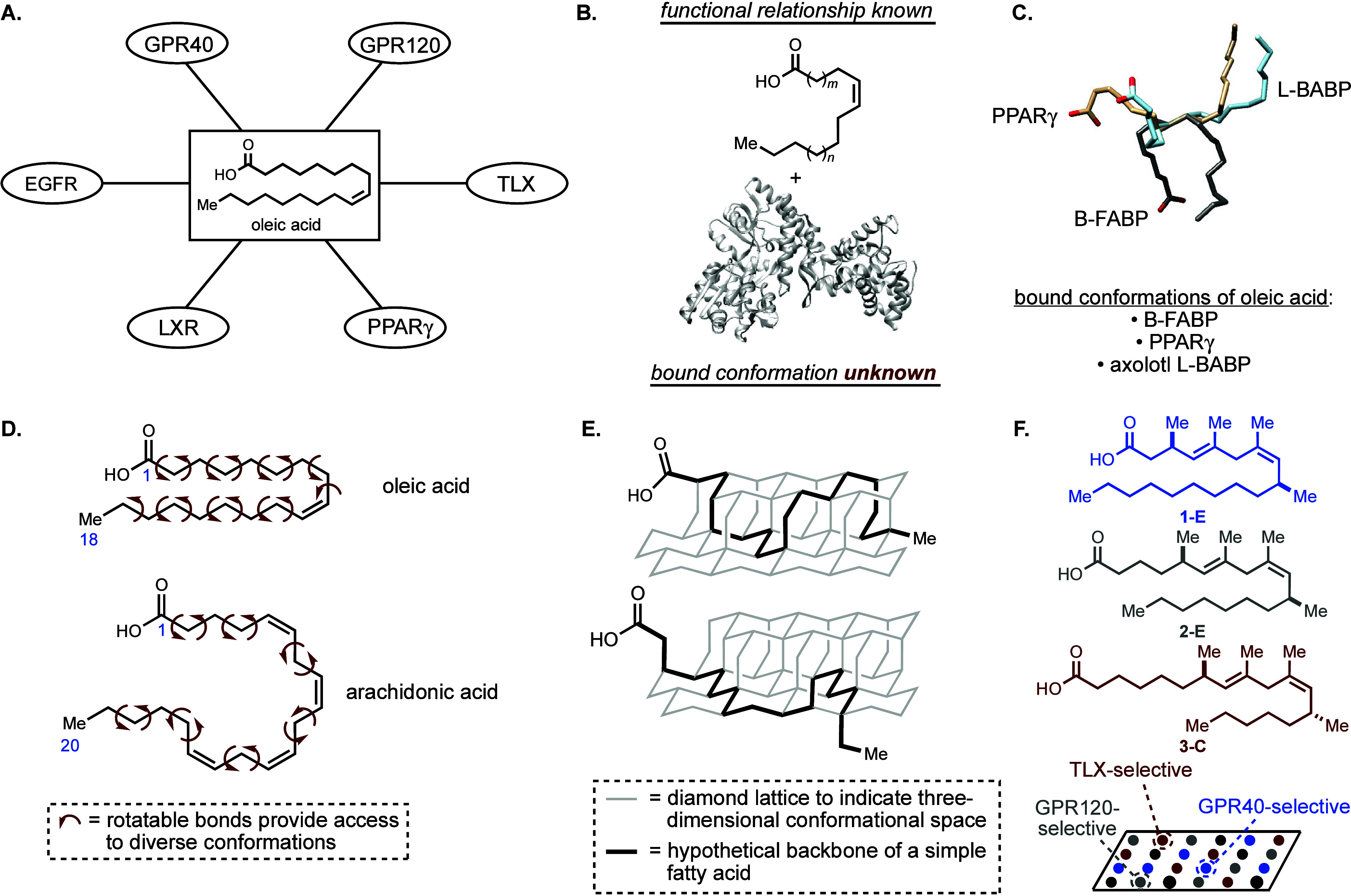
Introduction to conformational profiling. (A) Free fatty acids can serve as signaling molecules as endogenous ligands to many different receptors. (B) Free fatty acids are conformationally heterogeneous and are known to adopt a variety of different conformations when bound to their target receptors (PDB IDs: B-FABP = 1FE3; PPARγ = 6MD0; axolotl L-BABP = 2FTB (B-FABP = brain fatty acid binding protein; L-BABP = chicken liver bile acid binding protein; PPARγ = peroxisome proliferator-activated receptor gamma)). (C) While functional relationships between endogenous fatty acids and their target receptor are known, oftentimes the bound conformation of the fatty acid is not (PDB = 4XAJ (TLX)). (D) Depiction of the abundance of rotatable bonds and lack of conformational constraints present in a couple examples of natural fatty acids. (E) Traces of a carbon backbone through a diamond lattice to provide examples of different conformations one could envision a fatty acid taking. (F) Oleic acid mimetics from this work that are uniquely selective for GPR40, GPR120, and TLX.

This proof-of-concept study began by selecting oleic acid as the natural product fatty acid target of interest. As illustrated in Figure 1A, oleic acid is known to functionally modulate a wide range of receptors, including GPR40, GPR120, TLX, PPARγ, LXR, and EGFR, among others.^2,4−8^ While the conformations that oleic acid takes when bound to many of its targets remain generally unclear (e.g., GPR40 and TLX; Figure 1B), it is clear from available structural information that it adopts a range of distinct and diffuse conformations when bound to different biological macromolecules (Figure 1C). This reality is perhaps unsurprising because oleic acid, like many other endogenous fatty acids, is achiral and contains many rotatable bonds that offer the ability to easily adopt a range of diverse and often chiral conformations (Figure 1D,E).^9^

Herein, our efforts began with the goal of probing conformational space accessible to oleic acid with a panel of designed mimetics and then using a panel of biological assays to inform as to the potency and selectivity profiles of particular ligands within the collection of mimetics prepared. As illustrated in Figure 1F, it was hoped that these efforts would result in identifying clear differential activities of members of the collection, specifying particular natural-product-inspired mimetics that were selective at GPR40, GPR120, or TLX.^2,4−8^ Notably, these three receptors are of great interest in biology and medicine: GPR40 (FFAR1) plays a role in incretin release, glucose-dependent insulin secretion, taste, and inflammation and is a therapeutic target for many metabolic diseases, including type 2 diabetes;^2,4−8,10^ GPR120 (FFAR4) regulates fat metabolism and is a therapeutic target for the treatment of obesity;^11^ and TLX (NR2E1) is known to modulate adult neurogenesis and is a potential therapeutic target for neurological and psychiatric diseases such as Alzheimer’s, glioblastoma, and schizophrenia.^12,13^

## Results and Discussion

**Figure 2:**
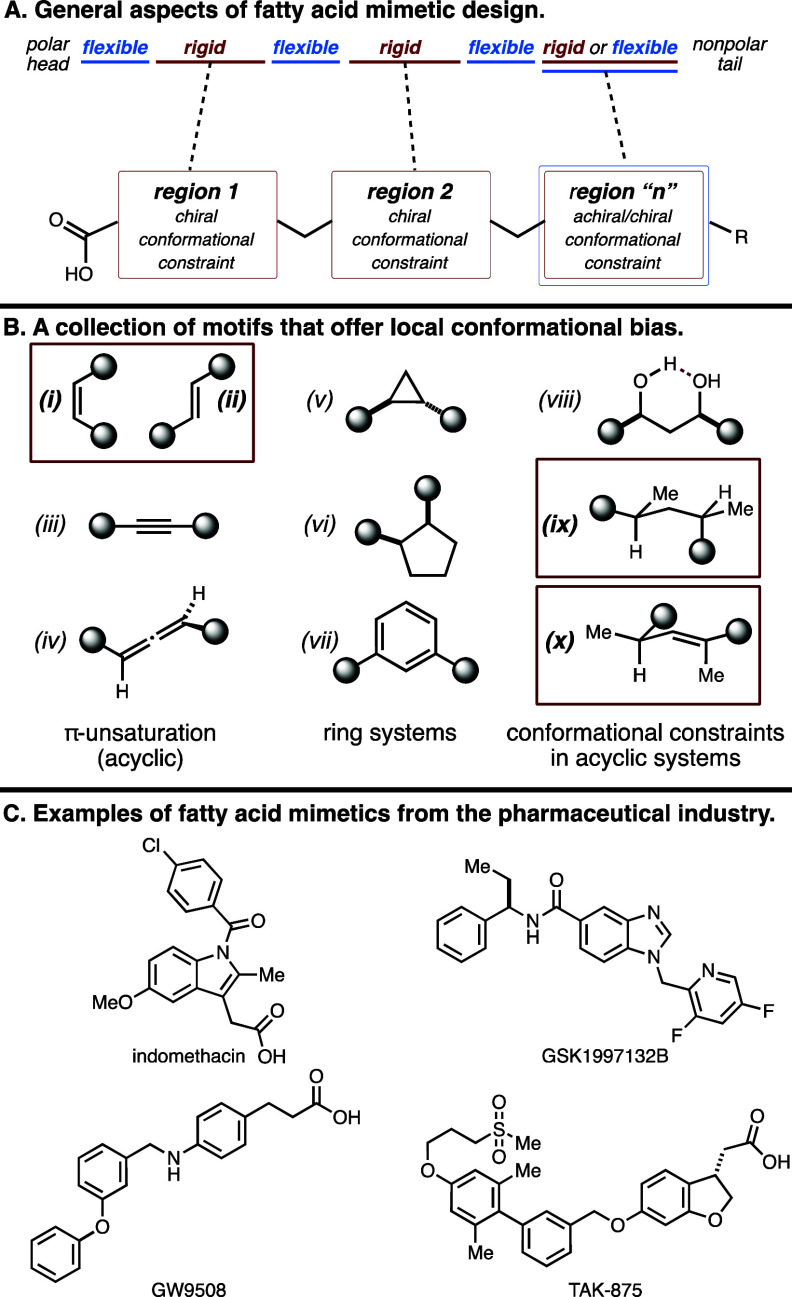
Molecular design. (A) Generic strategy. (B) Conformational constraints considered. (C) Examples of pharmaceutically derived fatty acid mimetics.

With oleic acid as our target, we contemplated basic features of ligand design and settled on the generic approach depicted in Figure 2A. Basically, retention of the polar carboxylic headgroup and hydrophobic tail (“R”) would be followed by strategic installation of chiral conformational constraints differentially positioned about the C_18_ acyclic carbon skeleton of the parent fatty acid. In this way, the acyclic backbones of the designed mimetics would be defined by alternating regions of molecular flexibility and rigidity, delivering chiral mimetics that have some degree of conformational flexibility yet boast clear local chiral conformational preferences at distinct locations along the backbone.

Next, the type of molecular moieties to be employed as conformational rigidifying elements were considered. As illustrated in Figure 2B, simple π unsaturation (i–iv), cyclic moieties (v–vii), and acyclic subunits (viii–x) were contemplated. Being inspired by the types of conformational constraints typically seen in endogenous free fatty acids and polyketide-derived natural products, we took inspiration from natural-product-inspired motifs that contain simple π unsaturation (e.g., i and ii) and those that offer conformational biasing based on minimization of A-1,3 strain and *syn*-pentane-like interactions (e.g., ix and x). Indeed, such natural-product-inspired conformational constraints are not common in pharmaceutically derived fatty acid mimetics (for examples, see Figure 2C).

**Figure 3:**
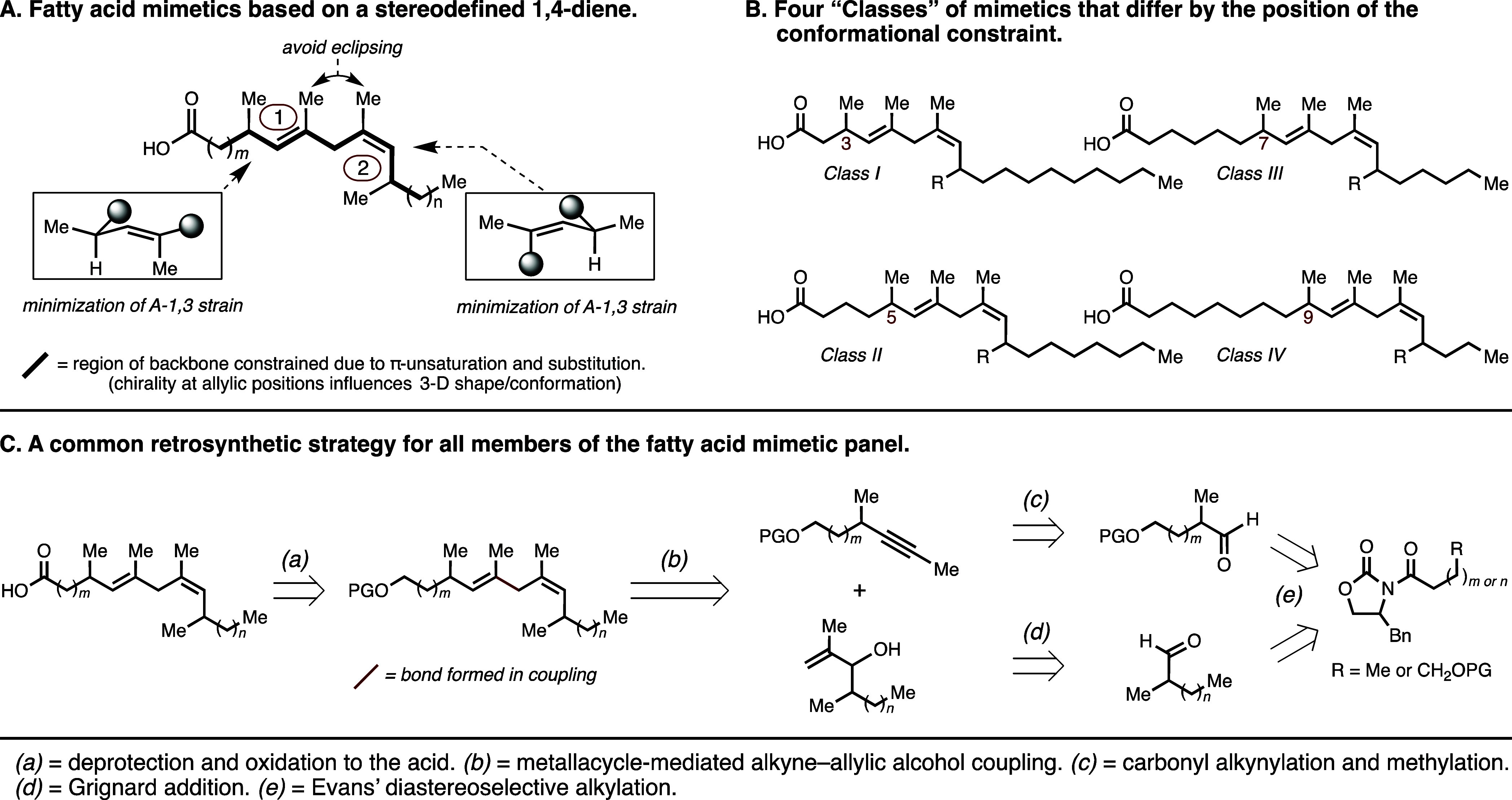
Ligand design for conformational profiling and a common retrosynthetic strategy for all panel members.

In efforts to establish a general molecular strategy for our conformational profiling set, we recognized that 1,4-dienes (a.k.a., skipped dienes) are present in many natural products, including fatty acids (e.g., arachidonic acid; Figure 1D).^14^ In fact, skipped polyenes are seen as structural motifs in scores of natural products, and we have previously developed a variety of synthetic methods capable of assembling such architecture in a convergent fashion.^15−19^ With the inspiration to exploit this type of functionality in our compound set, we imagined that the highly substituted and stereodefined 1,4-diene system depicted in Figure 3A could be particularly attractive as a structural motif that offers several features that impart local conformational preferences. Here, each alkene is stereodefined and trisubstituted (one *E* and one *Z*), and each can be flanked by an allylic stereocenter. Such a molecular scenario establishes two distinct loci within the fatty acid backbone where there would be clear chiral conformational preferences based on minimization of the A-1,3 strain. Finally, because of the nature and substitution of each alkene, it was expected that the conformational preference of the system would avoid coplanarity of the two alkenes (e.g., avoiding eclipsing *syn*-pentane-like interactions between the two allylic methyl groups). While not a focus of the current pursuits, it was recognized that this conformational biasing may impart additional stability to these mimetics that would differ from simple endogenous fatty acids that possess disubstituted (*Z*,*Z*)-1,4-dienes (e.g., arachidonic acid and linoleic acid).^14^

As illustrated in Figure 3B, four classes of mimetic were envisioned that differ by the position of the conformational biasing elements installed in the C_18_ backbone (termed Classes I–IV). It was anticipated that this molecular design would enable each member of the contemplated compound collection to be accessible from a similar sequence of carbon–carbon bond-forming reactions, thereby facilitating the synthetic chemistry required as the foundation of this program. In essence, we aimed to identify a single synthetic strategy that could be employed to access *all* of the members of the proposed collection, hence establishing a single synthetic means to populate diverse conformational space.

As illustrated in Figure 3C, the common synthetic pathway envisioned to accomplish these goals was based on late-stage deprotection and oxidation to generate the fully functionalized panel members. In turn, the substrate for deprotection was envisioned to derive from a convergent coupling process that simultaneously establishes each stereodefined trisubstituted alkene. It was recognized that regio- and stereoselective metallacycle-mediated coupling of simple internal alkynes with allylic alcohols would be uniquely effective in this regard.^15−19^ This coupling reaction would be preceded by synthetic chemistry capable of converting simple oxazolidinone starting materials to stereodefined chiral aldehydes and subsequently to the desired alkyne and allylic alcohol coupling partners.^20,21^

**Figure 4:**
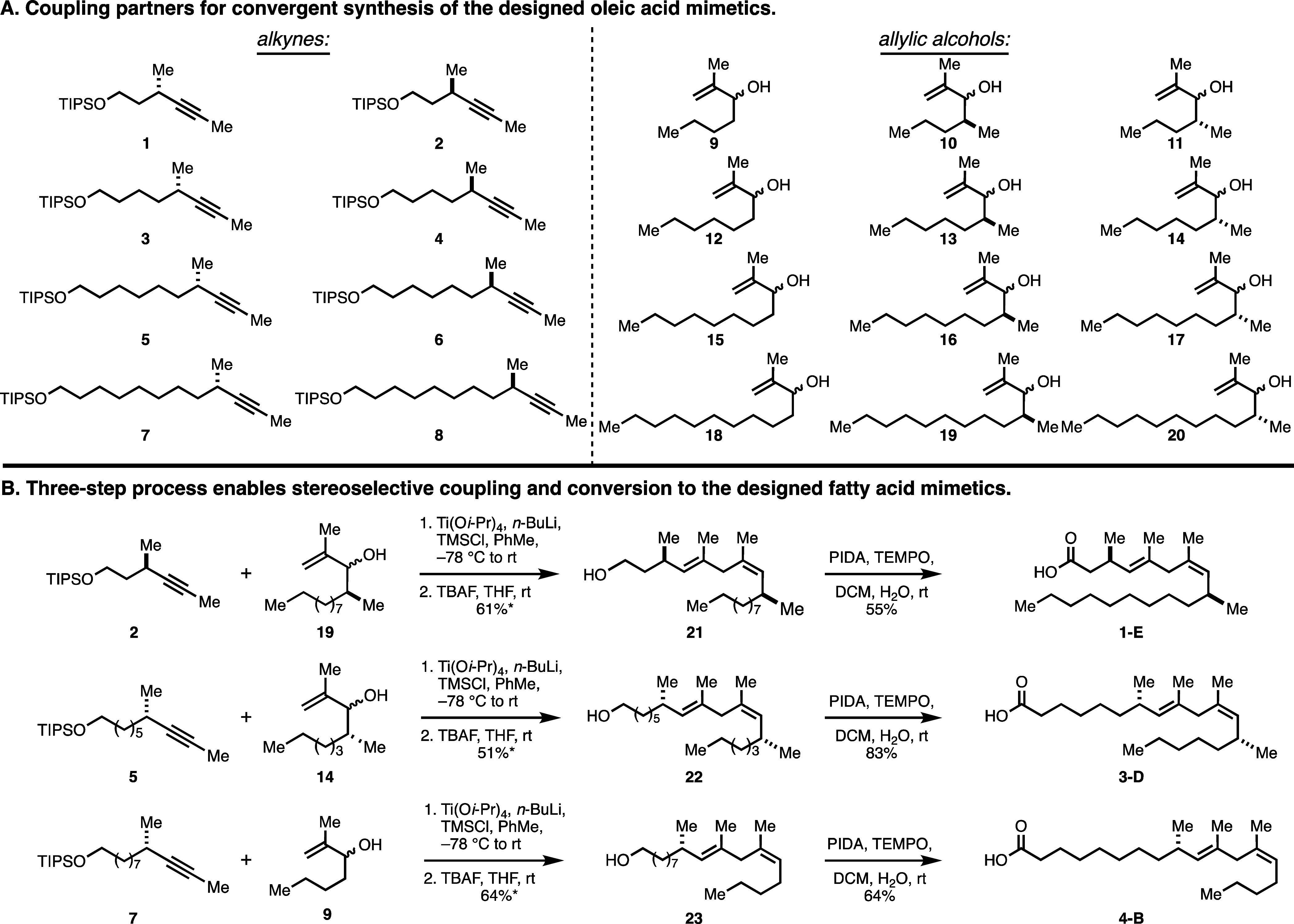
Convergent synthesis of members of the fatty acid mimetic collection. (A) Structure of the alkynes and allylic alcohols employed. (B) Examples of the chemical pathway for the synthesis of members of the fatty acid mimetic collection. [* = yield includes all isomers after deprotection: for **21**, rs = 3:1 and *Z*:*E* (of major regioisomer) = 13:1; for **22**, rs = 4:1 and *Z*:*E* (of major regioisomer) = 10:1; for **23**, rs = 4:1 and *Z*:*E* (of major regioisomer) = 7:1; only one alkene stereoisomer visible via ^1^H NMR for the minor regioisomeric coupling product].

The collection of alkynes (**1**–**8**) and allylic alcohols (**9**–**20**) employed in the synthesis of the fatty acid mimetic panel are illustrated in Figure 4A, all of which were prepared by related chemical methods beginning with different starting materials (see the Supporting Information). With samples of these coupling partners in hand, each fatty acid mimetic was assembled through metallacycle-mediated alkyne–allylic alcohol coupling, followed by desilylation and subsequent oxidation to the carboxylic acid (see Figure 4B for examples). Notably, the key convergent coupling reaction proved to be uniformly effective across the broad substrate scope explored, delivering the targeted 1,4-diene products [one alkene is set specifically as the *E* isomer due to the mechanism of the reaction, while the other alkene is selectively generated as the *Z* isomer].

An important characteristic of this coupling reaction is its indifference to the relative stereochemistry of the coupling partners and its ability to convert a mixture of allylic alcohol diastereomers to the same stereodefined (*Z*)-alkene-containing product. While useful here, it is important to appreciate that metallacycle-mediated coupling reactions of internal alkynes are often plagued by challenges associated with regioselection,^22^ and herein the coupling reactions proceeded with a regioselectivity (rs) of 3–4:1. Due to the modest levels of regioselectivity, careful purification was required to enrich samples to be used in subsequent profiling experiments.^23^

**Figure 5:**
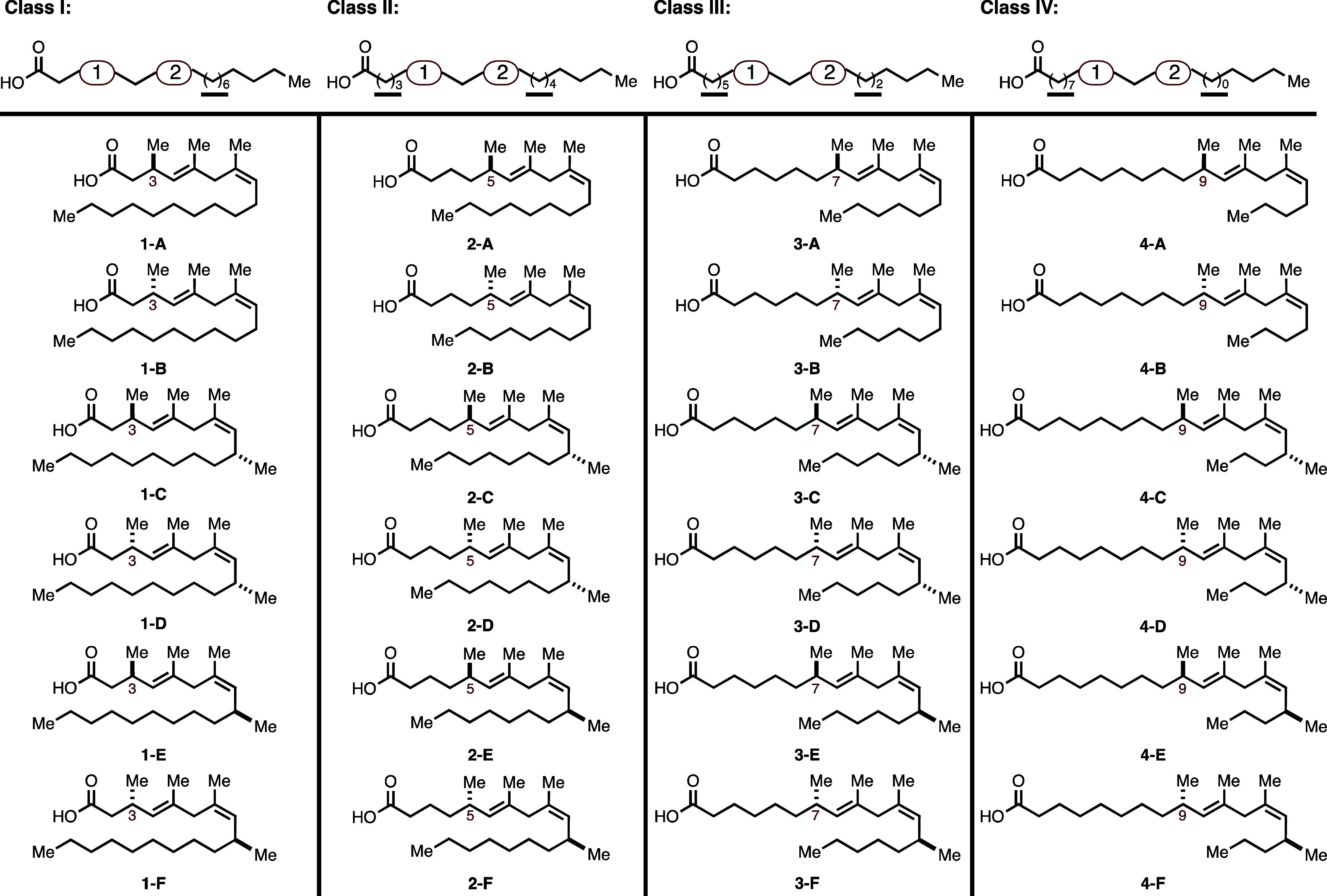
Structures of 24 oleic acid mimetics prepared. The carbon number where each conformational constraint begins within each ligand class is specified in red.

The panel of oleic acid mimetics that was prepared is depicted in Figure 5 and includes six different compounds for each mimetic class (**1A**–**F**, **2A**–**F**, **3A**–**F**, and **4A**–**F**). For Class I compounds, the first chiral conformational constraint appears at C3 of the fatty acid backbone. Each subsequent class is differentiated based on the positioning of the chiral constraint within the backbone (beginning at C5, then C7, then C9). Comparing the mimetics across each class, ligands with the same alphabetical descriptor (e.g., “**A**” vs “**B**” vs “**C**”, etc.) share the same stereodefined constraint (same substitution and stereochemistry).

**Figure 6:**
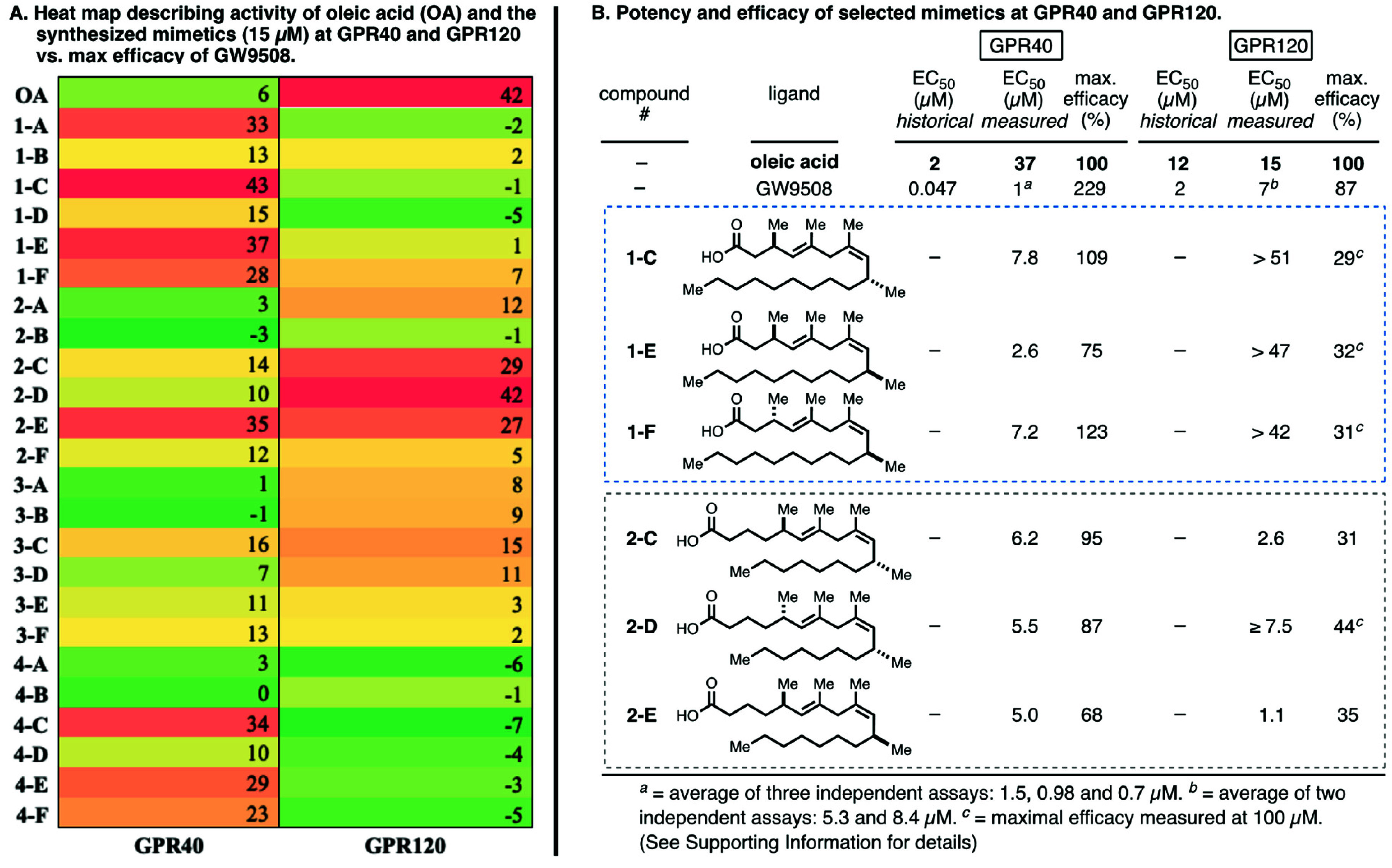
Evaluation of the designed fatty acid mimetics as ligands to GPR40 and GPR120. (A) Heat map showing % maximal efficacy of the collection for GPR40 and GPR120 (single dose at 15 μM). (B) Comparison of EC_50_ values for heat-map-directed select compounds at GPR40 and GPR120.

With these compounds in hand, attention was directed at evaluating their properties as ligands to GPR40, GPR120,^24^ and TLX. Initial profiling experiments were conducted targeting the GPCR targets at a single concentration (15 μM) in a commercially available β-arrestin assay. The results from these profiling experiments are depicted as a heat map in Figure 6A. Apparent from this representation of the data acquired is that the oleic acid mimetics that have maximal efficacy for GPR40 (e.g., **1-A**, **1-C**, **1-E**) are distinct from those that appear optimal for GPR120 (**2-C**, **2-D**, and **2-E**).

Following up on these single-concentration experiments, a handful of compounds were selected for investigation in dose–response titrations that would provide an indication of their intrinsic potency in comparison to oleic acid and the pharmaceutically derived dual GPR40 and GPR120 agonist GW9508. Notably, oleic acid has been reported to have potent stimulatory activity on CHO-mGPR40 cells (EC_50_ = 2 μM) and induces insulin secretion in MIN6 cells at concentrations as low as 1 μM^25,26^ while also being an agonist of GPR120 with an EC_50_ of 12 μM.^27^ Similarly, GW9508 has been reported as an agonist of GPR40 with an EC_50_ of 0.047 μM and an agonist of GPR120 with an EC_50_ of 2.2 μM.^28^

Here our evaluation of all compounds, including oleic acid and GW9508, was conducted with a PathHunter β-arrestin assay that monitors the activation of a GPCR in a homogeneous, nonimaging assay format using enzyme fragment complementation (EFC) with β-galactosidase as the functional reporter (see the Supporting Information). In this particular assay, oleic acid has an EC_50_ of 37 μM at GPR40 and 15 μM at GPR120, while GW9508 was observed to have an EC_50_ of 1 μM at GPR40 and 7 μM at GPR120 (Figure 6B). The reporter assay utilized delivers results for these positive controls at GPR40 that are right-shifted by ∼20-fold for both oleic acid and GW9508 relative to their reported historical activities, as discussed above.

Notably, from the collection of fatty acid mimetics analyzed, those within Class I were more enriched with GPR40 agonists than the other classes. Moving forward to determine the dose–responses of **1-C**, **1-E**, and **1-F**, it was found that they all proved to be exceptionally selective and potent agonists of GPR40 in comparison to oleic acid. These compounds activate GPR40 with similar % activation (efficacy) to oleic acid (76 and 124%) while displaying substantial selectivity over GPR120. The potency values on GPR40 were up to 14 times larger than with our control oleic acid (i.e., **1-E**). It is important to point out that these GPR40-selective agonists have a selectivity profile that is *opposite* to that seen for oleic acid, being several-fold more active at GPR40 in relation to GPR120 (oleic acid: GPR40 EC_50_ = 37 μM; GPR120 EC_50_ = 15 μM; see Figure 6B).

Unlike the GPR40-selective fatty acid mimetics from Class I, Class II appeared enriched with ligands that were effective in agonizing GPR120. Follow up dose–response titrations led to identifying ligands that have distinct potency and selectivity profiles in comparison to oleic acid. For example, as illustrated in Figure 6B, **2-C**, **2-D**, and **2-E** were found to be agonists of GPR120 with EC_50_ values ranging from 1 to ∼7 μM while surprisingly showing efficacy as agonists of GPR40 similar to oleic acid with EC_50_ values of ∼5 μM (∼7× more potent than oleic acid)—indeed, these Class II compounds have a very different selectivity profile than that established for the Class I compounds, which were found to be selective GPR40 agonists. Notably, unlike the efficacies observed for Class I compounds at GPR40 that mimic what was observed for oleic acid, the potent Class II compounds identified were found to be less effective at agonizing GPR120 than oleic acid (max efficacy between 31 and 45%). Nevertheless, compound **2-E** was found to have a unique potency and selectivity profile in comparison to either oleic acid or GW9508, showing 5-fold selectivity for GPR120 and a 7–15-fold enhanced potency.

Notably, the fatty acid mimetics that were identified as the most potent agonists of GPR120 in these studies (e.g., **2-C** and **2-E**) possess unsaturation at C6 and C9 of their carbon backbones. Recent structural studies of the GPR120–oleic acid complex by cryo-electron microscopy have concluded that there is an overall “L-configuration” of the ligand that exists bound inside the seven-transmembrane (7-TM) helix bundle of the receptor.^24^ These recent studies present how GPR120 differentiates “rigid double bonds” and “flexible single bonds”. Interestingly, our most potent mimetics contain a stereodefined (*Z*)-alkene at C9—albeit trisubstituted, the position of this π bond in these polyunsaturated fatty acid mimetics (**2-C** and **2-E**) is the same as the position of the *Z*-disubstituted alkene in oleic acid. We offer no additional insight regarding this observation and appreciate that the most potent functional fatty acid mimetics identified here for GPR120 have substantially depressed maximum % efficacy (as judged by the maximum response observed in the dose–response assays performed: oleic acid = 100% vs **2-E** = 35%) despite being significantly more potent than oleic acid (oleic acid EC_50_ = 15 μM; **2-E** IC_50_ = 1.1 μM).

**Figure 7:**
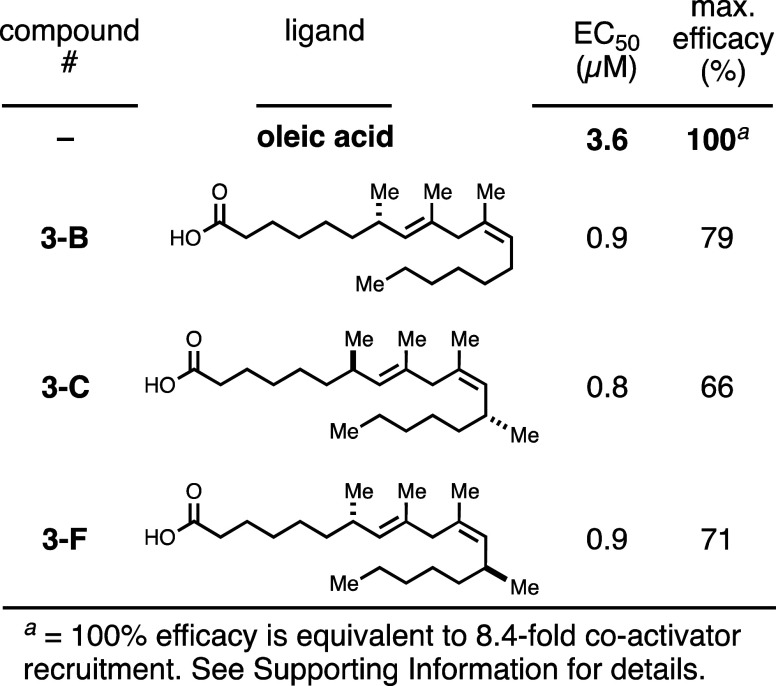
Evaluation of the designed fatty acid mimetics as ligands to TLX. Compounds with submicromolar potency are depicted. Full data associated with TLX activity of all fatty acid mimetics is presented in the Supporting Information.

In contrast to the GPCRs, TLX, a nuclear receptor, showed a preference for Class III ligands (Figure 7). TLX activity was determined using a ligand sensing assay that assesses the ability of a putative ligand to induce a conformational change within the ligand binding domain of TLX that facilitates recruitment of a transcriptional coactivator protein. This assay format has been used successfully to identify retinoids and oleic acid as ligands for TLX.^29,30^ As depicted in Figure 7, oleic acid displayed a potency of 3.6 μM in this assay and enhanced recruitment of a coactivator protein fragment by 8.4-fold (see the Supporting Information). Although several of the mimetics exhibited improved potency relative to oleic acid, Class III compounds **3-B**, **3-C**, and **3-F** displayed submicromolar potency (EC_50_ values from 0.8 to 0.9 μM). This represents an ∼4–4.5-fold improvement in potency relative to oleic acid. Importantly, the mimetics that displayed the highest degree of selectivity for TLX were distinct from those most selective for GPR40 and GPR120.

**Figure 8:**
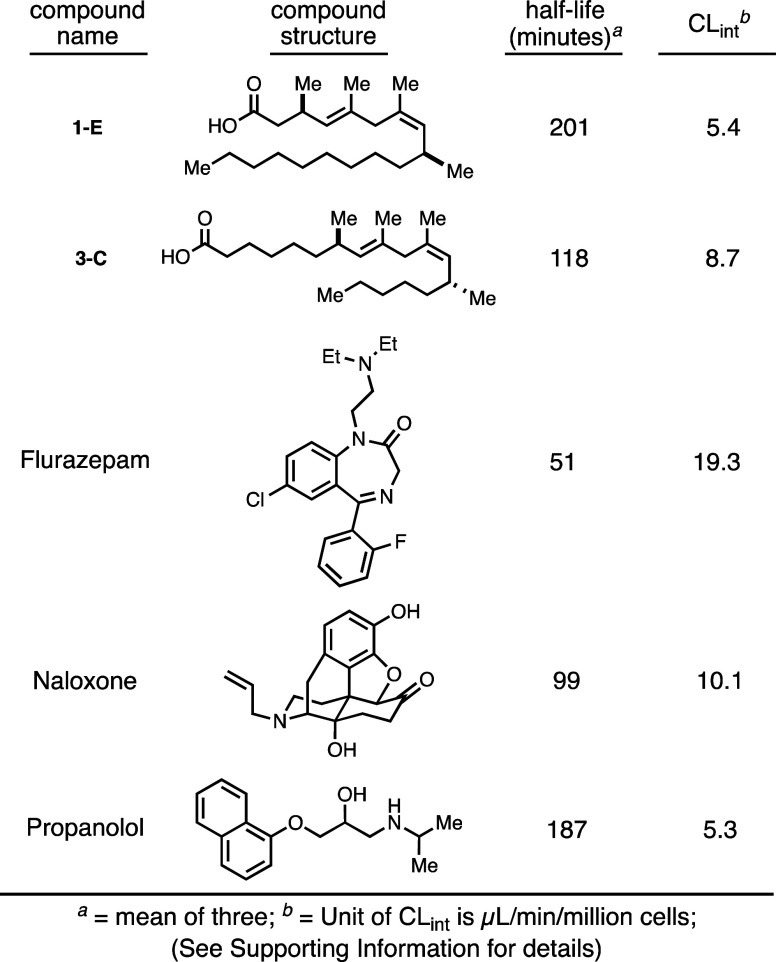
Metabolic stability in hepatocytes of mimetics versus positive controls.

Finally, while these studies were focused on the creation of a molecular platform to fuel the discovery of novel fatty acid mimetics that possess unique potency and selectivity profiles in comparison to oleic acid, the molecular structure of the ligands prepared might be anticipated to be highly unstable due to the presence of their central 1,4-dienes. To gain insight into potential metabolic issues, a preliminary assessment of **1-E** and **3-C** in a metabolic stability assay utilizing human hepatocytes was conducted. As illustrated in Figure 8, these fatty acids were found to have half-lives of 201 and 118 min, respectively, both of which are substantially greater than that of flurazepam and on the order of that observed for the naloxone and propanolol controls. The CL_int_ values observed for substrates **1-E** and **3-C** of 5.4 and 8.7 μL/min/million cells, respectively, would indicate a low turnover of the carboxylic acids assessed in the *in vitro* system.

**Figure 9:**
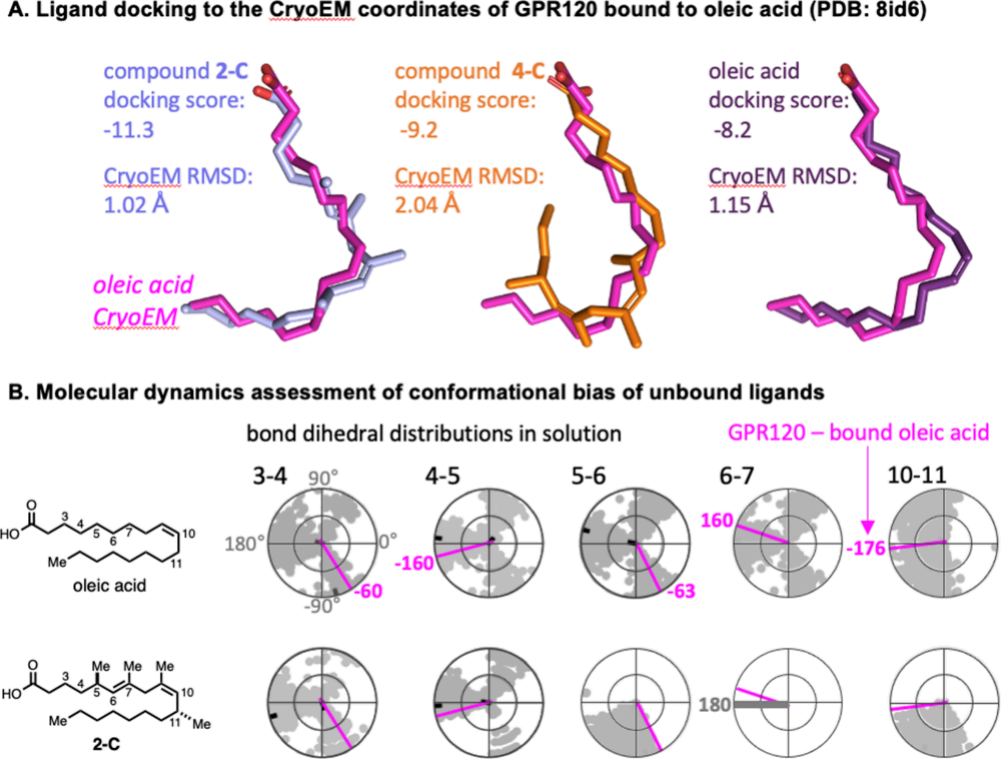
(A) Docking of **2-C**, **4-C**, and oleic acid to the GPR120 cryo-EM structure (PDB ID 8id6) stripped of the experimentally observed oleic acid molecule. Oleic acid and **2-C**, with agonist activity against GPR120, show minimal residual mean square deviation (RMSD) of their aliphatic chains relative to the cryo-EM structure, while **4-C**, which is inactive against GPR120, has a higher RMSD. For additional data regarding **2-E**, see Supplemental Figures 1 and 2. (B) Conformational analysis of oleic acid and **2-C** from molecular dynamics simulations of the unbound molecules in solution. The radar plots illustrate the dihedral angles populated at each bond depicted through molecular dynamics simulations. Several bond dihedrals in **2-C** show conformational bias toward the angles experimentally observed in the GPR120-bound oleic acid conformation. Bond dihedrals not shown in the figure show similar distributions between oleic acid and compound **2-C**.

To evaluate our hypothesis of selective conformational bias of the ligands prepared in this study, we took advantage of the recently reported structure of GPR120 bound to oleic acid (PDB ID 8id6).^24^ Molecular docking to the GPR120 coordinates showed that **2-C**, **2-E**, and oleic acid readily adopt a binding mode and aliphatic chain conformation which deviates minimally from the oleic acid molecule modeled in the cryo-EM structure. Conversely, **4-C** that was found to be inactive against GPR120 (Figure 6A) exhibited a marked deviation from the experimental oleic acid molecule, especially in the conformation associated with the region spanning carbons 14–18 (Figure 9 and Supplemental Figure 1). To further investigate whether the improved docking parameters for GPR120-active compounds relative to those of an inactive compound arose from a differential conformational bias between these molecules in their unbound state, we performed molecular dynamics (MD) simulations of the unbound molecules in water. Inspection of bond dihedral angle distributions over the course of the MD trajectories of **2-C** (Figure 9B) and **2-E** (Supplemental Figures 1 and 2) showed a preference for subsets of dihedral angles near the angles observed in the GPR120-bound conformation of oleic acid at the bonds between C3–C4, C4–C5, C5–C6, and C10–C11 compared to the MD trajectory of unbound oleic acid (Figure 9B and Supplemental Figure 2). Conversely, **4-C** showed a conformational bias against the dihedral angles observed in the GPR120-bound conformation of oleic acid at the bonds between carbons spanning C13–C16 and also at C9–C10 (the position of the cis double bond in oleic acid; Supplemental Figure 3). In conclusion, while limited to the benchmark of the GPR120-bound oleic structure, as structures of oleic acid bound to GPR40 and TLX have not yet been determined, computational analysis of the conformational space and predicted binding modes of the ligands discussed supports the hypothesis of a preselection of binding-competent ligand shapes and topologies through conformational bias.

## Conclusions

In summary, the present study has been focused on the challenge of designing potent and selective fatty acid mimetics in cases where the structure of the endogenous fatty acid–receptor complex is not well understood. Due to the conformational heterogeneity of fatty acids and the promiscuity of their many receptors, this lack of structural information leaves ligand design programs ill-informed as to the molecular details required to achieve potent and selective natural-product-inspired ligands that offer similar activation parameters as the endogenous fatty acids yet do so with enhanced potency and selectivity profiles. To address this issue, we have designed a panel of fatty acid mimetics that boast unique conformational preferences as a means to accomplish what we have termed “conformational profiling”, a process conceived to help determine which conformational preferences are best to mimic the activity of a particular fatty acid at a selected receptor, doing so in a manner that provides selectivity not possible with the endogenous fatty acid ligands.

Using oleic acid as a test case, these goals have resulted in the design of a 24-member collection of fatty acid mimetics that is constrained by a stereodefined 1,4-diene optionally flanked by an allylic stereocenter. The synthetic chemistry selected to fuel these studies was based on the application of a convergent coupling reaction that enables stereoselective union of internal alkynes with simple allylic alcohols. Notably, this convergent coupling reaction (an alkoxide-directed metallacycle-mediated cross-coupling) resulted in a unified synthetic strategy that was *applicable to all panel members*. In this way, a single general synthetic strategy proved capable of populating diverse regions of conformational space inspired by the parent fatty acid—in this case, oleic acid.

Initial exploration of the activities of members of the panel was achieved at a single concentration at GPR40 and GPR120 (see the heat map in Figure 6A). The results of this analysis revealed that the panel of fatty acid mimetics contained members that were clearly functionally distinct from one another and led to subsequent dose–response studies to identify the uniquely potent and selective modulators of GPR40 and GPR120 (Figure 6B). Moving away from GPCR targets to an example of a nuclear receptor, our studies revealed a different subpopulation of fatty acid mimetics that are submicromolar functional ligands to TLX (Figure 7). With the goal of establishing preliminary metabolic stability data for the natural-product-inspired fatty acid mimetics prepared here, a study of the intrinsic clearance in human hepatocytes of two of the ligands prepared was conducted. Notably, each ligand evaluated had a half-life similar to those of the positive controls examined (including naloxone and propanolol) and low CL_int_ values, making these acids attractive for further studies.

These studies have resulted in the identification of leads for the development of potentially valuable agonists of GPR40^31^ that reside within the same arbitrary class of mimetics prepared (Class I) but contain different stereochemical features. For example, the less potent GPR40 agonist congeners **1-C** and **1-F** are enantiomers of one another, while the more potent mimetic (**1-E**) is a unique enantiodefined diastereomer of these other ligands. In contrast, the most potent GPR120 agonist identified (**2-E**) is enantiomeric to the less potent congener **2-D** and is diastereoisomeric to **2-C**. The results for TLX are similar in that all ligands with submicromolar potency belong to Class III (possessing alkenes in the same position within the carbon backbone with respect to one another), two of which are an enantiomeric pair (**3-C** and **3-F**). The identification of enantiomeric pairs that function at each of the receptors investigated here was unanticipated and remains poorly understood, albeit potentially resulting from the promiscuity of each receptor. That said, we expect that further conformational rigidification of the leads identified in the present study will chart a course to even more potent and selective mimetics targeting selected receptors. We look forward to pursuing such studies and expanding this approach to conformational profiling to other medically relevant fatty acids.

## Supporting Information Available

Materials and methods, experimental procedures (chemical synthesis), biological procedures and data, and NMR spectra of novel compounds (PDF)
