# Supplementary material for: From Functional Fatty Acids to Potent and Selective Natural-Product-Inspired Mimetics via Conformational Profiling
Source: ACS Cent Sci. 2024 Feb 12;10(2):477–86. doi: 10.1021/acscentsci.3c01155 (PMC10906247; doi:10.1021/acscentsci.3c01155)

Supporting Information

From Functional Fatty Acids to Potent and Selective Natural Product-Inspired Mimetics via Conformational Profiling

Lauren E. Markham[†], Thomas Koelblen[#], Harry R. Chobanian[††], Ariele Viacava Follis[††], Thomas P. Burris[#], and Glenn C. Micalizio[†,*]

[†] 6128 Burke Laboratory, Department of Chemistry, Dartmouth College, Hanover, NH 03755, USA
[#] University of Florida, Genetics Institute, PO Box 103610, 2033 Mowry Rd., Gainesville, FL 32610, USA
[††] ROME Therapeutics, 201 Brookline Ave. Suite 1001, Boston, MA 02215, USA

Corresponding Author Email: glenn.c.micalizio@dartmouth.edu

Table of Contents Page

1. **Materials and Methods** S2
   A. **Reagents and Solvents** S2
   B. **Reaction Set-Up and Purification** S2
   C. **Characterization Data for New Compounds** S2
      i. Nuclear Magnetic Resonance Spectroscopy S2
      ii. Infrared Spectroscopy S3
      iii. High Resolution Mass Spectrometry S3
      iv. Optical Rotation S3

2. **Experimental Procedures** S4
   A. **General Procedure for the Synthesis of Chiral Allylic Alcohols** S4
   B. **General Procedure for the Synthesis of Achiral Carboxylic Acids** S6
   C. **General Procedure for the Synthesis of Chiral Imides** S7
   D. **General Procedure for the Synthesis of Chiral Alcohols** S9
   E. **General Procedure for the Synthesis of Chiral Alkynes** S11
   F. **General Procedure for the Metallacycle-Mediated Coupling** S12
   G. **General Procedure for the Synthesis of Coupled Chiral Acids** S19

3. **Conformational Analysis of Ligands (2-C, 2-E, 4-C and oleic acid) and Docking to the GPR120 structure (PDB: 8id6)** S24

4. **Biological Procedures** S28

5. **NMR Spectra of Novel Compounds** S47

1. Materials and Methods

A. Reagents and Solvents

All reagents and starting materials were purchased from commercial sources and used as received, unless otherwise indicated. Anhydrous dichloromethane (DCM), tetrahydrofuran (THF), toluene (PhMe), and dimethylformamide (DMF) were obtained by passing HPLC grade solvents through a column of activated alumina using a Glass Contour Solvent Purification System by Pure Process Technology, LLC. For flash chromatography, HPLC grade solvents were used without further purification. Titanium isopropoxide ($Ti(Oi\text{–}Pr)_4$) was distilled prior to use and stored in a foil-wrapped round bottom flask under an atmosphere of nitrogen. *n*-BuLi was purchased from Sigma–Aldrich as a 2.5 M solution in hexanes and used without further titration, NaHMDS and TBAF were purchased from Sigma–Aldrich as 1.0 M solutions in THF and used without further titration, and $LiBH_4$ was purchased from Sigma–Aldrich as a 2.0 M solution in THF and used without further titration. All Grignard reagents were titrated prior to use.

B. Reaction Set-Up and Purification

All reactions were conducted in flame-dried glassware under an atmosphere of dry nitrogen and in anhydrous solvents unless otherwise indicated. Reaction mixtures were magnetically stirred and their progress was monitored by thin layer chromatography (TLC) on EMD TLC silica 60 F_{254} glass-backed plates. TLC plates were visualized by exposure to UV-light (254 nm), followed by staining with *p*-anisaldehyde, potassium permanganate ($KMnO_4$), or cerium ammonium molybdate (CAM).

Purification of crude isolates was achieved by flash column chromatography on a Biotage® Isolera OneTM Automated Liquid Chromatography System using Biotage® Sfar Silica HC D 5–100 g silica gel cartridges, a Biotage® Sfar Silica D 5 g silica gel cartridge, or performed using a forced flow of the indicated solvent system on Sorbent Technologies™ silica gel 60 Å (40–63 µm particle size). Concentration of reaction product solutions and chromatography fractions was accomplished by rotary evaporation at 26–40 °C under the appropriate pressure, followed by concentration at room temperature on a vacuum pump (approx. 0–1 mbar). Yields refer to chromatographically purified and spectroscopically pure compounds unless otherwise indicated.

C. Characterization Data for New Compounds

i. Nuclear Magnetic Resonance Spectroscopy

^{1}H NMR data was recorded on a Bruker Avance III 500 MHz NMR spectrometer (TBI probe) and/or a Bruker Avance III 600 MHz spectrometer (BBFO probe). ^{1}H chemical shifts are reported in parts per million (ppm, δ scale) downfield from tetramethylsilane and are referenced to the residual protium in $CDCl_3$ (7.26 ppm) and C_6D_6 (7.16 ppm). NMR coupling constants are measured in Hertz (Hz), and splitting patterns are indicated as follows: br, broad; s, singlet; d, doublet; dd, doublet of doublets; dt, doublet of triplets; t, triplet; td, triplet of doublets; q, quartet; p, pentet; m, multiplet. ^{13}C {^{1}H decoupled} NMR data were recorded at 150 MHz on a Bruker Avance III 600 MHz spectrometer (BBFO probe). ^{13}C NMR chemical shifts are reported in parts per million (ppm, δ scale) and are referenced to the central line of the carbon resonances of the solvents: $CDCl_3$ (77.16 ppm), C_6D_6 (128.06). Structural assignments for new compounds were supported by two-dimensional NMR experiments (COSY, HSQC, and HMBC) recorded on a Bruker Avance III 600 MHz spectrometer (BBFO probe).

ii. Infrared Spectroscopy

Infrared spectra were collected on a JASCO FT/IR-4100 Fourier Transform Infrared Spectrometer. IR absorption is reported as strong (s), medium (m), weak (w), or broad (br).

iii. High Resolution Mass Spectrometry

HRMS (EI-TOF) analyses were performed at the Mass Spectrometry Laboratory of the University of Illinois at Urbana-Champaign.

iv. Optical Rotation

Optical rotations (α) were obtained on a JASCO-P-2000 polarimeter equipped with tungsten-halogen lamp (WI) and interface filter set to 589 nm, using a sample cell with a pathlength of 100 nm. Specific rotations are reported as: $[\alpha]_{589}^{T(℃)}$ (*c*, solvent) and are based on the equation $[\alpha]_{589}^{T(℃)}$ = (100•α)/(*l*•*c*), where the concentration (*c*) is reported as g/ml and the pathlength (*l*) in decimeters.

2. Experimental Procedures

A. General Procedure for the Synthesis of Chiral Allylic Alcohols*

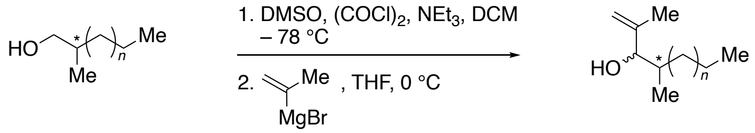

To a stirring solution of DMSO (2.0 equiv) in DCM (0.85 M in DMSO) at –78 °C was added oxalyl chloride (1.5 equiv) slowly dropwise. The solution was allowed to stir 5 min then a solution of the chiral alcohol (1.0 equiv) in DCM (0.43 M in the chiral alcohol) was added dropwise. The solution was allowed to stir for 10 min at –78 °C then triethylamine (4.0 equiv) was added, the ice bath was removed, and the reaction mixture was allowed to warm to room temperature. It was then diluted with DCM and washed sequentially with $KHSO_4$ (1.0 M), a saturated aqueous solution of $NaHCO_3$, then brine. The combined organic phase was dried over Na_2SO_4, filtered, and concentrated *in vacuo*.

The crude aldehyde residue was added dropwise to a flask cooled to 0 °C containing isopropenylmagnesium bromide (1.2 equiv, 0.50 M solution in THF), using THF to aid in transferring (2 mL). The resulting solution was stirred 40 min at 0 °C then a saturated aqueous solution of ammonium chloride was added (1/3 total Grignard volume) and the reaction mixture was extracted with Et_2O. The combined organic phase was dried over Na_2SO_4, filtered, and concentrated *in vacuo*. The crude concentrate was purified on silica by flash column chromatography with a gradient from 5–15% EtOAc in hexanes to afford an inconsequential mixture of allylic alcohol diastereomers as a yellow oil.

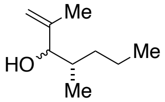

10

Data for compound 10: Yield: 44% (2 steps); **TLC:** R_f = 0.50 (15% EtOAc in hexanes); **^{1}H NMR** (600 MHz, C_6D_6): δ 4.96–4.77 (m, 2H), 3.68–3.53 (m, 1H), 1.73–1.38 (m, 5H), 1.35–1.07 (m, 3H), 0.98–0.78 (m, 7H); **^{13}C NMR** (150 MHz, C_6D_6): δ 147.4, 147.4, 112.0, 111.2, 80.9, 79.1, 36.2, 35.9, 35.6, 34.2, 20.7, 20.6, 18.4, 17.6, 16.4, 14.7, 14.5, 14.0; **IR** (neat, cm^{-1}): 3398 (br), 2963 (s), 2934 (m), 2861 (m), 1645 (w), 1448 (m), 1367 (w), 1021 (m), 893 (m); **HRMS** (ESI-TOF) (*m/z*): $[M+H]^+$ calcd for $C_9H_{19}O$ 143.1436; found, 143.1434.

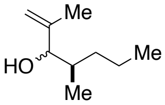

11

---

* The ^{1}H NMR shifts are reported as combined integrations for both diastereomers, meaning, the integrations were done as if only one diastereomer was present. For ^{13}C NMR individual signals are reported for both diastereomers.

Data for compound 11: Yield: 21% (2 steps); **TLC:** R_f = 0.50 (15% EtOAc in hexanes); **^{1}H NMR** (600 MHz, C_6D_6): δ 4.99–4.77 (m, 2H), 3.68–3.53 (m, 1H), 1.73–1.38 (m, 5H), 1.36–1.03 (m, 3H), 0.97–0.77 (m, 7H); **^{13}C NMR** (150 MHz, C_6D_6): δ 147.4, 147.4, 112.0, 111.2, 80.9, 79.1, 36.2, 35.9, 35.6, 34.2, 20.7, 20.6, 18.4, 17.6, 16.4, 14.7, 14.5, 14.0; **IR** (neat, cm^{-1}): 3407 (br), 2955 (s), 2915 (m), 2857 (m), 1652 (w), 1456 (m), 1379 (w), 1026 (m), 903 (m); **HRMS** (ESI-TOF) (*m/z*): $[M+H]^+$ calcd for $C_9H_{19}O$ 143.1436; found, 143.1442.

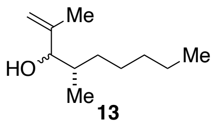

13

Data for compound 13: Yield: 60% (2 steps); **TLC:** R_f = 0.45 (15% EtOAc in hexanes); **^{1}H NMR** (600 MHz, $CDCl_3$): δ 4.95–4.85 (m, 2H), 3.88–3.74 (m, 1H), 1.73–1.66 (m, 3H), 1.65–1.03 (m, 10H), 0.92–0.80 (m, 6H); **^{13}C NMR** (150 MHz, $CDCl_3$): δ 147.0, 146.9, 112.5, 111.5, 81.1, 79.5, 36.0, 35.6, 33.7, 32.4, 32.2, 31.7, 27.0, 26.9, 22.9, 22.8, 18.5, 17.6, 16.3, 14.2, 14.2, 13.9; **IR** (neat, cm^{-1}): 3380 (br), 2952 (m), 2926 (s), 2853 (m), 1652 (w), 1469 (w), 1382 (w), 1017 (w), 901 (w); **HRMS** (ESI-TOF) (*m/z*): $[M]^+$ calcd for $C_{11}H_{22}O$ 170.1671; found, 170.1669.

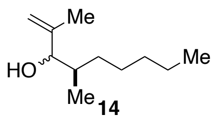

14

Data for compound 14: Yield: 56% (2 steps); **TLC:** R_f = 0.45 (15% EtOAc in hexanes); **^{1}H NMR** (600 MHz, $CDCl_3$): δ 4.97–4.84 (m, 2H), 3.90–3.73 (m, 1H), 1.73–1.67 (m, 3H), 1.65–1.03 (m, 10H), 0.94–0.80 (m, 6H); **^{13}C NMR** (150 MHz, $CDCl_3$): δ 147.0, 146.9, 112.5, 111.5, 81.1, 79.5, 36.0, 35.6, 33.7, 32.4, 32.2, 31.7, 27.0, 26.9, 22.9, 22.8, 18.5, 17.6, 16.3, 14.3, 14.2, 13.9; **IR** (neat, cm^{-1}): 3383 (br), 2959 (s), 2923 (s), 2853 (m), 1653 (w), 1455 (m), 1371 (w), 1010 (w), 901 (m); **HRMS** (ESI-TOF) (*m/z*): $[M+H]^+$ calcd for $C_{11}H_{23}O$ 171.1749; found, 171.1746.

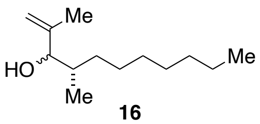

16

Data for compound 16: Yield: 85% (2 steps); **TLC:** R_f = 0.54 (15% EtOAc in hexanes); **^{1}H NMR** (600 MHz, $CDCl_3$): δ 4.95–4.86 (m, 2H), 3.88–3.75 (m, 1H), 1.74–1.67 (m, 3H), 1.66–1.03 (m, 14H), 0.93–0.80 (m, 6H); **^{13}C NMR** (150 MHz, $CDCl_3$): δ 147.0, 146.9, 112.5, 111.5, 81.1, 79.5, 36.0, 35.6, 33.7, 32.1, 32.0, 31.7, 30.1, 30.0, 29.5, 29.5, 27.4, 27.3, 22.8, 22.8, 18.5, 17.7, 16.3, 14.3, 14.3, 13.9; **IR** (neat, cm^{-1}): 3382 (br), 2955 (s), 2919 (s), 2857 (s), 1649 (w), 1459 (m), 1379 (w), 1019 (w), 891 (m); **HRMS** (ESI-TOF) (*m/z*): $[M]^+$ calcd for $C_{13}H_{26}O$ 198.1984; found, 198.1979.

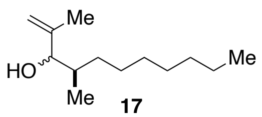

17

Data for compound 17: Yield: 50% (2 steps); **TLC:** R_f = 0.54 (15% EtOAc in hexanes); **^{1}H NMR** (600 MHz, $CDCl_3$): δ 4.95–4.85 (m, 2H), 3.88–3.75 (m, 1H), 1.74–1.67 (m, 3H), 1.66–1.03 (m, 14H), 0.92–0.80 (m, 6H); **^{13}C NMR** (150 MHz, $CDCl_3$): δ 147.0, 146.9, 112.5, 111.5, 81.1, 79.5, 36.0, 35.6, 33.7, 32.1, 32.0, 31.7, 30.1, 30.0, 29.5, 29.5, 27.4, 27.3, 22.8, 22.8, 18.5, 17.7, 16.3, 14.3, 14.3, 13.9; **IR** (neat, cm^{-1}): 3401 (br), 3076 (w), 2967 (s), 2923 (s), 2861 (s), 1649 (w), 1451 (m), 1375 (m), 1021 (m), 907 (m); **HRMS** (ESI-TOF) (*m/z*): $[M]^+$ calcd for $C_{13}H_{26}O$ 198.1984; found, 198.1983.

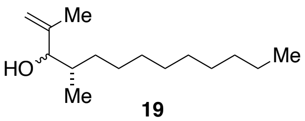

19

Data for compound 19: Yield: 77% (2 steps); **TLC:** R_f = 0.56 (15% EtOAc in hexanes); **^{1}H NMR** (600 MHz, $CDCl_3$): δ 4.95–4.86 (m, 2H), 3.88–3.75 (m, 1H), 1.73–1.68 (m, 3H), 1.65–1.02 (m, 18H), 0.92–0.80 (m, 6H); **^{13}C NMR** (150 MHz, $CDCl_3$): δ 147.0, 146.9, 112.5, 111.5, 81.1, 79.5, 36.0, 35.6, 33.7, 32.1, 32.1, 31.7, 30.2, 30.0, 30.0, 29.9, 29.8, 29.8, 29.8, 29.5, 29.5, 27.4, 27.2, 22.8, 18.5, 17.7, 16.3, 14.3, 14.3, 13.9, ; **IR** (neat, cm^{-1}): 3398 (br), 2955 (s), 2923 (s), 2850 (s), 1649 (w), 1459 (m), 1382 (w), 1013 (m), 981 (w), 903 (m); **HRMS** (ESI-TOF) (*m/z*): $[M]^+$ calcd for $C_{15}H_{30}O$ 226.2297; found, 226.2293.

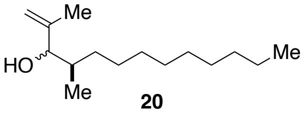

20

Data for compound 20: Yield: 80% (2 steps); **TLC:** R_f = 0.56 (15% EtOAc in hexanes); **^{1}H NMR** (600 MHz, $CDCl_3$): δ 4.96–4.86 (m, 2H), 3.88–3.74 (m, 1H), 1.73–1.68 (m, 3H), 1.65–1.56 (m, 1H), 1.47–1.02 (m, 17 H), 0.92–0.81 (m, 6H); **^{13}C NMR** (150 MHz, $CDCl_3$): δ 147.0, 146.9, 112.5, 111.5, 81.1, 79.5, 36.0, 35.6, 33.7, 32.1, 32.1, 31.7, 30.2, 30.0, 30.0, 29.9, 29.8, 29.8, 29.8, 29.5, 29.5, 27.4, 27.2, 22.8, 18.5, 17.6, 16.3, 14.3, 14.3, 13.9; **IR** (neat, cm^{-1}): 3382 (br), 2966 (s), 2926 (s), 2857 (s), 1652 (w), 1452 (m), 1376 (m), 1005 (m), 975 (w), 895 (m), 753 (m); **HRMS** (ESI-TOF) (*m/z*): $[M]^+$ calcd for $C_{15}H_{30}O$ 226.2297; found, 226.2298.

B. General Procedure for the Synthesis of Achiral Carboxylic Acids

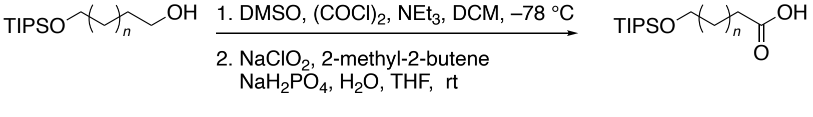

To a stirring solution of DMSO (2.0 equiv) in DCM (0.85 M in DMSO) at –78 °C was added oxalyl chloride (1.5 equiv) slowly dropwise. The solution was allowed to stir 5 min then a solution of the chiral alcohol (1.0 equiv) in DCM (0.43 M in the chiral alcohol) was added dropwise. The solution was allowed to stir for 10 min at –78 °C then triethylamine (4.0 equiv) was added, the ice

bath was removed, and the reaction mixture was allowed to warm to room temperature. It was diluted with DCM and washed sequentially with $KHSO_4$ (1.0 M), a saturated aqueous solution of $NaHCO_3$, then brine. The combined organic phase was dried over Na_2SO_4, filtered, and concentrated *in vacuo*.

The crude aldehyde residue was dissolved in THF (0.13 M in the aldehyde) and 2-methyl-2-butene (3.0 equiv) was added to the solution. $NaClO_2$ (3.0 equiv) and NaH_2PO_4 (3.0 equiv) were dissolved in water (0.78 M in $NaClO_2$) and the solution was added dropwise to the reaction mixture at room temperature. After 30 min of stirring, most of the organic phase was removed *via* decantation then the remaining mixture was extracted with DCM. The combined organic phase was dried over $MgSO_4$, filtered, and concentrated *in vacuo*. The crude concentrate was purified on silica by flash column chromatography with a gradient from 0–15% EtOAc in hexanes to afford the desired carboxylic acid as a colorless oil.

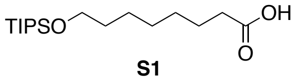

S1

Data for compound S1: Yield: 95% (2 steps); **TLC:** $R_f = 0.38$ (15% EtOAc in hexanes); **^{1}H NMR** (600 MHz, $CDCl_3$): δ 11.9–9.67 (br, 1H), 3.66 (t, *J* = 6.6 Hz, 2H), 2.35 (t, *J* = 7.5 Hz, 2H), 1.64 (p, *J* = 7.4 Hz, 2H), 1.53 (p, *J* = 7.3 Hz, 2H), 1.39–1.30 (m, 6H), 1.11–1.03 (m, 21H); **^{13}C NMR** (150 MHz, $CDCl_3$): δ 179.3, 63.6, 34.0, 33.1, 29.2, 29.2, 25.8, 24.8, 18.2, 12.2; **IR** (neat, cm^{-1}): 3100 (br), 2949 (s), 2865 (s), 1716 (s), 1452 (w), 1271 (w), 1096 (m), 884 (m); **HRMS** (ESI-TOF) (*m/z*): $[M+H]^+$ calcd for $C_{17}H_{37}O_3Si$ 317.2512; found, 317.2505.

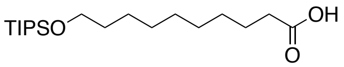

S2

Data for compound S2: Yield: 86% (2 steps); **TLC:** $R_f = 0.41$ (15% EtOAc in hexanes); **^{1}H NMR** (600 MHz, $CDCl_3$): δ 11.38–10.34 (br, 1H), 3.66 (t, *J* = 6.3 Hz, 2H), 2.35 (t, *J* = 7.7 Hz, 2H), 1.63 (p, *J* = 7.3 Hz, 2H), 1.53 (p, *J* = 7.4 Hz, 2H), 1.37–1.27 (m, 10H), 1.13–1.00 (m, 21H); **^{13}C NMR** (150 MHz, $CDCl_3$): δ 179.6, 63.6, 34.1, 33.2, 29.6, 29.5, 29.3, 29.2, 25.9, 24.8, 18.2, 12.2; **IR** (neat, cm^{-1}): 3084 (br), 2929 (s), 2845 (s), 1703 (s), 1451 (w), 1258 (w), 1091 (m), 877 (w); **HRMS** (ESI-TOF) (*m/z*): $[M+H]^+$ calcd for $C_{19}H_{41}O_3Si$ 345.2825; found, 345.2809.

C. General Procedure for the Synthesis of Chiral Imides

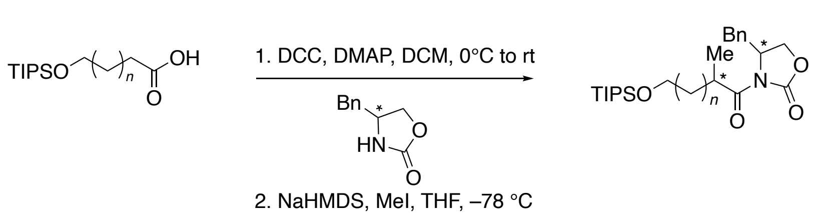

To a stirring solution of the carboxylic acid (1.0 equiv) in DCM (0.75 M in the carboxylic acid) was added the desired chiral 4-benzyl-2-oxazolidinone (1.0 equiv) and DMAP (0.13 equiv). The reaction mixture was cooled to 0 °C and DCC (1.0 equiv) was added in one portion. After 10 min of stirring at 0 °C, the solution was allowed to warm to room temperature and stirred overnight (15–22 h). The dicyclohexylurea formed was then removed *via* filtration through a fritted funnel, and the precipitate was washed with DCM. The filtrate was washed with a saturated aqueous

solution of $NaHCO_3$, dried over Na_2SO_4, and concentrated *in vacuo*. The resulting residue was then filtered through a pad of silica with DCM to remove any remaining unreacted chiral auxillary starting material, and the filtrate was once again concentrated *in vacuo*.

The resulting residue was azeotroped 3 times with anhydrous benzene, then dissolved in THF (0.26 M in the starting carboxylic acid) and cooled to –78 °C. NaHMDS (1.2 equiv, 1.0 M solution in THF) was then added dropwise and the resulting solution was stirred for 1 h at –78 °C. MeI (2.0 equiv) was then added and the reaction mixture was stirred for an additional 4 h at –78 °C. Finally, a saturated aqueous solution of NH_4Cl (1/4 total THF volume) was added, and the resulting mixture was allowed to warm to room temperature. The mixture was concentrated *in vacuo* to remove most of the solvent, then the remaining residue was extracted with EtOAc and the combined organic phase was washed with brine, dried over Na_2SO_4, filtered, and concentrated *in vacuo*. The crude concentrate was purified on silica by flash column chromatography with a gradient from 0–9% EtOAc in hexanes to afford the desired auxillary-containing chiral imide as a colorless oil.

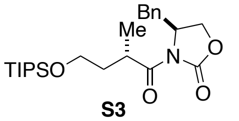

S3

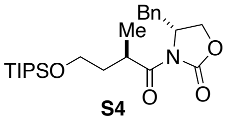

S4

Data for compound S3 and S4: Yield: 47% (**S3**), 41% (**S4**) – (2 steps); **TLC:** R_f = 0.46 (15% EtOAc in hexanes); **^{1}H NMR** (600 MHz, $CDCl_3$): δ 7.33 (t, *J* = 7.6 Hz, 2H), 7.28 (app. d, *J* = 7.8 Hz, 1H), 7.22 (d, *J* = 7.2 Hz, 2H), 4.68–4.64 (m, 1H), 4.16 (d, *J* = 4.7 Hz, 2H), 3.95–3.86 (m, 1H), 3.78–3.69 (m, 2H), 3.26 (dd, *J* = 13.4, 3.4 Hz, 1H), 2.77 (dd, *J* = 13.4, 9.6 Hz, 1H), 2.12–2.02 (m, 1H), 1.70–1.64 (m, 1H), 1.26 (d, *J* = 6.9 Hz, 3H), 1.07–1.02 (m, 21H); **^{13}C NMR** (150 MHz, $CDCl_3$): δ 177.1, 153.0, 135.5, 129.6, 129.1, 127.5, 66.1, 61.4, 55.5, 38.1, 36.2, 34.8, 18.1, 17.8, 12.1; **IR** (neat, cm^{-1}): 2400 (br), 2935 (s), 2871 (s), 1766 (s), 1690 (m), 1459 (w), 1375 (m), 1202 (w), 1106 (m); **HRMS** (ESI-TOF) (*m/z*): $[M+H]^+$ calcd for $C_{24}H_{40}NO_4Si$ 434.2727; found, 434.2720 (**S3**), 434.2732 (**S4**); $[\alpha]_{589}^{22.0}$: (**S3**) +53.1 (c = 0.63, $CHCl_3$), $[\alpha]_{589}^{22.0}$: (**S4**) –50.8 (c = 0.47, $CHCl_3$).

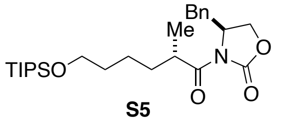

S5

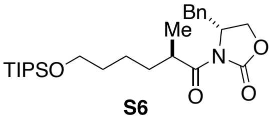

S6

Data for compound S5 and S6: Yield: 72% (**S5**), 48% (**S6**) – (2 steps); **TLC:** R_f = 0.47 (15% EtOAc in hexanes); **^{1}H NMR** (600 MHz, $CDCl_3$): δ 7.33 (t, *J* = 7.6 Hz, 2H), 7.28 (app. d, *J* = 7.8 Hz, 1H), 7.22 (d, *J* = 7.2 Hz, 2H), 4.68–4.64 (m, 1H), 4.21–4.15 (m, 2H), 3.75–3.70 (m, 1H), 3.67 (td, *J* = 6.5, 2.2 Hz, 2H), 3.27 (dd, *J* = 13.4, 3.4 Hz, 1H), 2.77 (dd, *J* = 13.4, 9.6 Hz, 1H), 1.80–1.72 (m, 1H), 1.60–1.50 (m, 2H), 1.49–1.30 (m, 3H), 1.22 (d, *J* = 6.9 Hz, 3H), 1.07–1.02 (m, 21H); **^{13}C NMR** (150 MHz, $CDCl_3$): δ 177.4, 153.2, 135.5, 129.6, 129.1, 127.5, 66.1, 66.3, 55.5, 38.1, 37.8, 33.4, 33.1, 23.7, 18.2, 17.5, 12.1; **IR** (neat, cm^{-1}): 3400 (br), 2941 (s), 2861 (s), 1780 (s), 1704 (s), 1459 (m), 1382 (s), 1349 (m), 1287 (w), 1244 (m), 1207 (m), 1097 (s), 1010 (w), 882 (m), 692 (m); **HRMS** (ESI-TOF) (*m/z*): $[M+H]^+$ calcd for $C_{26}H_{44}NO_4Si$ 462.3040; found, 462.3041 (**S5**), 462.3037 (**S6**); $[\alpha]_{589}^{22.4}$: (**S5**) +47.3 (c = 0.37, $CHCl_3$), $[\alpha]_{589}^{21.2}$: (**S6**) –47.1 (c = 0.70, $CHCl_3$).

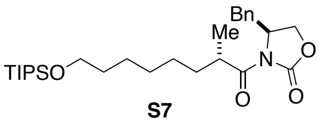

S7

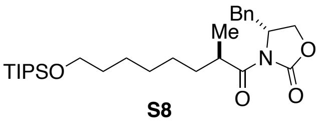

S8

Data for compound S7 and S8: Yield: 58% (**S7**), 57% (**S8**) – (2 steps); **TLC:** R_f = 0.48 (15% EtOAc in hexanes); **^{1}H NMR** (600 MHz, $CDCl_3$): δ 7.33 (t, *J* = 7.6 Hz, 2H), 7.28 (app. d, *J* = 7.8 Hz, 1H), 7.22 (d, *J* = 7.2 Hz, 2H), 4.68–4.64 (m, 1H), 4.21–4.15 (m, 2H), 3.74–3.68 (m, 1H), 3.66 (t, *J* = 6.6 Hz, 2H), 3.27 (dd, *J* = 13.4, 3.3 Hz, 1H), 2.76 (dd, *J* = 13.3, 9.6 Hz, 1H), 1.77–1.70 (m, 1H), 1.56–1.50 (m, 2H), 1.45–1.38 (m, 1H), 1.37–1.26 (m, 6H), 1.22 (d, *J* = 6.8 Hz, 3H), 1.07–1.02 (m, 21H); **^{13}C NMR** (150 MHz, $CDCl_3$): δ 177.5, 153.2, 135.5, 129.6, 129.1, 127.5, 66.1, 63.6, 55.5, 38.1, 37.9, 33.5, 33.1, 29.7, 27.4, 25.9, 18.2, 17.5, 12.2; **IR** (neat, cm^{-1}): 3355 (br), 2929 (s), 2858 (m), 1781 (s), 1690 (m), 1465 (w), 1374 (w), 1226 (w), 1096 (m); **HRMS** (ESI-TOF) (*m/z*): $[M+H]^+$ calcd for $C_{28}H_{48}NO_4Si$ 490.3353; found, 490.3359 (**S7**), 490.3355 (**S8**); $[\alpha]_{589}^{22.6}$: (**S7**) +41.3 (c = 0.96, $CHCl_3$), $[\alpha]_{589}^{21.8}$: (**S8**) –45.4 (c = 0.40, $CHCl_3$).

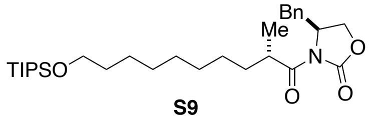

S9

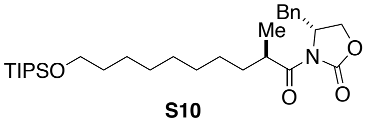

S10

Data for compound S9 and S10: Yield: 62% (**S9**), 67% (**S10**) – (2 steps); **TLC:** R_f = 0.49 (15% EtOAc in hexanes); **^{1}H NMR** (600 MHz, $CDCl_3$): δ 7.33 (t, *J* = 7.6 Hz, 2H), 7.28 (app. d, *J* = 7.8 Hz, 1H), 7.22 (d, *J* = 7.2 Hz, 2H), 4.70–4.65 (m, 1H), 4.22–4.15 (m, 2H), 3.73–3.68 (m, 1H), 3.66 (t, *J* = 6.7 Hz, 2H), 3.27 (dd, *J* = 13.4, 3.3 Hz, 1H), 2.76 (dd, *J* = 13.4, 9.6 Hz, 1H), 1.77–1.70 (m, 1H), 1.56–1.50 (m, 2H), 1.44–1.37 (m, 1H), 1.35–1.25 (m, 10H), 1.22 (d, *J* = 6.8 Hz, 3H), 1.07–1.02 (m, 21H); **^{13}C NMR** (150 MHz, $CDCl_3$): δ 177.5, 153.2, 135.5, 129.6, 129.1, 127.5, 66.1, 63.6, 55.5, 38.1, 37.9, 33.6, 33.2, 29.8, 29.7, 29.6, 27.4, 26.0, 18.2, 17.8, 12.2; **IR** (neat, cm^{-1}): 3387 (br), 2935 (s), 2851 (s), 1781 (s), 1690 (w), 1471 (w), 1388 (m), 1220 (w), 1096 (m); **HRMS** (ESI-TOF) (*m/z*): $[M+H]^+$ calcd for $C_{30}H_{52}NO_4Si$ 518.3666; found, 518.3666; $[\alpha]_{589}^{22.0}$: (**S9**) +42.4 (c = 0.41, $CHCl_3$), $[\alpha]_{589}^{22.5}$: (**S10**) –39.2 (c = 0.55, $CHCl_3$).

D. General Procedure for the Synthesis of Chiral Alcohols

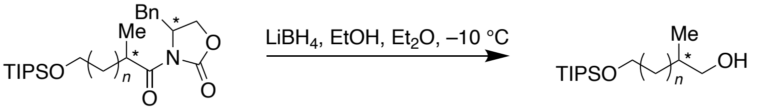

To a –10 °C stirring solution of the chiral imide (1.0 equiv) in ethanol (6.9 M in the chiral imide) and diethyl ether (0.27 M in the chiral imide) was added lithium borohydride (2.4 equiv, 2.0 M solution in THF). The resulting mixture was stirred for 1.5 h at –10 °C then warmed to 0 °C and stirred an additional 30 min. Finally, 1 M NaOH (1/3 total EtOH and Et_2O volume) was added and the mixture was stirred 15 min at –10 °C before extracting with diethyl ether. The combined organic layers were washed with brine, dried over $MgSO_4$, filtered, and concentrated *in vacuo*. The crude concentrate was purified on silica by flash column chromatography with a gradient from 5–15% EtOAc in hexanes to afford the desired chiral alcohol as a colorless oil.

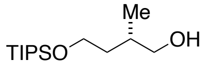

S11

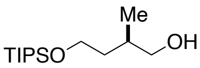

S12

Data for compound S11 and S12: Yield: 98% (**S11**), 87% (**S12**); **TLC:** R_f = 0.31 (15% EtOAc in hexanes); **^{1}H NMR** (600 MHz, $CDCl_3$): δ 3.84 (p, *J* = 5.3 Hz, 1H), 3.74 (dt, *J* = 10.3, 6.0 Hz, 1H), 3.55–3.50 (m, 1H), 3.46–3.41 (m, 1H), 3.08–3.03 (m, 1H), 1.87–1.79 (m, 1H), 1.58 (q, *J* = 5.9 Hz, 2H), 1.11–1.00 (m, 21H), 0.92 (d, *J* = 6.9 Hz, 3H); **^{13}C NMR** (150 MHz, $CDCl_3$): δ 68.4, 62.2, 37.9, 34.8, 18.1, 17.6, 12.1; **IR** (neat, cm^{-1}): 3355 (br), 2935 (s), 2858 (s), 1645 (w), 1465 (w), 1103 (m); **HRMS** (ESI-TOF) (*m/z*): $[M+H]^+$ calcd for $C_{14}H_{33}O_2Si$ 261.2250; found, 261.2238 (**S11**), 261.2250 (**S12**); $[\alpha]^{22.8}_{589}$: (**S11**) –9.7 (c = 0.32, $CHCl_3$), $[\alpha]^{22.1}_{589}$: (**S12**) +10.0 (c = 0.45, $CHCl_3$).

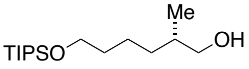

S13

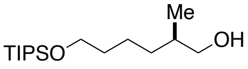

S14

Data for compound S13 and S14: Yield: 80% (**S13**), 84% (**S14**); **TLC:** R_f = 0.36 (15% EtOAc in hexanes); **^{1}H NMR** (500 MHz, $CDCl_3$): δ 3.68 (t, *J* = 6.5 Hz, 2H), 3.51 (dd, 10.5, 5.7 Hz, 1H), 3.42 (dd, *J* = 10.5, 6.5 Hz, 1H), 1.67–1.26 (m, 7H), 1.17–1.03 (m, 22H), 0.92 (d, *J* = 6.7 Hz, 3H); **^{13}C NMR** (150 MHz, $CDCl_3$): δ 68.5, 63.5, 35.9, 33.4, 33.1, 23.3, 18.2, 16.7, 12.2; **IR** (neat, cm^{-1}): 3335 (br), 2930 (s), 2861 (s), 1455 (m), 1379 (w), 1247 (w), 1116 (m), 1050 (m), 889 (m); **HRMS** (ESI-TOF) (*m/z*): $[M+H]^+$ calcd for $C_{16}H_{37}O_2Si$ 289.2563; found, 289.2559 (**S13**), 289.2563 (**S14**); $[\alpha]^{20.9}_{589}$: (**S13**) –6.8 (c = 0.48, $CHCl_3$), $[\alpha]^{21.3}_{589}$: (**S14**) +7.9 (c = 0.40, $CHCl_3$).

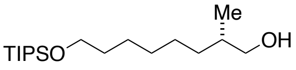

S15

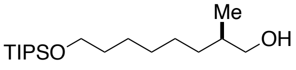

S16

Data for compound S15 and S16: Yield: 98% (**S15**), 87% (**S16**); **TLC:** R_f = 0.33 (15% EtOAc in hexanes); **^{1}H NMR** (600 MHz, $CDCl_3$): δ 3.66 (t, *J* = 6.7 Hz, 2H), 3.53–3.48 (m, 1H), 3.44–3.38 (m, 1H), 1.64–1.57 (m, 1H), 1.54 (p, *J* = 7.0 Hz, 2H), 1.44–1.23 (m, 8H), 1.14–1.00 (m, 22H), 0.91 (d, *J* = 6.7 Hz, 3H); **^{13}C NMR** (150 MHz, $CDCl_3$): δ 68.6, 63.6, 35.9, 33.2, 33.2, 29.9, 27.1, 26.0, 18.2, 16.7, 12.2; **IR** (neat, cm^{-1}): 3332 (br), 2928 (s), 2858 (s), 1452 (w), 1113 (s), 1029 (w), 875 (w); **HRMS** (ESI-TOF) (*m/z*): $[M+H]^+$ calcd for $C_{18}H_{41}O_2Si$ 317.2876; found, 317.2874; $[\alpha]^{22.1}_{589}$: (**S15**) –5.6 (c = 0.40, $CHCl_3$), $[\alpha]^{22.8}_{589}$: (**S16**) +5.5 (c = 0.54, $CHCl_3$).

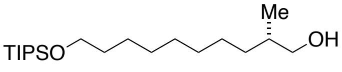

S17

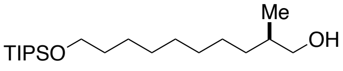

S18

Data for compound S17 and S18: Yield: 93% (**S17**), 92% (**S18**); **TLC:** R_f = 0.33 (15% EtOAc in hexanes); **^{1}H NMR** (600 MHz, $CDCl_3$): δ 3.66 (t, *J* = 6.7 Hz, 2H), 3.50 (dd, *J* = 10.5, 5.8 Hz, 1H), 3.41 (dd, *J* = 10.5, 6.6 Hz, 1H), 1.64–1.57 (m, 1H), 1.56–1.50 (m, 2H), 1.39–1.26 (m, 12H), 1.11–1.02 (m, 22H), 0.91 (d, *J* = 6.7 Hz, 3H); **^{13}C NMR** (150 MHz, $CDCl_3$): δ 68.6, 63.7, 35.9, 33.32, 33.2, 30.0, 29.8, 29.6, 27.1, 26.0, 18.2, 16.7, 12.2; **IR** (neat, cm^{-1}): 3352 (br), 2928 (s), 2851 (s), 1645 (w), 1459 (m), 1099 (m), 1036 (w), 779 (w); **HRMS** (ESI-TOF) (*m/z*): $[M+H]^+$ calcd for $C_{20}H_{45}O_2Si$ 345.3189; found, 345.3184 (**S17**), 345.3188 (**S18**); $[\alpha]^{21.5}_{589}$: (**S17**) –6.1 (c = 0.42, $CHCl_3$), $[\alpha]^{22.7}_{589}$: (**S18**) +4.7 (c = 0.32, $CHCl_3$).

E. General Procedure for the Synthesis of Chiral Alkynes

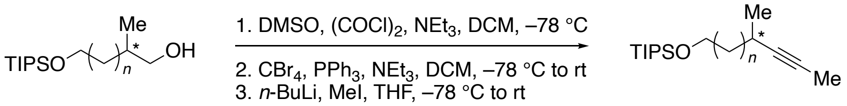

To a stirring solution of DMSO (2.0 equiv) in DCM (0.85 M in DMSO) at –78 °C was added oxalyl chloride (1.5 equiv) slowly dropwise. The solution was allowed to stir 5 min, then a solution of the chiral alcohol (1.0 equiv) in DCM (0.43 M in the chiral alcohol) was added dropwise. The resulting mixture was stirred for 10 min at –78 °C then triethylamine (4.0 equiv) was added, the ice bath was removed, and the reaction mixture was allowed to warm to room temperature. It was then diluted with DCM and washed sequentially with $KHSO_4$ (1.0 M), a saturated aqueous solution of $NaHCO_3$, then brine. The combined organic phase was dried over Na_2SO_4, filtered, and concentrated *in vacuo*.

PPh_3 (4.1 equiv) was dissolved in DCM (0.14 M in the chiral alcohol), and then CBr_4 (2.0 equiv) was added, and the resulting mixture was cooled to –78 °C after a homogenous solution was obtained. To this solution, at –78 °C, was added dropwise a solution of the crude aldehyde in DCM (0.43 M in the chiral alcohol) then triethylamine (1.0 equiv), and the resulting solution was stirred 30 min at –78 °C, then warmed to room temperature and stirred and additonal 10 min. The reaction mixture was then diluted with hexanes (2/3 total DCM volume) and filtered through a short pad of silica. The silica was washed with a 25% solution of EtOAc in hexanes and the organic phase was concentrated *in vacuo*. The concentrate was then run through another short pad of silica with hexanes and the organic phase was concentrated *in vacuo* to give the expected dibromo olefin intermediate.

The dibromo olefin was then azeotroped with benzene, subsequently dissolved in THF (0.11 M in the chiral alcohol), and cooled to –78 °C. To this solution at –78 °C was added *n*-BuLi (2.0 equiv, 2.5 M solution in hexanes) and the reaction mixture was then warmed to –20 °C and stirred 1.5 h. After this time period, MeI (5.0 equiv) was added dropwise and the solution was stirred 10 min more at –20 °C then warmed to 0 °C and stirred 1 h before warming to room temperature and stirring overnight (14–19 h). The reaction was then quenched with a saturated aqueous solution of NH_4Cl (1/2 total THF volume) and the aqueous phase was extracted with diethyl ether. The combined organic phase was dried over Na_2SO_4, filtered, and concentrated *in vacuo*. The crude concentrate was purified on silica by flash column chromatography with a gradient from 0–5% EtOAc in hexanes to afford the desired chiral alkyne as a colorless oil.

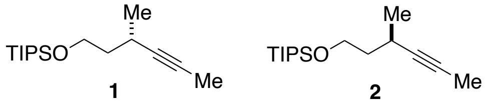

1 2

Data for compound 1 and 2: Yield: 96% (**1**), 80% (**2**) – (3 steps); **TLC:** R_f = 0.33 (2% EtOAc in hexanes); **^{1}H NMR** (600 MHz, $CDCl_3$): δ 3.80 (t, *J* = 6.3 Hz, 2H), 2.64–2.55 (m, 1H), 1.78 (d, *J* = 2.4 Hz, 3H), 1.67–1.57 (m, 2H), 1.15 (d, *J* = 7.0 Hz, 3H), 1.13–1.04 (m, 21H); **^{13}C NMR** (150 MHz, $CDCl_3$): δ 83.8, 75.8, 61.5, 40.5, 22.5, 21.6, 18.2, 12.2, 3.6; **IR** (neat, cm^{-1}): 2929 (s), 2858 (s), 1632 (w), 1451 (w), 1103 (m); **HRMS** (ESI-TOF) (*m/z*): $[M-H]^+$ calcd for $C_{16}H_{31}OSi$ 267.2144; found, 267.2140 (**1**), 267.2144 (**2**); $[\alpha]_{589}^{22.2}$: (**1**) +31.6 (c = 0.37, $CHCl_3$), $[\alpha]_{589}^{22.1}$: (**2**) –34.5 (c = 0.42, $CHCl_3$).

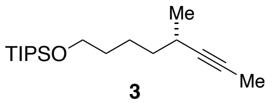

3

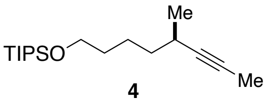

4

Data for compound 3 and 4: Yield: 99% (**3**), 90% (**4**) – (3 steps); **TLC:** R_f = 0.56 (2% EtOAc in hexanes); **^{1}H NMR** (600 MHz, $CDCl_3$): δ 3.68 (t, *J* = 6.5 Hz, 2H), 2.40–2.33 (m, 1H), 1.78 (d, *J* = 2.5 Hz, 3H), 1.60–1.48 (m, 3H), 1.45–1.35 (m, 3H), 1.12 (d, *J* = 7.0 Hz, 3H), 1.10–1.03 (m, 21H); **^{13}C NMR** (150 MHz, $CDCl_3$): δ 84.1, 75.6, 63.5, 37.3, 33.0, 26.1, 23.9, 21.6, 18.2, 12.2, 3.6; **IR** (neat, cm^{-1}): 2934 (s), 2861 (s), 1467 (m), 1379 (w), 1244 (w), 1116 (m), 885 (m); **HRMS** (ESI-TOF) (*m/z*): (**3**) $[M+H]^+$ calcd for $C_{18}H_{37}OSi$ 297.2614; found, 297.2609, (**4**) $[M-H]^+$ calcd for $C_{18}H_{35}OSi$ 295.2457; found, 295.2456; $[\alpha]_{589}^{21.7}$: (**3**) +20.3 (c = 0.30, $CHCl_3$), $[\alpha]_{589}^{22.1}$: (**4**) –20.8 (c = 0.53, $CHCl_3$).

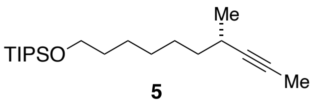

5

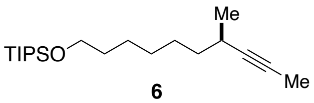

6

Data for compound 5 and 6: Yield: 84% (**5**), 91% (**6**) – (3 steps); **TLC:** R_f = 0.40 (2% EtOAc in hexanes); **^{1}H NMR** (600 MHz, $CDCl_3$): δ 3.67 (t, *J* = 6.6 Hz, 2H), 2.40–2.30 (m, 1H), 1.79 (d, *J* = 2.4 Hz, 3H), 1.54 (p, *J* = 7.0 Hz, 2H), 1.50–1.41 (m, 1H), 1.40–1.28 (m, 7H), 1.12 (d, *J* = 6.9 Hz, 3H), 1.11–1.02 (m, 21H); **^{13}C NMR** (150 MHz, $CDCl_3$): δ 84.2, 75.5, 63.7, 37.4, 33.2, 29.5, 27.6, 26.1, 25.9, 21.6, 18.2, 12.2, 3.7; **IR** (neat, cm^{-1}): 2949 (s), 2865 (s), 1451 (w), 1096 (m), 897 (w); **HRMS** (ESI-TOF) (*m/z*): $[M+H]^+$ calcd for $C_{20}H_{41}OSi$ 325.2927; found, 325.2914 (**5**), 325.2913 (**6**); $[\alpha]_{589}^{22.1}$: (**5**) +22.5 (c = 0.39, $CHCl_3$), $[\alpha]_{589}^{21.9}$: (**6**) –21.4 (c = 0.52, $CHCl_3$).

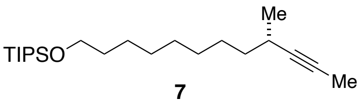

7

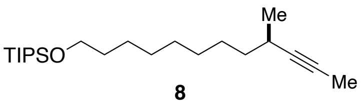

8

Data for compound 7 and 8: Yield: 76% (**7**), 72% (**8**) – (3 steps); **TLC:** R_f = 0.35 (2% EtOAc in hexanes); **^{1}H NMR** (600 MHz, $CDCl_3$): δ 3.66 (t, *J* = 6.7 Hz, 2H), 2.38–2.31 (m, 1H), 1.79 (d, *J* = 2.4 Hz, 3H), 1.58–1.50 (m, 2H), 1.44–1.25 (m, 12H), 1.12 (d, *J* = 6.9 Hz, 3H), 1.10–1.04 (m, 21H); **^{13}C NMR** (150 MHz, $CDCl_3$): δ 84.3, 75.5, 63.7, 37.5, 33.2, 29.8, 29.6, 27.6, 26.1, 26.0, 21.6, 18.8, 18.2, 12.2, 3.7; **IR** (neat, cm^{-1}): 2928 (s), 2851 (s), 1452 (w), 1099 (m); **HRMS** (ESI-TOF) (*m/z*): $[M+H]^+$ calcd for $C_{22}H_{45}OSi$ 353.3240; found, 353.3228 (**7**), 353.3225 (**8**); $[\alpha]_{589}^{22.6}$: (**7**) +19.1 (c = 0.40, $CHCl_3$), $[\alpha]_{589}^{22.0}$: (**8**) –16.4 (c = 0.41, $CHCl_3$).

F. General Procedure for the Metallacycle-Mediated Coupling

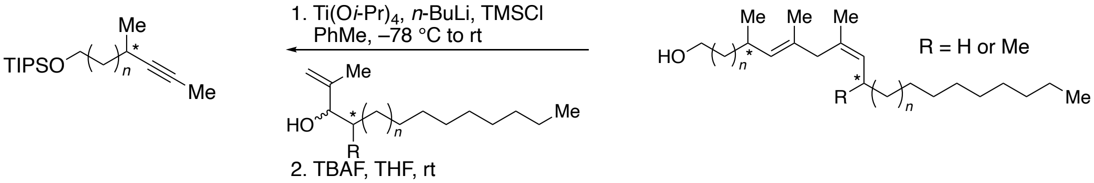

To a stirring solution of the alkyne (2.5 equiv) in toluene (0.40 M in the alkyne) was added $Ti(O\textit{i}\text{–}Pr)_4$ (2.5 equiv). The reaction flask was cooled to –78 °C, *n*-BuLi (5.0 equiv, 2.5 M solution in hexanes) was added dropwise, and the resulting mixture was first allowed to warm to room temperature then heated at 50 °C for 1 h. The solution was then cooled to room temperature, then to –78 °C, and TMSCl (6.0 equiv) was added. The resulting mixture was stirred for 1 h at –78 °C. Meanwhile in a separate flask, the allylic alcohol (1.0 equiv) was dissolved in toluene (0.20 M in the allylic alcohol) and cooled to –78 °C before *n*-BuLi (1.0 equiv, 2.5 M in hexanes) was added dropwsie. The resulting solution containing intermediate alkoxide was warmed to room temperature and then was added dropwise at –78 °C to the Ti-alkyne complex after it had been stirring with TMSCl for 1h. The cooling bath was packed with dry ice and the reaction was then allowed to stir overnight with slow warming to room temperature (17–21 h). The reaction was then quenched with a saturated aqueous solution of NH_4Cl (1x the total toluene volume) and the resulting mixture was stirred for 20 min at room temperature, after which time it was extracted with diethyl ether and ethyl acetate. The combined organic phase was washed with brine and the aqueous phase was extracted twice again with EtOAc then filtered through a frit with a thin layer of silica. The frit was rinsed with EtOAc then the filtrate phases were separated and the aqueous phase was extracted once again with EtOAc. The combined organic phase was dried over Na_2SO_4, filtered, and concentrated *in vacuo*. The crude concentrate was used in the next step without further purification.

The crude concentrate was dissolved in THF (0.10 M in the starting allylic alcohol) and TBAF (2.5 equiv) was added at room temperature. The resulting solution was stirred 1 h at room temperature, after which time water was added (1/4x the total THF volume) and the mixture was extracted with diethyl ether. The combined organic phase was dried over Na_2SO_4, filtered, and concentrated *in vacuo*. The crude concentrate was purified on silica by flash column chromatography with a gradient from 10–15% EtOAc in hexanes plus 5% DCM additive to afford the alcohol product as a mixture of isomers.* At this point, a yield for the reaction including all isomers was taken and ratios of isomeric products was evaluated via ^{1}H NMR. The isomeric mixture could then be further purified and enriched with the desired product *via* high pressure liquid chromatography with a gradient from 0–15% EtOAc in hexanes.

> Note: Even after careful chromatography (including HPLC) products from this procedure were typically contaminated with minor quantities of other isomers (e.g., regioisomers and (*E*)/(*Z*)-isomers)—see NMR spectra for each sample. As a result, we do not report optical rotation data for these samples and the corresponding carboxylic acids made from them.

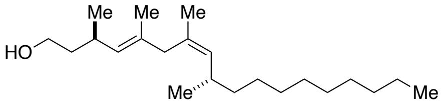

21

Data for compound 21: Yield: 61% (2 steps; rs = 3:1, *Z*:*E* (of major regioisomer) = 13:1; only one alkene stereoisomer visible *via* ^{1}H NMR for the minor regioisomeric coupling product); **TLC:** R_f = 0.35 (1:2:17; DCM:EtOAc:hexanes); **^{1}H NMR** (600 MHz, $CDCl_3$): δ 5.00 (d, J = 9.9 Hz, 1H), 4.95 (d, J = 9.9 Hz, 1H), 3.66–3.55 (m, 2H), 2.72 (d, J = 14.0 Hz, 1H), 2.63 (d, J = 14.7 Hz, 1H),

---

* The isolated isomeric mixture is assumed to be composed of both regio- and stereoisomers of the skipped diene product by analogy with our previously described coupling reactions of allylic alcohols and alkynes.[1,2,3,4,5]

2.57–2.49 (m, 1H), 2.37–2.29 (m, 1H), 1.65–1.59 (m, 1H), 1.58 (s, 3H), 1.56 (s, 3H), 1.50–1.43 (m, 1H), 1.32–1.13 (m, 17H), 0.96 (d, J = 6.5 Hz, 3H), 0.92–0.85 (m, 6H); **^{13}C NMR** (150 MHz, $CDCl_3$): δ 134.0, 132.5, 131.7, 131.1, 61.9, 42.2, 40.7, 38.0, 32.4, 32.1, 30.1, 29.9, 29.8, 29.6, 29.5, 27.7, 23.3, 22.8, 21.6, 21.5, 16.2, 14.3; **IR** (neat, cm^{-1}): 3349 (br), 2954 (s), 2916 (s), 2865 (s), 1451 (m), 1374 (w), 1220 (w), 1045 (m), 851 (w), 755 (s); **HRMS** (ESI-TOF) (*m/z*): $[M]^+$ calcd for $C_{22}H_{42}O$ 322.3236; found, 322.3241.

^{1}H NMR of isomeric mixture for compound 21 (major product peaks picked)

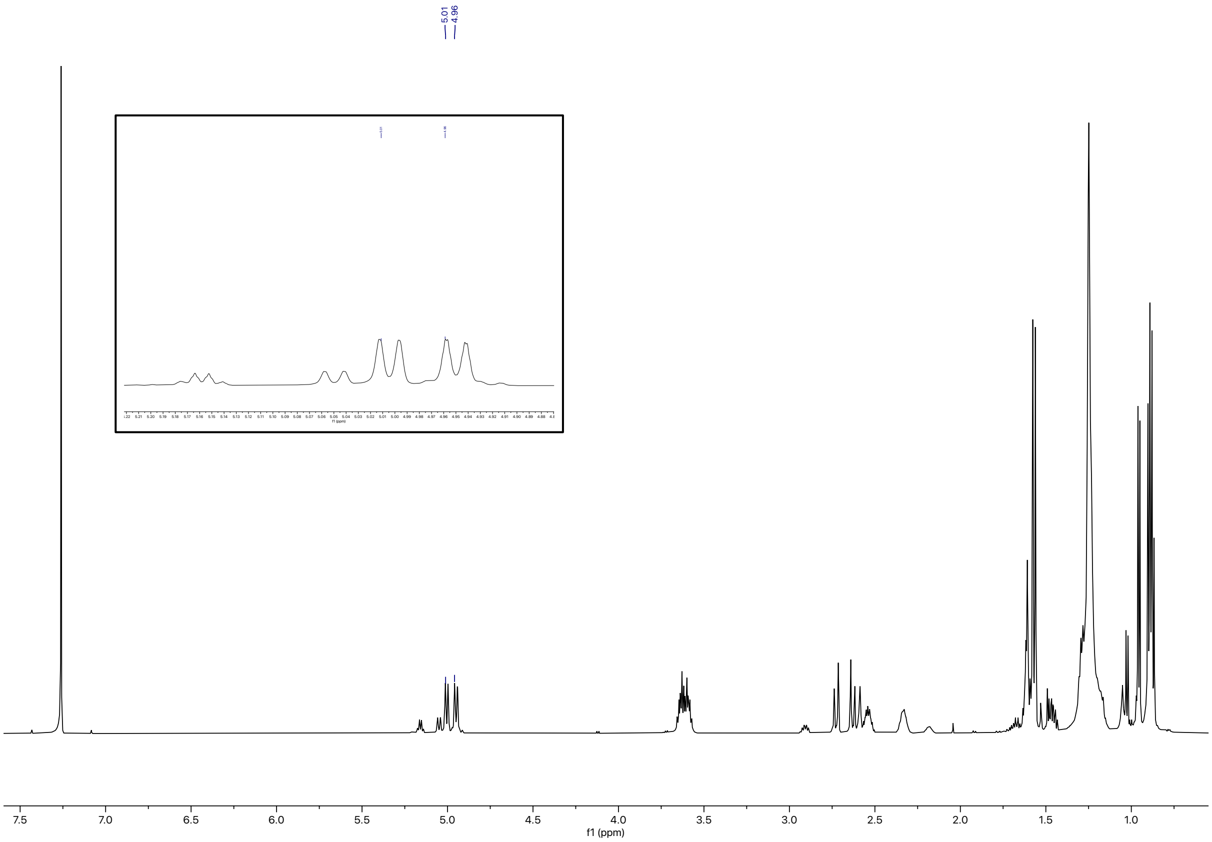

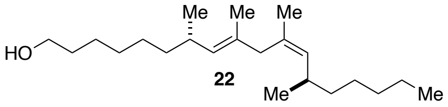

22

Data for compound 22: Yield: 51% (2 steps; rs = 4:1, *Z*:*E* (of major regioisomer) = 10:1; only one alkene stereoisomer visible *via* ^{1}H NMR for the minor regioisomeric coupling product); **TLC:** R_f = 0.31 (1:2:17; DCM:EtOAc:hexanes); **^{1}H NMR** (600 MHz, $CDCl_3$): δ 5.00 (d, J = 10.4 Hz, 1H), 4.90 (d, J = 10.4 Hz, 1H), 3.64 (t, J = 6.6 Hz, 2H), 2.70 (d, J = 13.7 Hz, 1H), 2.64 (d, J = 14.2 Hz, 1H), 2.41–2.28 (m, 2H), 1.58 (s, 3H), 1.57–1.53 (m, 2H), 1.52 (s, 3H), 1.37–1.12 (m, 17H), 0.93–0.84 (m, 9H); **^{13}C NMR** (150 MHz, $CDCl_3$): δ 133.8, 132.7, 131.5, 131.3, 63.3, 42.3, 38.0, 37.9, 33.0, 32.5, 32.3, 32.3, 29.8, 27.7, 27.1, 25.9, 23.3, 22.9, 21.5, 21.5, 16.2, 14.3; **IR** (neat, cm^{-1}): 3342 (br), 2949 (s), 2923 (s), 2851 (s), 1458 (m), 1367 (w), 1045 (w); **HRMS** (ESI-TOF) (*m/z*): $[M]^+$ calcd for $C_{22}H_{42}O$ 322.3236; found, 322.3243.

^{1}H NMR of isomeric mixture for compound 22 (major product peaks picked)

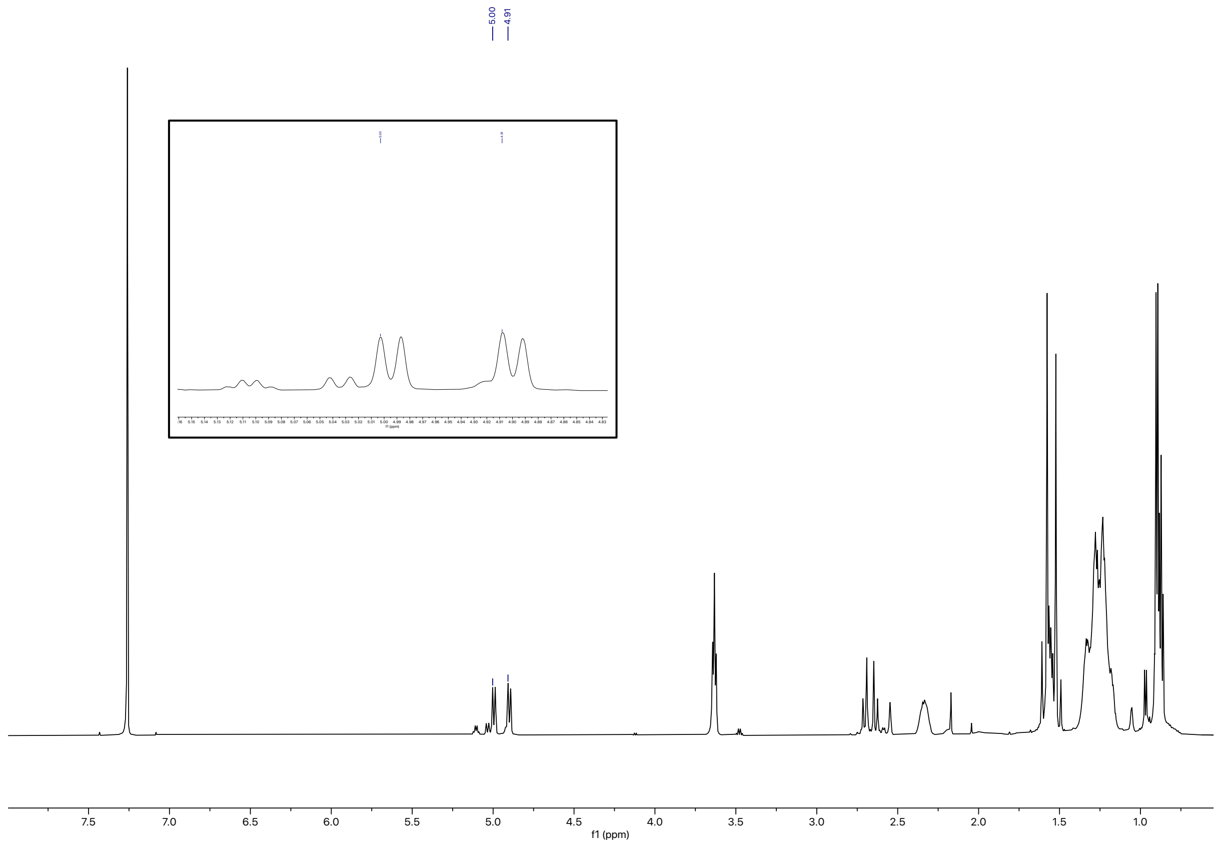

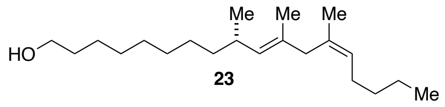

23

Data for compound 23: Yield: 64% (2 steps; rs = 4:1, *Z*:*E* (of major regioisomer) = 7:1; only one alkene stereoisomer visible *via* ^{1}H NMR for the minor regioisomeric coupling product); **TLC:** R_f = 0.30 (1:2:17; DCM:EtOAc:hexanes); **^{1}H NMR** (600 MHz, $CDCl_3$): δ 5.23 (t, *J* = 7.5 Hz, 1H), 4.90 (d, *J* = 9.7 Hz, 1H), 3.64 (t, *J* = 6.7 Hz, 2H), 2.67 (q, *J* = 16.3 Hz, 2H), 2.38–2.28 (m, 1H), 2.01 (q, *J* = 7.1 Hz, 2H), 1.59 (d, *J* = 1.4 Hz, 3H), 1.58–1.53 (m, 2H), 1.51 (d, *J* = 0.9 Hz, 3H), 1.38–1.12 (m, 17H), 0.95–0.85 (m, 6H); **^{13}C NMR** (150 MHz, $CDCl_3$): δ 133.2, 132.8, 131.2, 126.9, 63.3, 42.1, 38.0, 33.0, 32.5, 32.4, 29.9, 29.8, 29.6, 27.8, 27.7, 25.9, 23.3, 22.6, 21.5, 16.1, 14.2; **IR** (neat, cm^{-1}): 3393 (br), 2954 (s), 2916 (s), 2845 (s), 1451 (m), 1367 (w), 1058 (w); **HRMS** (ESI-TOF) (*m/z*): $[M]^+$ calcd for $C_{21}H_{40}O$ 308.3079; found, 308.3087.

^{1}H NMR of isomeric mixture for compound 23 (major product peaks picked)

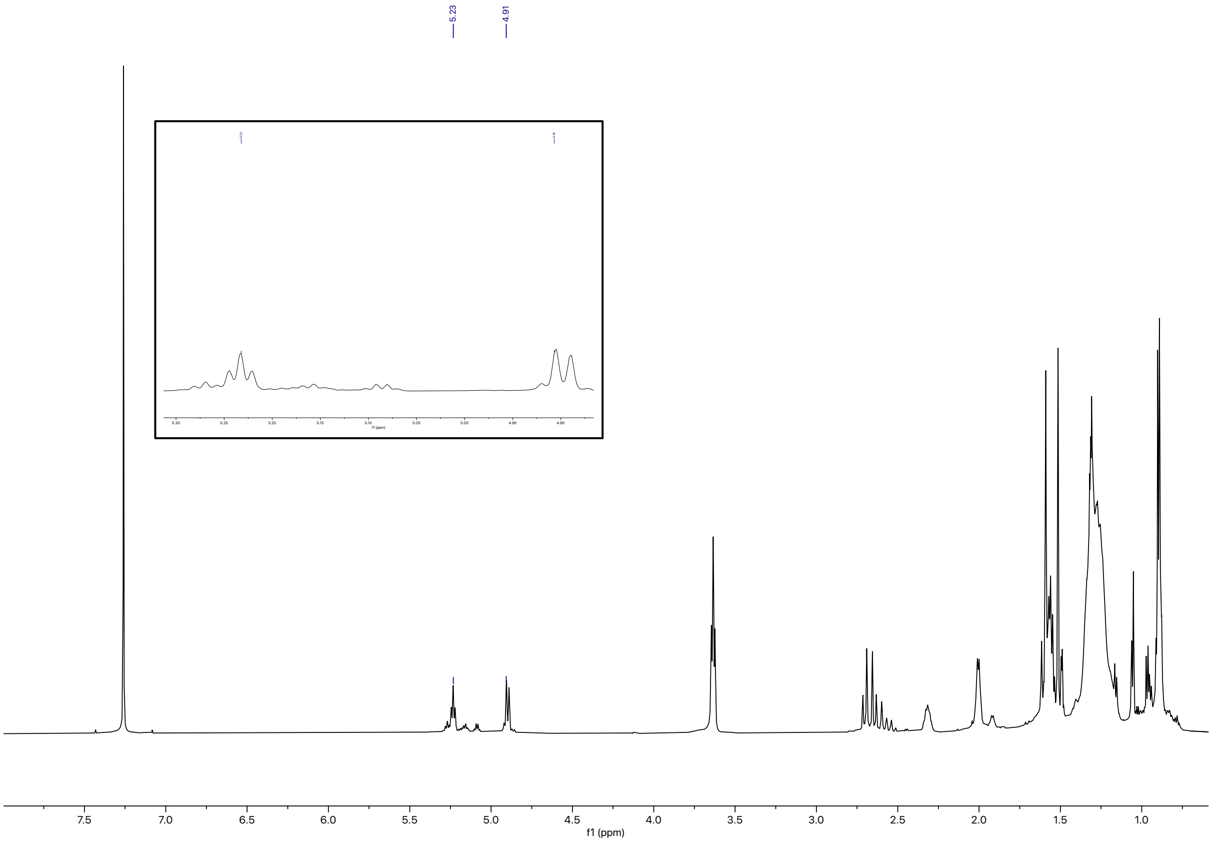

Characterization data for other mimetics prepared:

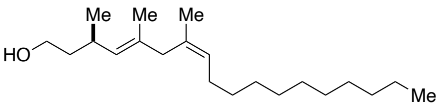

S19

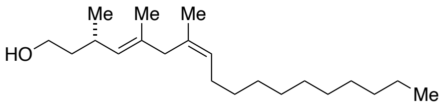

S20

Data for compound S19 and S20: TLC: R_f = 0.28 (1:2:17; DCM:EtOAc:hexanes); **^{1}H NMR** (600 MHz, $CDCl_3$): δ 5.24 (t, *J* = 6.8 Hz, 1H), 4.95 (d, *J* = 10.2 Hz, 1H), 3.66–3.56 (m, 2H), 2.68 (q, *J* = 11.0, 2H), 2.56–2.50 (m, 1H), 1.99 (q, *J* = 7.1 Hz, 2H), 1.63–1.61 (m, 1H), 1.59 (d, *J* = 1.1 Hz, 3H), 1.55 (d, *J* = 1.1 Hz, 3H), 1.47–1.43 (m, 1H), 1.35–1.21 (m, 17H), 0.96 (d, *J* = 6.7 Hz, 3H), 0.88 (t, *J* = 7.1 Hz, 3H); **^{13}C NMR** (150 MHz, $CDCl_3$): δ 132.8, 132.5, 131.7, 127.3, 61.9, 42.0, 40.7, 32.1, 30.2, 29.8, 29.8, 29.8, 29.6, 29.6, 29.5, 28.2, 23.3, 22.8, 21.6, 16.1, 14.3; **IR** (neat, cm^{-1}): 3323 (br), 2961 (s), 2916 (s), 2851 (s), 1445 (m), 1367 (w), 1058 (m), 755 (m); **HRMS** (ESI-TOF) (*m/z*): (**S19**) $[M]^+$ calcd for $C_{21}H_{40}O$ 308.3079; found, 308.3073, (**S20**) $[M+H]^+$ calcd for $C_{21}H_{41}O$ 309.3157; found, 309.3154.

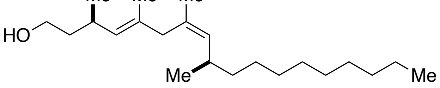

S21

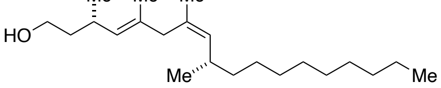

S23

Data for compound S21 and S23: TLC: R_f = 0.25 (1:2:17; DCM:EtOAc:hexanes); **^{1}H NMR** (500 MHz, $CDCl_3$): δ 5.00 (d, *J* = 9.8 Hz, 1H), 4.94 (d, *J* = 9.8 Hz, 1H), 3.67–3.55 (m, 2H), 2.73 (d, *J* = 14.6 Hz, 1H), 2.61 (d, *J* = 15.1 Hz, 1H), 2.57–2.49 (m, 1H), 2.37–2.27 (m, 1H), 1.65–1.59 (m, 1H), 1.57 (d, *J* = 1.2 Hz, 3H), 1.56 (d, *J* = 1.2 Hz, 3H), 1.50–1.42 (m, 1H), 1.32–1.13 (m, 17H), 0.95 (d, *J* = 6.6 Hz, 3H), 0.92–0.85 (m, 6H); **^{13}C NMR** (150 MHz, $CDCl_3$): δ 134.1, 132.5, 131.7, 131.1, 61.9, 42.2, 40.7, 38.1, 32.4, 32.1, 30.1, 29.9, 29.8, 29.6, 29.5, 27.7, 23.4, 22.8, 21.6, 21.6, 16.3, 14.3; **IR** (neat, cm^{-1}): 3342 (br), 2954 (s), 2916 (s), 2845 (s), 1439 (m), 1367 (w), 1052 (w), 748 (m); **HRMS** (ESI-TOF) (*m/z*): (**S21**) $[M]^+$ calcd for $C_{22}H_{42}O$ 322.3236; found, 322.3240, (**S23**) $[M+H]^+$ calcd for $C_{22}H_{43}O$ 323.3314; found, 323.3306.

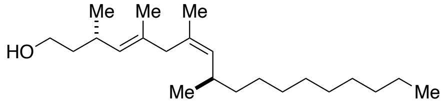

S22

Data for compound S22: See enantiomer **21** above for **TLC**, **^{1}H NMR**, **^{13}C NMR**, and **IR** data; **HRMS** (ESI-TOF) (*m/z*): $[M]^+$ calcd for $C_{22}H_{42}O$ 322.3236; found, 322.3227.

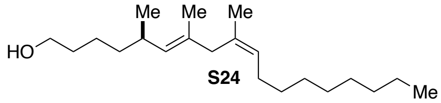

S24

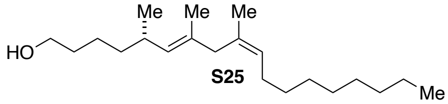

S25

Data for compound S24 and S25: TLC: R_f = 0.25 (1:2:17; DCM:EtOAc:hexanes); **^{1}H NMR** (600 MHz, $CDCl_3$): δ 5.23 (t, *J* = 6.7 Hz, 1H), 4.90 (d, *J* = 7.9 Hz, 1H), 3.66–3.58 (m, 2H), 2.67 (q, *J* = 12.2 Hz, 2H), 2.38–2.30 (m, 1H), 1.99 (q, *J* = 6.8 Hz, 2H), 1.59 (d, *J* = 1.0 Hz, 3H), 1.56–1.53 (m, 2H), 1.51 (d, *J* = 1.1 Hz, 3H), 1.36–1.17 (m, 17H), 0.91 (d, *J* = 6.6 Hz, 3H), 0.88 (t, *J* = 7.1 Hz, 3H); **^{13}C NMR** (150 MHz, $CDCl_3$): δ 133.1, 132.5, 131.6, 127.0, 63.3, 42.1, 37.7, 33.1, 32.5, 32.1, 30.2, 29.7, 29.6, 29.5, 28.2, 23.9, 23.3, 22.8, 21.5, 16.1, 14.3; **IR** (neat, cm^{-1}): 3342 (br), 2949 (s), 2916 (s), 2851 (s), 1439 (m), 1367 (w), 1220 (w), 1045 (m), 748 (s); **HRMS** (ESI-TOF) (*m/z*): $[M]^+$ calcd for $C_{21}H_{40}O$ 308.3079; found, 308.3078 (**S24**), 308.3073 (**S25**).

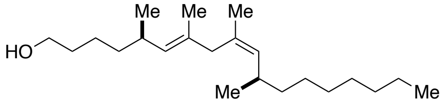

S26

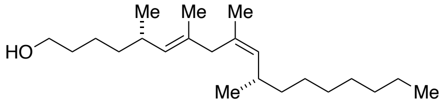

S29

Data for compound S26 and S29: TLC: R_f = 0.33 (1:2:17; DCM:EtOAc:hexanes); **^{1}H NMR** (600 MHz, $CDCl_3$): δ 5.00 (d, *J* = 9.5 Hz, 1H), 4.90 (d, *J* = 9.5 Hz, 1H), 3.62 (t, *J* = 6.5 Hz, 2H), 2.74 (d, *J* = 14.9 Hz, 1H), 2.60 (d, *J* = 13.2 Hz, 1H), 2.34 (br, 2H), 1.58 (s, 3H), 1.57–1.54 (m, 2H), 1.53 (s, 3H), 1.36–1.11 (m, 17H), 0.94–0.85 (m, 9H); **^{13}C NMR** (150 MHz, $CDCl_3$): δ 133.8, 132.4, 131.6, 131.4, 63.3, 42.3, 38.0, 37.7, 33.1, 32.5, 32.4, 32.1, 30.0, 29.5, 27.7, 23.9, 23.3, 22.8, 21.6, 21.5, 16.3, 14.3; **IR** (neat, cm^{-1}): 3330 (br), 2942 (s), 2910 (s), 2845 (s), 1445 (m), 1374 (w), 1045 (w); **HRMS** (ESI-TOF) (*m/z*): $[M]^+$ calcd for $C_{22}H_{42}O$ 322.3236; found, 322.3241 (**S26**), 322.3237 (**S29**).

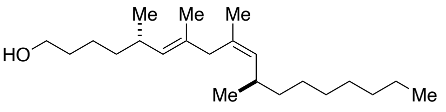

S27

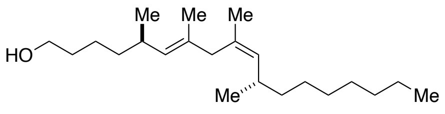

S28

Data for compound S27 and S28: TLC: R_f = 0.32 (1:2:17; DCM:EtOAc:hexanes); **^{1}H NMR** (600 MHz, $CDCl_3$): δ 5.00 (d, *J* = 9.7 Hz, 1H), 4.91 (d, *J* = 9.7 Hz, 1H), 3.62 (t, *J* = 6.4 Hz, 2H), 2.71 (d, *J* = 14.3 Hz, 1H), 2.63 (d, *J* = 14.5 Hz, 1H), 2.35 (br, 2H), 1.58 (s, 3H), 1.57–1.54 (m, 2H), 1.53 (s, 3H), 1.40–1.13 (m, 17H), 0.93–0.86 (m, 9H); **^{13}C NMR** (150 MHz, $CDCl_3$): δ 133.8, 132.5, 131.6, 131.4, 63.3, 42.3, 38.0, 37.7, 33.2, 32.5, 32.3, 32.1, 30.1, 29.5, 27.7, 23.9, 23.3, 22.8, 21.5, 21.5, 16.2, 14.3; **IR** (neat, cm^{-1}): 3323 (br), 2954 (s), 2916 (s), 2858 (s), 1451 (m), 1374 (w), 1038 (w); **HRMS** (ESI-TOF) (*m/z*): $[M]^+$ calcd for $C_{22}H_{42}O$ 322.3236; found, 322.3227 (**S27**), 322.3246 (**S28**).

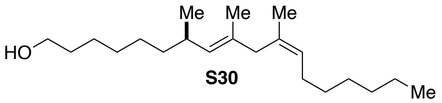

S30

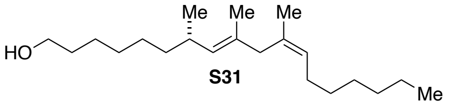

S31

Data for compound S30 and S31: TLC: R_f = 0.25 (1:2:17; DCM:EtOAc:hexanes); **^{1}H NMR** (600 MHz, $CDCl_3$): δ 5.23 (t, *J* = 6.9 Hz, 1H), 4.90 (d, *J* = 9.0 Hz, 1H), 3.63 (t, *J* = 6.3 Hz, 2H), 2.67 (q, *J* = 14.8 Hz, 2H), 2.37–2.28 (m, 1H), 2.00 (q, *J* = 7.2 Hz, 2H), 1.59 (d, *J* = 1.0 Hz, 3H), 1.58–1.53 (m, 2H), 1.51 (d, *J* = 1.1 Hz, 3H), 1.38–1.13 (m, 17H), 0.92–0.85 (m, 6H); **^{13}C NMR** (150 MHz, $CDCl_3$): δ 133.2, 132.7, 131.3, 127.0, 63.3, 42.1, 37.9, 33.0, 32.5, 32.0, 30.2, 29.8, 29.3, 28.2, 27.7, 25.9, 23.3, 22.8, 21.5, 16.1, 14.3; **IR** (neat, cm^{-1}): 3368 (br), 2954 (s), 2916 (s), 2851 (s), 1445 (m), 1374 (w), 1058 (w); **HRMS** (ESI-TOF) (*m/z*): $[M]^+$ calcd for $C_{21}H_{40}O$ 308.3079; found, 308.3078 (**S30**), 308.3083 (**S31**).

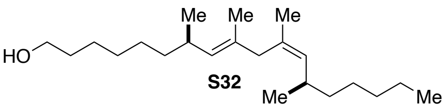

S32

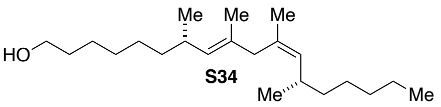

S34

Data for compound S32 and S34: TLC: R_f = 0.24 (1:2:17; DCM:EtOAc:hexanes); **^{1}H NMR** (600 MHz, $CDCl_3$): δ 4.99 (d, *J* = 10.3 Hz, 1H), 4.90 (d, *J* = 9.9 Hz, 1H), 3.63 (t, *J* = 6.6 Hz, 2H), 2.74 (d, *J* = 14.1 Hz, 1H), 2.60 (d, *J* = 14.7 Hz, 1H), 2.40–2.28 (m, 2H), 1.58 (s, 3H), 1.57–1.54 (m, 2H), 1.52 (s, 3H), 1.40–1.13 (m, 17H), 0.93–0.86 (m, 9H); **^{13}C NMR** (150 MHz, $CDCl_3$): δ 133.8, 132.7, 131.5, 131.3, 63.3, 42.3, 38.0, 37.9, 33.0, 32.5, 32.3, 32.3, 29.8, 27.7, 27.4, 25.9, 23.3, 22.8, 21.6, 21.5, 16.2, 14.3; **IR** (neat, cm^{-1}): 3422 (br), 2954 (s), 2916 (s), 2851 (s), 1446 (m), 1369 (w), 1055 (w); **HRMS** (ESI-TOF) (*m/z*): $[M]^+$ calcd for $C_{22}H_{42}O$ 322.3236; found, 322.3230 (**S32**), 322.3232 (**S34**).

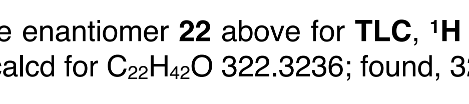

S33

Data for compound S33: See enantiomer **22** above for **TLC**, **^{1}H NMR**, **^{13}C NMR**, and **IR** data; **HRMS** (ESI-TOF) (*m/z*): $[M]^+$ calcd for $C_{22}H_{42}O$ 322.3236; found, 322.3234.

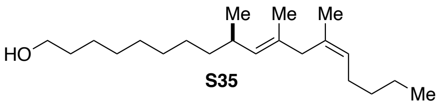

Data for compound S35: See enantiomer **23** above for **TLC**, **^{1}H NMR**, **^{13}C NMR**, and **IR** data; **HRMS** (ESI-TOF) (*m/z*): $[M]^+$ calcd for $C_{21}H_{40}O$ 308.3079; found, 308.3084.

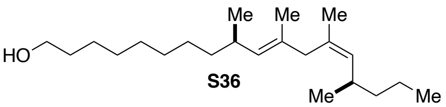

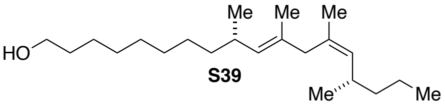

Data for compound S36 and S39: TLC: R_f = 0.24 (1:2:17; DCM:EtOAc:hexanes); **^{1}H NMR** (600 MHz, $CDCl_3$): δ 4.99 (d, *J* = 9.6 Hz, 1H), 4.90 (d, *J* = 9.6 Hz, 1H), 3.63 (t, *J* = 6.7 Hz, 2H), 2.74 (d, *J* = 14.3 Hz, 1H), 2.60 (d, *J* = 14.3 Hz, 1H), 2.41–2.28 (m, 2H), 1.58 (s, 3H), 1.57–1.54 (m, 2H), 1.52 (s, 3H), 1.38–1.12 (m, 17H), 0.93–0.82 (m, 9H); **^{13}C NMR** (150 MHz, $CDCl_3$): δ 133.7, 132.7, 131.5, 131.2, 63.3, 42.3, 40.3, 38.0, 33.0, 32.5, 32.1, 29.9, 29.8, 29.6, 27.7, 25.9, 23.4, 21.6, 21.5, 20.8, 16.2, 14.5; **IR** (neat, cm^{-1}): 3403 (br), 2960 (s), 2922 (s), 2851 (s), 1440 (m), 1369 (w), 1041 (w); **HRMS** (ESI-TOF) (*m/z*): $[M]^+$ calcd for $C_{22}H_{42}O$ 322.3236; found, 322.3237 (**S36**), 322.3238 (**S39**).

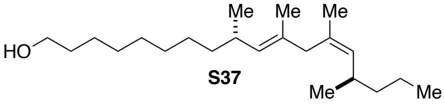

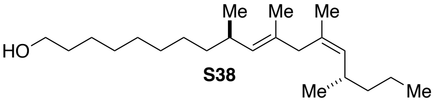

Data for compound S37 and S38: TLC: R_f = 0.28 (1:2:17; DCM:EtOAc:hexanes); **^{1}H NMR** (600 MHz, $CDCl_3$): δ 5.00 (d, *J* = 9.3 Hz, 1H), 4.90 (d, *J* = 10.4 Hz, 1H), 3.65 (t, *J* = 6.7 Hz, 2H), 2.70 (d, *J* = 15.1 Hz, 1H), 2.64 (d, *J* = 14.3 Hz, 1H), 2.41–2.28 (m, 2H), 1.58 (s, 3H), 1.57–1.54 (m, 2H), 1.52 (s, 3H), 1.39–1.12 (m, 17H), 0.93–0.83 (m, 9H); **^{13}C NMR** (150 MHz, $CDCl_3$): δ 133.7, 132.8, 131.5, 131.2, 63.3, 42.2, 40.3, 38.0, 33.0, 32.5, 32.1, 29.9, 29.8, 29.6, 27.7, 25.9, 23.3, 21.5, 21.5, 20.8, 16.2, 14.5; **IR** (neat, cm^{-1}): 3413 (br), 2954 (s), 2916 (s), 2851 (s), 1439 (m), 1362 (w), 1045 (w); **HRMS** (ESI-TOF) (*m/z*): $[M]^+$ calcd for $C_{22}H_{42}O$ 322.3236; found, 322.3230 (**S37**), 322.3242 (**S38**).

G. General Procedure for the Synthesis of Coupled Chiral Acids

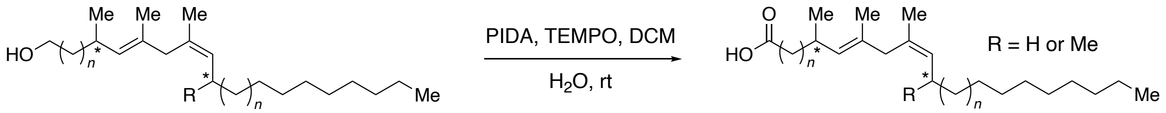

To a stirring solution of the alcohol (1.0 equiv) in DCM/H_2O (0.02 M in the alcohol; 1:1 DCM:H_2O) was added sequentially PIDA (2.2 equiv) then TEMPO (0.2 equiv) at room temperature. The resulting mixture was stirred overnight (~20 h) then quenched by the addition of a saturated aqueous solution of $Na_2S_2O_3$ (1/2 total DCM/H_2O volume). The aqueous phase was extracted with DCM and the combined organic phase was dried over Na_2SO_4, filtered, and concentrated *in vacuo*. The crude material was then run though a short silica pipet column, eluting first with DCM to remove impurities, then EtOAc to isolate the acid as a pale oil.

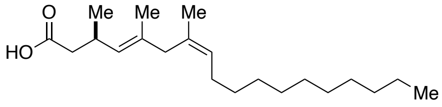
1-A

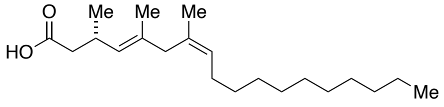
1-B

Data for compound 1-A and 1-B: Yield: 82% (**1-A**), 63% (**1-B**); **TLC:** R_f = 0.29 (3:2:15; EtOAc:DCM:hexanes); **^{1}H NMR** (600 MHz, $CDCl_3$): δ 11.02–9.98 (br, 1H), 5.24 (t, *J* = 6.9 Hz, 1H), 4.97 (d, *J* = 9.4 Hz, 1H), 2.98–2.88 (m, 1H), 2.67 (q, *J* = 13.1 Hz, 2H), 2.29 (t, *J* = 8.2 Hz, 2H), 1.98 (q, *J* = 7.2 Hz, 2H), 1.56 (s, 6H), 1.38–1.20 (m, 16H), 1.01 (d, *J* = 6.8 Hz, 3H), 0.88 (t, *J* = 7.1 Hz, 3H); **^{13}C NMR** (150 MHz, $CDCl_3$): δ 177.9, 133.3, 132.7, 129.8, 127.3, 42.0, 41.9, 32.1, 30.2, 29.9, 29.8, 29.8, 29.8, 29.6, 29.5, 28.1, 23.1, 22.8, 21.1, 16.0, 14.3; **IR** (neat, cm^{-1}): 3445 (br), 2954 (w), 2915 (s), 2851 (w), 1703 (m), 1652 (s), 1465 (w); **HRMS** (ESI-TOF) (*m/z*): $[M\text{-}H]^+$ calcd for $C_{21}H_{37}O_2$ 321.2794; found, 321.2791(**1-A**), 321.2789 (**1-B**).

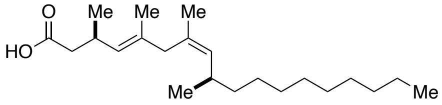
1-C

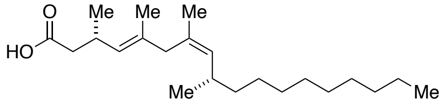
1-F

Data for compound 1-C and 1-F: Yield: 63% (**1-C**), 56% (**1-F**); **TLC:** R_f = 0.34 (3:2:15; EtOAc:DCM:hexanes); **^{1}H NMR** (600 MHz, $CDCl_3$): δ 11.06–10.10 (br, 1H), 4.98 (dd, *J* = 14.8, 9.5 Hz, 2H), 2.97–2.89 (m, 1H), 2.73 (d, *J* = 14.4 Hz, 1H), 2.59 (d, *J* = 14.1 Hz, 1H), 2.37–2.24 (m, 3H), 1.57 (s, 3H), 1.55 (s, 3H), 1.38–1.12 (m, 16 H), 1.02 (d, *J* = 6.7 Hz, 3H), 0.93–0.86 (m, 6H); **^{13}C NMR** (150 MHz, $CDCl_3$): δ 177.9, 134.1, 133.4, 131.0, 129.7, 42.1, 42.0, 38.0, 32.4, 32.1, 30.1, 29.9, 29.9, 29.8, 29.5, 27.7, 23.2, 22.8, 21.6, 21.1, 16.2, 14.3; **IR** (neat, cm^{-1}): 3410 (br), 2954 (w), 2916 (s), 2858 (w), 1709 (s), 1632 (s), 1440 (w); **HRMS** (ESI-TOF) (*m/z*): $[M\text{-}H]^+$ calcd for $C_{22}H_{39}O_2$ 335.2950; found, 335.2943 (**1-C**), 335.2949 (**1-F**).

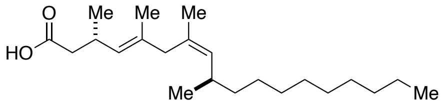
1-D

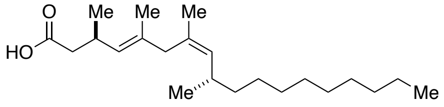
1-E

Data for compound 1-D and 1-E: Yield: 56% (**1-D**), 55% (**1-E**); **TLC:** R_f = 0.32 (3:2:15; EtOAc:DCM:hexanes); **^{1}H NMR** (600 MHz, $CDCl_3$): δ 11.72–10.04 (br, 1H), 4.99 (dd, *J* = 14.9, 9.5 Hz, 2H), 2.97–2.89 (m, 1H), 2.72 (d, *J* = 14.2 Hz, 1H), 2.62 (d, *J* = 14.0 Hz, 1H), 2.37–2.25 (m, 3H), 1.57 (s, 3H), 1.55 (s, 3H), 1.37–1.12 (m, 16 H), 1.02 (d, *J* = 6.6 Hz, 3H), 0.93–0.85 (m, 6H); **^{13}C NMR** (150 MHz, $CDCl_3$): δ 177.9, 134.1, 133.4, 131.0, 129.8, 42.1, 41.9, 38.0, 32.4, 32.1, 30.1, 29.9, 29.8, 29.8, 29.5, 27.7, 23.2, 22.8, 21.5, 21.1, 16.1, 14.3; **IR** (neat, cm^{-1}): 3433 (br), 2954 (w), 2916 (s), 2851 (w), 1710 (s), 1645 (s), 1439 (w); **HRMS** (ESI-TOF) (*m/z*): $[M\text{-}H]^+$ calcd for $C_{22}H_{39}O_2$ 335.2950; found, 335.2943 (**1-D**), 335.2941 (**1-E**).

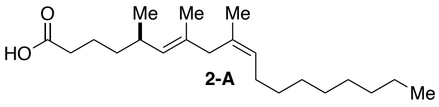
2-A

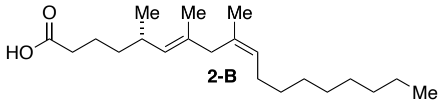
2-B

Data for compound 2-A and 2-B: Yield: 82% (**2-A**), 81% (**2-B**); **TLC:** R_f = 0.32 (3:2:15; EtOAc:DCM:hexanes); **^{1}H NMR** (600 MHz, $CDCl_3$): δ 12.00–10.20 (br, 1H), 5.24 (t, *J* = 7.3 Hz, 1H), 4.90 (d, *J* = 8.5 Hz, 1H), 2.67 (q, *J* = 10.5 Hz, 2H), 2.40–2.28 (m, 3H), 1.99 (q, *J* = 6.9 Hz, 2H), 1.59 (s, 3H), 1.52 (s, 3H), 1.39–1.19 (m, 16H), 0.93 (d, *J* = 6.4 Hz, 3H), 0.88 (t, *J* = 7.0 Hz, 3H); **^{13}C NMR** (150 MHz, $CDCl_3$): δ 179.3, 133.0, 132.6, 132.0, 127.1, 42.0, 37.2, 34.2, 32.3, 32.1, 30.2, 29.7, 29.6, 29.5, 28.2, 23.3, 23.0, 22.8, 21.4, 16.1, 14.3; **IR** (neat, cm^{-1}): 3410 (br), 2954 (w), 2916 (s), 2858 (w), 1709 (s), 1638 (s), 1459 (w); **HRMS** (ESI-TOF) (*m/z*): $[M-H]^+$ calcd for $C_{21}H_{37}O_2$ 321.2794; found, 321.2787.

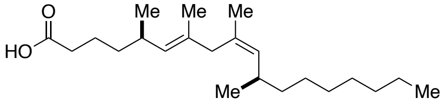

2-C

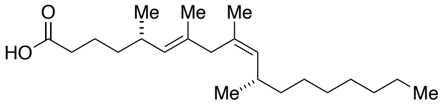

2-F

Data for compound 2-C and 2-F: Yield: 91% (**2-C**), quantitative (**2-F**); **TLC:** R_f = 0.33 (3:2:15; EtOAc:DCM:hexanes); **^{1}H NMR** (600 MHz, $CDCl_3$): δ 11.61–11.50 (br, 1H), 5.00 (d, *J* = 9.6 Hz, 1H), 4.90 (d, *J* = 9.5 Hz, 1H), 2.73 (d, *J* = 14.2 Hz, 1H), 2.61 (d, *J* = 14.2 Hz, 1H), 2.41–2.29 (m, 4H), 1.58 (s, 3H), 1.54 (s, 3H), 1.39–1.13 (m, 16H), 0.95–0.86 (m, 9H); **^{13}C NMR** (150 MHz, $CDCl_3$): δ 179.3, 133.8, 131.9, 131.8, 131.3, 42.2, 38.0, 37.2, 34.3, 32.3, 32.3, 32.0, 30.0, 29.5, 27.7, 23.3, 22.9, 22.8, 21.5, 21.3, 16.2, 14.2; **IR** (neat, cm^{-1}): 3419 (br), 2954 (m), 2916 (s), 2865 (w), 1710 (s), 1645 (s), 1451 (w); **HRMS** (ESI-TOF) (*m/z*): $[M-H]^+$ calcd for $C_{22}H_{39}O_2$ 335.2950; found, 335.2939 (**2-C**), 335.2950 (**2-F**).

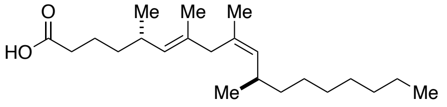

2-D

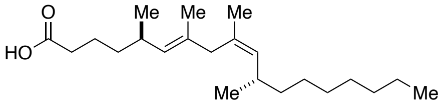

2-E

Data for compound 2-D and 2-E: Yield: 80% (**2-D**), 77% (**2-E**); **TLC:** R_f = 0.34 (3:2:15; EtOAc:DCM:hexanes); **^{1}H NMR** (600 MHz, $CDCl_3$): δ 11.21–10.05 (br, 1H), 5.00 (d, *J* = 9.6 Hz, 1H), 4.91 (d, *J* = 9.4 Hz, 1H), 2.73 (d, *J* = 13.0 Hz, 1H), 2.62 (d, *J* = 13.4 Hz, 1H), 2.46–2.27 (m, 4H), 1.58 (s, 3H), 1.53 (s, 3H), 1.43–1.12 (m, 16H), 0.96–0.84 (m, 9H); **^{13}C NMR** (150 MHz, $CDCl_3$): δ 179.0, 133.8, 132.0, 131.9, 131.3, 42.2, 38.0, 37.2, 34.2, 32.3, 32.3, 32.1, 30.0, 29.5, 27.7, 23.3, 23.0, 22.8, 21.5, 21.4, 16.2, 14.2; **IR** (neat, cm^{-1}): 3419 (br), 2949 (m), 2916 (s), 2851 (w), 1703 (m), 1632 (s), 1439 (w); **HRMS** (ESI-TOF) (*m/z*): $[M-H]^+$ calcd for $C_{22}H_{39}O_2$ 335.2950; found, 335.2943 (**2-D**), 335.2945 (**2-E**).

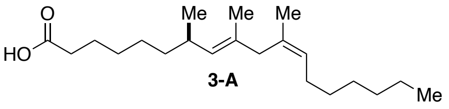

3-A

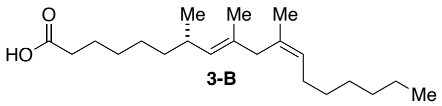

3-B

Data for compound 3-A and 3-B: Yield: 71% (**3-A**), 73% (**3-B**); **TLC:** R_f = 0.22 (3:2:15; EtOAc:DCM:hexanes); **^{1}H NMR** (600 MHz, $CDCl_3$): δ 11.20–10.21 (br, 1H), 5.24 (t, *J* = 6.8 Hz, 1H), 4.89 (d, *J* = 8.7 Hz, 1H), 2.67 (q, *J* = 14.0, 2H), 2.33 (t, *J* = 7.2 Hz, 3H), 2.00 (q, *J* = 7.7 Hz, 2H), 1.59 (s, 3H), 1.51 (s, 3H), 1.39–1.12 (m, 16H), 0.93–0.82 (m, 6H); **^{13}C NMR** (150 MHz, $CDCl_3$): δ 178.9, 133.1, 132.6, 131.4, 127.0, 42.0, 37.7, 34.0, 32.5, 32.0, 30.2, 29.4, 29.3, 28.1, 27.3, 24.9, 23.3, 22.8, 21.5, 16.1, 14.2; **IR** (neat, cm^{-1}): 3436 (br), 2960 (m), 2922 (s), 2851 (w), 1703 (m), 1638 (s), 1440 (w); **HRMS** (ESI-TOF) (*m/z*): $[M-H]^+$ calcd for $C_{21}H_{37}O_2$ 321.2794; found, 321.2790 (**3-A**), 321.2783 (**3-B**).

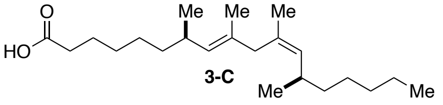
3-C

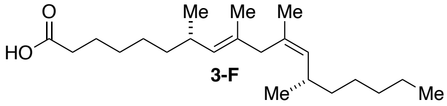
3-F

Data for compound 3-C and 3-F: Yield: 66% (**3-C**), 77% (**3-F**); **TLC:** R_f = 0.22 (3:2:15; EtOAc:DCM:hexanes); **^{1}H NMR** (600 MHz, $CDCl_3$): δ 10.36–9.84 (br, 1H), 4.99 (d, *J* = 10.14 Hz, 1H), 4.89 (d, *J* = 9.8 Hz, 1H), 2.74 (d, *J* = 13.7 Hz, 1H), 2.60 (d, *J* = 15.0 Hz, 1H), 2.38–2.29 (m, 4H), 1.58 (s, 3H), 1.52 (s, 3H), 1.39–1.12 (m, 16H), 0.93–0.85 (m, 9H); **^{13}C NMR** (150 MHz, $CDCl_3$): δ 178.3, 133.8, 132.5, 131.5, 131.4, 42.2, 38.0, 37.7, 33.9, 32.5, 32.3, 32.3, 29.4, 27.4, 27.3, 24.9, 23.3, 22.8, 21.6, 21.5, 16.2, 14.3; **IR** (neat, cm^{-1}): 3429 (br), 2967 (m), 2916 (m), 2858 (w), 1703 (s), 1638 (s), 1452 (w); **HRMS** (ESI-TOF) (*m/z*): $[M-H]^+$ calcd for $C_{22}H_{39}O_2$ 335.2950; found, 335.2941 (**3-C**), 335.2940 (**3-F**).

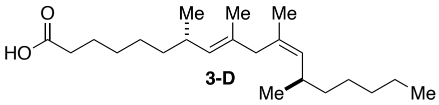
3-D

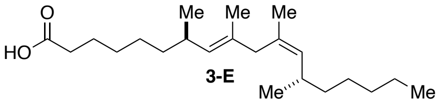
3-E

Data for compound 3-D and 3-E: Yield: 83% (**3-D**), 73% (**3-E**); **TLC:** R_f = 0.24 (3:2:15; EtOAc:DCM:hexanes); **^{1}H NMR** (600 MHz, $CDCl_3$): δ 11.46–10.10 (br, 1H), 5.00 (d, *J* = 9.5 Hz, 1H), 4.89 (d, *J* = 9.4 Hz, 1H), 2.71 (d, *J* = 14.6 Hz, 1H), 2.63 (d, *J* = 14.0 Hz, 1H), 2.39–2.28 (m, 4H), 1.57 (s, 3H), 1.52 (s, 3H), 1.42–1.12 (m, 16H), 0.94–0.83 (m, 9H); **^{13}C NMR** (150 MHz, $CDCl_3$): δ 179.1, 133.8, 132.5, 131.4, 131.4, 42.2, 38.0, 37.7, 34.0, 32.5, 32.3, 32.3, 29.4, 27.3, 27.3, 24.9, 23.3, 22.8, 21.5, 21.5, 16.2, 14.2; **IR** (neat, cm^{-1}): 3433 (br), 2954 (m), 2916 (s), 2851 (w), 1710 (m), 1645 (s), 1452 (w); **HRMS** (ESI-TOF) (*m/z*): $[M-H]^+$ calcd for $C_{22}H_{39}O_2$ 335.2950; found, 335.2946 (**3-D**), 335.2944 (**3-E**).

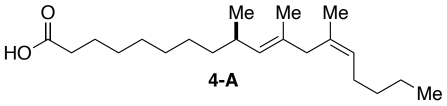
4-A

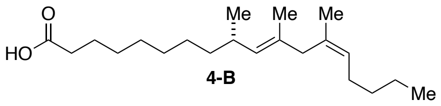
4-B

Data for compound 4-A and 4-B: Yield: 74% (**4-A**), 64% (**4-B**); **TLC:** R_f = 0.23 (3:2:15; EtOAc:DCM:hexanes); **^{1}H NMR** (600 MHz, $CDCl_3$): δ 11.43–10.07 (br, 1H), 5.23 (t, *J* = 7.2 Hz, 1H), 4.90 (d, *J* = 9.2 Hz, 1H), 2.67 (q, *J* = 15.7, 2H), 2.34 (t, *J* = 7.9 Hz, 3H), 2.01 (q, *J* = 7.1 Hz, 2H), 1.59 (s, 3H), 1.52 (s, 3H), 1.39–1.12 (m, 16H), 0.95–0.85 (m, 6H); **^{13}C NMR** (150 MHz, $CDCl_3$): δ 179.3, 133.2, 132.8, 131.3, 126.9, 42.1, 37.9, 34.1, 32.5, 32.4, 29.7, 29.4, 29.2, 27.8, 27.6, 24.8, 23.3, 22.6, 21.5, 16.1, 14.2; **IR** (neat, cm^{-1}): 3426 (br), 2961 (m), 2923 (s), 2845 (w), 1710 (m), 1639 (s), 1445 (w); **HRMS** (ESI-TOF) (*m/z*): $[M-H]^+$ calcd for $C_{21}H_{37}O_2$ 321.2794; found, 321.2791 (**4-A**), 321.2784 (**4-B**).

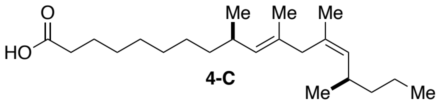
4-C

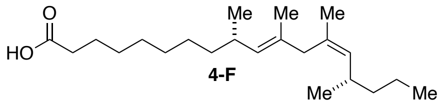
4-F

Data for compound 4-C and 4-F: Yield: 69% (**4-C**), 78% (**4-F**); **TLC:** R_f = 0.21 (3:2:15; EtOAc:DCM:hexanes); ^{1}**H NMR** (600 MHz, $CDCl_3$): δ 12.03–10.17 (br, 1H), 5.00 (d, *J* = 9.9 Hz, 1H), 4.90 (d, *J* = 9.1 Hz, 1H), 2.74 (d, *J* = 14.1 Hz, 1H), 2.60 (d, *J* = 14.7 Hz, 1H), 2.40–2.28 (m, 4H), 1.58 (s, 3H), 1.53 (s, 3H), 1.39–1.12 (m, 16H), 0.95–0.82 (m, 9H); ^{13}**C NMR** (150 MHz, $CDCl_3$): δ 179.6, 133.7, 132.7, 131.5, 131.3, 42.2, 40.3, 37.9, 34.1, 32.5, 32.1, 29.7, 29.4, 29.2, 27.6, 24.8, 23.3, 21.5, 21.5, 20.8, 16.2, 14.5; **IR** (neat, cm^{-1}): 3400 (br), 2954 (m), 2923 (s), 2851 (w), 1703 (m), 1645 (s), 1451 (w); **HRMS** (ESI-TOF) (*m/z*): $[M-H]^+$ calcd for $C_{22}H_{39}O_2$ 335.2950; found, 335.2945 (**4-C**), 335.2941 (**4-F**).

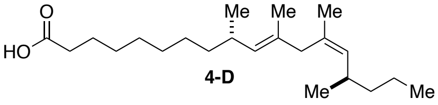

4-D

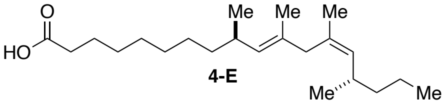

4-E

Data for compound 4-D and 4-E: Yield: 72% (**4-D**), 77% (**4-E**); **TLC:** R_f = 0.21 (3:2:15; EtOAc:DCM:hexanes); ^{1}**H NMR** (600 MHz, $CDCl_3$): δ 11.19–10.05 (br, 1H), 5.00 (d, *J* = 9.4 Hz, 1H), 4.90 (d, *J* = 10.2 Hz, 1H), 2.71 (d, *J* = 14.3 Hz, 1H), 2.64 (d, *J* = 13.8 Hz, 1H), 2.40–2.28 (m, 4H), 1.58 (s, 3H), 1.52 (s, 3H), 1.37–1.12 (m, 16H), 0.93–0.83 (m, 9H); ^{13}**C NMR** (150 MHz, $CDCl_3$): δ 178.9, 133.7, 132.7, 131.5, 131.3, 42.2, 40.3, 37.9, 34.0, 32.5, 32.1, 29.8, 29.4, 29.2, 27.6, 24.9, 23.3, 21.5, 21.5, 20.8, 16.2, 14.5; **IR** (neat, cm^{-1}): 3441 (br), 2954 (m), 2928 (s), 2858 (w), 1709 (m), 1645 (s), 1440 (w); **HRMS** (ESI-TOF) (*m/z*): $[M-H]^+$ calcd for $C_{22}H_{39}O_2$ 335.2950; found, 335.2945 (**4-D**), 335.2943 (**4-E**).

References

[1] Kolundzic, F.; Micalizio, G. C. Synthesis of Substituted 1,4-Dienes by Direct Alkylation of Allylic Alcohols. *J. Am. Chem. Soc.* **2007**, *129*, 15112.

[2] Diez, P. S.; Micalizio, G. C. Chemoselective Reductive Cross-Coupling of 1,5-Diene-3-ols with Alkynes: A Facile Entry to Stereodefined Skipped Trienes. *J. Am. Chem. Soc.* **2010**, *132*, 9576–9578.

[3] Jeso, V.; Micalizio, G. C.; Total Synthesis of Lehualide B by Allylic Alcohol–Alkyne Reductive Cross-Coupling. *J. Am. Chem. Soc.* **2010**, *132*, 11422–11424.

[4] Diez, P. S.; Micalizio, G. C. Convergent Synthesis of Deoxypropionates. *Angew. Chem. Int. Ed. Engl.* **2012**, *51*, 5152–5156.

[5] Macklin, T. K.; Micalizio, G. C. Convergent and stereospecific synthesis of complex skipped polyenes and polyunsaturated fatty acids. *Nat. Chem.* **2010**, *2*, 638–643.

3. Conformational Analysis of Ligands (2-C, 2-E, 4-C and oleic acid) and Docking to the GPR120 structure (PDB: 8id6).

Methods (Computational): Molecular docking and molecular dynamics simulations were performed using the Schrodinger programs suite. Initial ligand structures were generated using LigPrep with Epik protonation prediction. Ligand docking was performed using Glide XP with an output of 5 minimized poses for each ligand. The receptor grid was generated from the coordinates of GPR120 in the PDB entry 8id6, positioned at the experimentally observed oleic acid site and set to fit ligands of similar size. The same ligand input structures were prepared in size-optimized orthorhombic water boxes with periodic boundaries and simulated using Desmond for a duration of 50ns at constant 300K temperature and 1 bar pressure with frames recorded every 50ps (total 1000 frames). The dihedral angles analysis was performed with the Desmond Simulation Interactions Diagram tool. Finally, the graphics that depict the results of these docking studies were generated with PyMol.

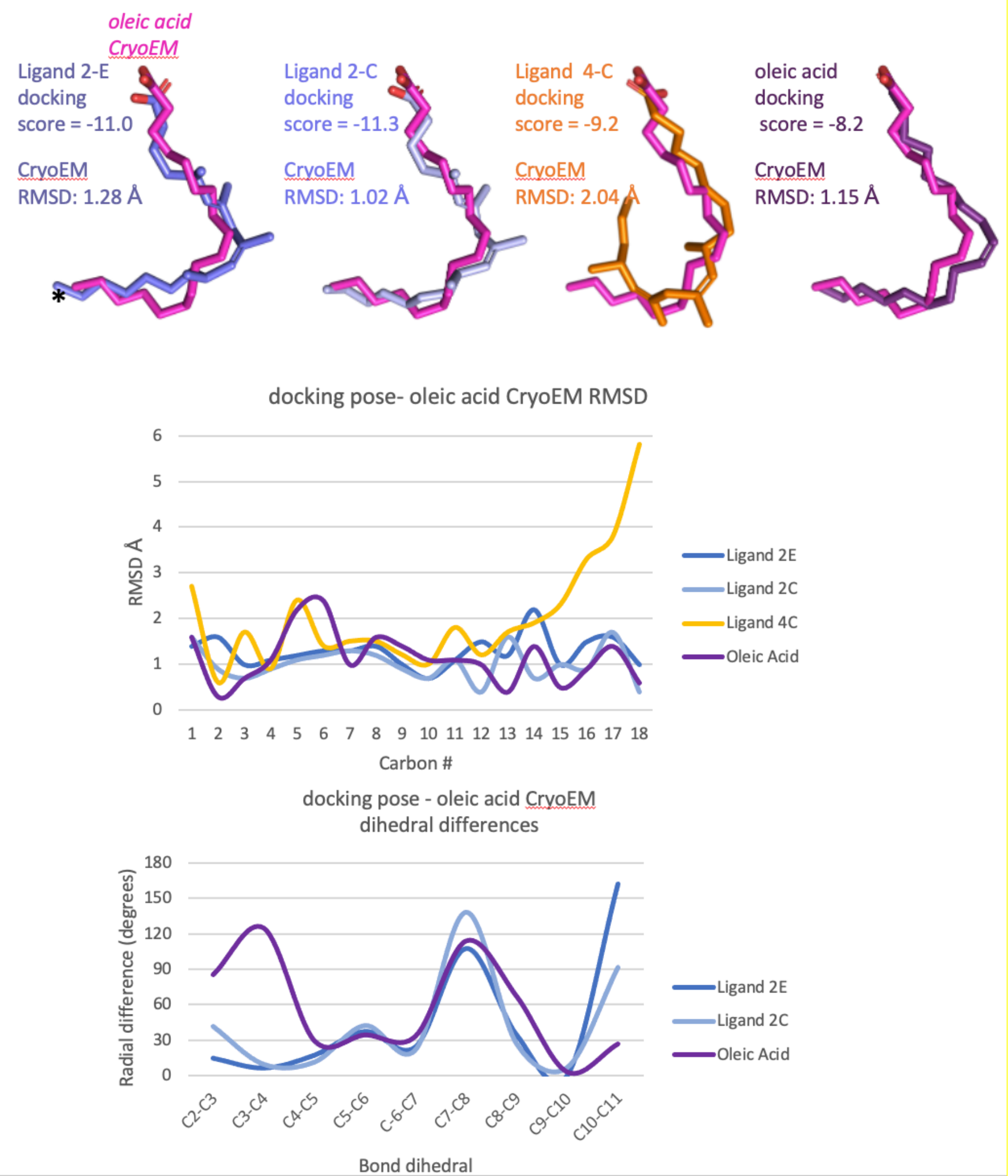

Supplemental Figure 1. Docking of **2-C**, **2-E** and oleic acid to the GPR120 CryoEM structure stripped of the experimentally observed oleic acid molecule (Glide XP; showing the pose with the lowest RMSD from the CryoEM structure out of 5 poses sampled for each ligand). Individual carbon's RMSDs and bond dihedral angle variations from the CryoEM structure are comparable between **2-C**, **2-E** and the docking pose of oleic acid, while the terminus of the aliphatic chain of **4-C** deviates from the experimental conformation, incompatible with the conformational bias of the active ligands (oleic acid, **2-C** and **2-E**) in the region from C9–C15 in this molecule.

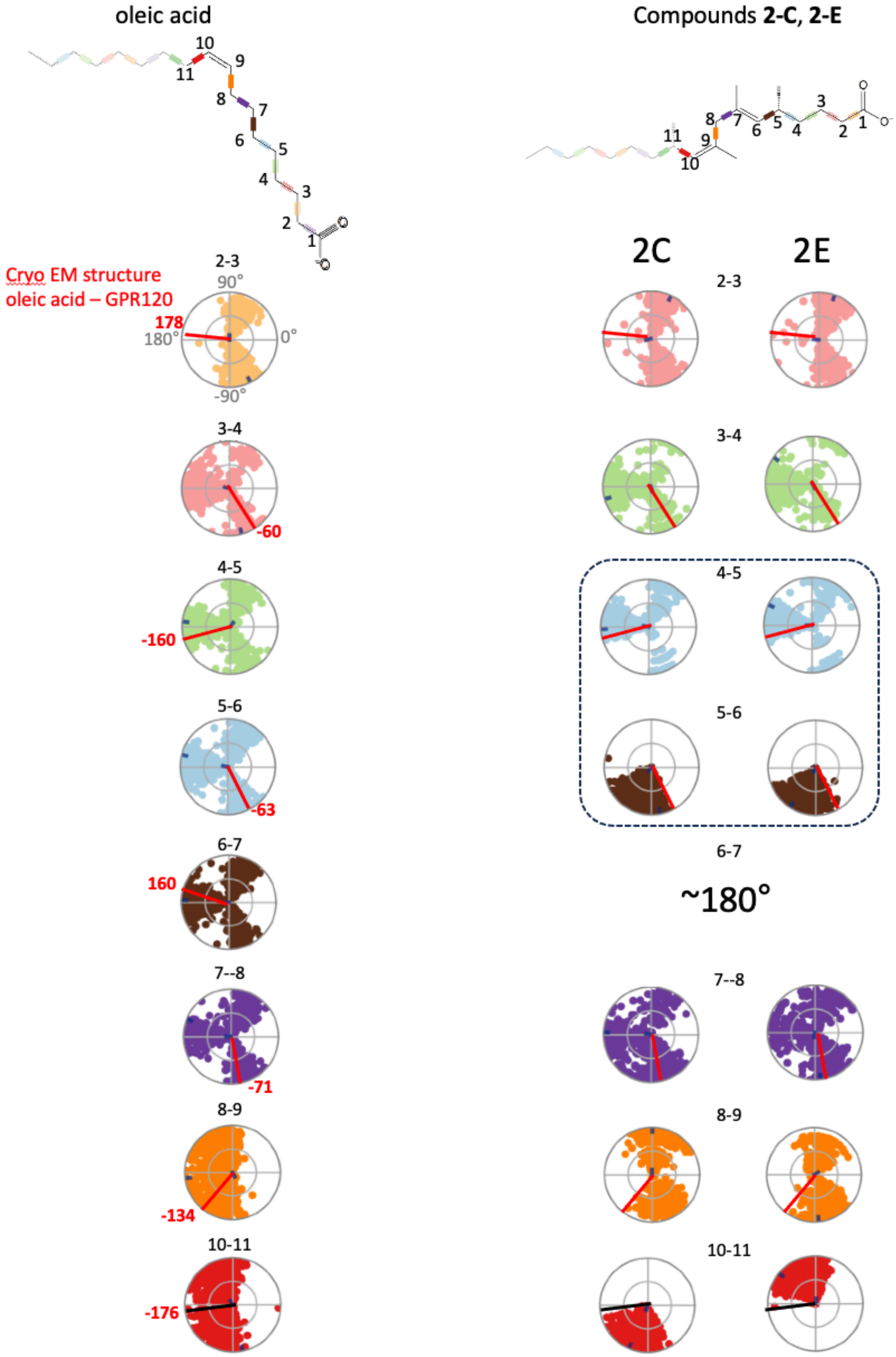

Supplemental Figure 2. Bond angles populations of oleic acid, **2-C** and **2-E** over a 50 ns molecular dynamics simulation of the unbound molecules in water at 300K. The dihedrals measured for the equivalent bonds in the CryoEM structure of oleic acid bound to GPR120 are marked with red lines. The conformational bias in **2-C** and **2-E** at the C4–C5 and C5–C6 bonds encompasses the angles observed in the GPR120-bound conformation of oleic acid.

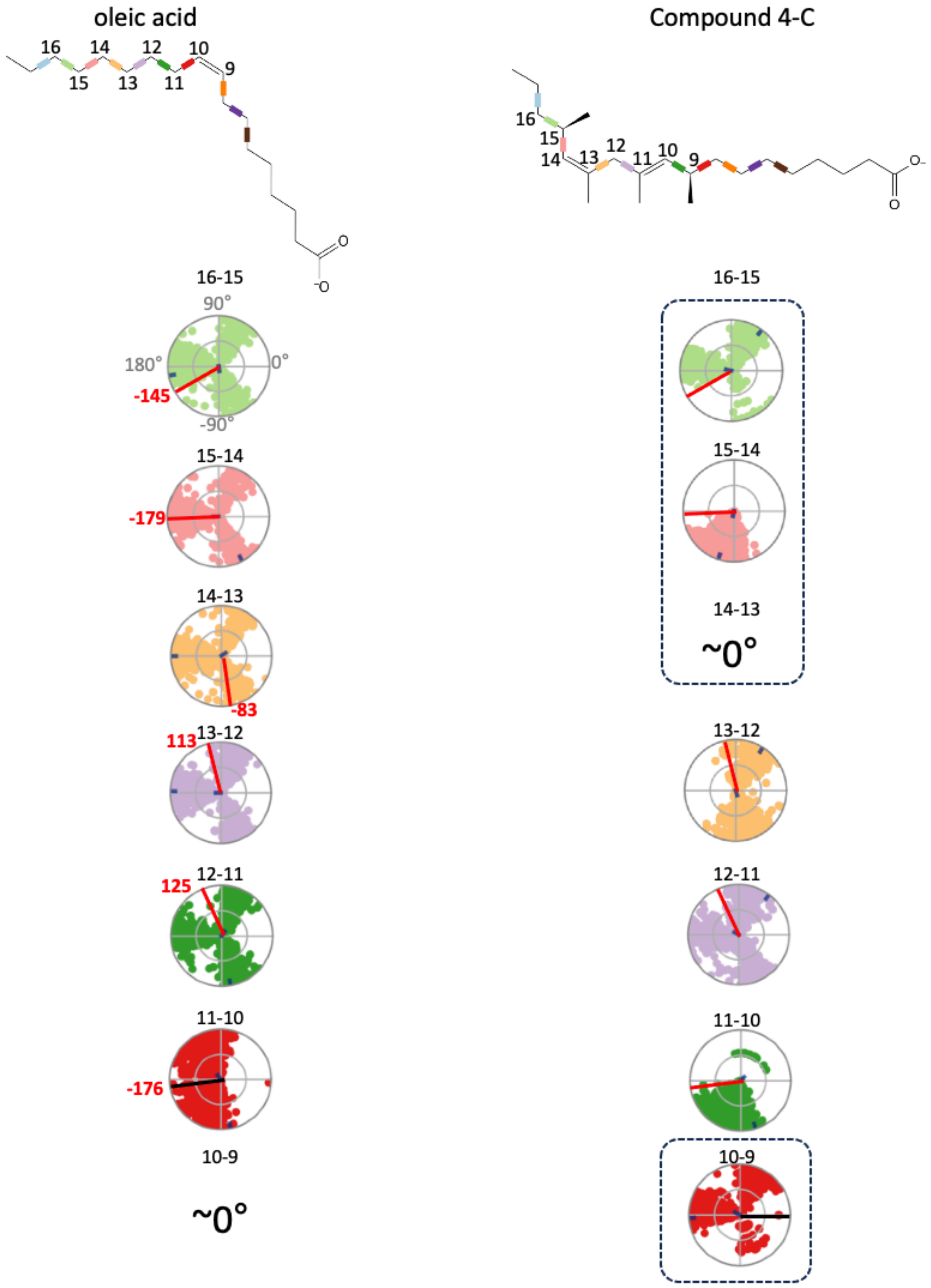

Supplemental Figure 3. Bond angles populations of oleic acid and **4-C** over a 50 ns molecular dynamics simulation of the unbound molecules in water at 300K. The dihedrals measured for the equivalent bonds in the CryoEM structure of oleic acid bound to GPR120 are marked with red lines. The reduced conformational space of compound **4-C** in the C13–C16 region and the missing *cis*-double bond between C9–C10 biases this molecule against the angles observed in the GPR120-bound conformation of oleic acid.

4. Biological Procedures

Functional assays at GPR40 and GPR120 were conducted by Eurofins Discovery services using their PathHunter® β-Arrestin assay that monitors the activation of a GPCR in a homogenous, non-imaging assay format using a technology developed by DiscoverX called Enzyme Fragment Complementation (EFC) with β-galactosidase (β-Gal) as the functional reporter. β-Arrestin GPCR cell lines built on the principles of EFC are engineered to co-express the ProLink™ (PK) tagged GPCR and the Enzyme Acceptor (EA) tagged b-Arrestin. Activation of the GPCR-PK induces b-Arrestin-EA recruitment, forcing complementation of the two b-galactosidase enzyme fragments (EA and PK). The resulting functional enzyme hydrolyzes substrate to generate chemiluminescent signal.

Additional information can be found at: www.eurofinsdiscovery.com/solution/b-arrestin

Below are dose-response curves generated from Eurofins Discovery services for activity of ligands depicted in Figure 6B at GPR40 (FFAR1) and GPR120.

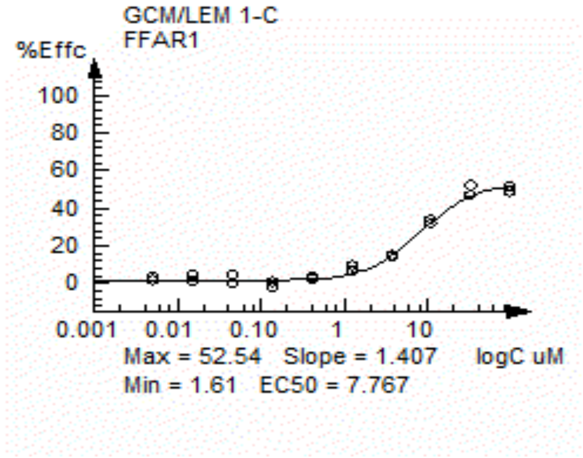

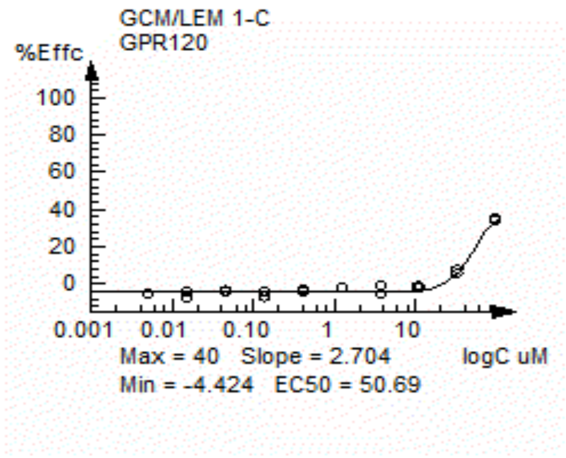

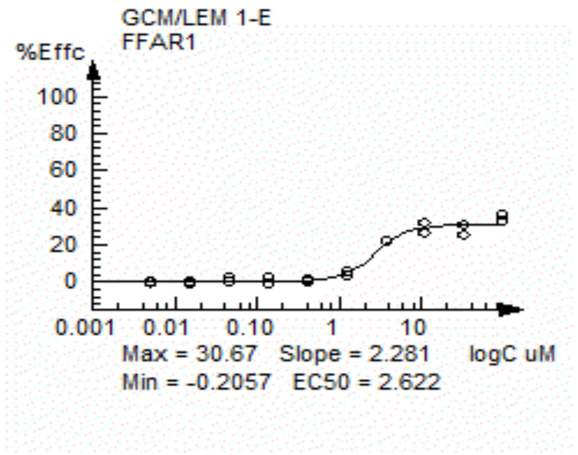

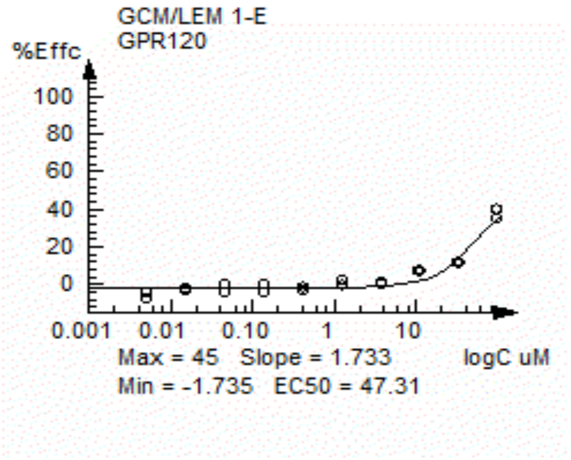

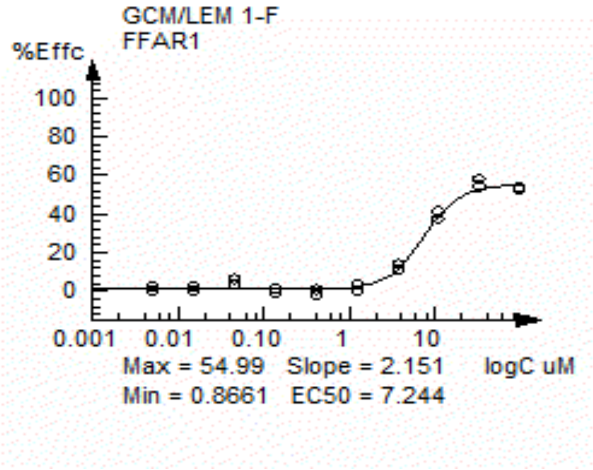
GCM/LEM 1-F
FFAR1
%Effc
100
80
60
40
20
0
0.001
0.01
0.10
1
10
logC uM
Max = 54.99 Slope = 2.151
Min = 0.8661 EC50 = 7.244

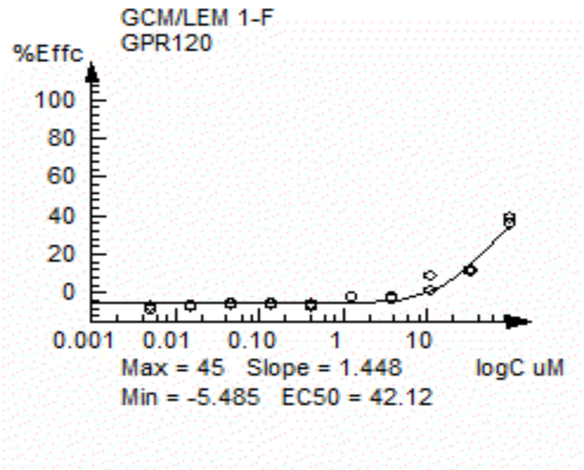
GCM/LEM 1-F
GPR120
%Effc
100
80
60
40
20
0
0.001
0.01
0.10
1
10
logC uM
Max = 45 Slope = 1.448
Min = -5.485 EC50 = 42.12

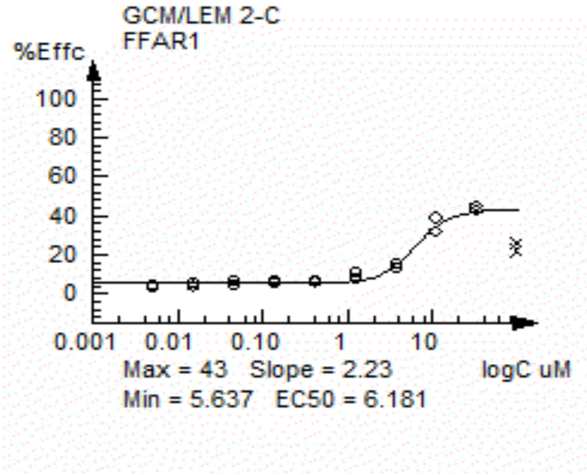
GCM/LEM 2-C
FFAR1
%Effc
100
80
60
40
20
0
0.001
0.01
0.10
1
10
logC uM
Max = 43 Slope = 2.23
Min = 5.637 EC50 = 6.181

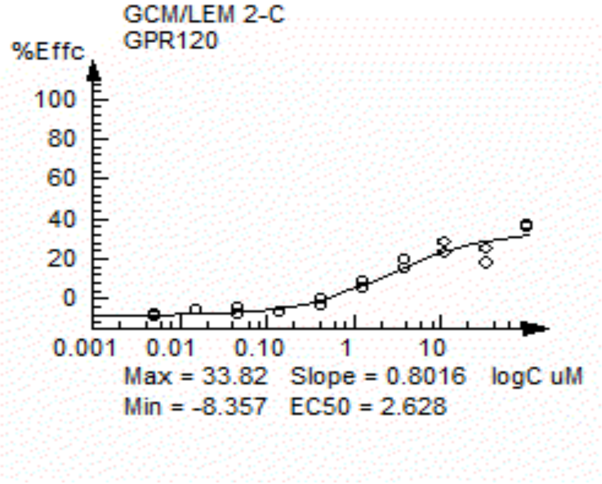
GCM/LEM 2-C
GPR120
%Effc
100
80
60
40
20
0
0.001
0.01
0.10
1
10
logC uM
Max = 33.82 Slope = 0.8016
Min = -8.357 EC50 = 2.628

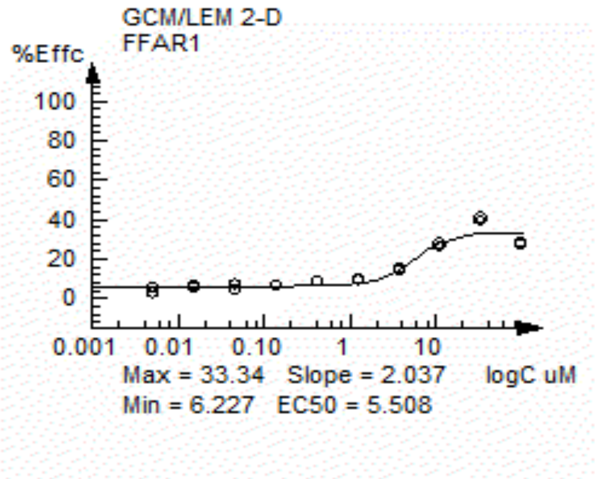
GCM/LEM 2-D
FFAR1
%Effc
100
80
60
40
20
0
0.001
0.01
0.10
1
10
Max = 33.34 Slope = 2.037 logC uM
Min = 6.227 EC50 = 5.508

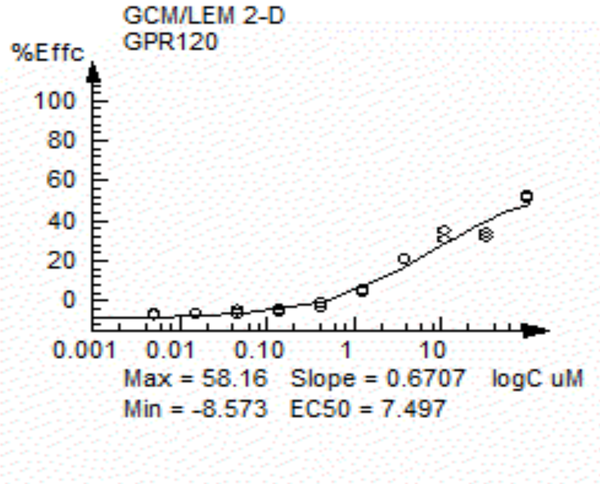
GCM/LEM 2-D
GPR120
%Effc
100
80
60
40
20
0
0.001
0.01
0.10
1
10
Max = 58.16 Slope = 0.6707 logC uM
Min = -8.573 EC50 = 7.497

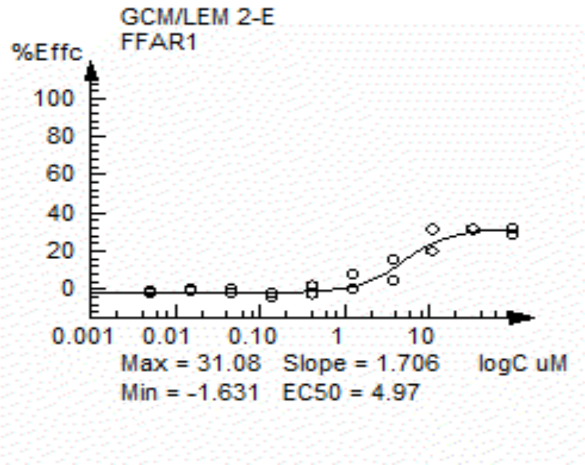
GCM/LEM 2-E
FFAR1
%Effc
100
80
60
40
20
0
0.001
0.01
0.10
1
10
Max = 31.08 Slope = 1.706 logC uM
Min = -1.631 EC50 = 4.97

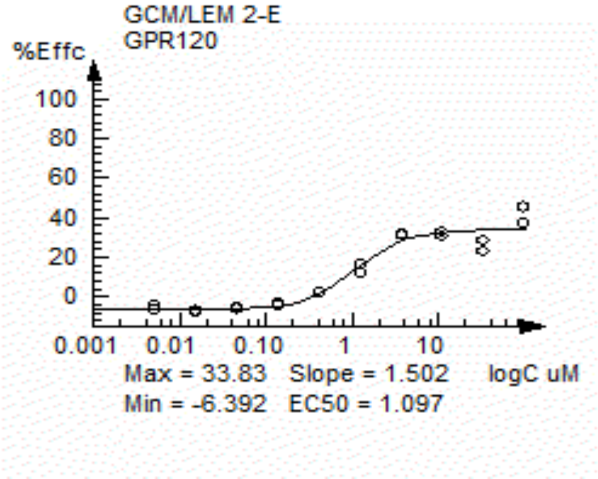
GCM/LEM 2-E
GPR120
%Effc
100
80
60
40
20
0
0.001
0.01
0.10
1
10
Max = 33.83 Slope = 1.502 logC uM
Min = -6.392 EC50 = 1.097

Controls:

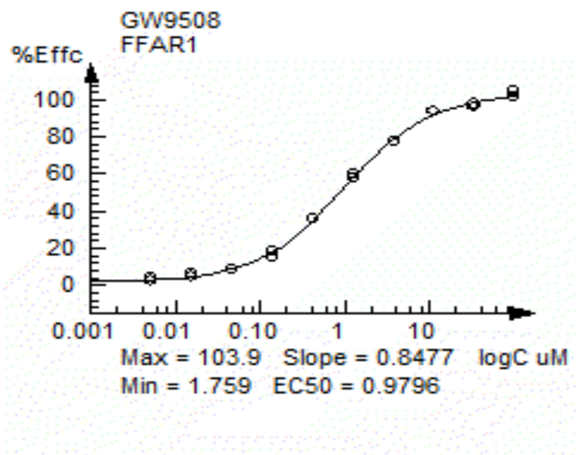
GW9508
FFAR1
%Effc
100
80
60
40
20
0
0.001
0.01
0.10
1
10
Max = 103.9 Slope = 0.8477 logC uM
Min = 1.759 EC50 = 0.9796

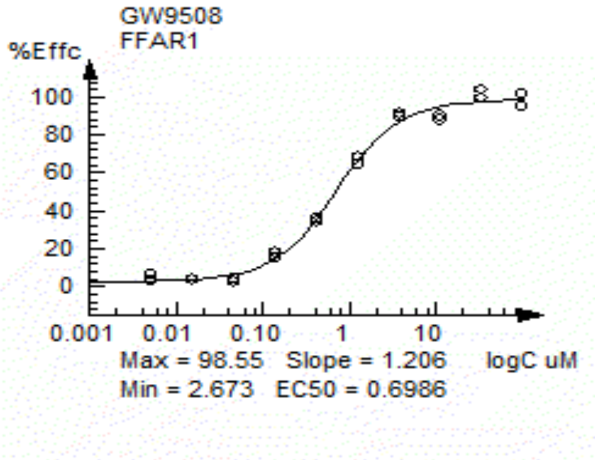
GW9508
FFAR1
%Effc
100
80
60
40
20
0
0.001
0.01
0.10
1
10
Max = 98.55 Slope = 1.206 logC uM
Min = 2.673 EC50 = 0.6986

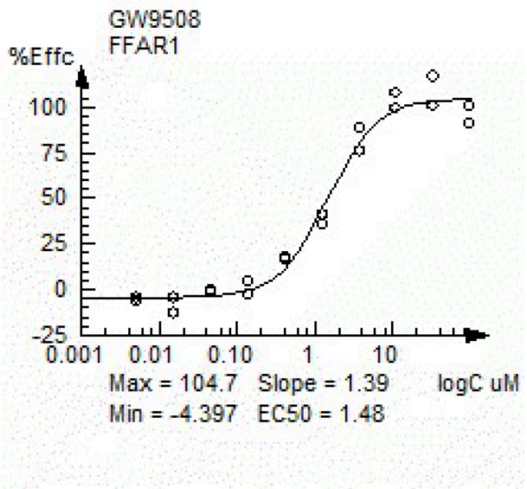

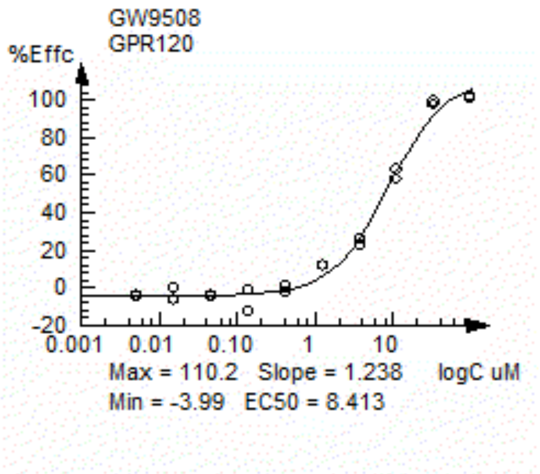

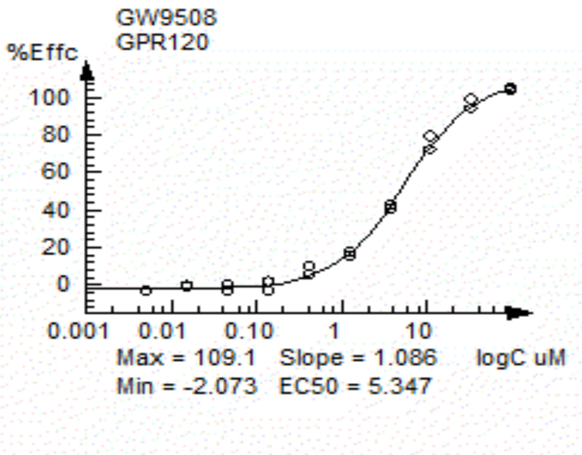

Oleic acid (labeled as LEM 5-A)

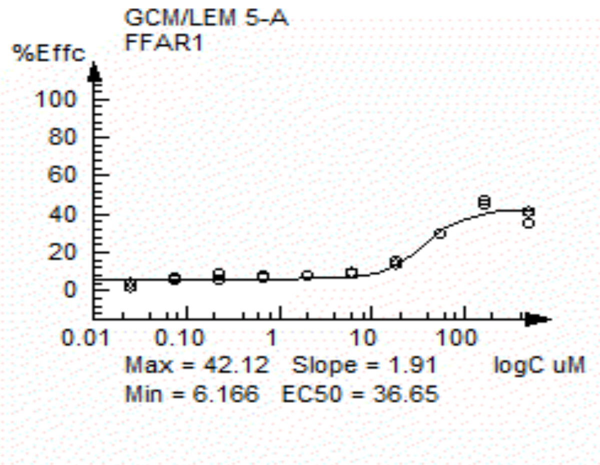

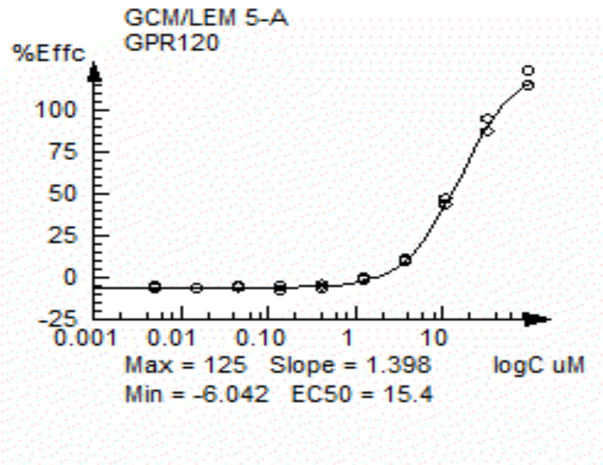

TLX Assay:

Compounds screening in ALPHAscreen
-
TLX Protocol:

Buffer composition	
**TRIS-HCl pH7.5**	20mM
**NaCl**	300mM
**CHAPS**	0.03%
**DTT**	1mM*

* Add DTT at the last minute in the buffer quantity needed for this specific experiment

Consumable needed:

- V bottom 96-well plate for drug dilution (Thermo ref#249944)
- ALPHAplate384 light gray untreated, PerkinElmer (ref# 6005359)
- ALPHAscreen Nickel Chelate Kit. Perkin Elmer (ref# 6760619)

Reagent:	Stock Concentration:	Final Concentration
**TLX Novalix**	98.8uM	250nM
**Biot-RIP140_L6**	100uM	25nM
**ALPHA beads**	5mg/ml	10ug/mL

Plate maps (cf. Annex 1 at the end)

Procedure:

1) Take the volume of buffer you will need for your experiment and add DTT to 1mM

2) Take the protein aliquot needed out of the -80C and thaw it in ice
   a. Proteins are temperature sensitive, keep it in ice all the time.

3) Prepare tube for 4X mix Protein/Biot-Peptide
   a. Incubate for at least 10minutes on Ice

4) Prepare drug dilution(s) at 50X in DMSO (see specifics below)

5) After at least 10min of incubation, add the beads (Acceptor and Donor) to the 4X mix Protein/Biot-Peptide and buffer to make it a 2X mix.
   a. Incubate for at least 15minutes in ice with aluminum foil around the tube (beads are light sensitives)

6) Prepare drug dilutions at 2X in Buffer with DTT (see specifics below)

7) Add drug to the assay plate (ALPHAplate PerkinElmer)
   a. Drugs = 10ul/well

8) After at least 15minutes of incubation, add 10ul of 2X protein/Biot-peptide/Beads mix to assay plate on top of the drug that was previously added in **step 7**
    a. Centrifuge the plate (qPCR centrifuge)
    b. **Incubate at least 60minute** at RT on orbital shaker (300-320RPM) in aluminum foil.

9) Read in the Biotek Neo2ALPHA with the protocol named “OptiPlate384w ALPHA screen_gain150”

Each well in the 384well plate will contain the following:
10ul = 2X drug dilution (step 7)
10ul = 2X protein/Biot-peptide/Beads (step 8)

Total Final Volume per Well = 20ul

Example for 1plate

FULL DOSE RESPONSE (11pts) No replicate

You will need:

- 25 mL of buffer without DTT
- 25 µL of DTT
- 81 µL of His-TLX (at 98.8µM)
- 16µL of Biot-Atrophin (at 100uM)
- 16uL of each beads (Acceptor and Donor)

Perform drug dilutions in two steps:

- 50X drug plate in DMSO
- 2X drug plate in Assay Buffer 1mM DTT

50X Drug Plate:

Example shown if 1st point = 100uM with 1/3 serial dilution for 11points screening

- Drug Plate 50X = highest drug concentration post dilution = 5mM

Perform standard 1:3 dilutions

50X Drug Plate Layout:

Reagent/ Wells	1	2	3	4	5	6	7	8	9	10	11	12
**DMSO**	7uL	10ul	10ul	10ul	10ul	10ul	10ul	10ul	10ul	10ul	10ul	10ul
**10mM Drug**	7uL	5ul carry over	5ul carry over	5ul carry over	5ul carry over	5ul carry over	5ul carry over	5ul carry over	5ul carry over	5ul carry over	5ul carry over	0
**C (uM) 100X**	5000	1666.67	555.56	185.19	61.73	20.58	6.86	2.29	0.76	0.25	0.085	XXX

2X Drug Plate:

- only 10ul per drug dilution is needed (when doing only 1 replicate)

2X Drug Plate Layout:

Reagent /Wells	1	2	3	4	5	6	7	8	9	10	11	12
**Buffer with DTT**	48ul	48ul	48ul	48ul	48ul	48ul	48ul	48ul	48ul	48ul	48ul	48ul
**Drug(from 50X)**	2ul	2ul	2ul	2ul	2ul	2ul	2ul	2ul	2ul	2ul	2ul	2ul
**C (uM) 2X**	200	66.67	22.22	7.41	2.47	0.82	0.27	0.091	0.031	0.01	0.0033	XXX

Dispatch 10uL/well of drug from the 2X plate into the assay plate

4X Protein/Biot-Peptide mix:
Prepare 2ml of this mix per plate

⇒ Dilution factor for TLX = 24.7 => 98.8/(4x1)

⇒ Dilution factor for Biot-ATRO= 125= 100/(4x0.2)

For 2000ul pipette 81 µl of TLX + 16 µl of Biot-ATRO + 1903 µl of Buffer + DTT

2X Protein/Biot-Peptide/Beads mix:
Prepare 4ml of this mix per plate

⇒ Dilution factor for Beads = 250 => 5000/(2x10)

For 4000ul, add 16 µl of Acceptor beads + 16 µl of Donor beads + 1984 µl of Buffer with DTT to the 4X Protein/Biot-Peptide mix

Dispatch 10uL per well of protein/Biot-ATRO/Beads mix on top of the drug in the assay plate. Incubate for at least 60minutes on orbital shaker (300-320RPM) in the dark (aluminum foil)

Data Analysis

This assay is a displacement assay. Meaning that we are looking at the displacement of the -Biot-Peptide induce by the binding of the test drug. If the test drug is binding to the receptor we should see a decrease of the signal (ALPHA).
If we do not see a decrease, it can mean two things:

- The test drug does not bind the receptor
- The test drug binds the receptor but do not induce a displacement of the biot-Peptide

Procedure:

1. Do an average of your DMSO value **per plate** and divide all of your test values, **from the same plate**, by this DMSO average.
    a. DMSO is your maximum binding in this specific condition

2. Add the Normalized value in Prisme and transform them using: X=Log(X)

3. Plot the transformed value a 1st time using “Log(inhibitor vs. response – Variable slope (four parameter)” in the “Dose-response-Inhibition” folder of equation to obtain the IC50

Annex 1: Plate maps

6 points screening (1 replicate):

	1	2	3	4	5	6	7	8	9	10	11	12	13	14	15	16	17	18	19	20	21	22	23	24	
A	A1	B1	A2	B2	A3	B3	A4	B4	A5	B5	A6	B6	A7	B7	A8	B8	A9	B9	A10	B10	A11	B11	A12	B12	Plate1
B	C1	D1	C2	D2	C3	D3	C4	D4	C5	D5	C6	D6	C7	D7	C8	D8	C9	D9	C10	D10	C11	D11	C12	D12	
C	E1	F1	E2	F2	E3	F3	E4	F4	E5	F5	E6	F6	E7	F7	E8	F8	E9	F9	E10	F10	E11	F11	E12	F12	
D	G1	H1	G2	H2	G3	H3	G4	H4	G5	H5	G6	H6	G7	H7	G8	H8	G9	H9	G10	H10	G11	H11	G12	H12	
E	A1	B1	A2	B2	A3	B3	A4	B4	A5	B5	A6	B6	A7	B7	A8	B8	A9	B9	A10	B10	A11	B11	A12	B12	Plate2
F	C1	D1	C2	D2	C3	D3	C4	D4	C5	D5	C6	D6	C7	D7	C8	D8	C9	D9	C10	D10	C11	D11	C12	D12	
G	E1	F1	E2	F2	E3	F3	E4	F4	E5	F5	E6	F6	E7	F7	E8	F8	E9	F9	E10	F10	E11	F11	E12	F12	
H	G1	H1	G2	H2	G3	H3	G4	H4	G5	H5	G6	H6	G7	H7	G8	H8	G9	H9	G10	H10	G11	H11	G12	H12	
I	A1	B1	A2	B2	A3	B3	A4	B4	A5	B5	A6	B6	A7	B7	A8	B8	A9	B9	A10	B10	A11	B11	A12	B12	Plate3
J	C1	D1	C2	D2	C3	D3	C4	D4	C5	D5	C6	D6	C7	D7	C8	D8	C9	D9	C10	D10	C11	D11	C12	D12	
K	E1	F1	E2	F2	E3	F3	E4	F4	E5	F5	E6	F6	E7	F7	E8	F8	E9	F9	E10	F10	E11	F11	E12	F12	
L	G1	H1	G2	H2	G3	H3	G4	H4	G5	H5	G6	H6	G7	H7	G8	H8	G9	H9	G10	H10	G11	H11	G12	H12	
M	A1	B1	A2	B2	A3	B3	A4	B4	A5	B5	A6	B6	A7	B7	A8	B8	A9	B9	A10	B10	A11	B11	A12	B12	Plate4
N	C1	D1	C2	D2	C3	D3	C4	D4	C5	D5	C6	D6	C7	D7	C8	D8	C9	D9	C10	D10	C11	D11	C12	D12	
O	E1	F1	E2	F2	E3	F3	E4	F4	E5	F5	E6	F6	E7	F7	E8	F8	E9	F9	E10	F10	E11	F11	E12	F12	
P	G1	H1	G2	H2	G3	H3	G4	H4	G5	H5	G6	H6	G7	H7	G8	H8	G9	H9	G10	H10	G11	H11	G12	H12	
													C+	DMSO											

Plate1

	1–6	7–12
A	Compoud 01	Compoud 33
B	Compoud 02	Compoud 34
C	Compoud 03	Compoud 35
D	Compoud 04	Compoud 36
E	Compoud 05	Compoud 37
F	Compoud 06	Compoud 38
G	Compoud 07	Compoud 39
H	Compoud 08	Compoud 40

Plate2

	1–6	7–12
A	Compoud 09	Compoud 41
B	Compoud 10	Compoud 42
C	Compoud 11	Compoud 43
D	Compoud 12	Compoud 44
E	Compoud 13	Compoud 45
F	Compoud 14	Compoud 46
G	Compoud 15	Compoud 47
H	Compoud 16	Compoud 48

Plate3

	1–6	7–12
A	Compoud 17	Compoud 49
B	Compoud 18	Compoud 50
C	Compoud 19	Compoud 51
D	Compoud 20	Compoud 52
E	Compoud 21	Compoud 53
F	Compoud 22	Compoud 54
G	Compoud 23	Compoud 55
H	Compoud 24	Compoud 56

Plate4

	1–6	7–12
A	Compoud 25	Compoud 57
B	Compoud 26	Compoud 58
C	Compoud 27	Compoud 59
D	Compoud 28	Compoud 60
E	Compoud 29	Compoud 61
F	Compoud 30	Compoud 62
G	Compoud 31	Compoud 63
H	Positive control	DMSO

11 points screening:

	1	2	3	4	5	6	7	8	9	10	11	12	13	14	15	16	17	18	19	20	21	22	23	24	
A	A1	B1	A2	B2	A3	B3	A4	B4	A5	B5	A6	B6	A7	B7	A8	B8	A9	B9	A10	B10	A11	B11	A12	B12	
B	C1	D1	C2	D2	C3	D3	C4	D4	C5	D5	C6	D6	C7	D7	C8	D8	C9	D9	C10	D10	C11	D11	C12	D12	Plate1
C	E1	F1	E2	F2	E3	F3	E4	F4	E5	F5	E6	F6	E7	F7	E8	F8	E9	F9	E10	F10	E11	F11	E12	F12	
D	G1	H1	G2	H2	G3	H3	G4	H4	G5	H5	G6	H6	G7	H7	G8	H8	G9	H9	G10	H10	G11	H11	G12	H12	
E	A1	B1	A2	B2	A3	B3	A4	B4	A5	B5	A6	B6	A7	B7	A8	B8	A9	B9	A10	B10	A11	B11	A12	B12	
F	C1	D1	C2	D2	C3	D3	C4	D4	C5	D5	C6	D6	C7	D7	C8	D8	C9	D9	C10	D10	C11	D11	C12	D12	Plate2
G	E1	F1	E2	F2	E3	F3	E4	F4	E5	F5	E6	F6	E7	F7	E8	F8	E9	F9	E10	F10	E11	F11	E12	F12	
H	G1	H1	G2	H2	G3	H3	G4	H4	G5	H5	G6	H6	G7	H7	G8	H8	G9	H9	G10	H10	G11	H11	G12	H12	
I	A1	B1	A2	B2	A3	B3	A4	B4	A5	B5	A6	B6	A7	B7	A8	B8	A9	B9	A10	B10	A11	B11	A12	B12	
J	C1	D1	C2	D2	C3	D3	C4	D4	C5	D5	C6	D6	C7	D7	C8	D8	C9	D9	C10	D10	C11	D11	C12	D12	Plate3
K	E1	F1	E2	F2	E3	F3	E4	F4	E5	F5	E6	F6	E7	F7	E8	F8	E9	F9	E10	F10	E11	F11	E12	F12	
L	G1	H1	G2	H2	G3	H3	G4	H4	G5	H5	G6	H6	G7	H7	G8	H8	G9	H9	G10	H10	G11	H11	G12	H12	
M	A1	B1	A2	B2	A3	B3	A4	B4	A5	B5	A6	B6	A7	B7	A8	B8	A9	B9	A10	B10	A11	B11	A12	B12	
N	C1	D1	C2	D2	C3	D3	C4	D4	C5	D5	C6	D6	C7	D7	C8	D8	C9	D9	C10	D10	C11	D11	C12	D12	Plate4
O	E1	F1	E2	F2	E3	F3	E4	F4	E5	F5	E6	F6	E7	F7	E8	F8	E9	F9	E10	F10	E11	F11	E12	F12	
P	G1	H1	G2	H2	G3	H3	G4	H4	G5	H5	G6	H6	G7	H7	G8	H8	G9	H9	G10	H10	G11	H11	G12	H12	
		C+																					DMSO		

Plate1

	1	2	3	4	5	6	7	8	9	10	11	12
A	Compoud 01											DMSO
B	Compoud 02											
C	Compoud 03											
D	Compoud 04											
E	Compoud 05											
F	Compoud 06											
G	Compoud 07											
H	Compoud 08											

Plate2

	1	2	3	4	5	6	7	8	9	10	11	12
A	Compoud 09											DMSO
B	Compoud 10											
C	Compoud 11											
D	Compoud 12											
E	Compoud 13											
F	Compoud 14											
G	Compoud 15											
H	Compoud 16											

Plate3

	1	2	3	4	5	6	7	8	9	10	11	12
A	Compoud 17											DMSO
B	Compoud 18											
C	Compoud 19											
D	Compoud 20											
E	Compoud 21											
F	Compoud 22											
G	Compoud 23											
H	Compoud 24											

Plate4

	1	2	3	4	5	6	7	8	9	10	11	12
A	Compoud 25											DMSO
B	Compoud 26											
C	Compoud 27											
D	Compoud 28											
E	Compoud 29											
F	Compoud 30											
G	Compoud 31											
H	Positive control											

TLX – ALPHAscreen_ATRO - Protocol - v1

TLX Ligand Sensing Assay –

A biochemical assay was used to characterize ligand binding to TLX by detecting alterations in the ability of the TLX ligand binding domain to recruit an NR box peptide derived from the nuclear receptor-interacting protein 1 (NRIP1; receptor-interacting protein 140 (RIP140) transcriptional cofactor. RIP140 has been previously identified as a TLX interacting protein (Corso-Diaz et al., 2016). AlphaScreen (Perkin Elmer, Shelton, CT) technology was used to detect the TLX-RIP140 NR box peptide interaction. The AlphaScreen assay was performed as we have previously described using other nuclear receptors including ROR, FXR, LXR, VDR and RXR(Bettoun et al., 2003; Bramlett et al., 2003; Houck et al., 2004; Kumar et al.; Wang et al., 2010) using the N-terminal his-tagged LBD of TLX (NovAliX SAS, Strasbourg, France) and an N-terminal biotinylated RIP140-L6 peptide with a N-terminal KGG linker (**KGG**QDTSKNSKLNSHQKVTLLQLLLGHKNEENV(aa484-513).

Bettoun DJ, Burris TP, Houck KA, Buck DW, 2nd, Stayrook KR, Khalifa B, Lu J, Chin WW and Nagpal S (2003) Retinoid X receptor is a nonsilent major contributor to vitamin D receptor-mediated transcriptional activation. *Mol Endocrinol* **17**:2320-2328.

Bramlett KS, Houck KA, Borchert KM, Dowless MS, Kulanthaivel P, Zhang YY, Beyer TP, Schmidt R, Thomas JS, Michael LF, Barr R, Montrose C, Eacho PI, Cao GQ and Burris TP (2003) A natural product ligand of the oxysterol receptor, liver X receptor. *Journal of Pharmacology and Experimental Therapeutics* **307**:291-296.

Corso-Diaz X, de Leeuw CN, Alonso V, Melchers D, Wong BK, Houtman R and Simpson EM (2016) Co-activator candidate interactions for orphan nuclear receptor NR2E1. *BMC Genomics* **17**:832.

Houck KA, Borchert KM, Hepler CD, Thomas JS, Bramlett KS, Michael LF and Burris TP (2004) T0901317 is a dual LXR/FXR agonist. *Mol Genet Metab* **83**:184-187.

Kumar N, Solt LA, Conkright JJ, Wang Y, Istrate MA, Busby SA, Garcia-Ordonez RD, Burris TP and Griffin PR The benzenesulfoamide T0901317 [N-(2,2,2-trifluoroethyl)-N-[4-[2,2,2-trifluoro-1-hydroxy-1-(trifluorometh yl)ethyl]phenyl]-benzenesulfonamide] is a novel retinoic acid receptor-related orphan receptor-alpha/gamma inverse agonist. *Mol Pharmacol* **77**:228-236.

Wang Y, Kumar N, Nuhant P, Cameron MD, Istrate MA, Roush WR, Griffin PR and Burris TP (2010) Identification of SR1078, a Synthetic Agonist for the Orphan Nuclear Receptors RORA and RORG. *ACS Chemical Biology*:1029-1034.

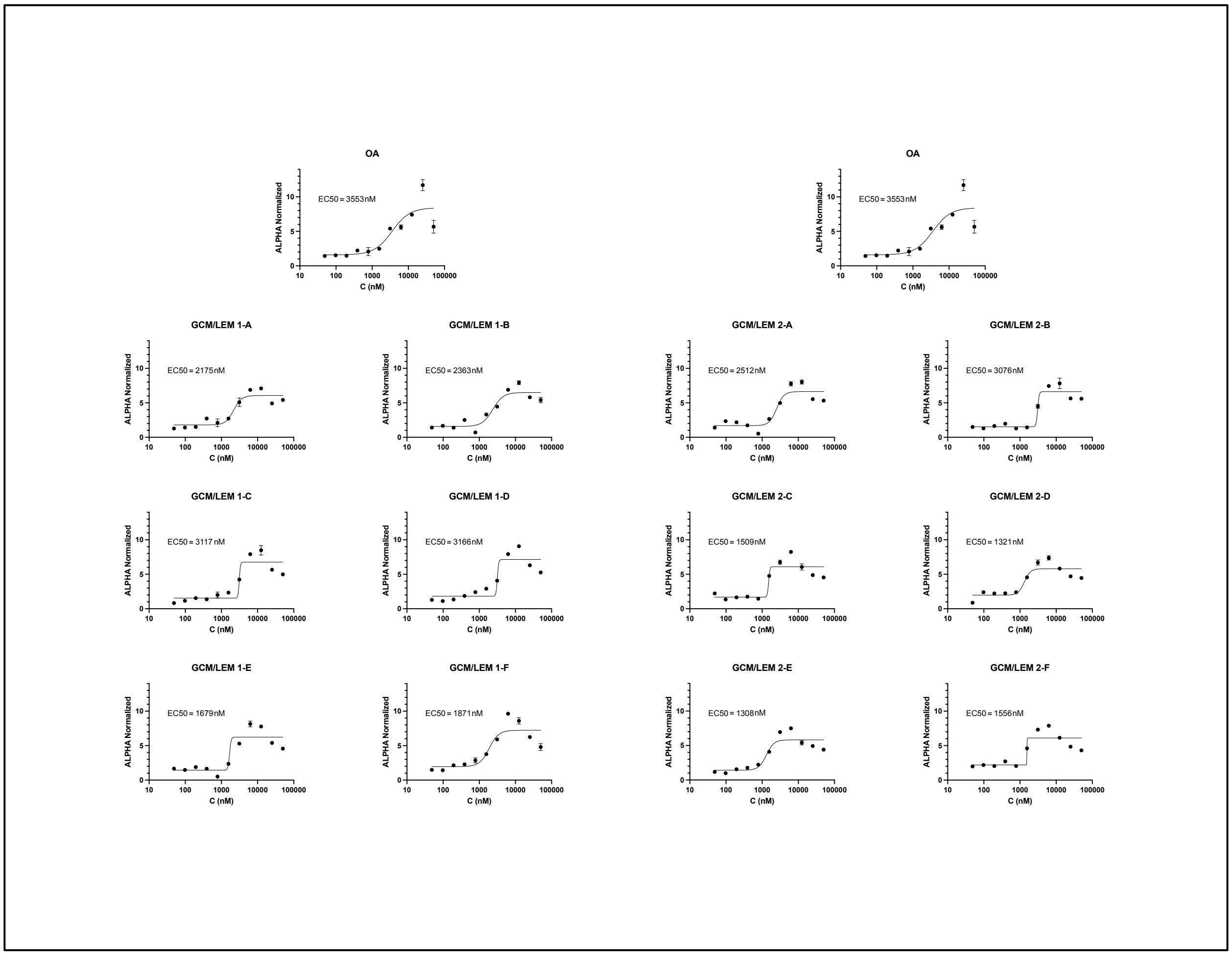
OA
EC50 = 3553nM
OA
EC50 = 3553nM
GCM/LEM 1-A
EC50 = 2175nM
GCM/LEM 1-B
EC50 = 2363nM
GCM/LEM 2-A
EC50 = 2512nM
GCM/LEM 2-B
EC50 = 3076nM
GCM/LEM 1-C
EC50 = 3117nM
GCM/LEM 1-D
EC50 = 3166nM
GCM/LEM 2-C
EC50 = 1509nM
GCM/LEM 2-D
EC50 = 1321nM
GCM/LEM 1-E
EC50 = 1679nM
GCM/LEM 1-F
EC50 = 1871nM
GCM/LEM 2-E
EC50 = 1308nM
GCM/LEM 2-F
EC50 = 1556nM
ALPHA Normalized
C (nM)

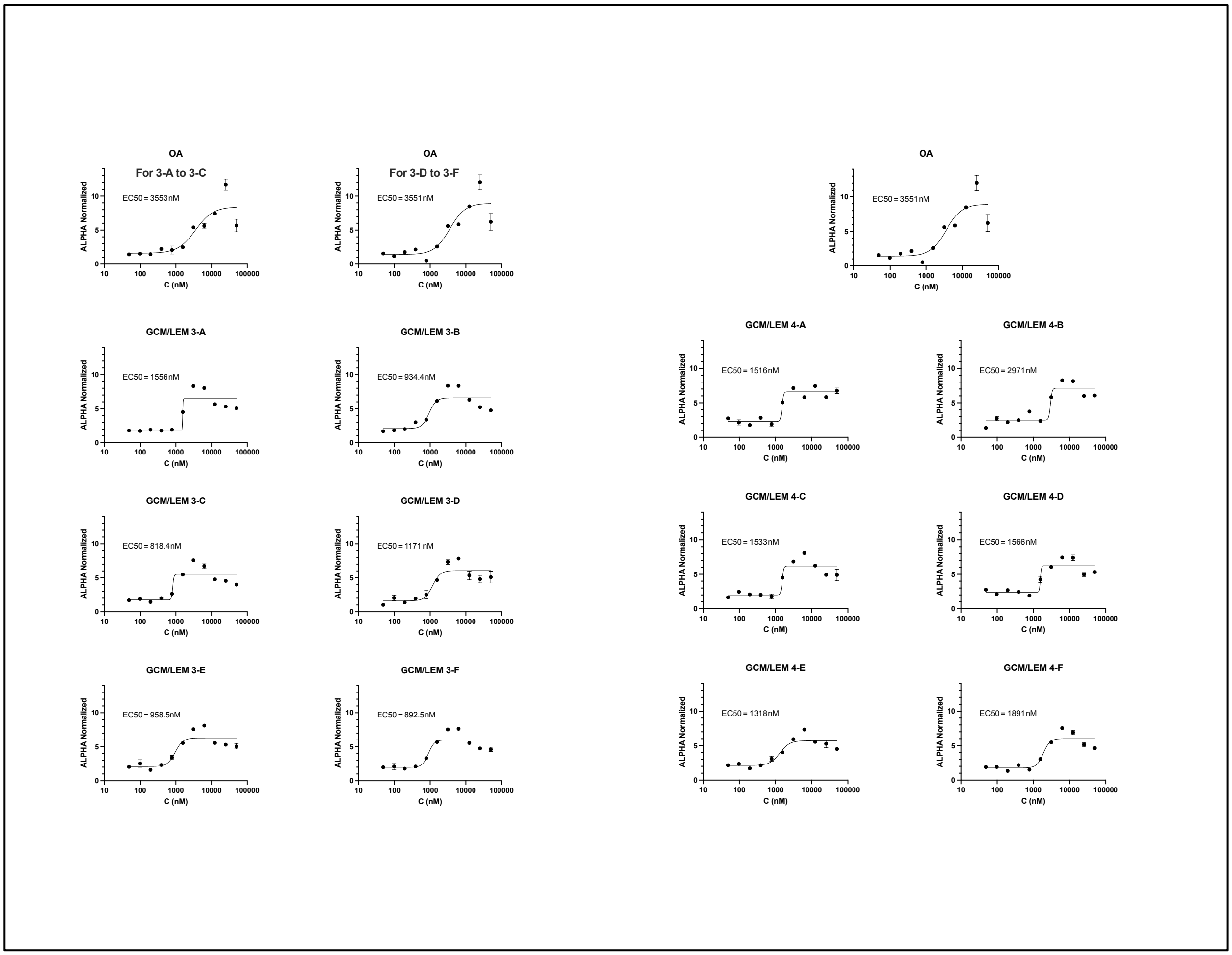
OA
For 3-A to 3-C
EC50 = 3553nM
OA
For 3-D to 3-F
EC50 = 3551nM
OA
EC50 = 3551nM
GCM/LEM 3-A
EC50 = 1556nM
GCM/LEM 3-B
EC50 = 934.4nM
GCM/LEM 4-A
EC50 = 1516nM
GCM/LEM 4-B
EC50 = 2971nM
GCM/LEM 3-C
EC50 = 818.4nM
GCM/LEM 3-D
EC50 = 1171nM
GCM/LEM 4-C
EC50 = 1533nM
GCM/LEM 4-D
EC50 = 1566nM
GCM/LEM 3-E
EC50 = 958.5nM
GCM/LEM 3-F
EC50 = 892.5nM
GCM/LEM 4-E
EC50 = 1318nM
GCM/LEM 4-F
EC50 = 1891nM
ALPHA Normalized
C (nM)

Metabolic Stability Assays:

6. COMPOUNDS

6.1. Test Compounds

Client Compound ID	Compound ID	Reference Number	Batch Number	FW	MW	Purity	Received Form	Stock solution	Flag
GCM/LEM 1-E	100066398-1	-	-	336.56	336.56	-	Liquid	1.E-02 M DMSO	-
GCM/LEM 3-C	100066398-2	-	-	336.56	336.56	-	Liquid	1.E-02 M DMSO	-

FW: Formula Weight - MW: Molecular Weight

6.2. Reference Compounds

In each experiment and if applicable, the respective reference compounds were tested concurrently with GCM/LEM 1-E and GCM/LEM 3-C, and the data were compared with historical values determined at Eurofins. The experiment was accepted in accordance with Eurofins validation Standard Operating Procedure.

7. RESULTS

7.1. ADME-Tox: *In Vitro* Metabolism

7.1.1. Test Compound Results

Compound I.D.	Client Compound I.D.	Test Concentration	Incubation Time(minutes)	% Compound Remaining (% remaining) 1st	2nd	3rd	Mean	Half-Life (minutes) 1st	2nd	3rd	Mean	Clint
**Intrinsic clearance (cryopreserved hepatocytes, human)**												
100066398-1	GCM/LEM 1-E	1.0E-06 M	0	100.0	100.0	100.0	100	206.7	130.1	267.2	201	5.4
100066398-1	GCM/LEM 1-E	1.0E-06 M	30	99.4	103.0	86.6	96					
100066398-1	GCM/LEM 1-E	1.0E-06 M	60	83.9	103.8	106.9	98					
100066398-1	GCM/LEM 1-E	1.0E-06 M	90	79.1	80.4	96.1	85					
100066398-1	GCM/LEM 1-E	1.0E-06 M	120	67.8	50.9	64.3	61					
100066398-2	GCM/LEM 3-C	1.0E-06 M	0	100.0	100.0	100.0	100	118.3	111.7	110.9	114	8.7
100066398-2	GCM/LEM 3-C	1.0E-06 M	30	97.3	95.1	96.7	96					
100066398-2	GCM/LEM 3-C	1.0E-06 M	60	72.5	71.7	79.8	75					
100066398-2	GCM/LEM 3-C	1.0E-06 M	90	59.1	54.4	{53.2}	57					
100066398-2	GCM/LEM 3-C	1.0E-06 M	120	53.3	52.1	48.9	51					

Note: Unit of Clint is µL/min/mg for microsomes, S9 and UGT assays; µL/min/pmol for CYP assays; µL/min/Million cells for hepatocyte assays

7.1.2. Reference Compound Results

Compound I.D.	Test Concentration	Half-Life (minutes) 1st	2nd	3rd	Mean	Clint
**Intrinsic clearance (cryopreserved hepatocytes, human)**						
Flurazepam	1.0E-06 M	52.9	47.8	53.7	51	19.3
HFC	1.0E-06 M	20.9	21.1	20.8	21	47.3
Naloxone	1.0E-06 M	96.4	88.2	111.1	99	10.1
Propranolol	1.0E-06 M	181.8	183.1	195.1	187	5.3

Note: Unit of Clint is µL/min/mg for microsomes, S9 and UGT assays; µL/min/pmol for CYP assays; µL/min/Million cells for hepatocyte assays

8. MATERIALS AND METHODS

8.1. Experimental Conditions

Minor variations to the experimental protocol described below may have occurred during the testing, they have no impact on the quality of the results obtained.

8.1.1. ADME-Tox: *In Vitro* Metabolism

Assay	Source	Substrate	Incubation	Measured Component	Detection Method	Bibl.
**ADME**						
**Intrinsic clearance (cryopreserved hepatocytes, human)**	Human cryopreserved hepatocytes (0.7 million viable cells/mL)	Test compound	0, 0.5, 1, 1.5, 2 hr 37°C	Test compound	HPLC-MS/MS	828

8.2. Analysis and expression of results

8.2.1. ADME-Tox: *In Vitro* Metabolism

Intrinsic Clearance (microsomes, S9, cryopreserved hepatocytes, recombinant CYP, recombinant UGT)

Metabolic stability, expressed as percent of the parent compound remaining, was calculated by comparing the peak area of the compound at the time point relative to that at time-0. The half-life ($T_{1/2}$) was estimated from the slope of the initial linear range of the logarithmic curve of compound remaining (%) vs. time, assuming the first-order kinetics. The apparent intrinsic clearance (CL_{int}, in µL/min/pmol, µL/min/mg or µL/min/Mcell) was calculated according to the following formula:

$$CL_{int} = \frac{0.693}{T_{1/2} * (\text{mg protein}/\mu L \text{ or million cells}/\mu L \text{ or pmol CYP isozyme}/\mu L)}$$

8.2. Analysis and expression of results

8.2.1. ADME-Tox: *In Vitro* Metabolism

Intrinsic Clearance (microsomes, S9, cryopreserved hepatocytes, recombinant CYP, recombinant UGT)

Metabolic stability, expressed as percent of the parent compound remaining, was calculated by comparing the peak area of the compound at the time point relative to that at time-0. The half-life ($T_{1/2}$) was estimated from the slope of the initial linear range of the logarithmic curve of compound remaining (%) vs. time, assuming the first-order kinetics. The apparent intrinsic clearance (CL_{int}, in µL/min/pmol, µL/min/mg or µL/min/Mcell) was calculated according to the following formula:

$$CL_{int} = \frac{0.693}{T_{1/2} * (\text{mg protein}/\mu L \text{ or million cells}/\mu L \text{ or pmol CYP isozyme}/\mu L)}$$

4. NMR Spectra of Novel Compounds

^{1}H NMR (600 MHz, C_6D_6) and ^{13}C NMR (150 MHz, C_6D_6) of **10**

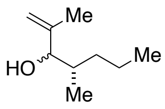

10

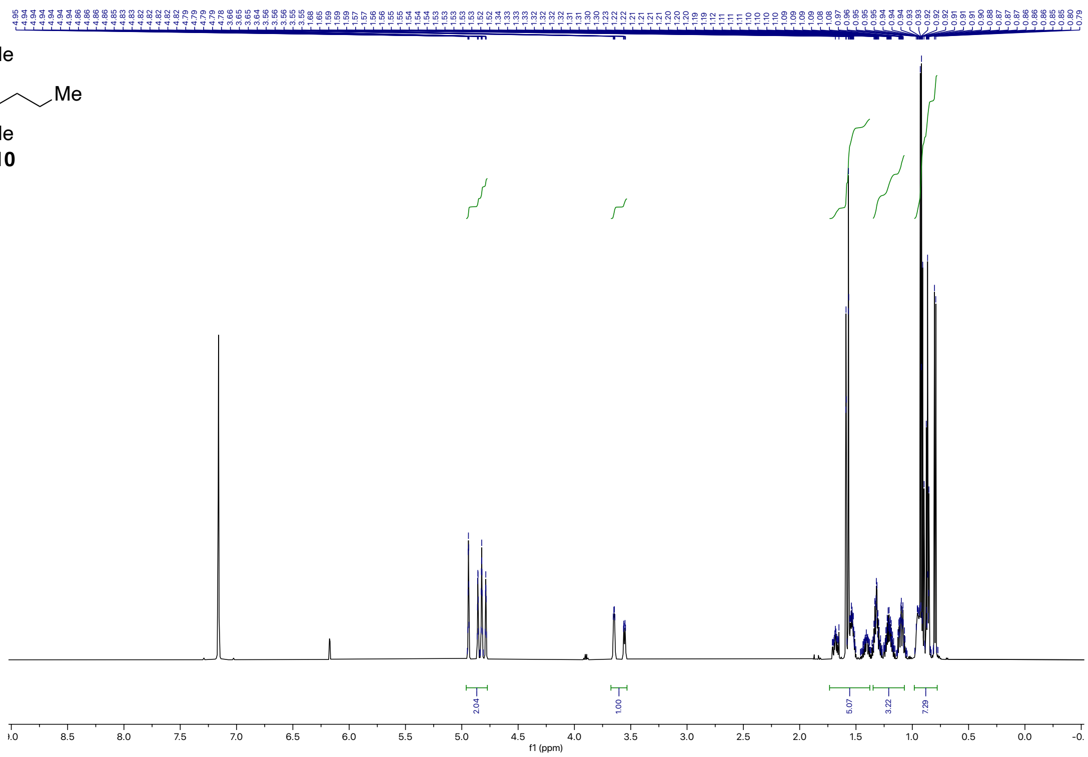

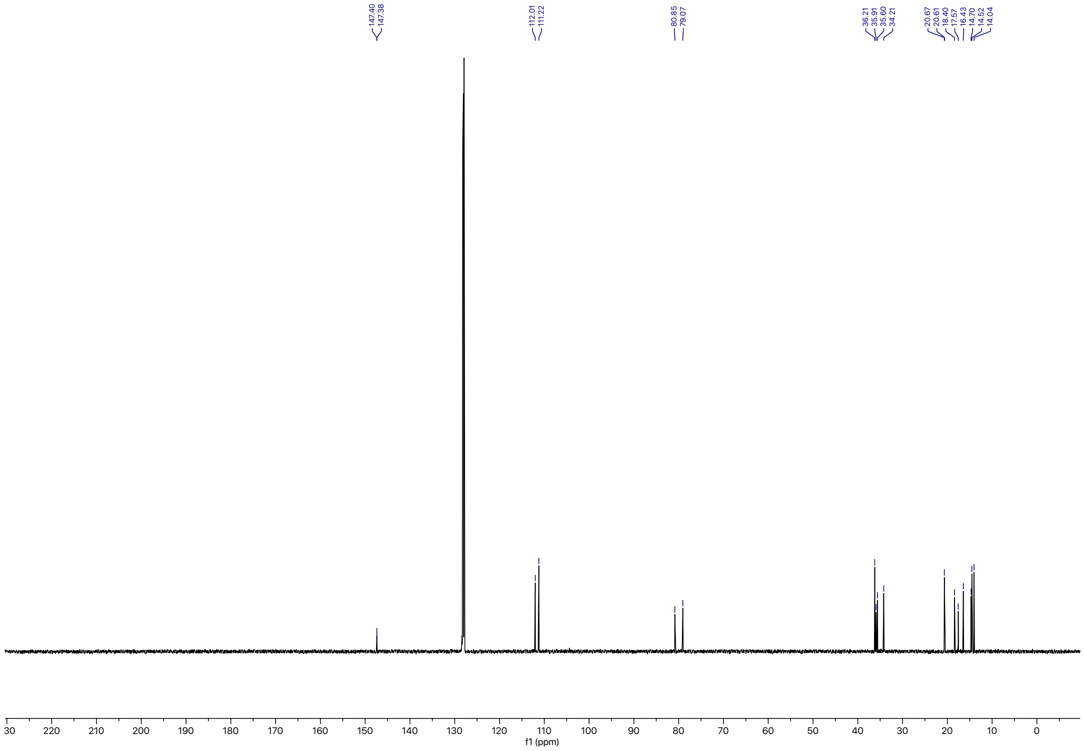

^{1}H NMR (600 MHz, C_6D_6) and ^{13}C NMR (150 MHz, C_6D_6) of **11** (Diastereomer ^{13}C signal at 147.4 ppm not selectable via NMR software)

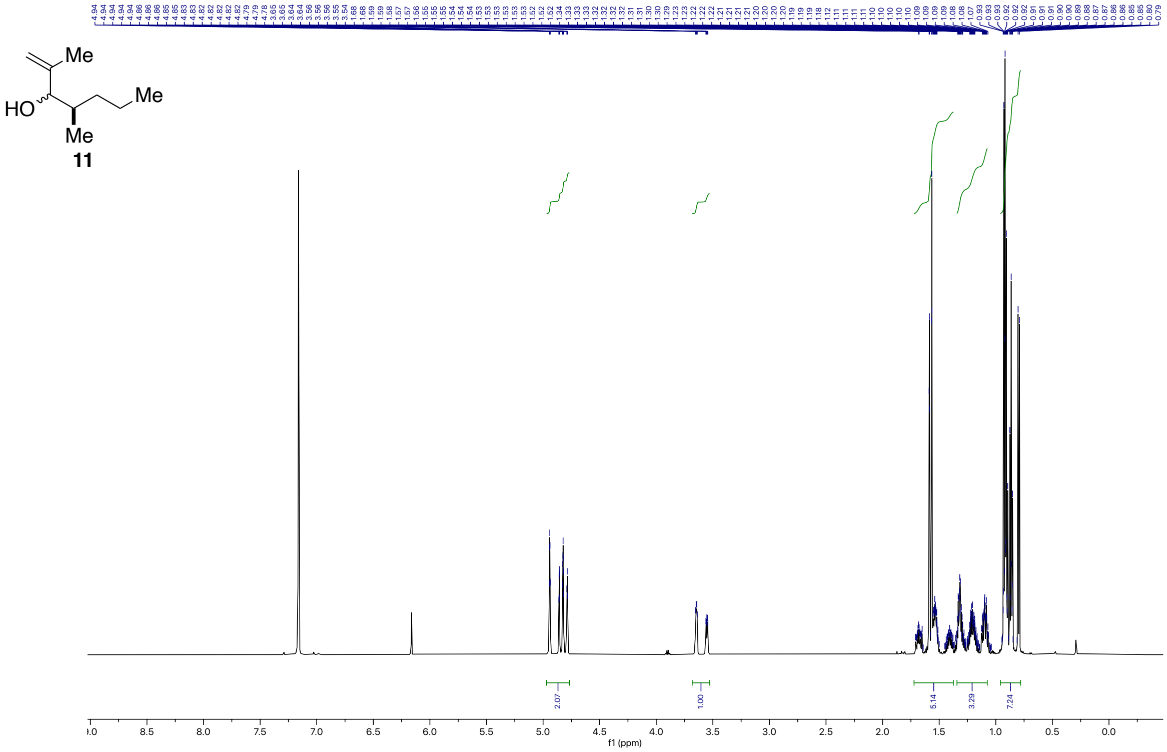

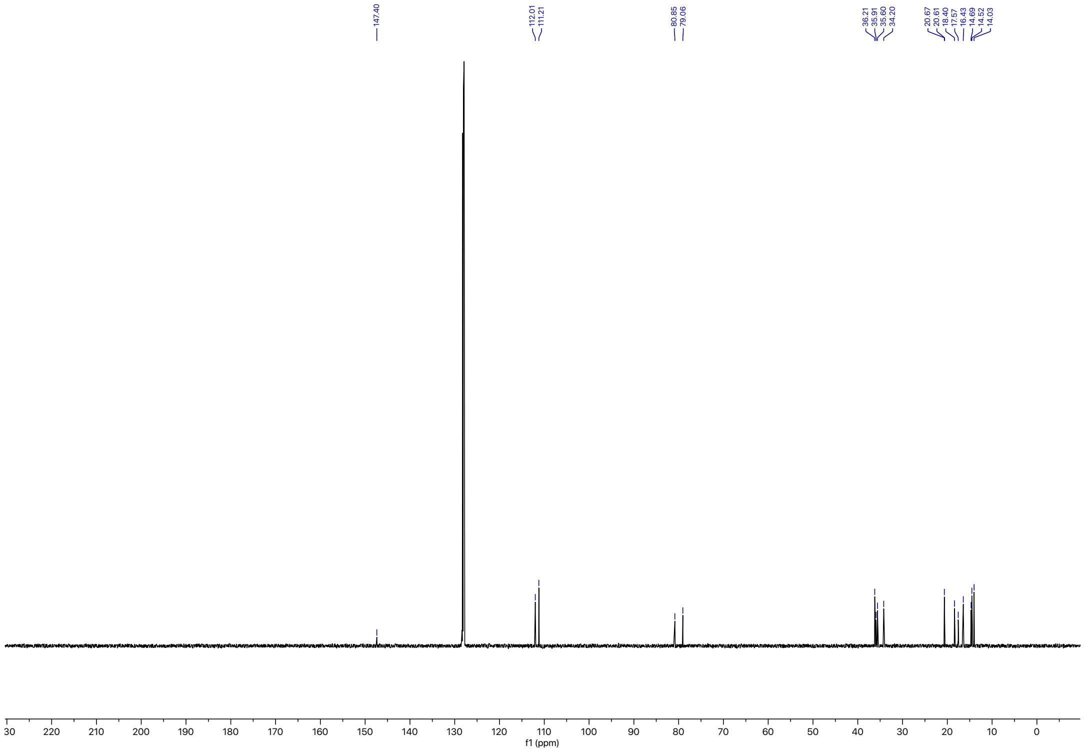

^{1}H NMR (600 MHz, $CDCl_3$) and ^{13}C NMR (150 MHz, $CDCl_3$) of **13**

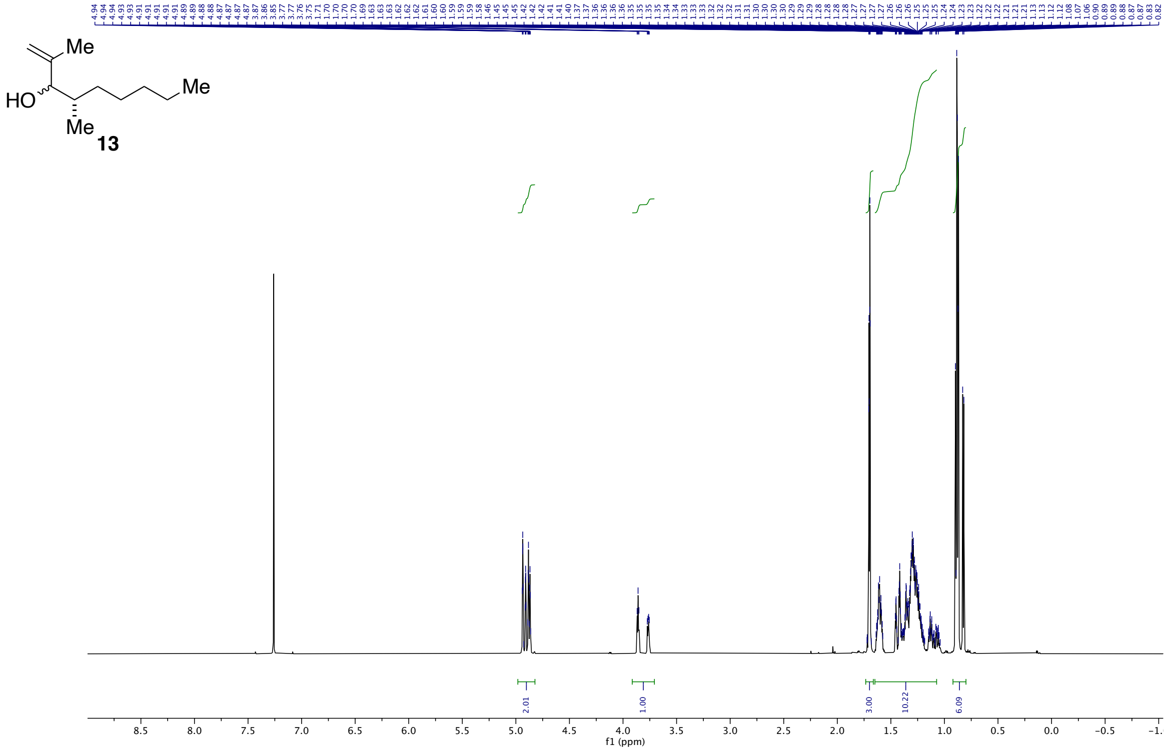

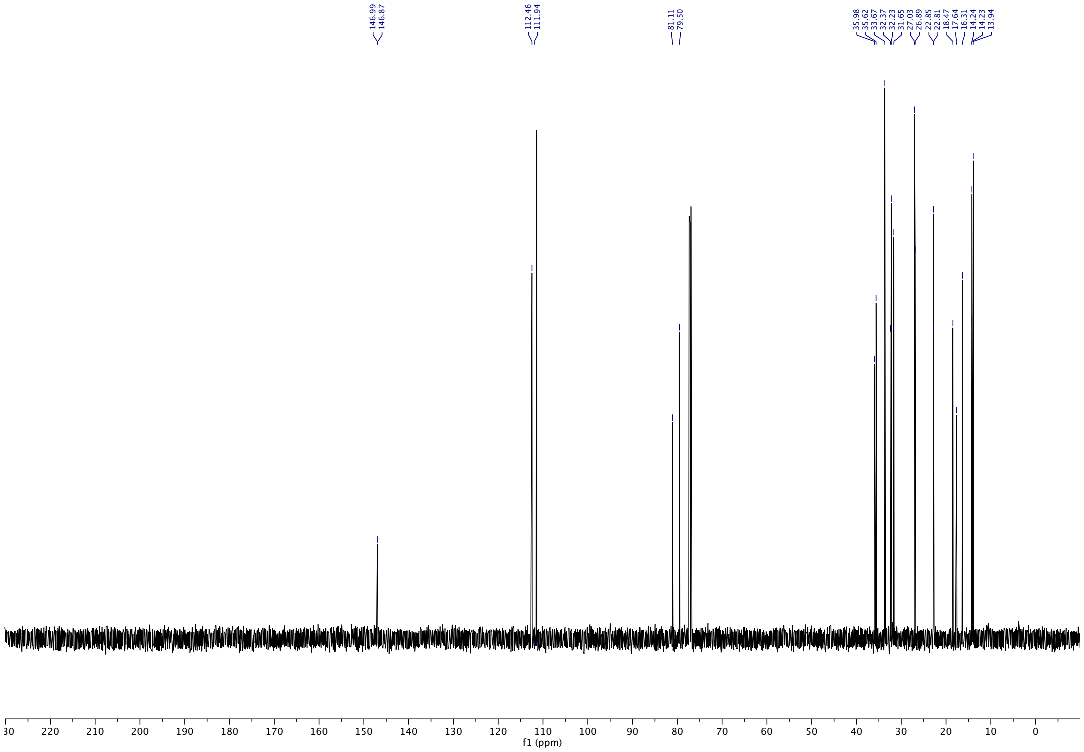

^{1}H NMR (600 MHz, $CDCl_3$) and ^{13}C NMR (150 MHz, $CDCl_3$) of **14**

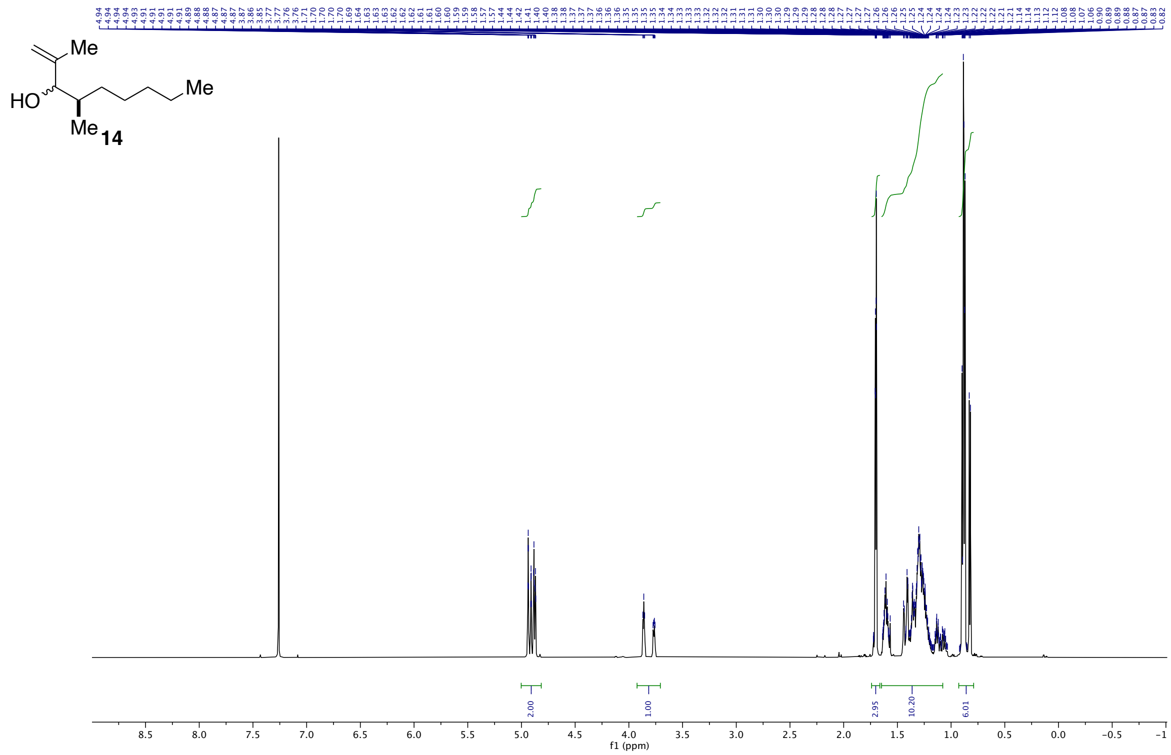

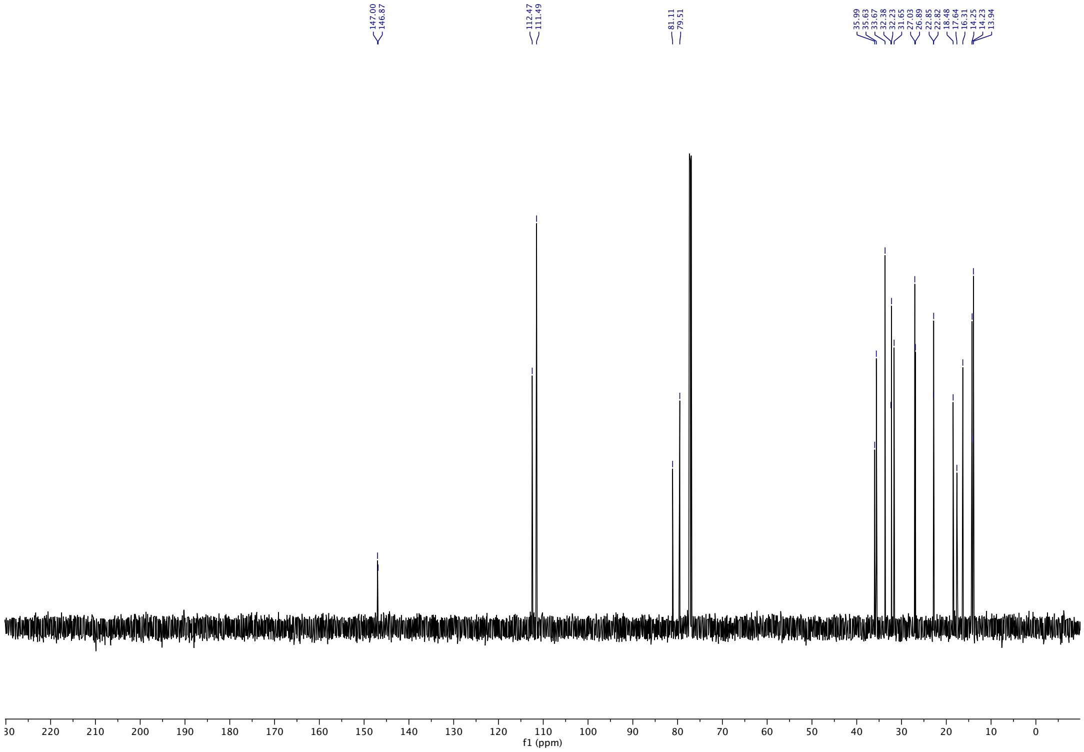

^{1}H NMR (600 MHz, $CDCl_3$) and ^{13}C NMR (150 MHz, $CDCl_3$) of **16** (Diastereomer ^{13}C signal at 22.8 and 14.3 ppm not selectable via NMR software)

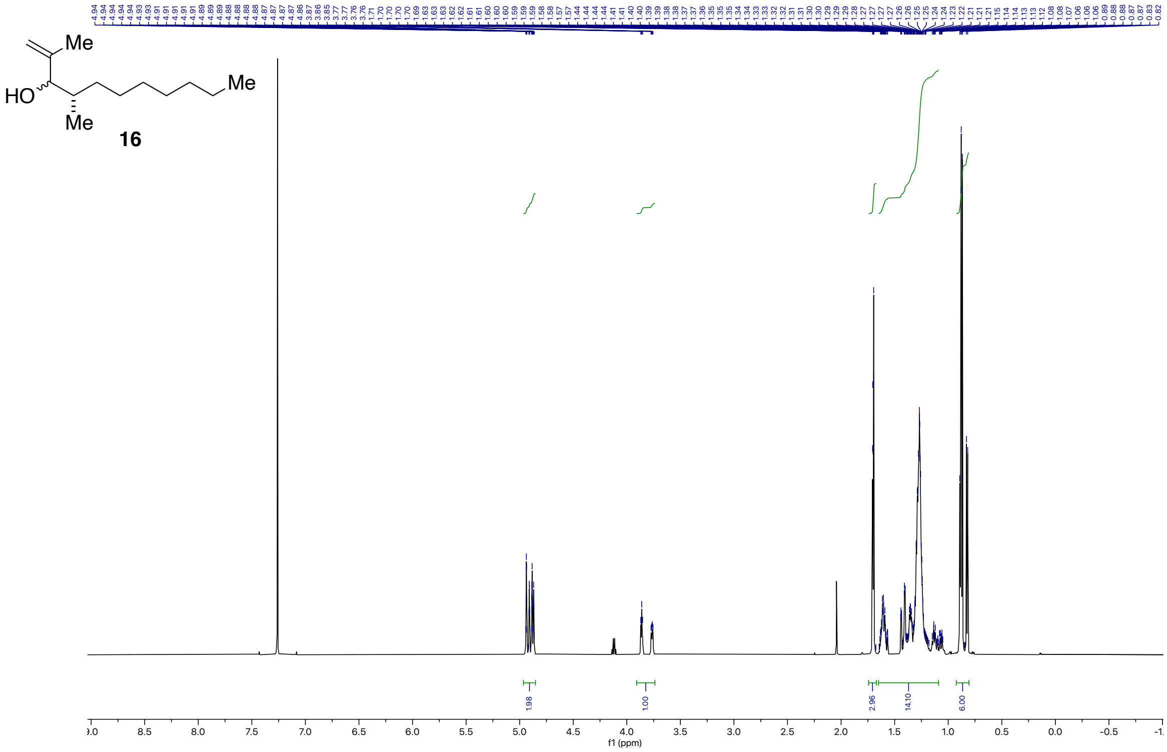

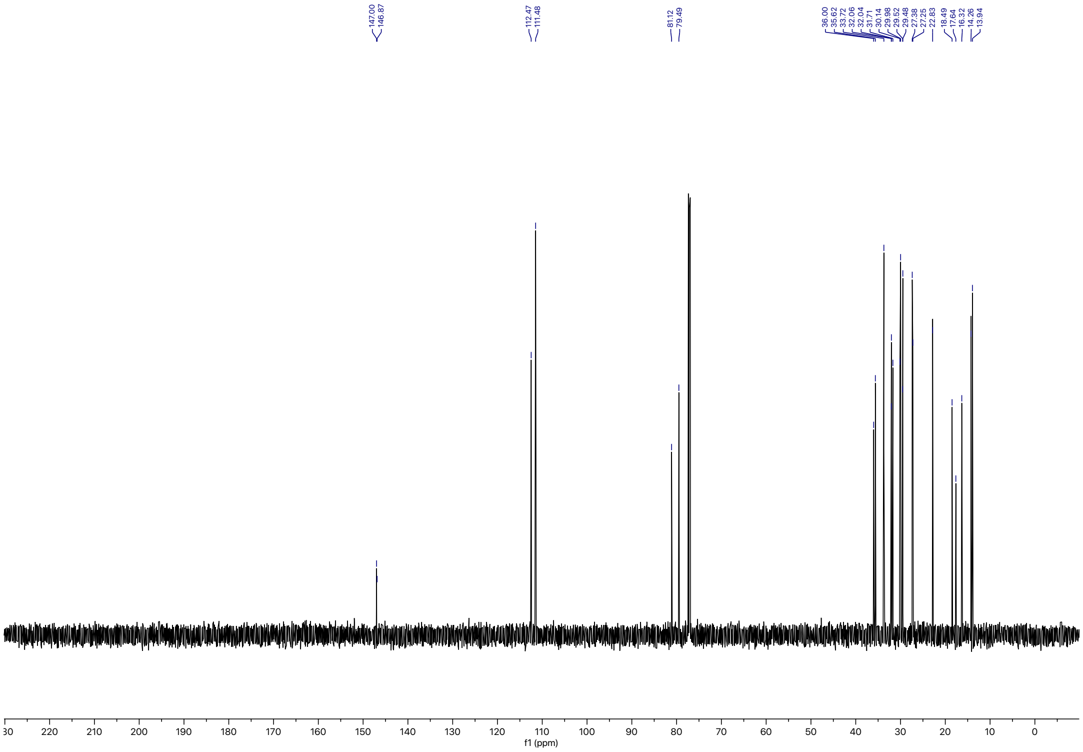

^{1}H NMR (600 MHz, $CDCl_3$) and ^{13}C NMR (150 MHz, $CDCl_3$) of **17** (Diastereomer ^{13}C signal at 22.8 and 14.3 ppm not selectable via NMR software)

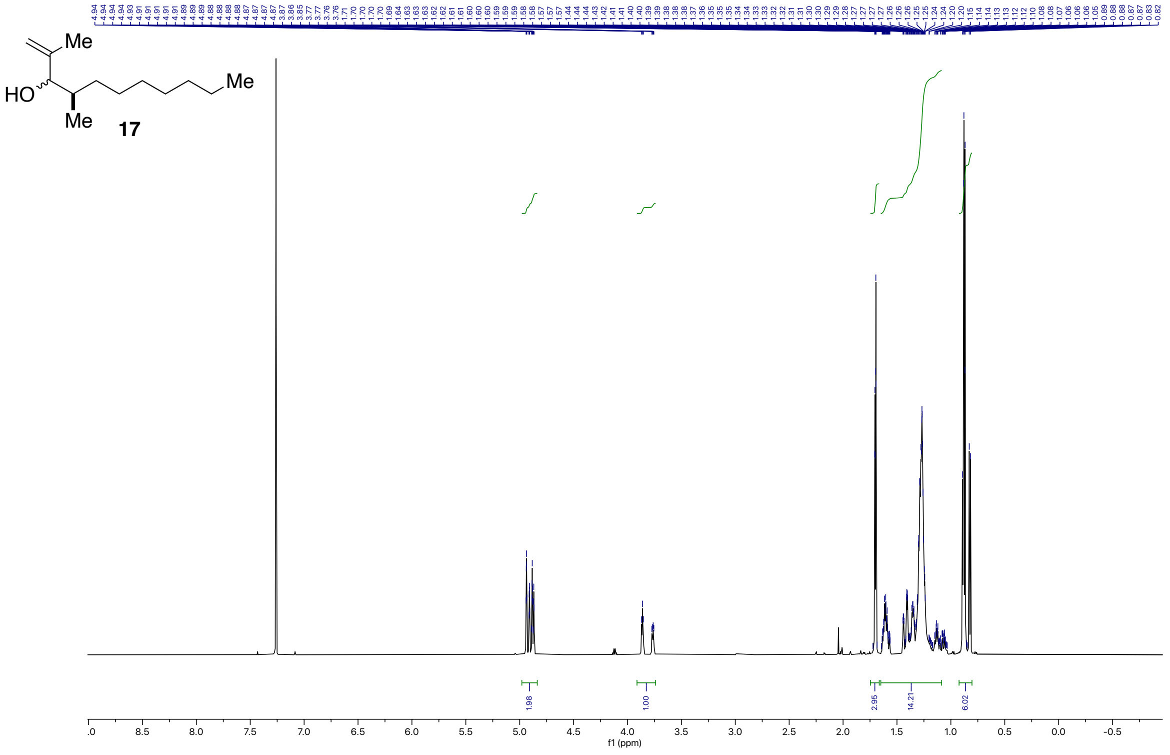

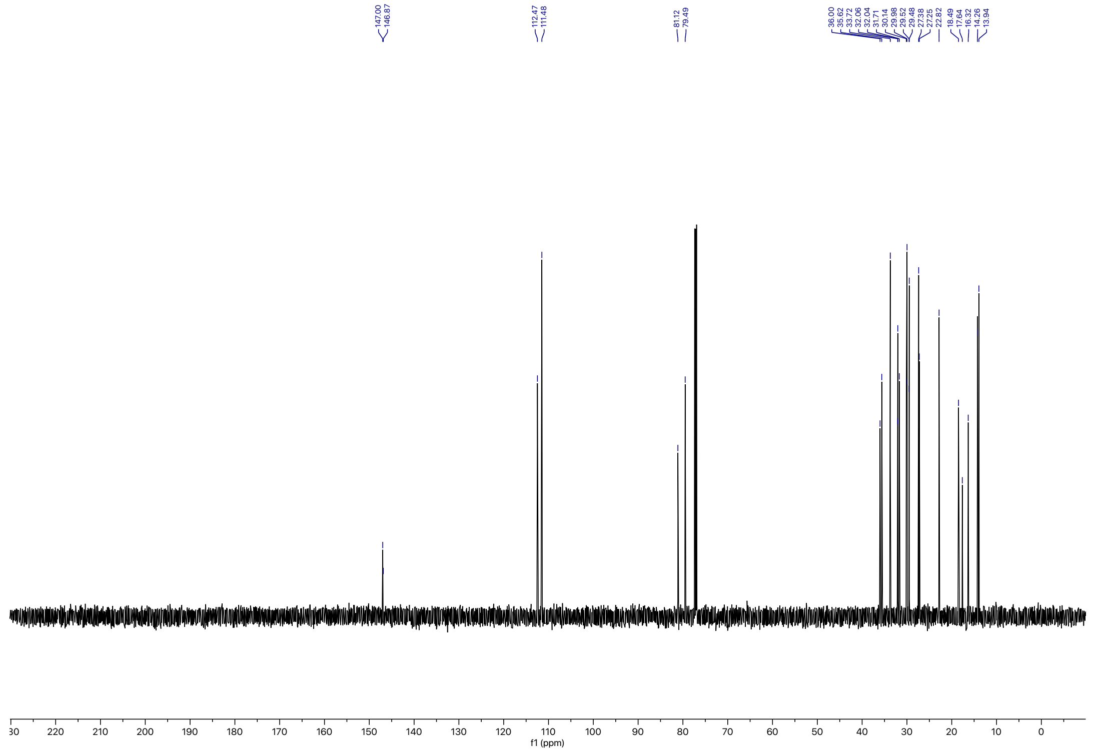

^1H NMR (600 MHz, $CDCl_3$) and ^{13}C NMR (150 MHz, $CDCl_3$) of **19** (Diastereomer ^{13}C signal at 32.1, 30.0, 29.5, and 14.3 ppm not selectable via NMR software)

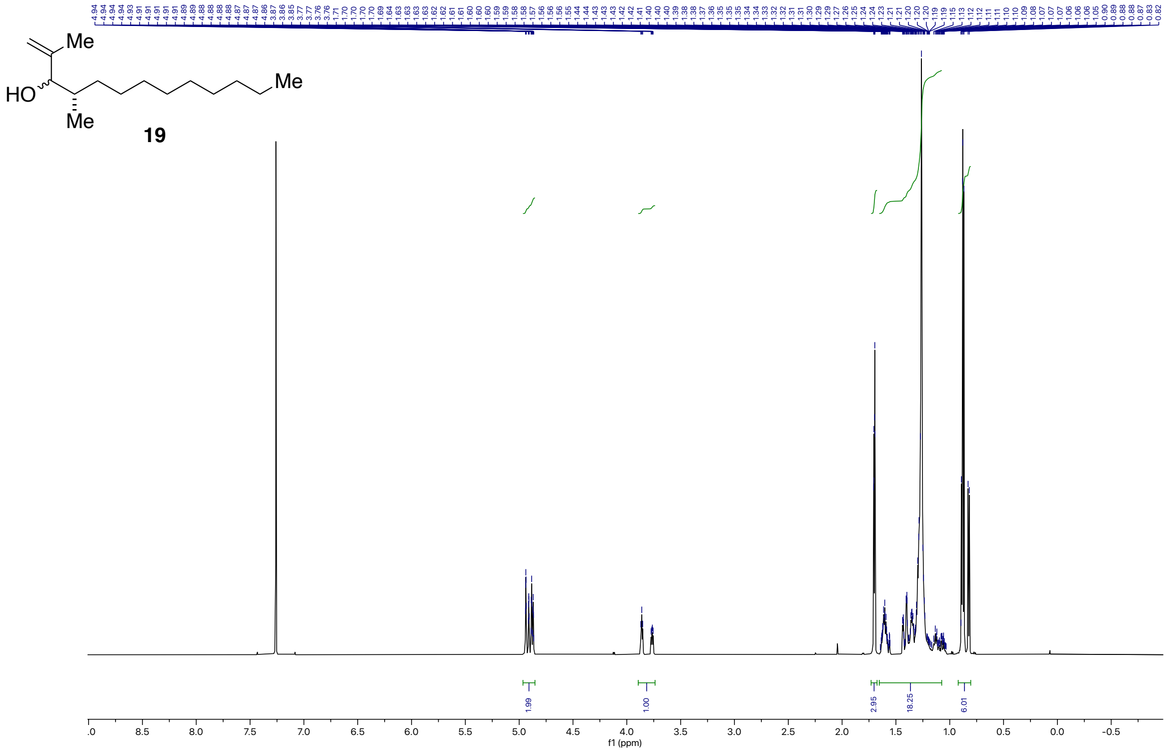

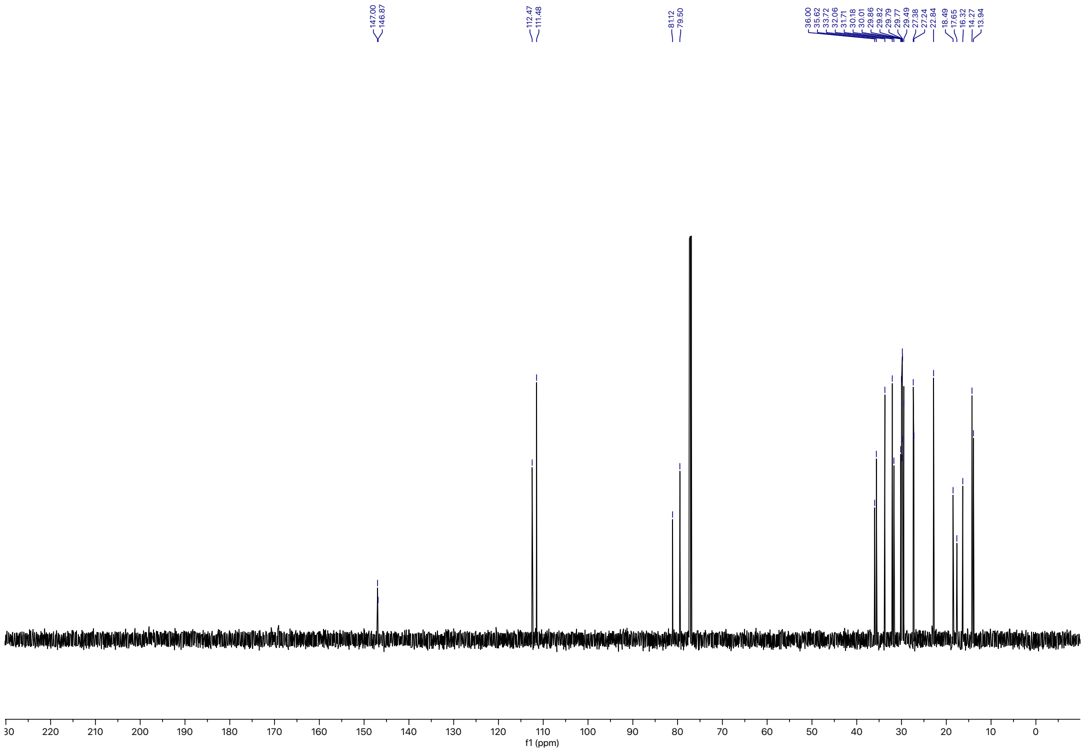

^{1}H NMR (600 MHz, $CDCl_3$) and ^{13}C NMR (150 MHz, $CDCl_3$) of **20** (Diastereomer ^{13}C signal at 32.1, 30.0, 29.5, and 14.3 ppm not selectable via NMR software)

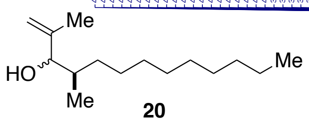

20

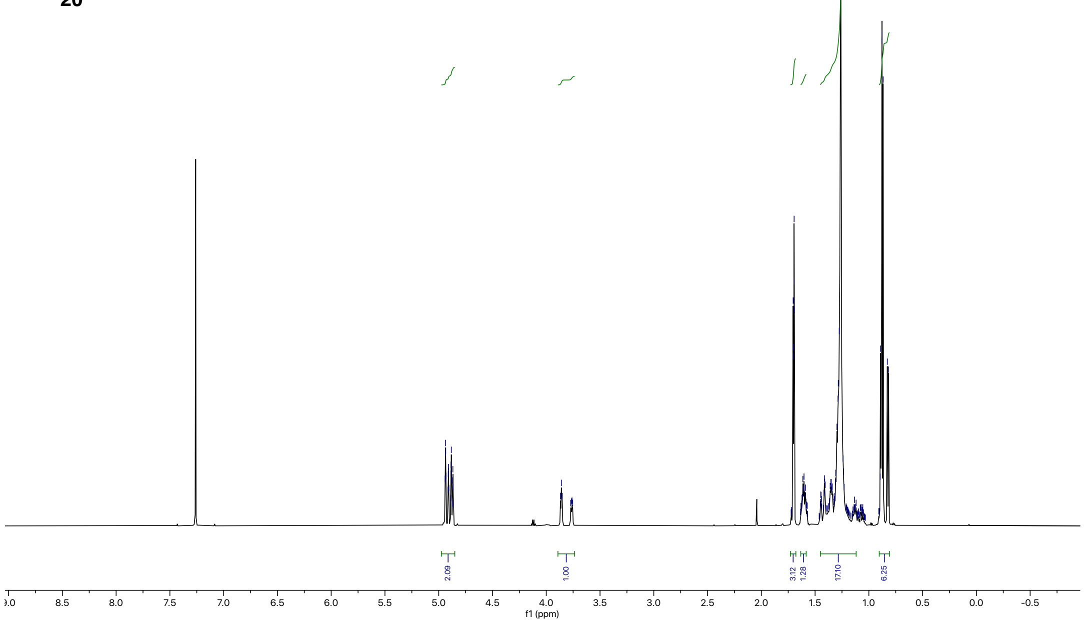

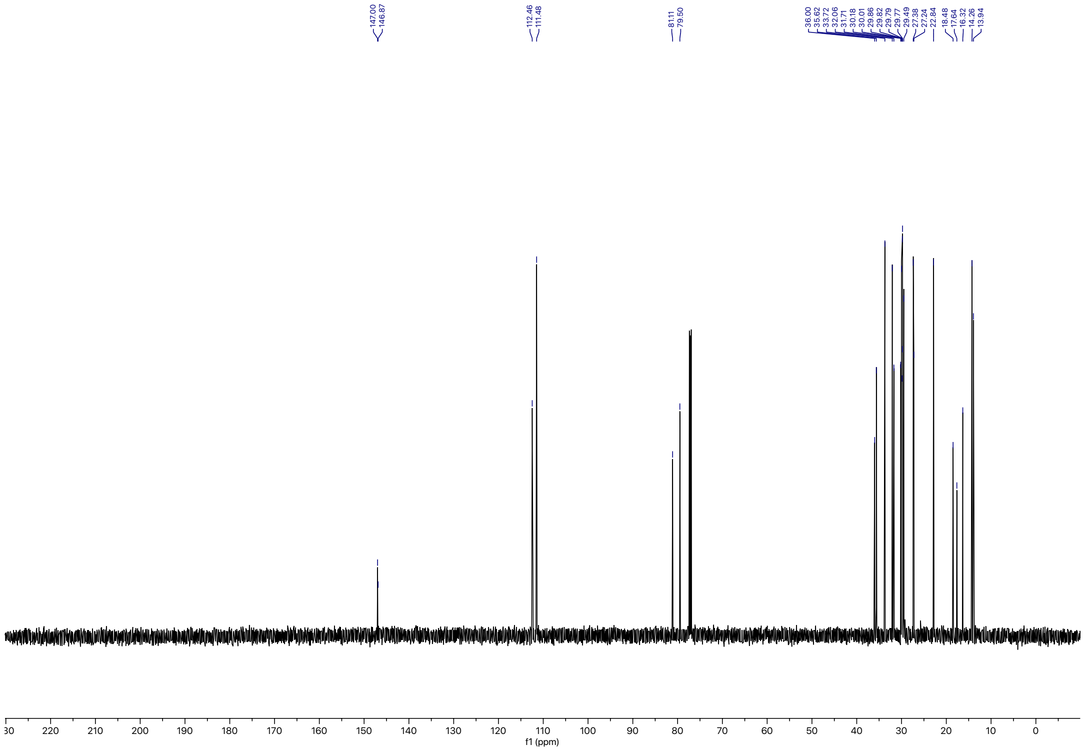

^{1}H NMR (600 MHz, $CDCl_3$) and ^{13}C NMR (150 MHz, $CDCl_3$) of **S1**

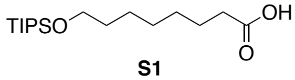

S1

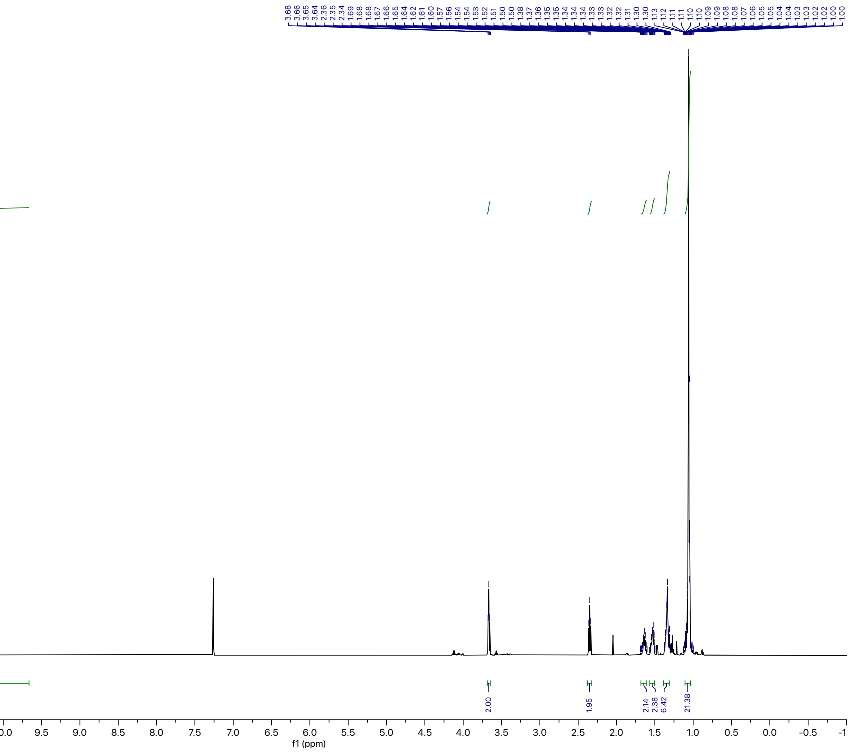

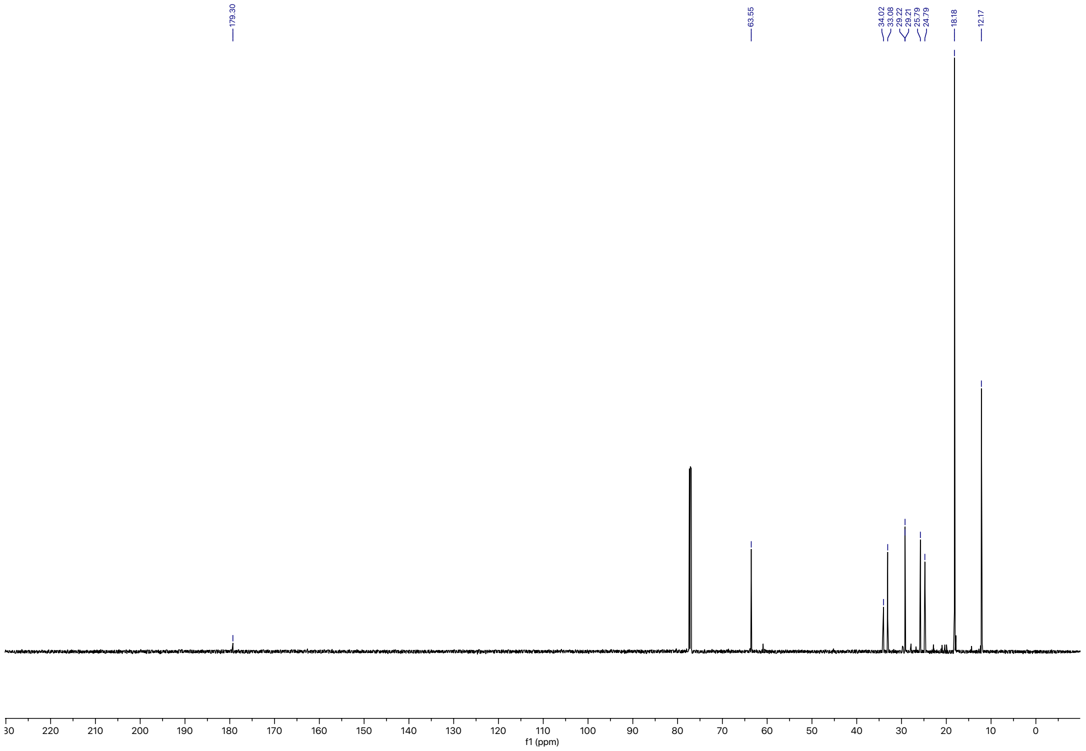

^1H NMR (600 MHz, $CDCl_3$) and ^{13}C NMR (150 MHz, $CDCl_3$) of **S2**

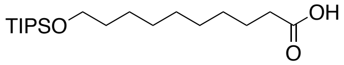

S2

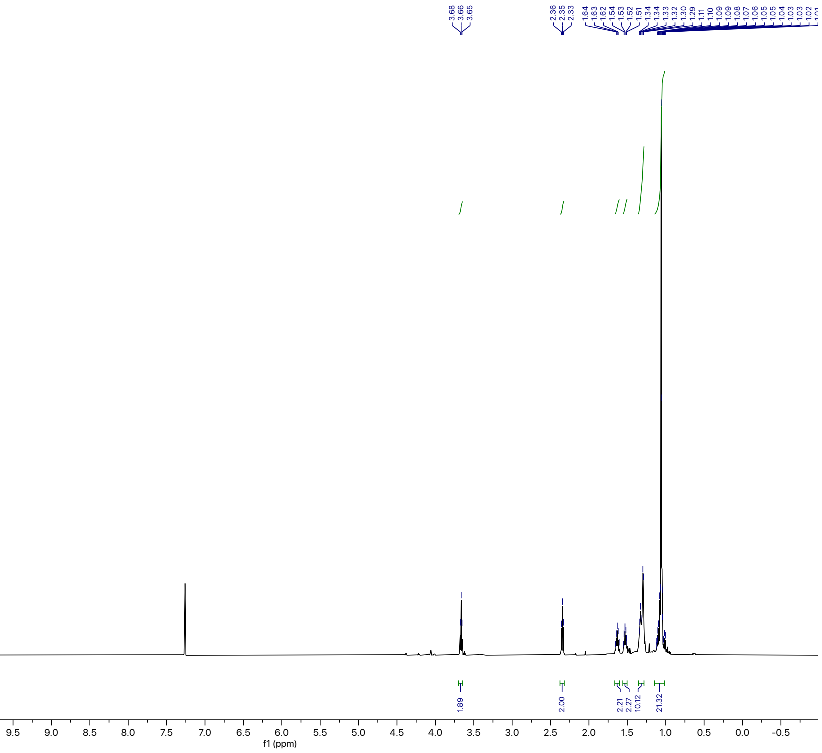

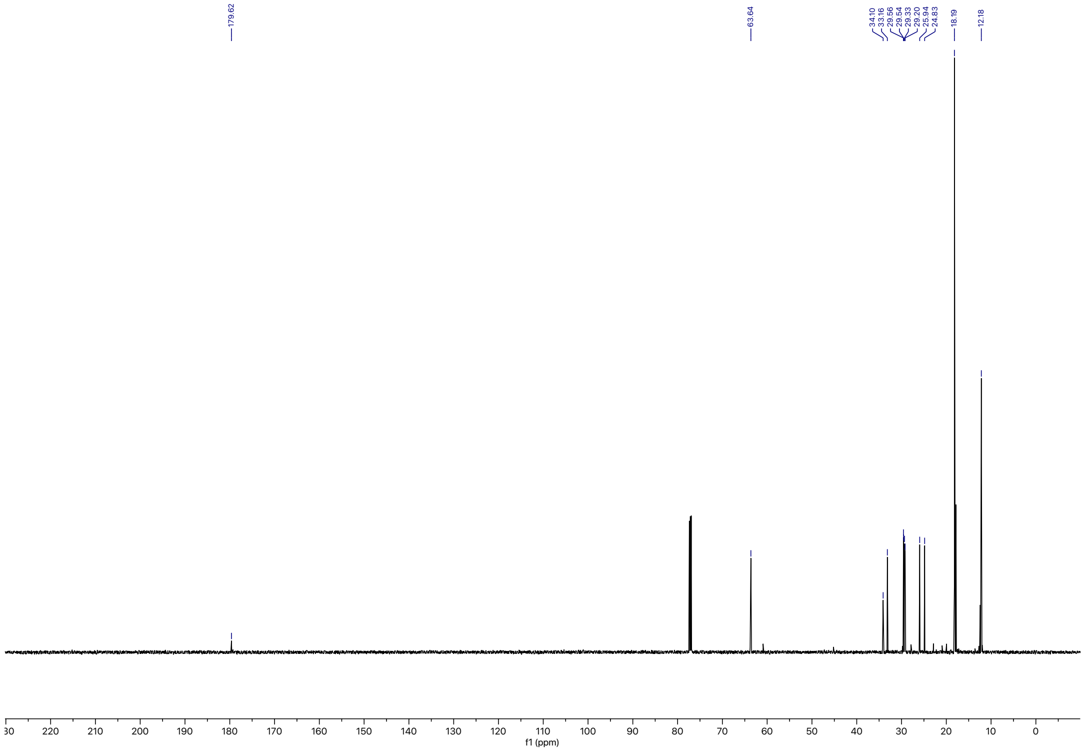

^{1}H NMR (600 MHz, $CDCl_3$) and ^{13}C NMR (150 MHz, $CDCl_3$) of **S3**

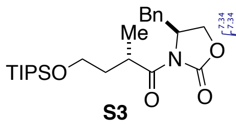

S3

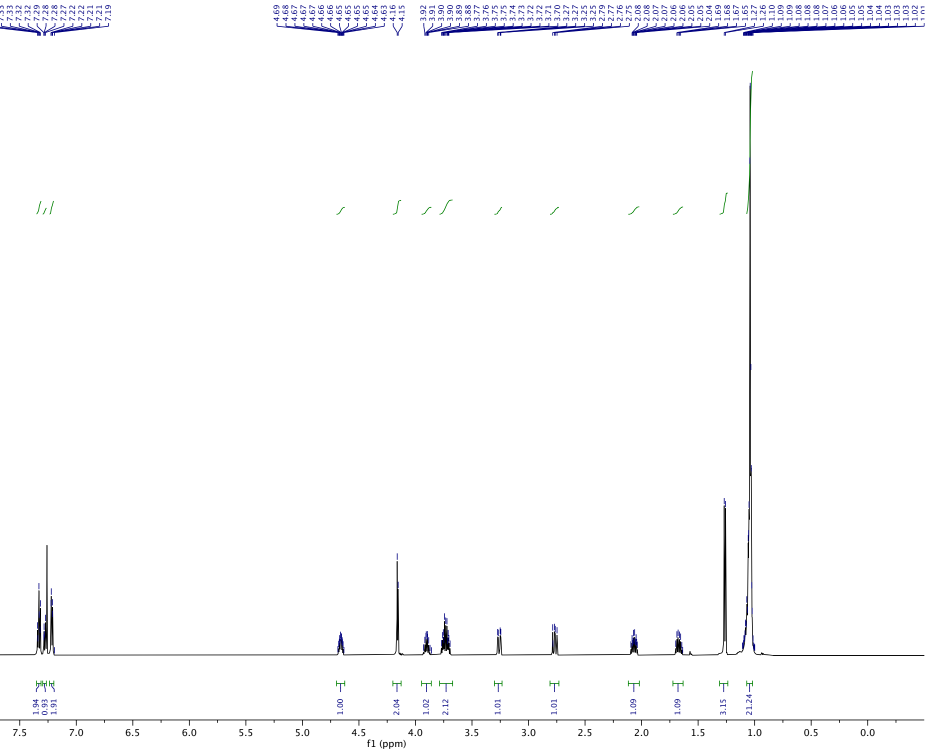

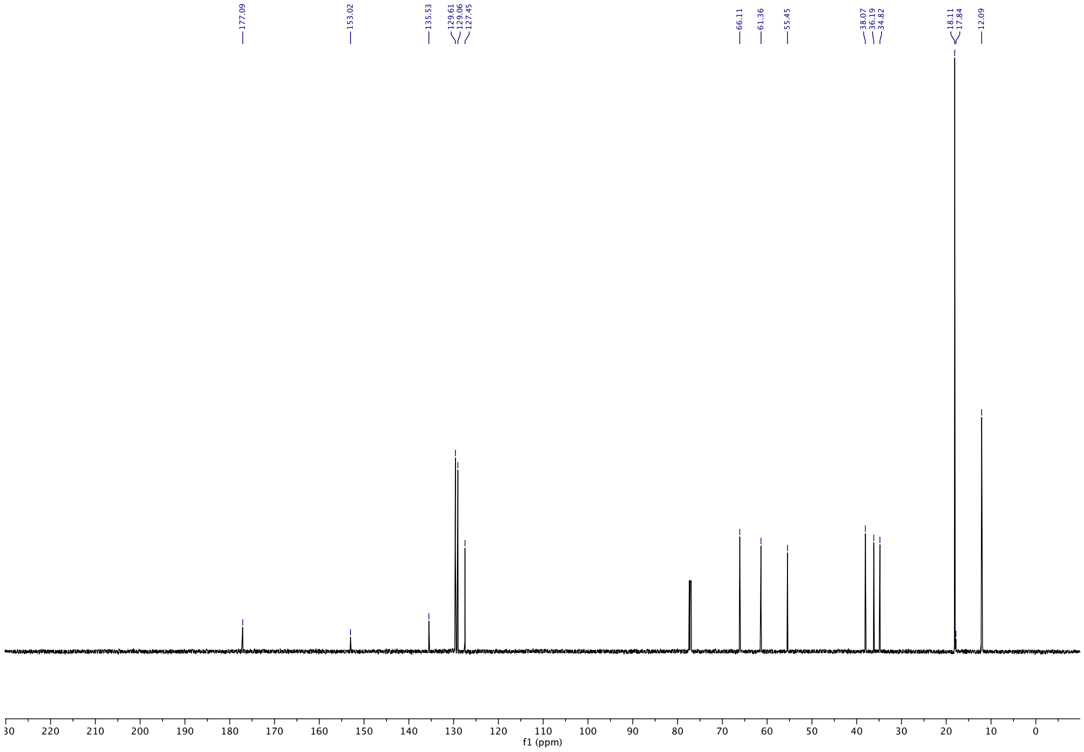

^{1}H NMR (600 MHz, $CDCl_3$) and ^{13}C NMR (150 MHz, $CDCl_3$) of **S4**

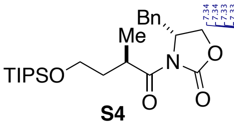

S4

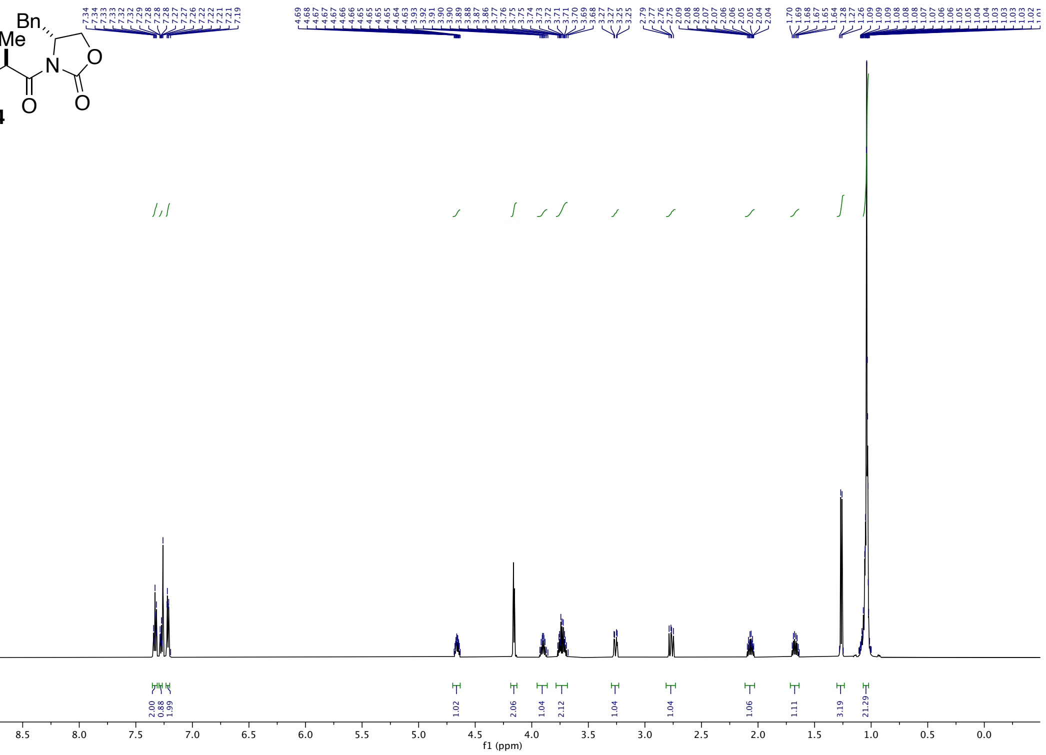

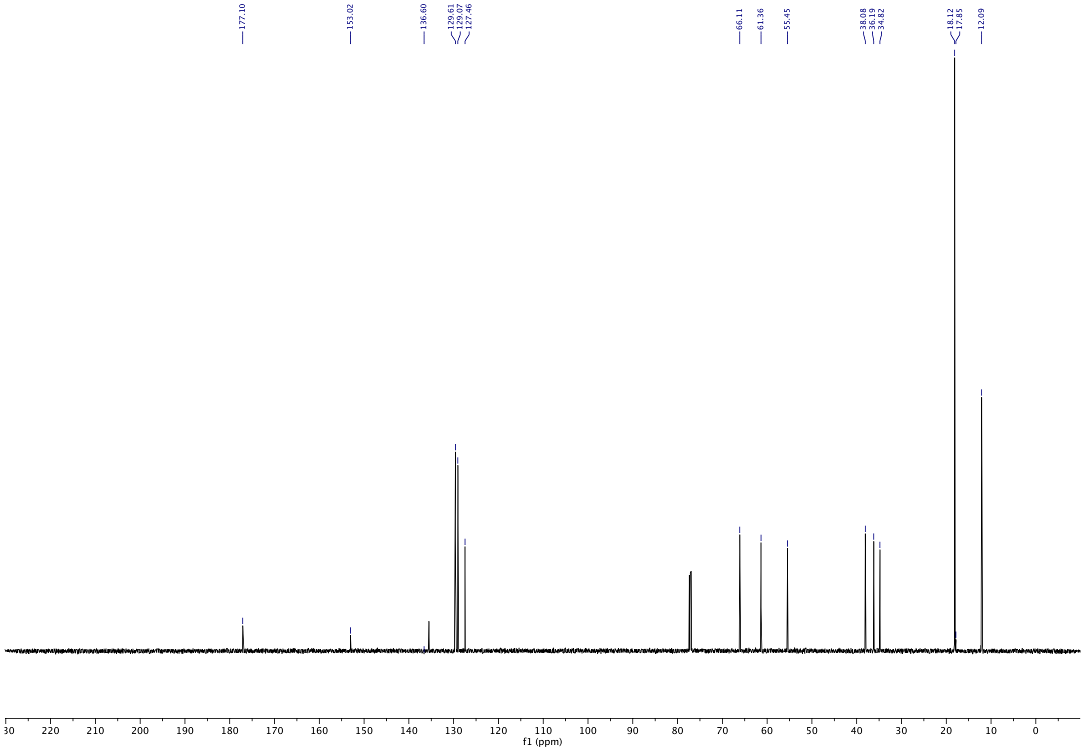

^{1}H NMR (600 MHz, $CDCl_3$) and ^{13}C NMR (150 MHz, $CDCl_3$) of **S5**

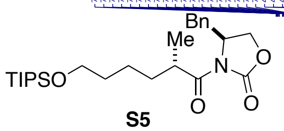

S5

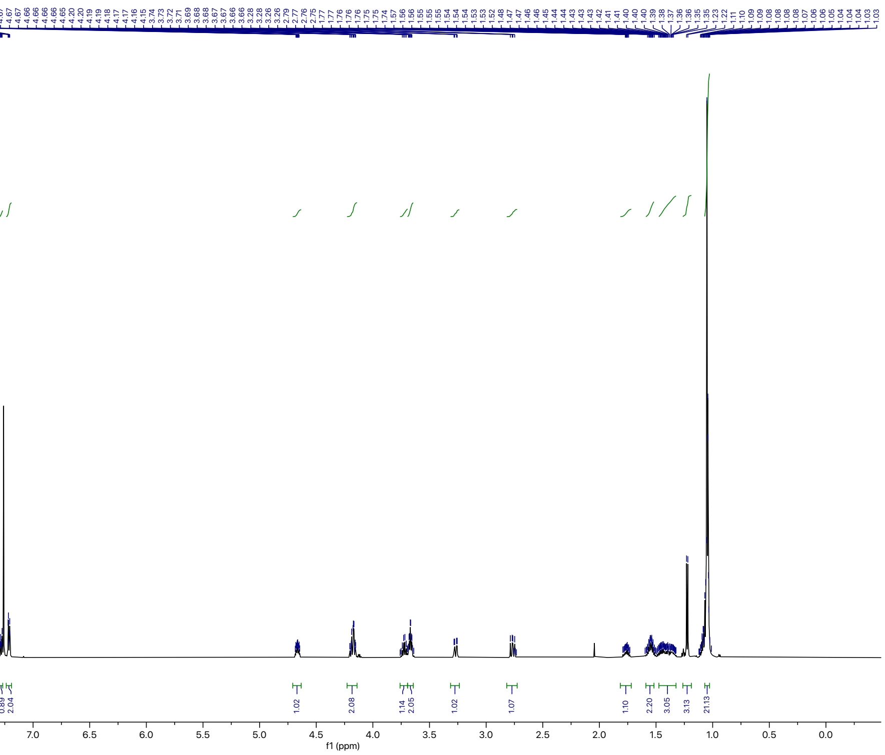

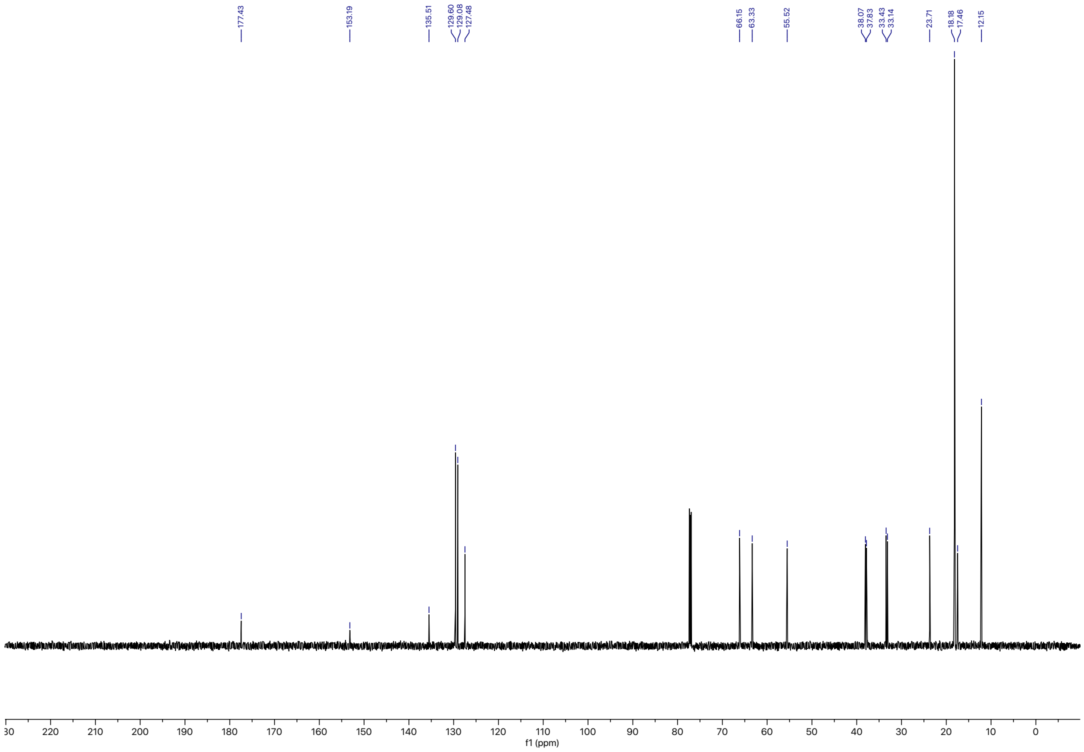

^1H NMR (600 MHz, $CDCl_3$) and ^{13}C NMR (150 MHz, $CDCl_3$) of **S6**

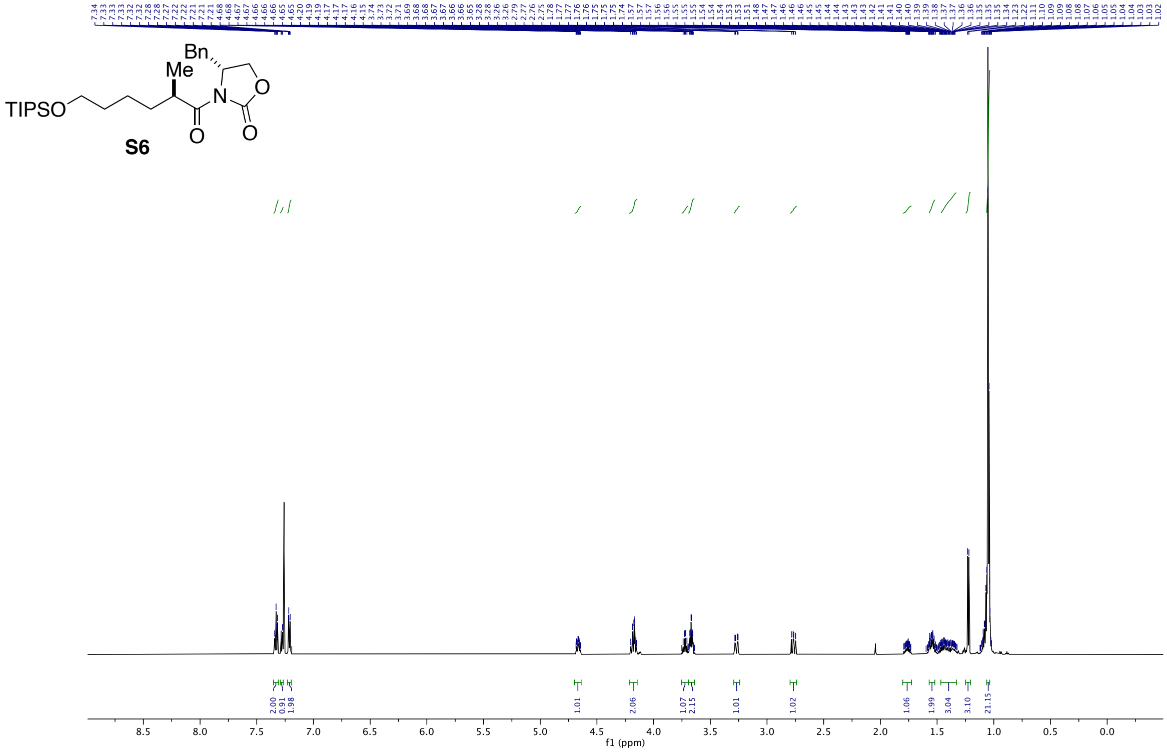

S6

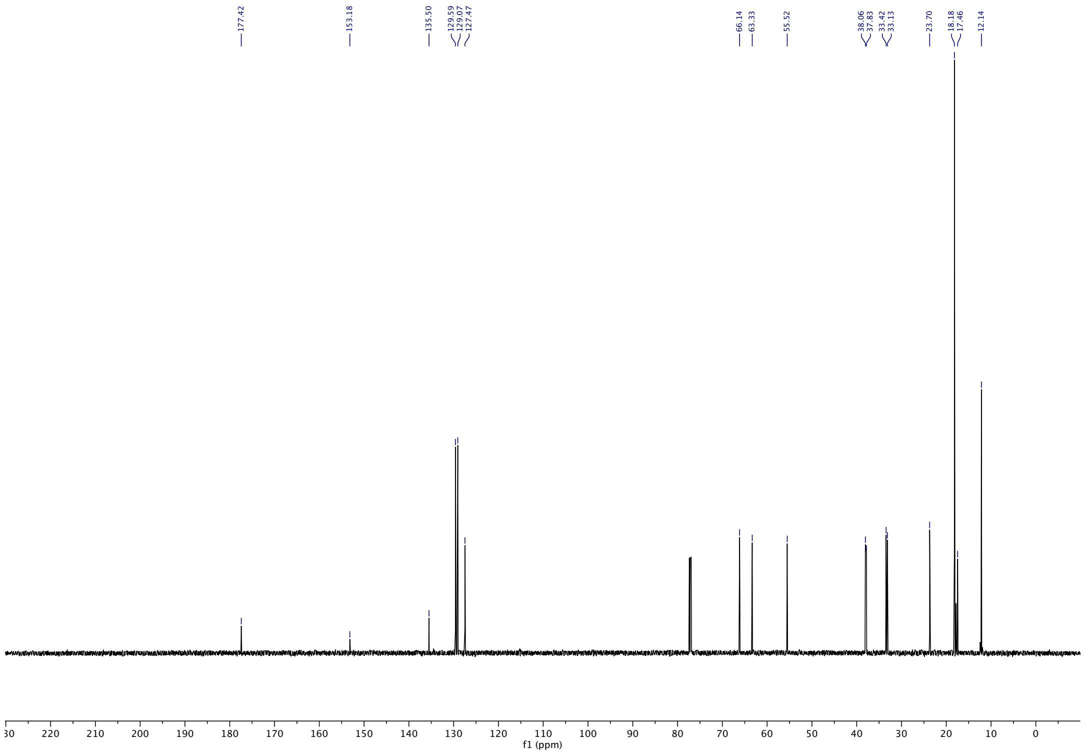

^{1}H NMR (600 MHz, $CDCl_3$) and ^{13}C NMR (150 MHz, $CDCl_3$) of **S7**

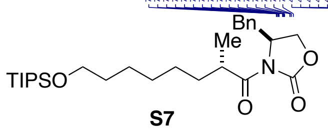

S7

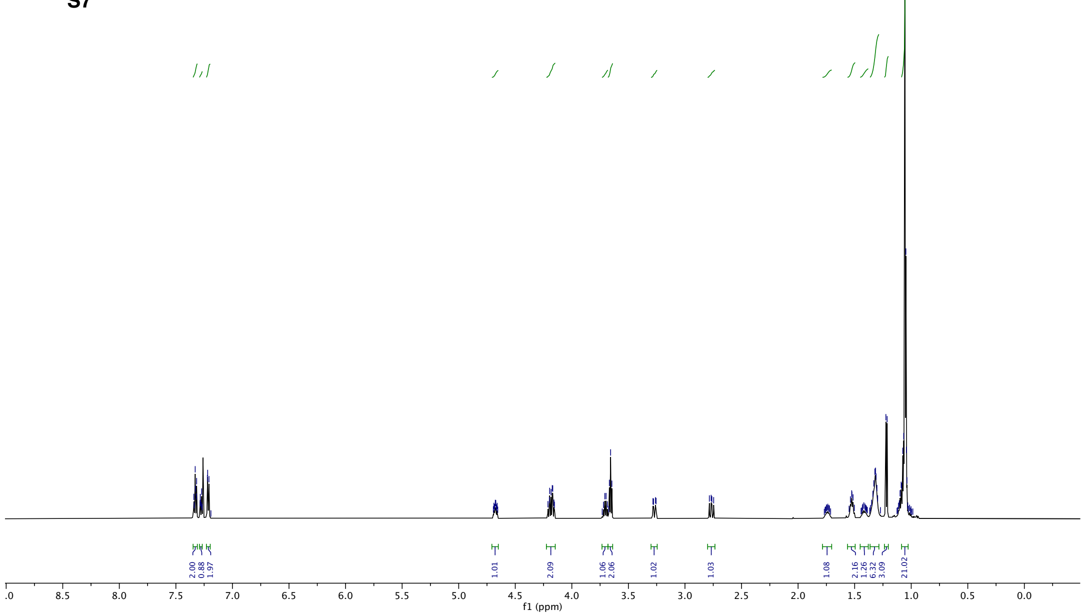

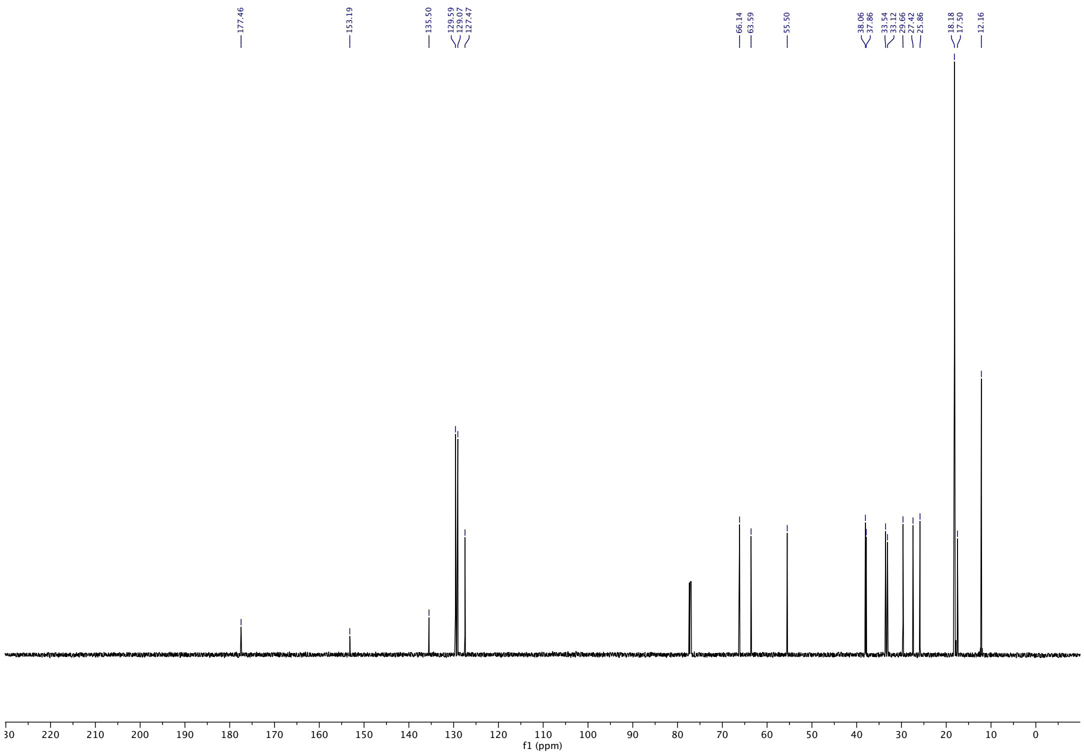

^{1}H NMR (600 MHz, $CDCl_3$) and ^{13}C NMR (150 MHz, $CDCl_3$) of **S8**

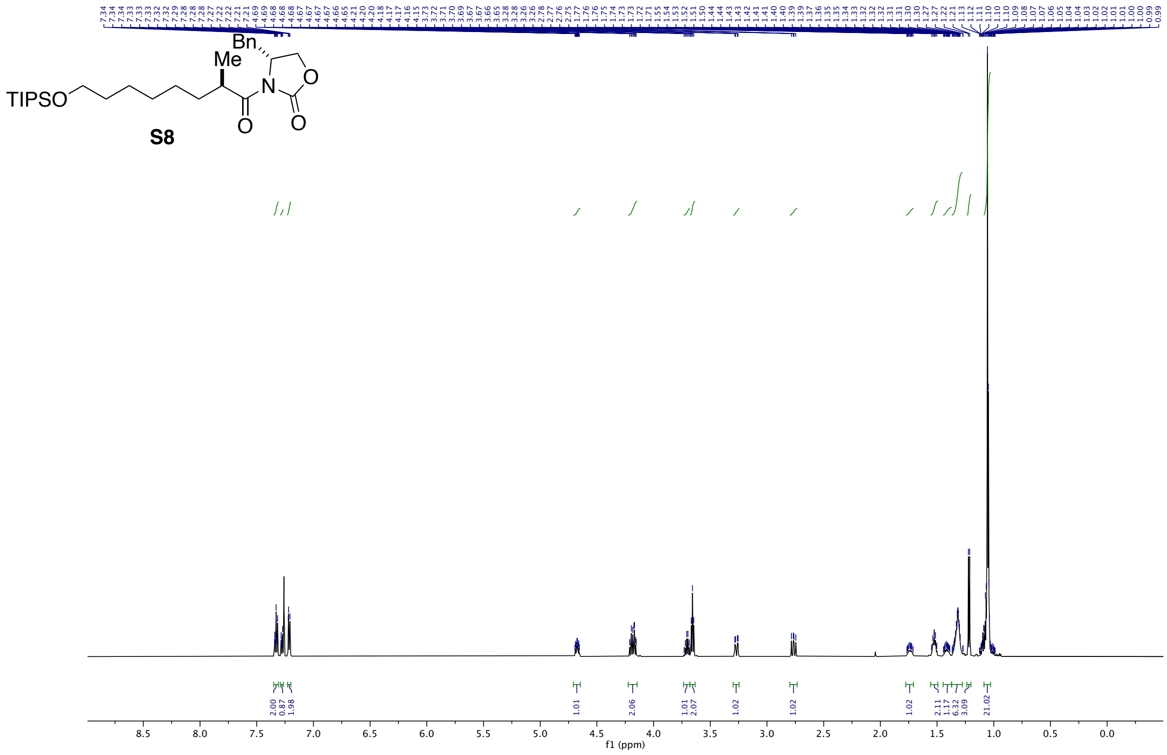

S8

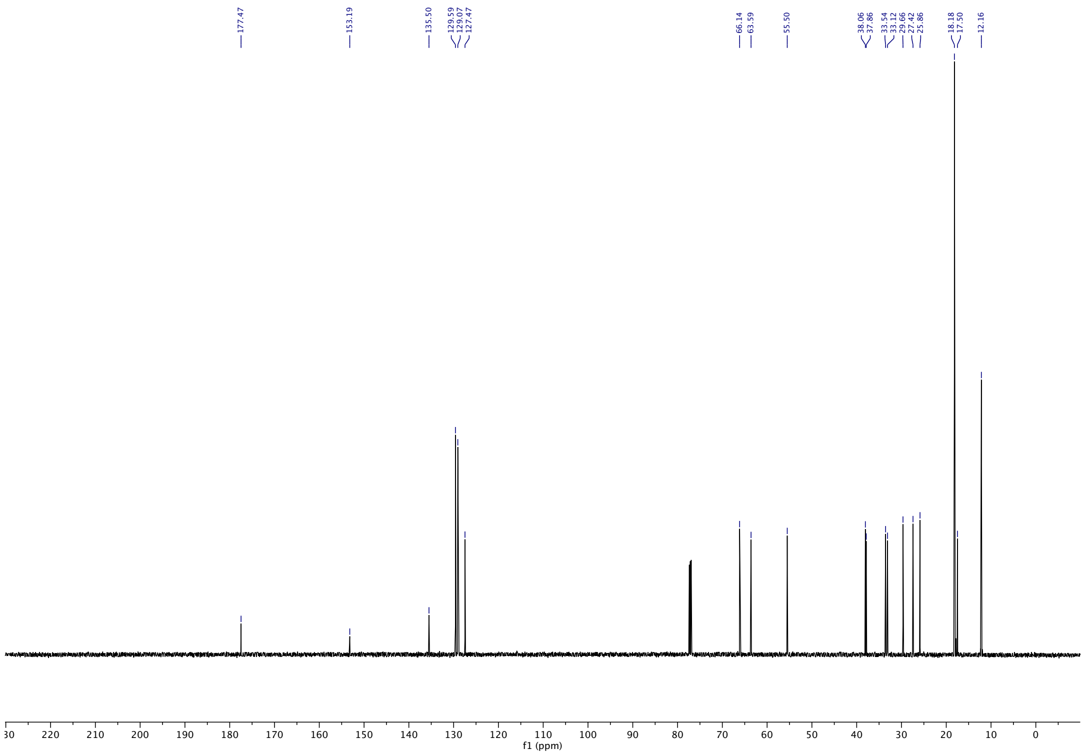

^{1}H NMR (600 MHz, $CDCl_3$) and ^{13}C NMR (150 MHz, $CDCl_3$) of **S9**

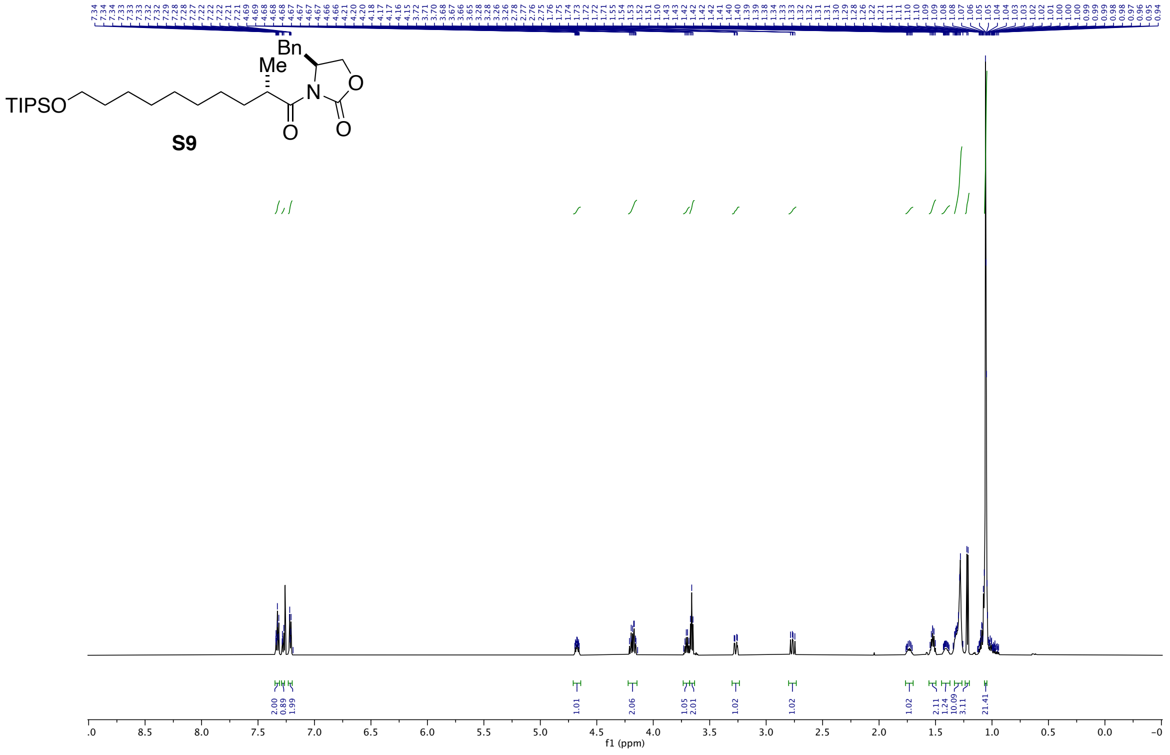

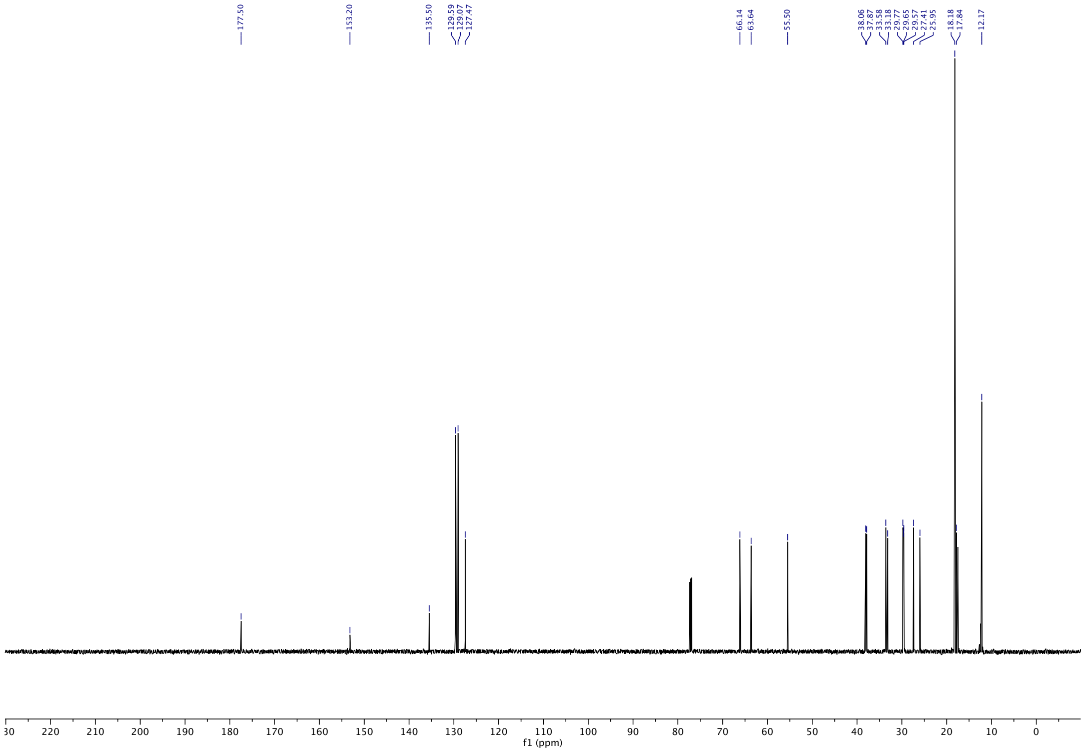

^{1}H NMR (600 MHz, $CDCl_3$) and ^{13}C NMR (150 MHz, $CDCl_3$) of **S10**

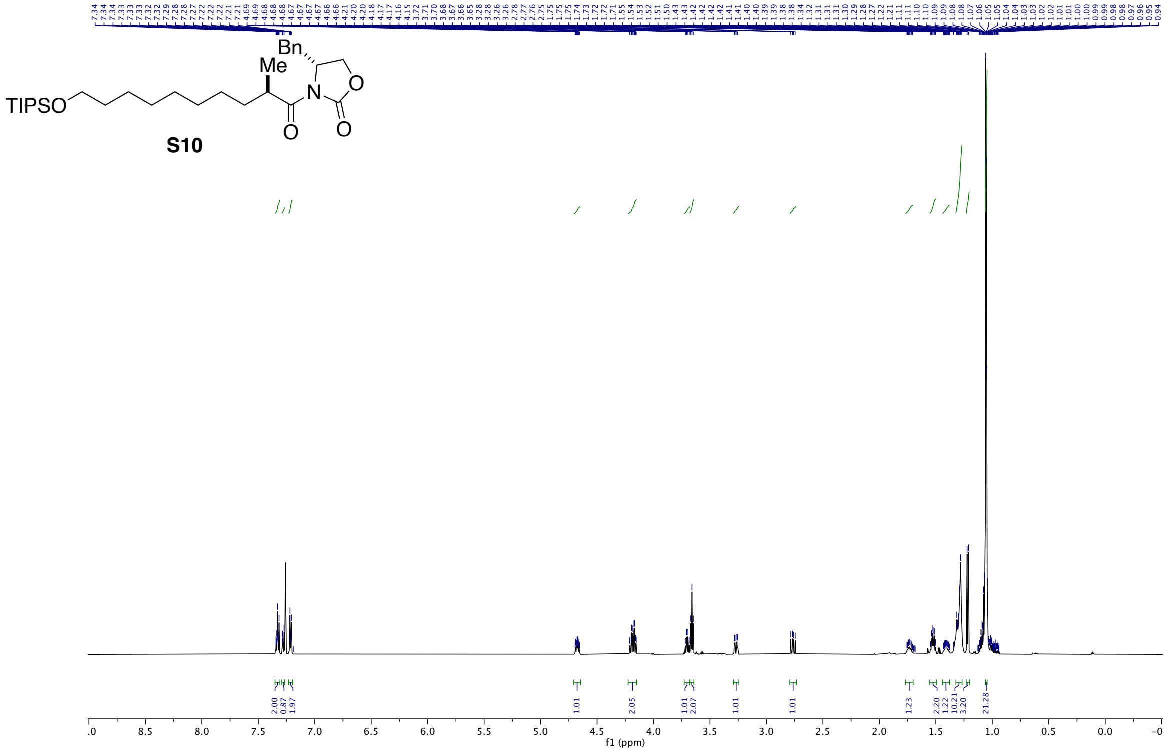

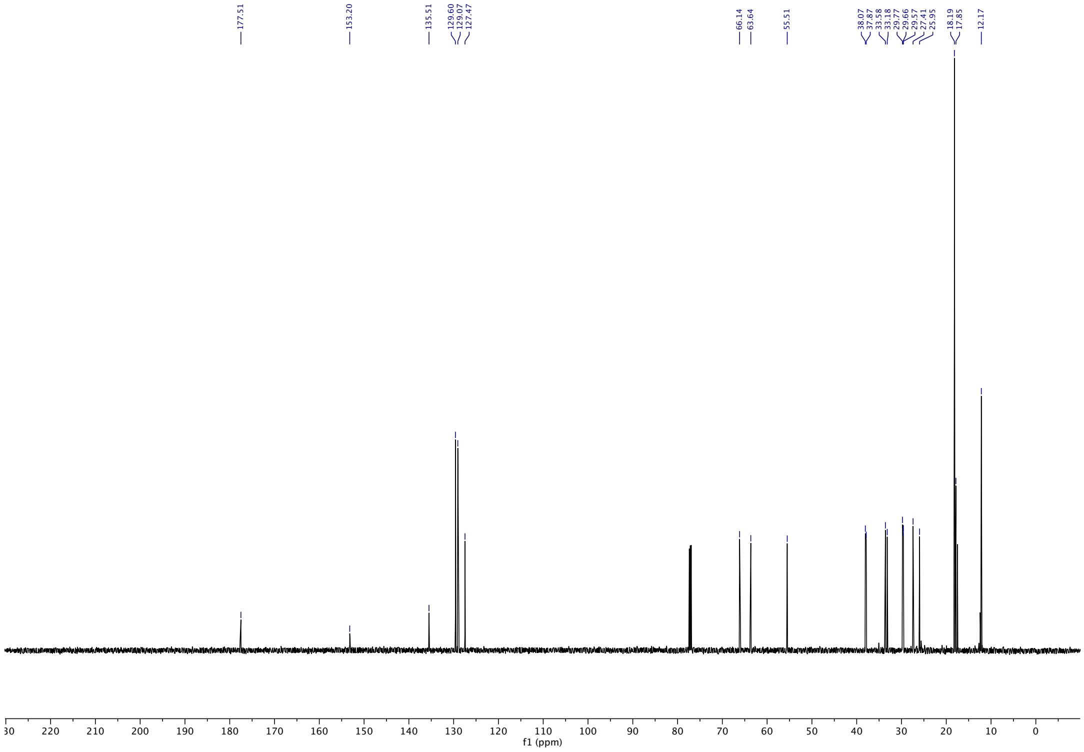

^{1}H NMR (600 MHz, $CDCl_3$) and ^{13}C NMR (150 MHz, $CDCl_3$) of **S11**

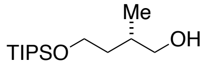

S11

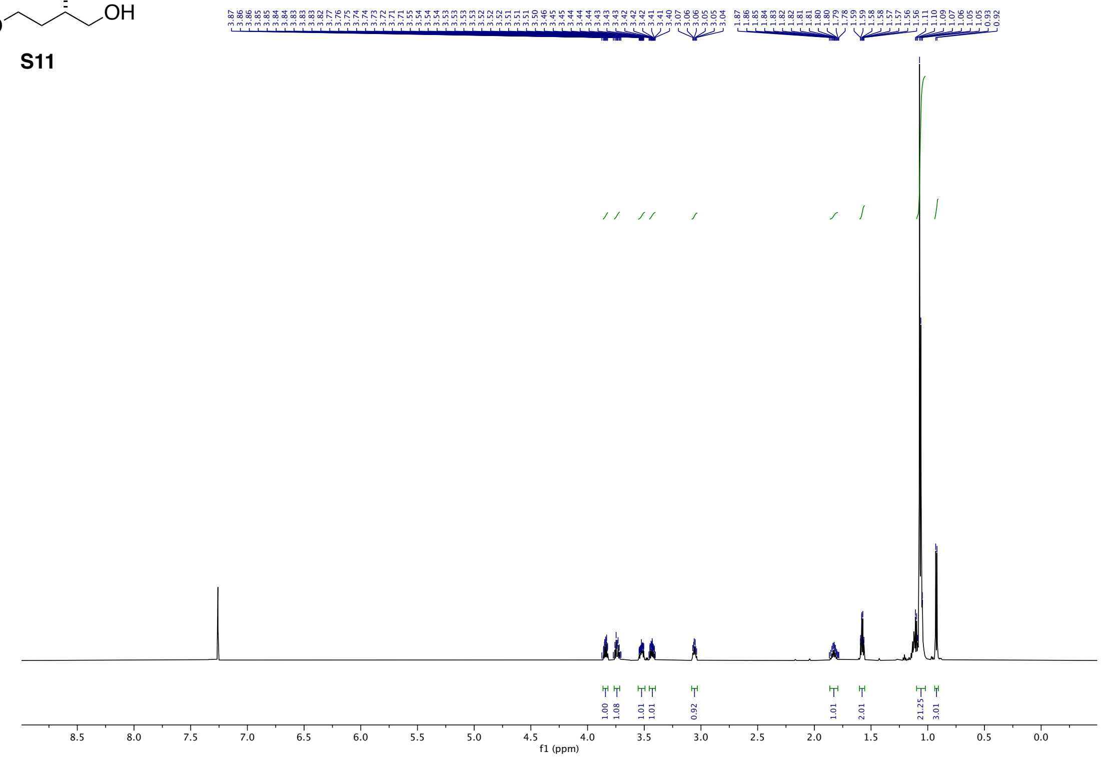

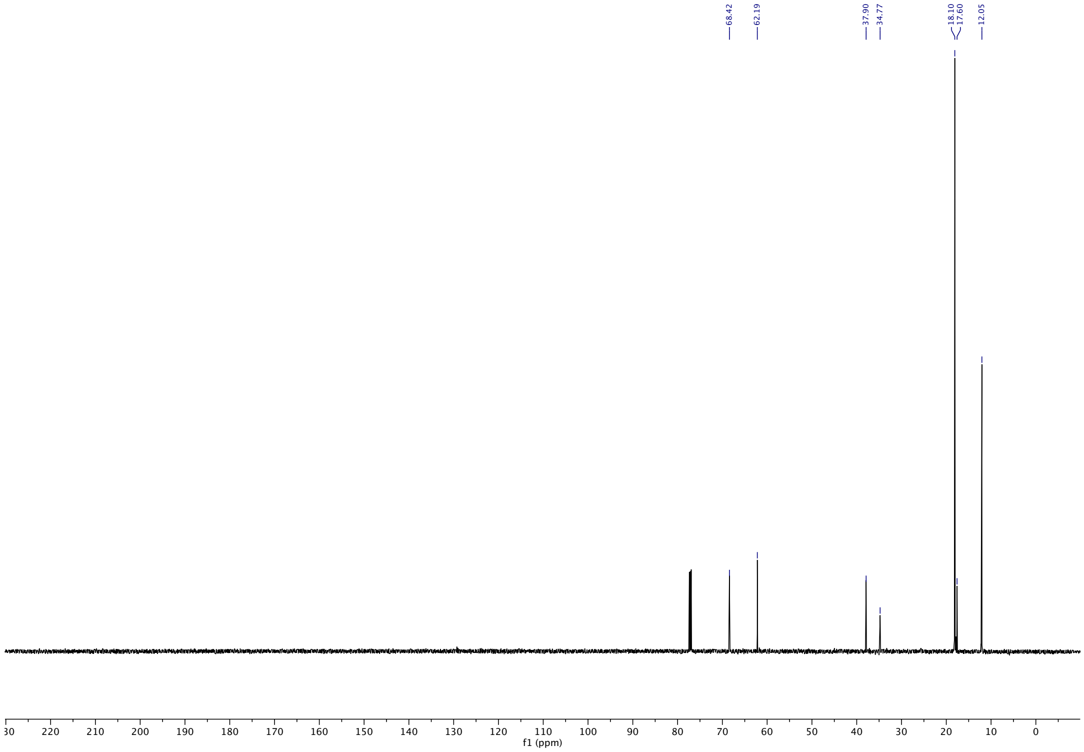

^{1}H NMR (600 MHz, $CDCl_3$) and ^{13}C NMR (150 MHz, $CDCl_3$) of **S12**

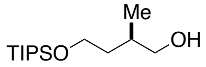

S12

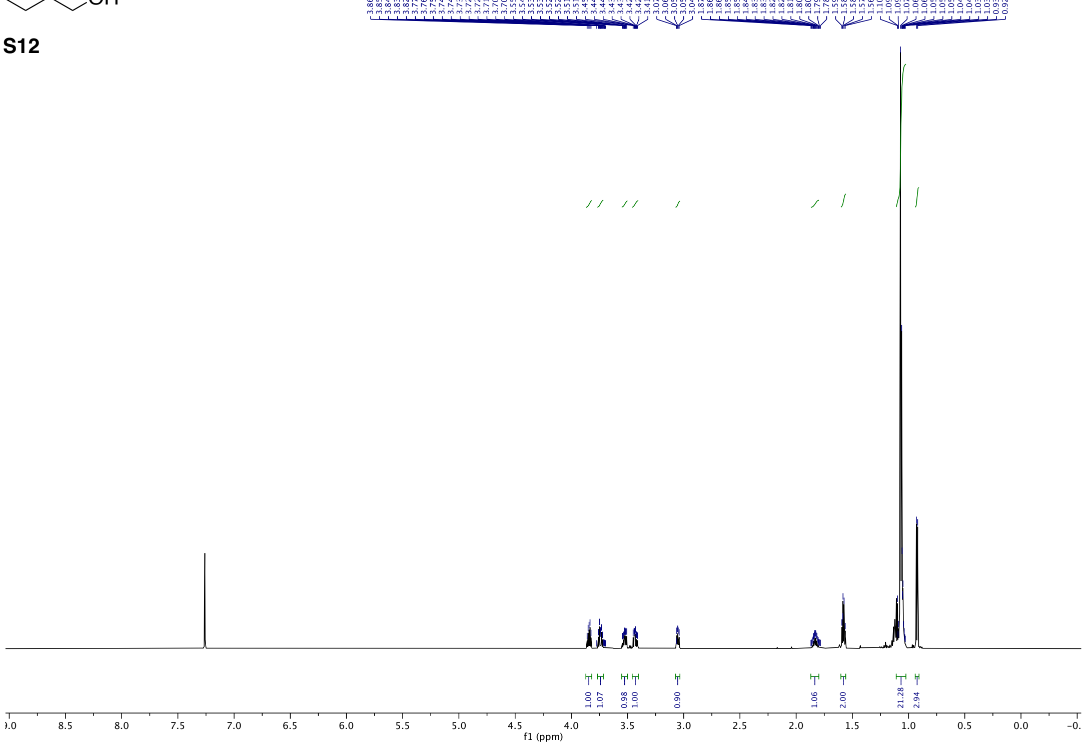

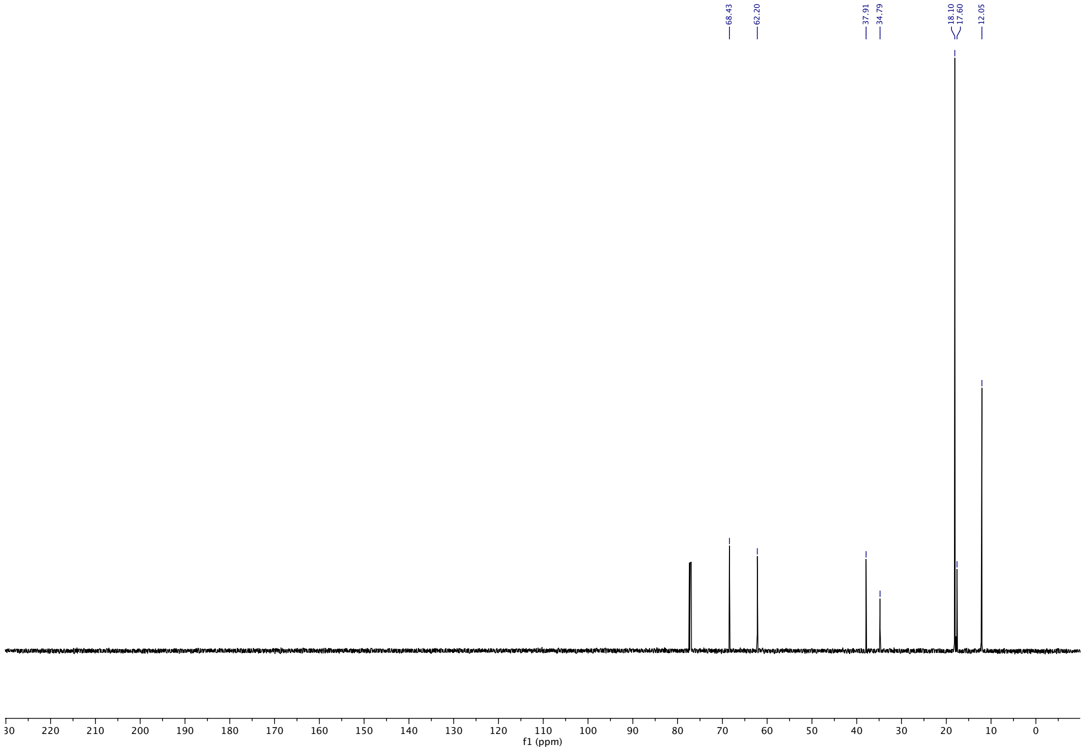

^{1}H NMR (500 MHz, $CDCl_3$) and ^{13}C NMR (150 MHz, $CDCl_3$) of **S13**

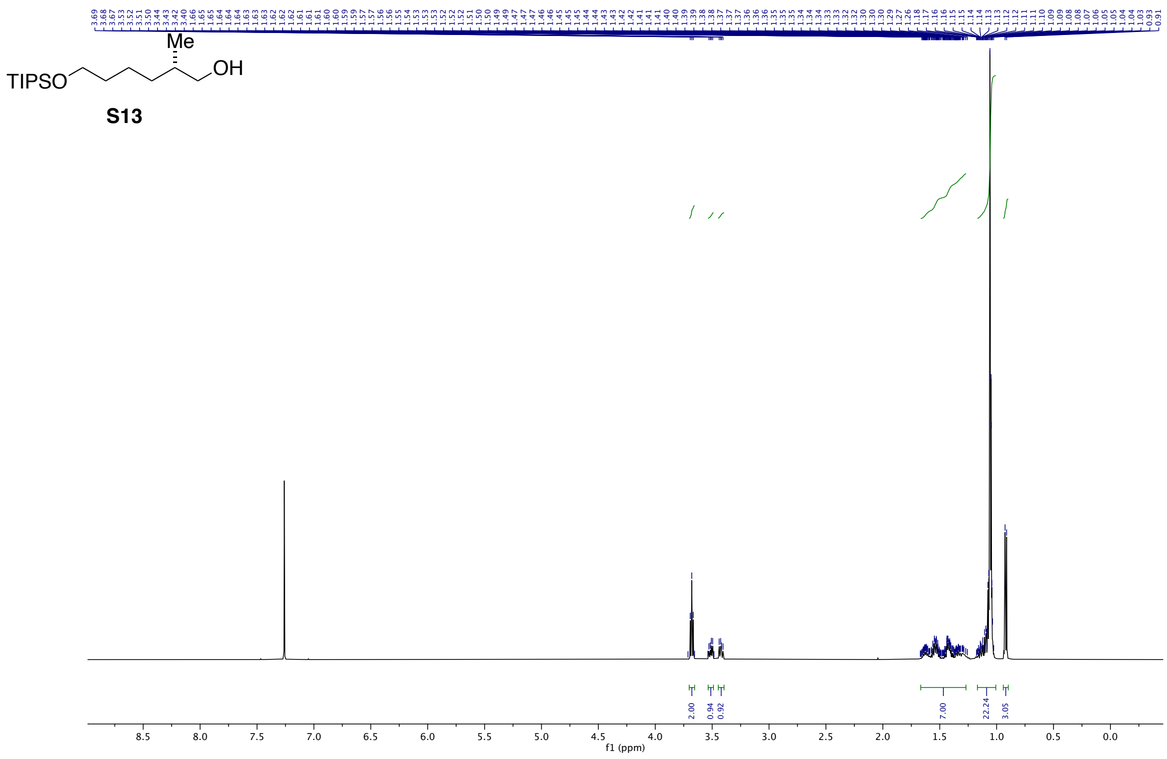

S13

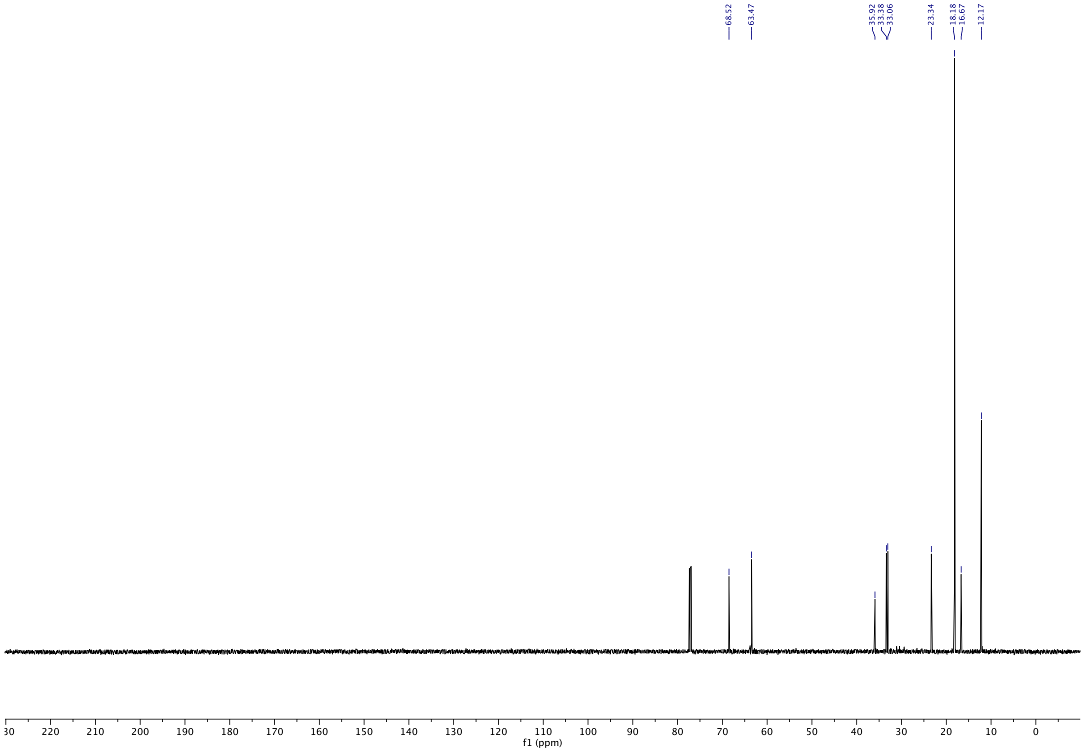

¹H NMR (600 MHz, $CDCl_3$) and ¹³C NMR (150 MHz, $CDCl_3$) of **S14**

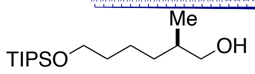

S14

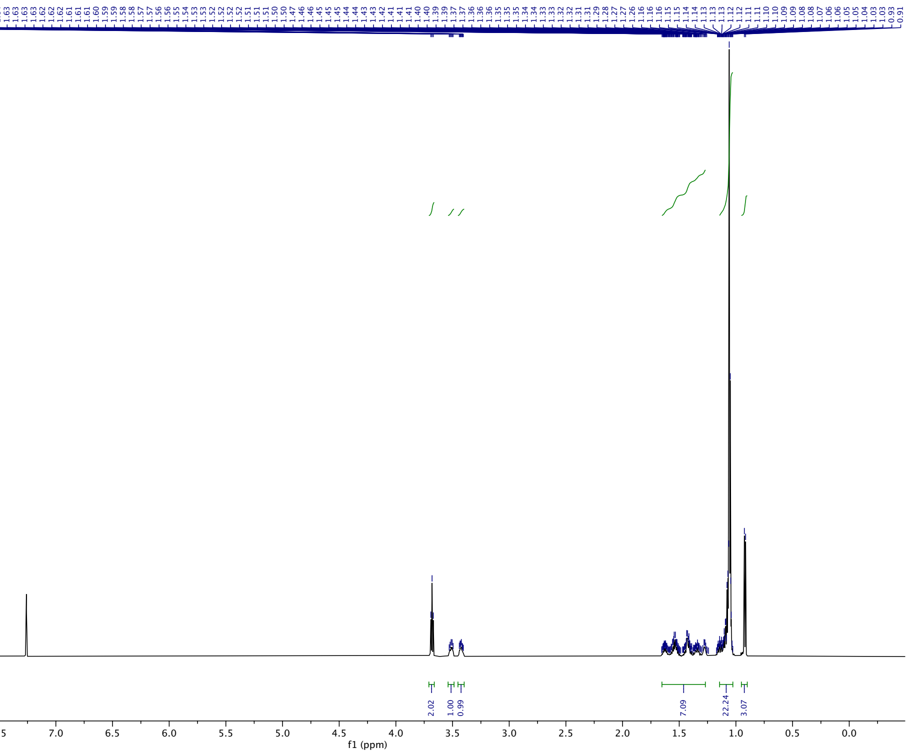

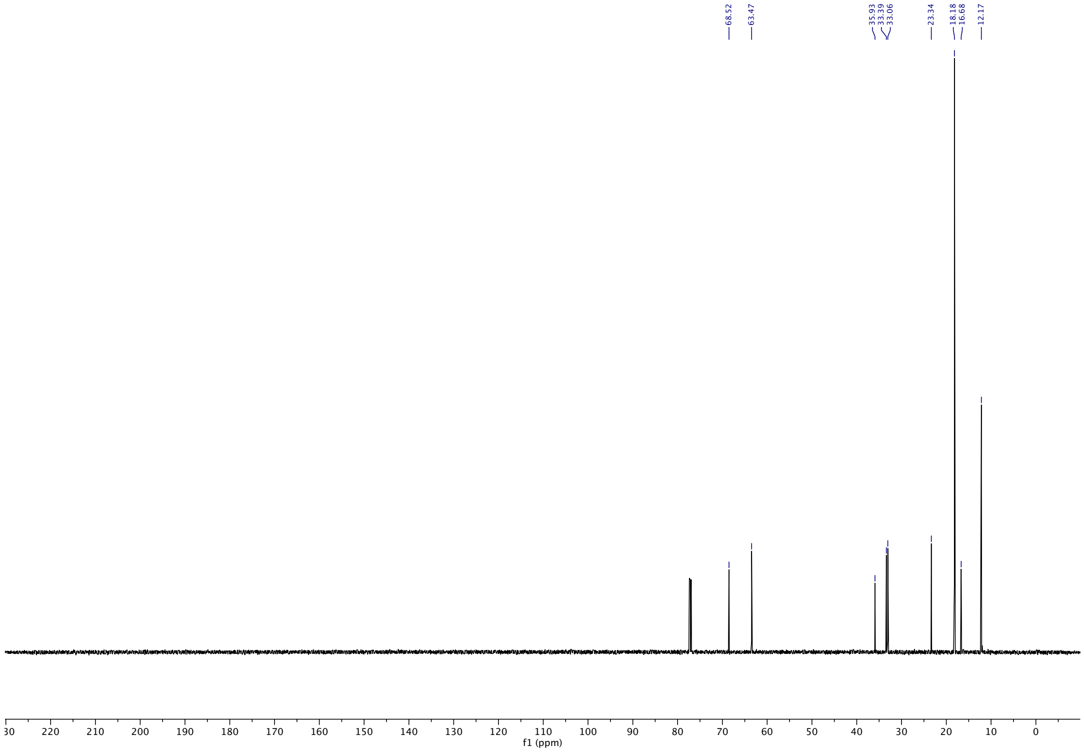

^{1}H NMR (600 MHz, $CDCl_3$) and ^{13}C NMR (150 MHz, $CDCl_3$) of **S15**

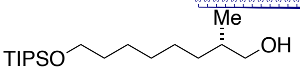

S15

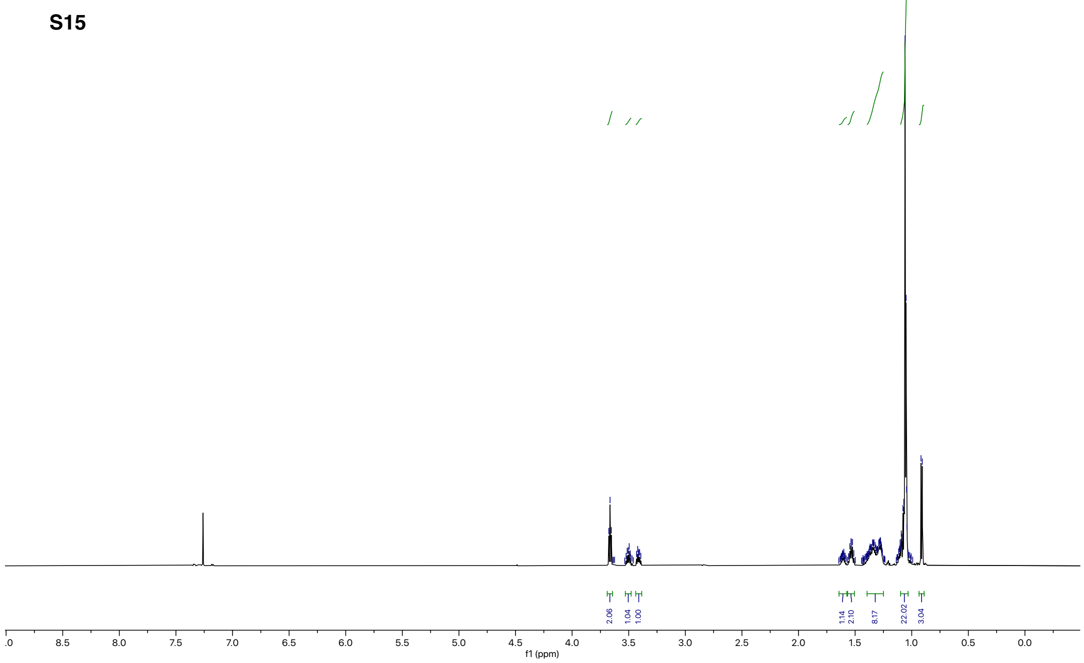

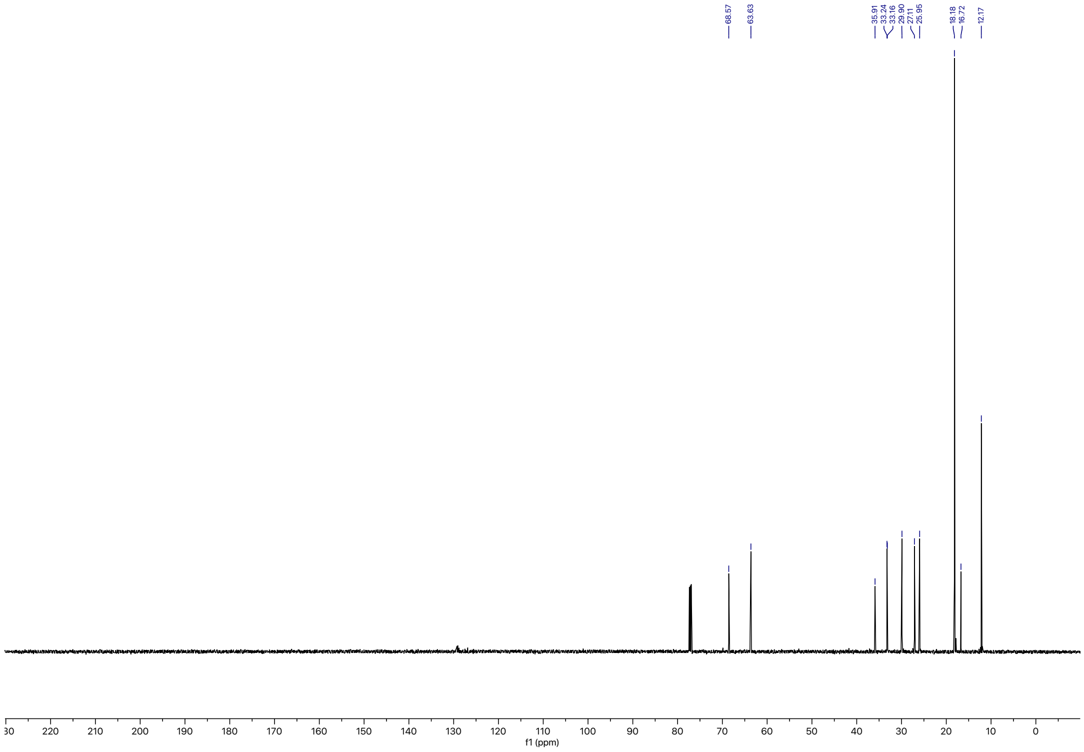

^{1}H NMR (600 MHz, $CDCl_3$) and ^{13}C NMR (150 MHz, $CDCl_3$) of **S16**

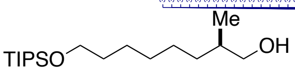

S16

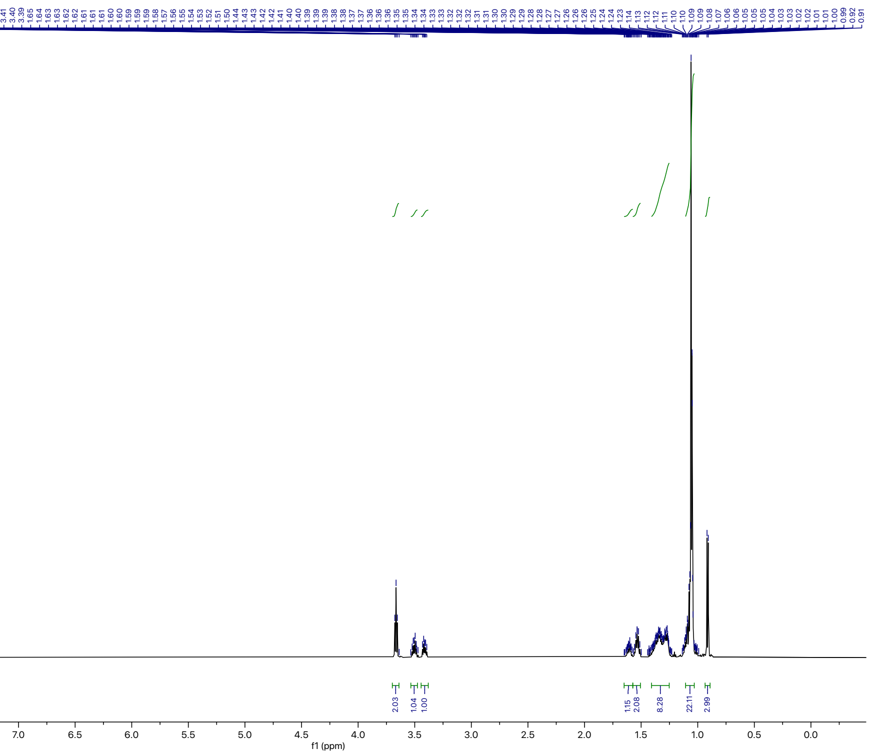

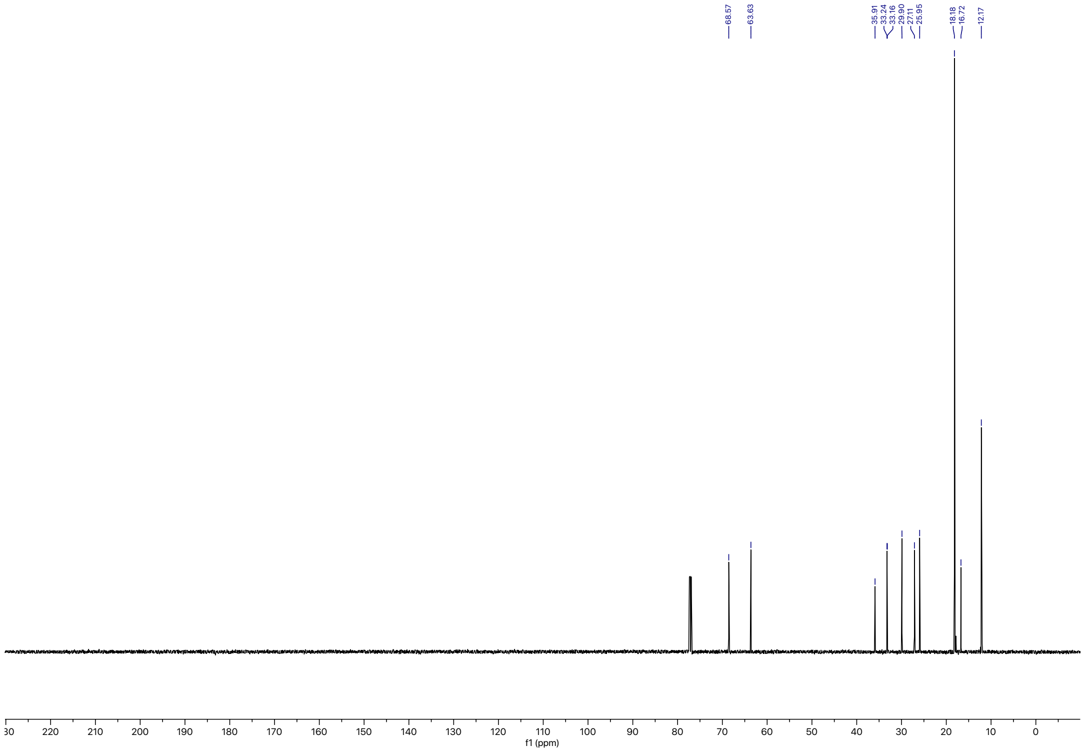

^1H NMR (600 MHz, $CDCl_3$) and ^{13}C NMR (150 MHz, $CDCl_3$) of **S17**

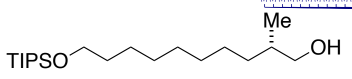

S17

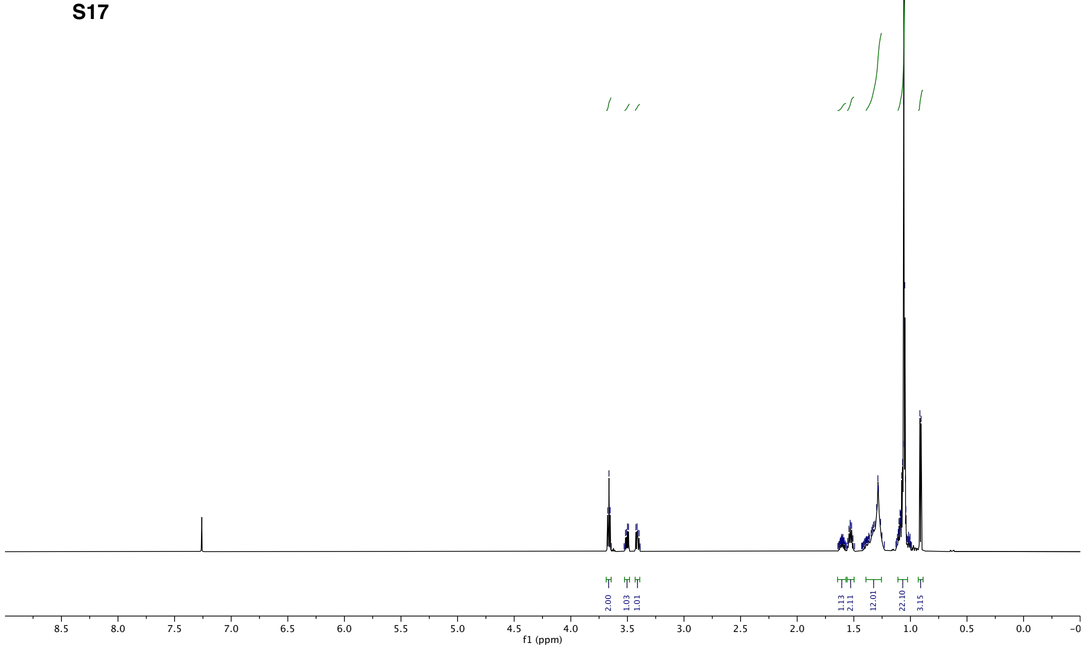

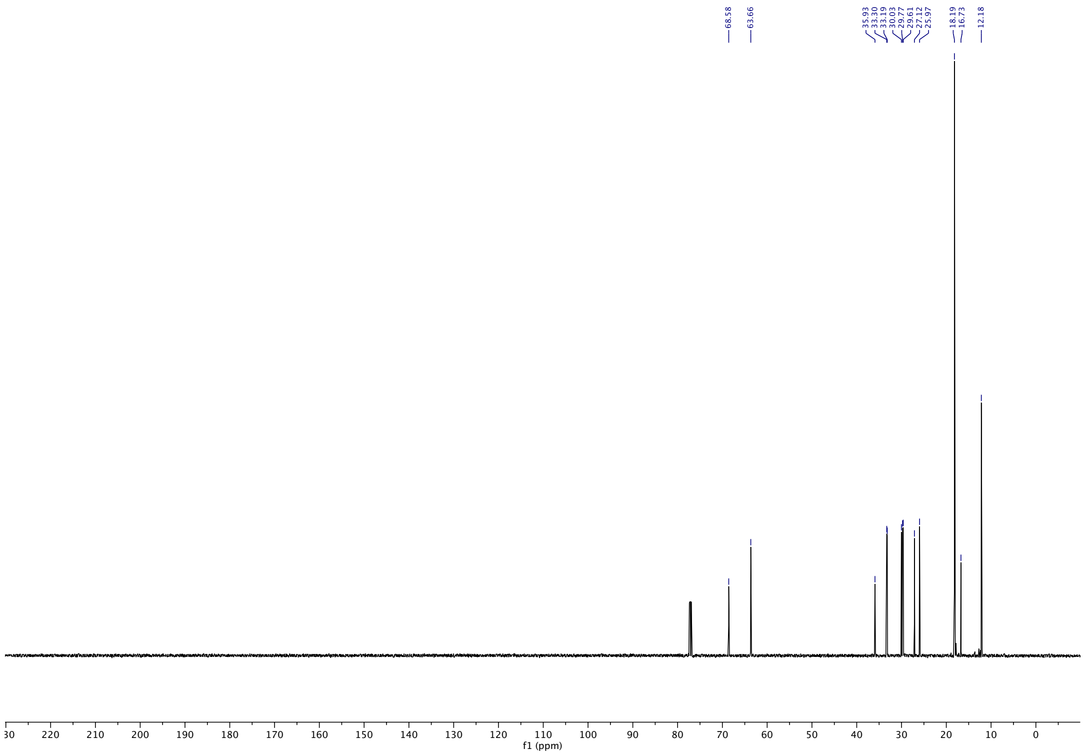

^{1}H NMR (600 MHz, $CDCl_3$) and ^{13}C NMR (150 MHz, $CDCl_3$) of **S18**

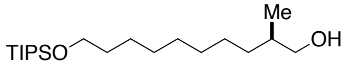

S18

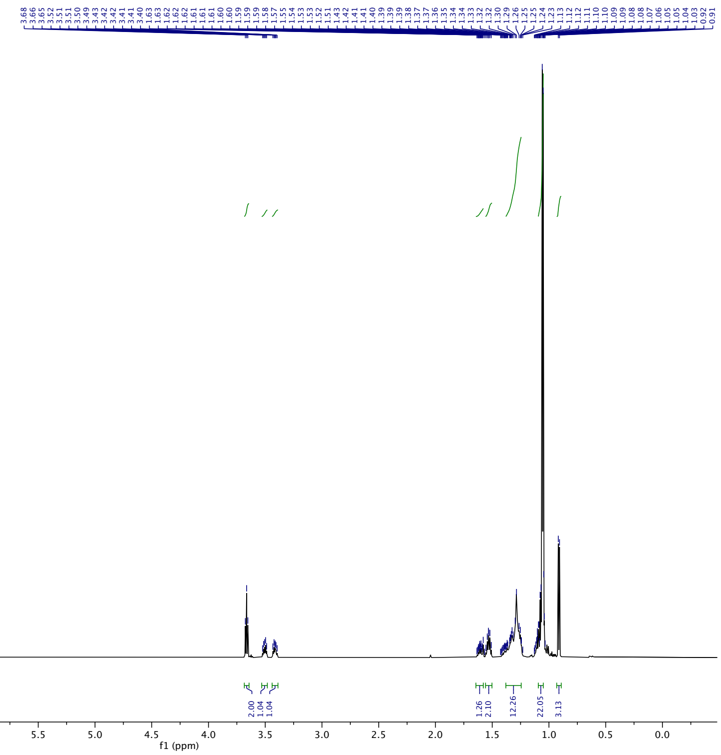

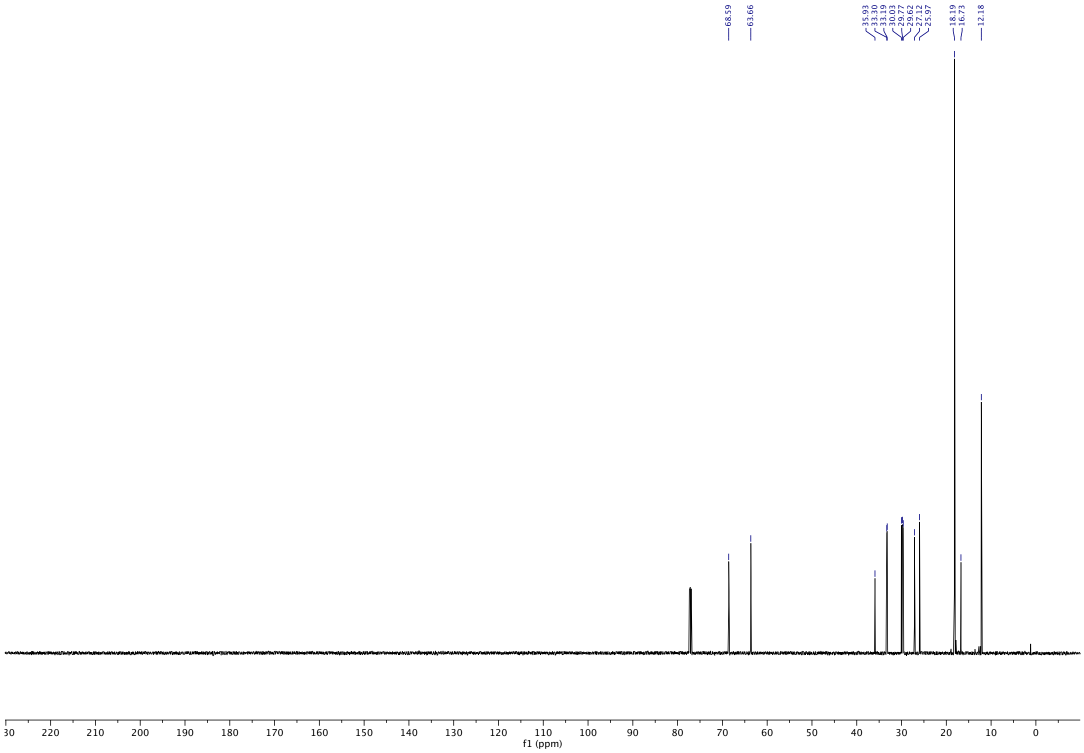

^1H NMR (600 MHz, $CDCl_3$) and ^{13}C NMR (150 MHz, $CDCl_3$) of **1**

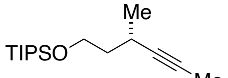

1

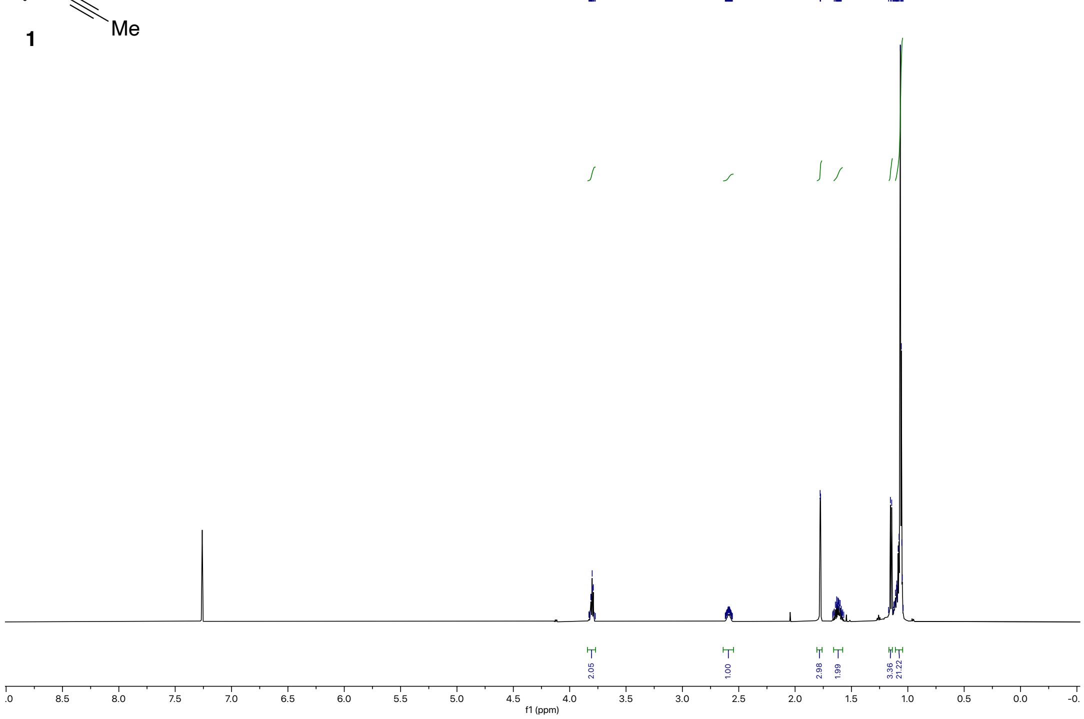

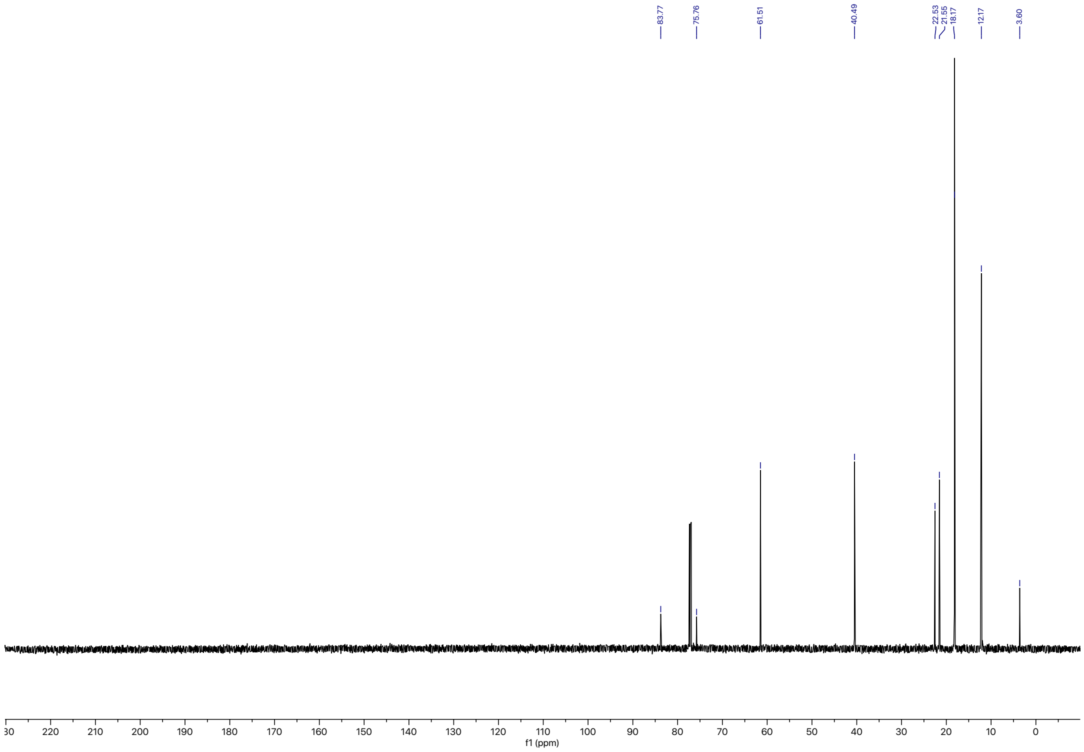

^{1}H NMR (600 MHz, $CDCl_3$) and ^{13}C NMR (150 MHz, $CDCl_3$) of **2**

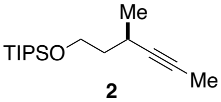

2

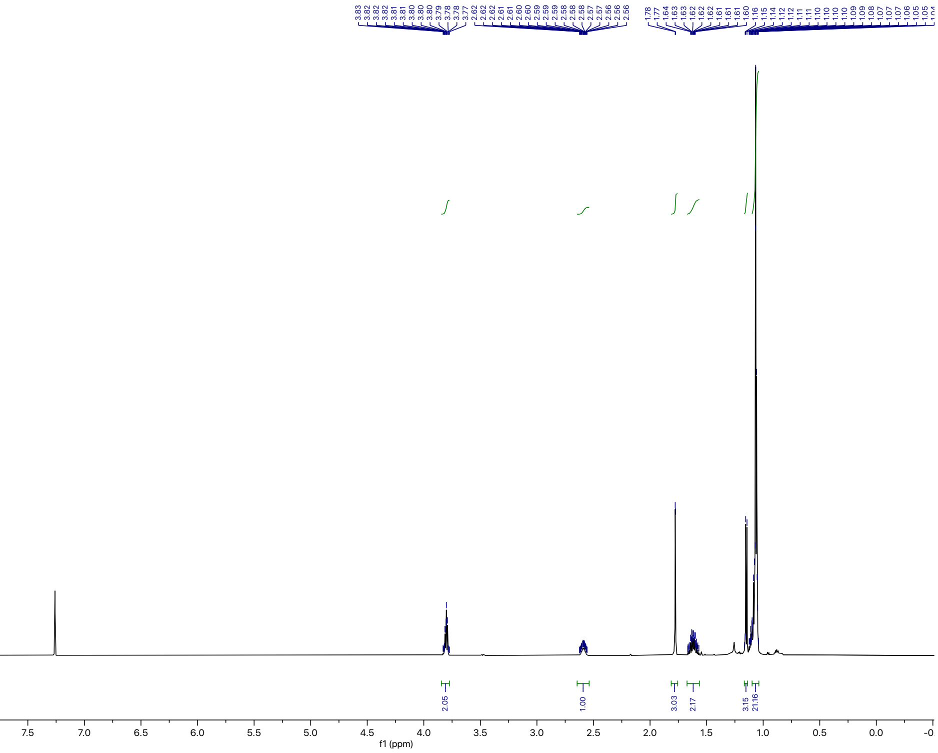

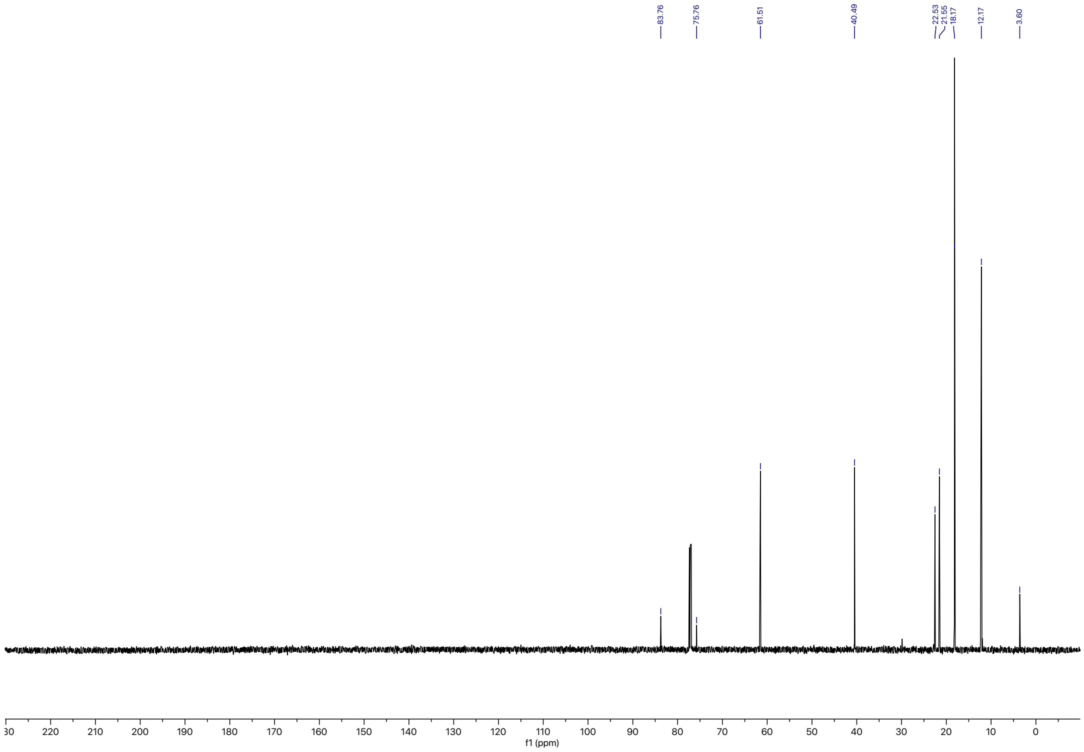

^{1}H NMR (600 MHz, $CDCl_3$) and ^{13}C NMR (150 MHz, $CDCl_3$) of **3**

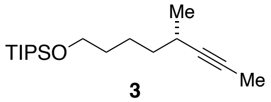

3

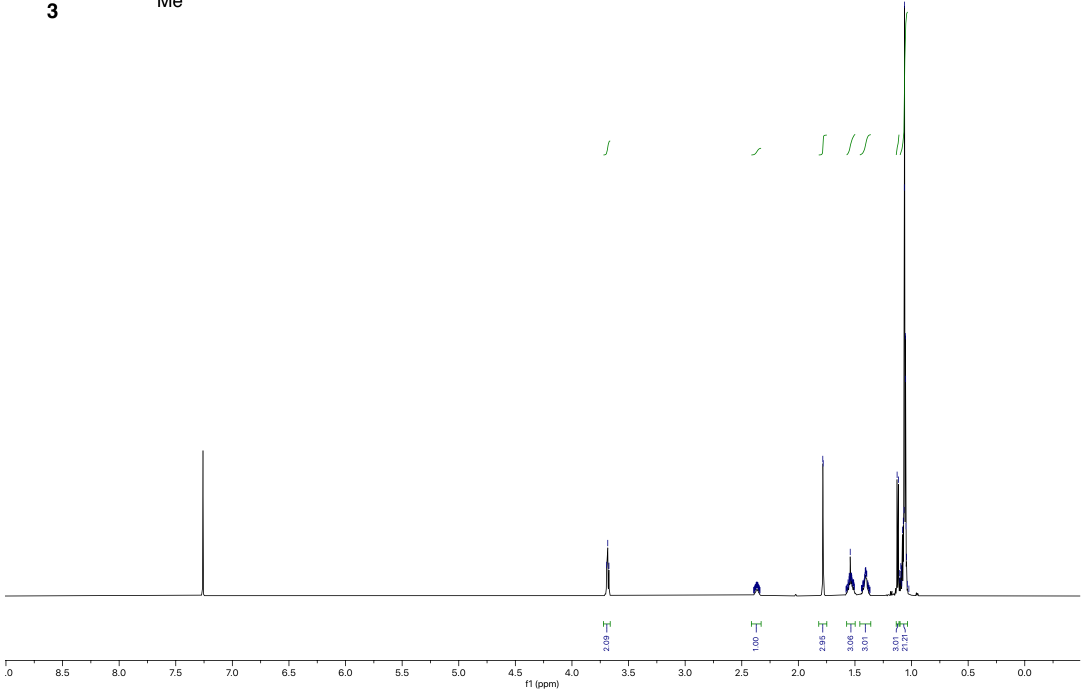

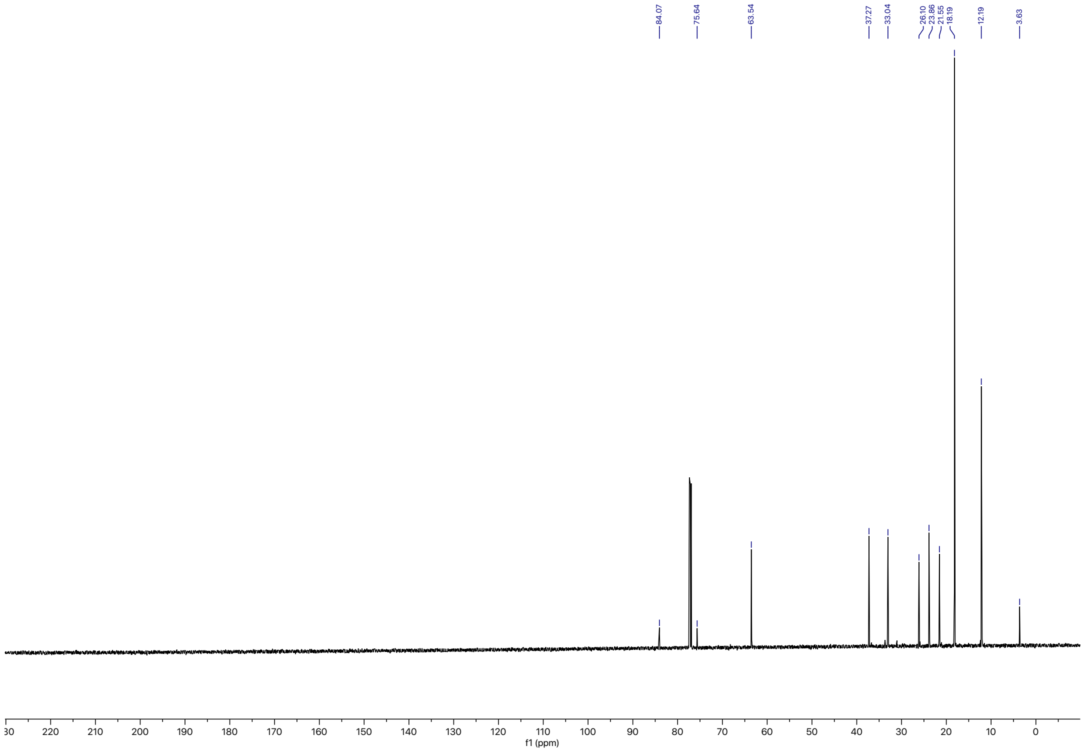

^1H NMR (600 MHz, $CDCl_3$) and ^{13}C NMR (150 MHz, $CDCl_3$) of **4**

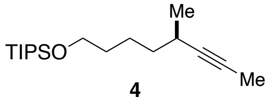

4

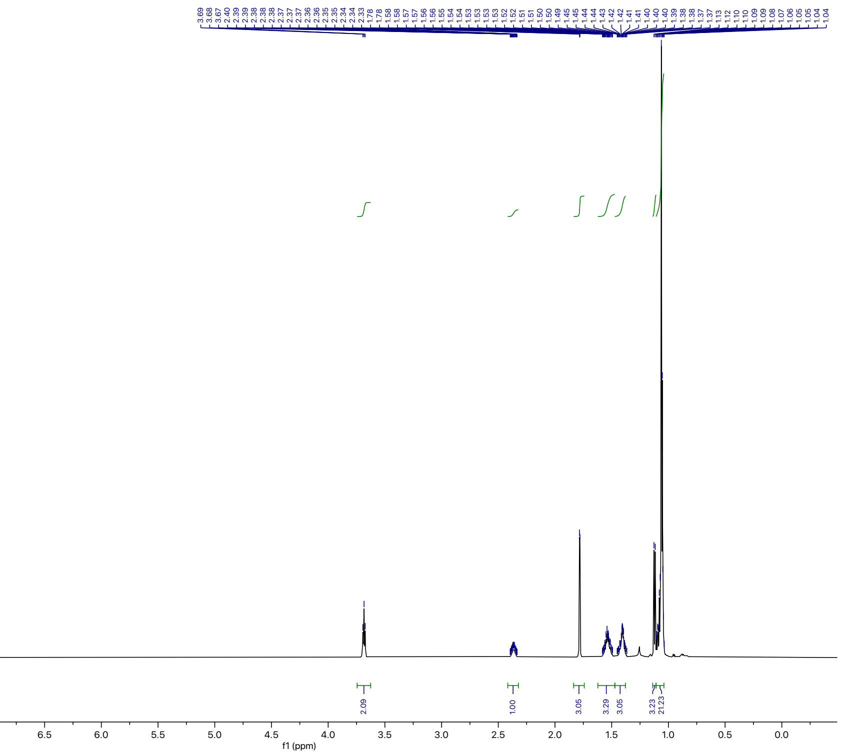

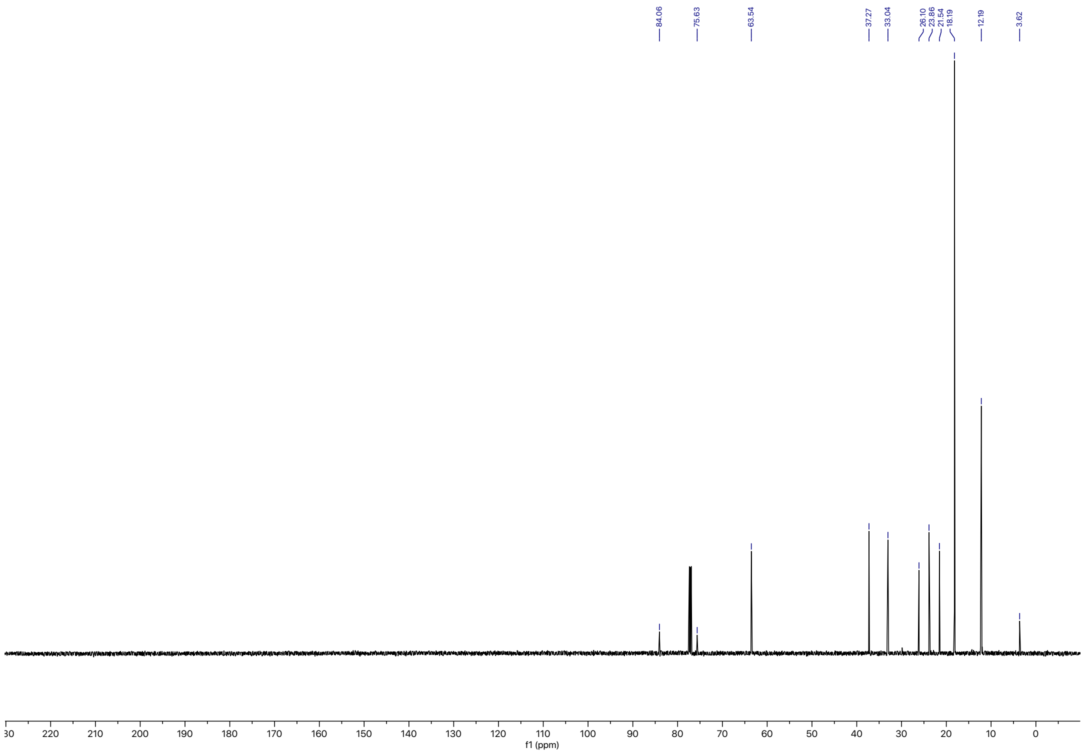

^1H NMR (600 MHz, $CDCl_3$) and ^{13}C NMR (150 MHz, $CDCl_3$) of **5**

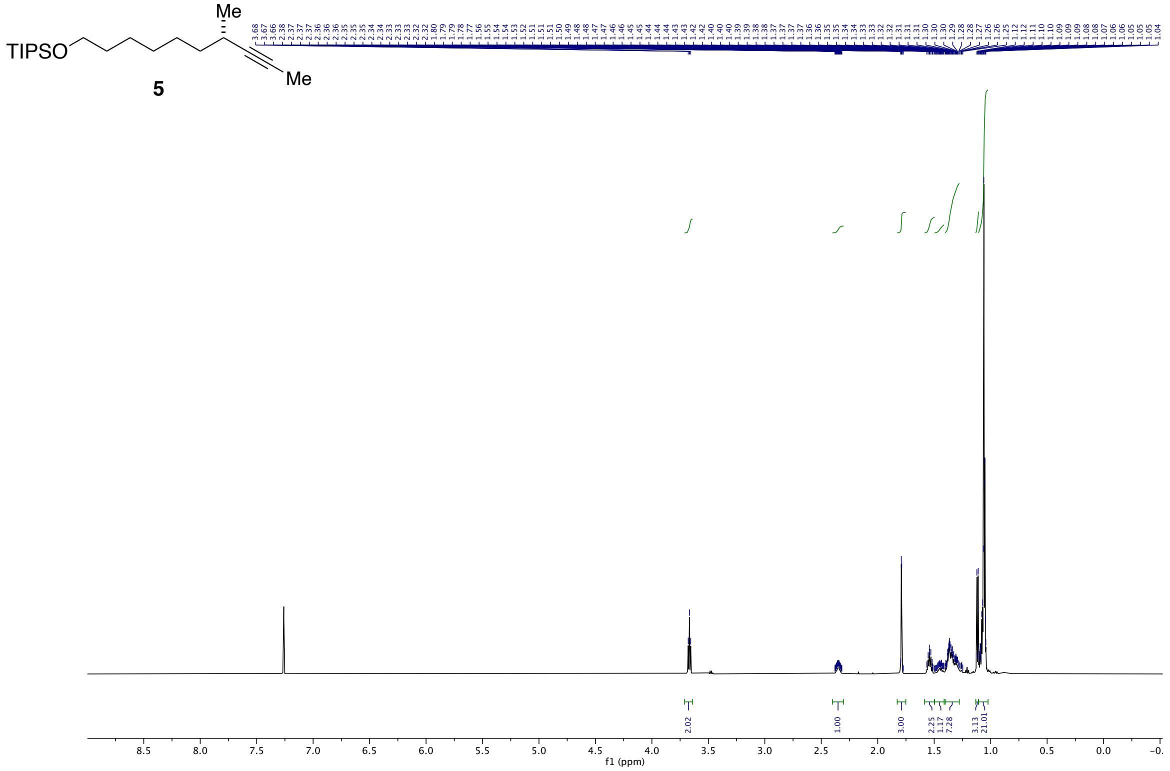

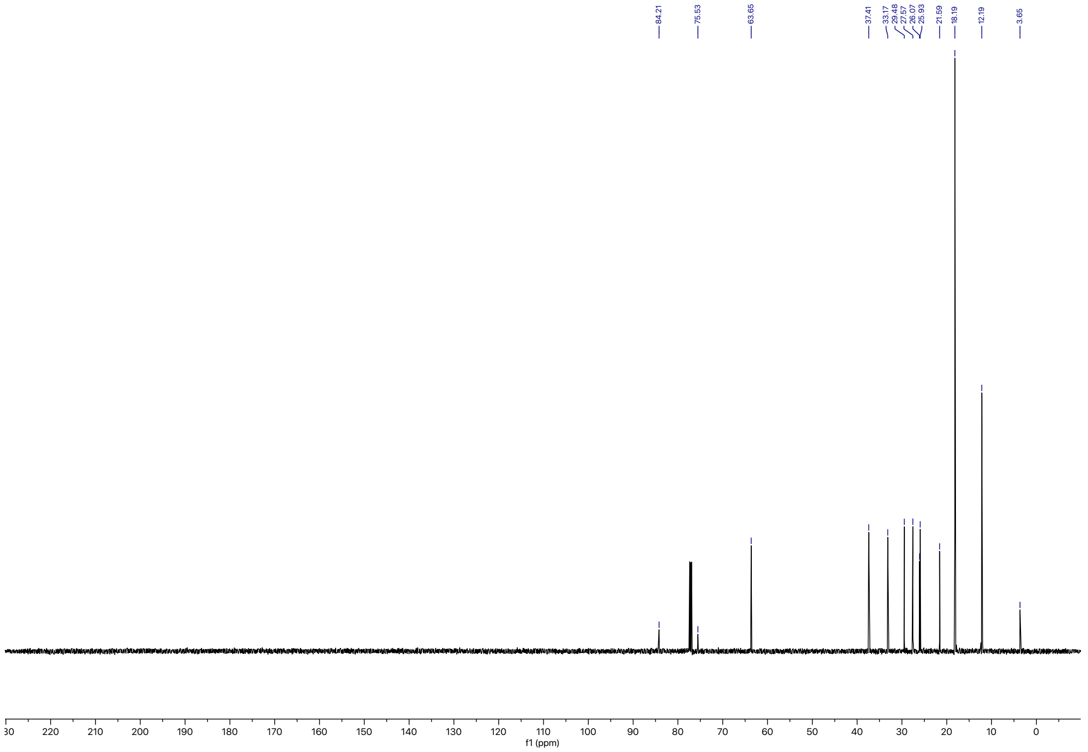

^{1}H NMR (600 MHz, $CDCl_3$) and ^{13}C NMR (150 MHz, $CDCl_3$) of **6**

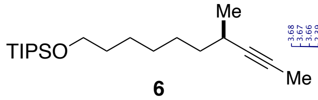

6

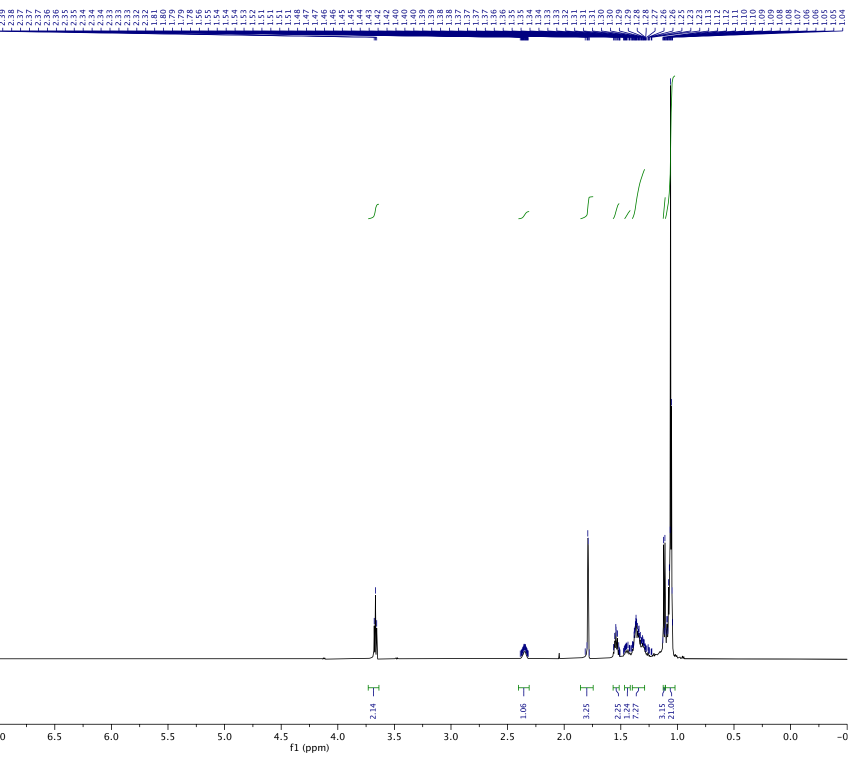

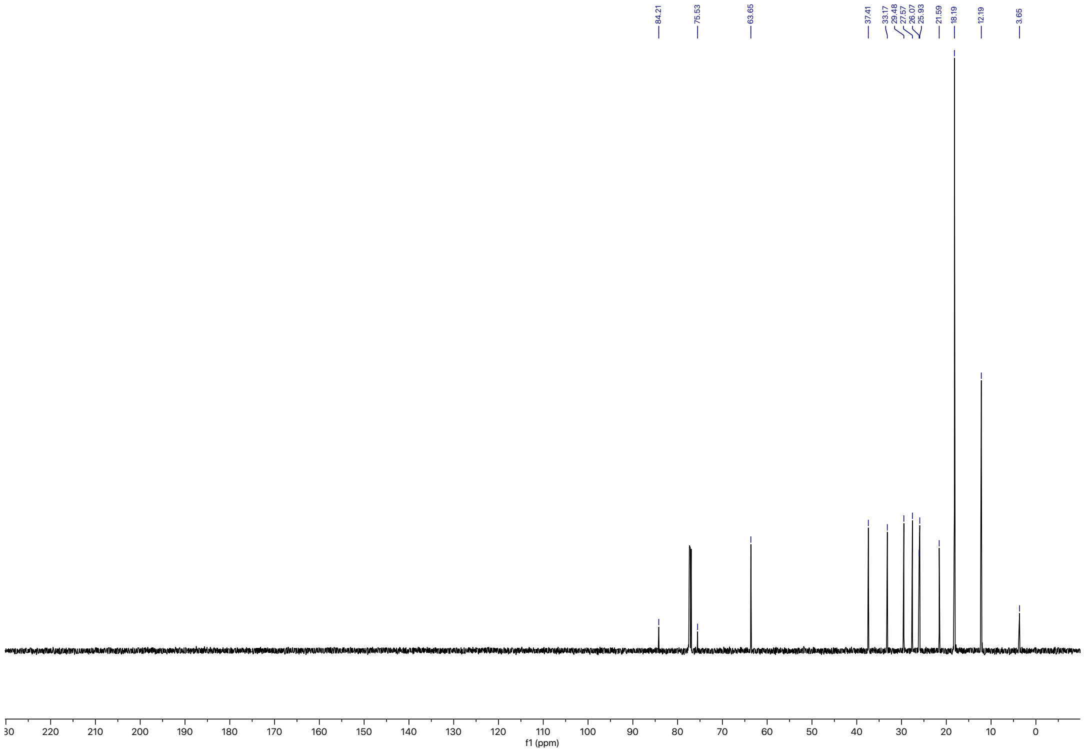

^{1}H NMR (600 MHz, $CDCl_3$) and ^{13}C NMR (150 MHz, $CDCl_3$) of **7**

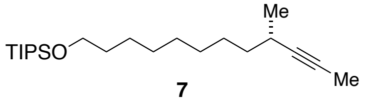

7

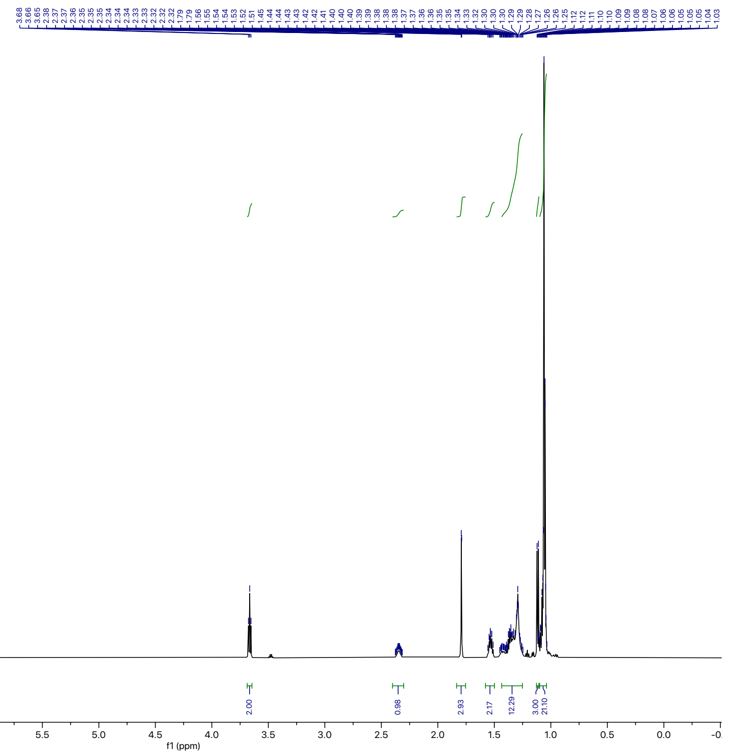

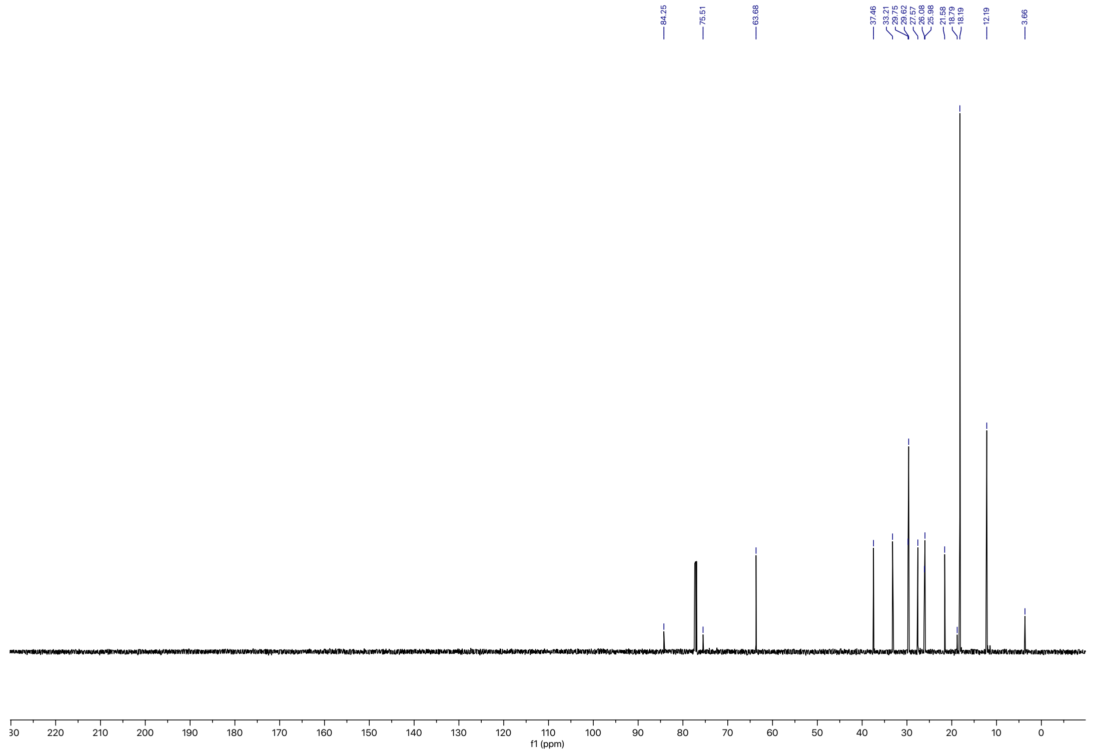

^{1}H NMR (600 MHz, $CDCl_3$) and ^{13}C NMR (150 MHz, $CDCl_3$) of **8**

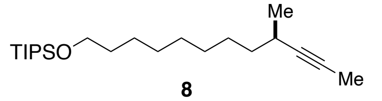

8

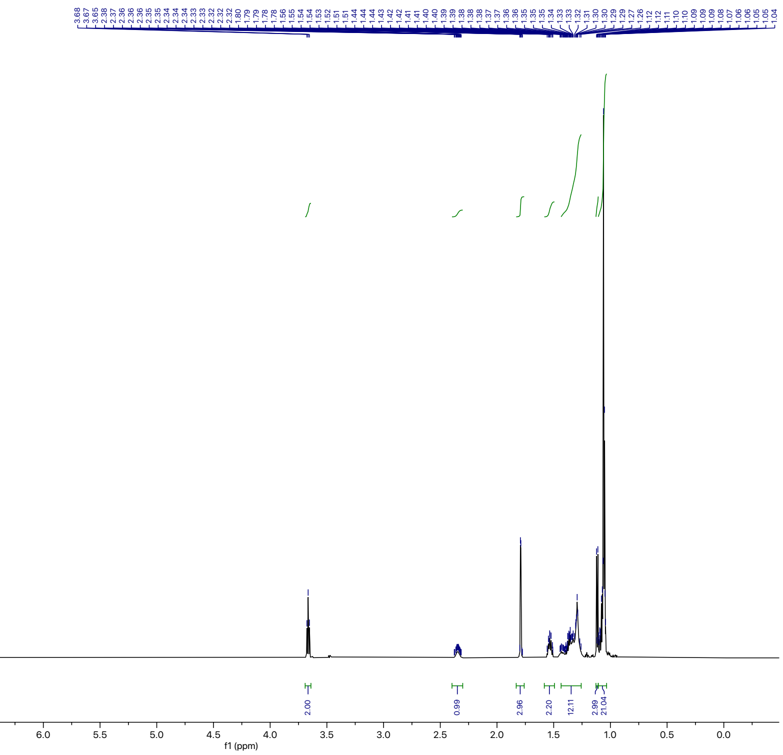

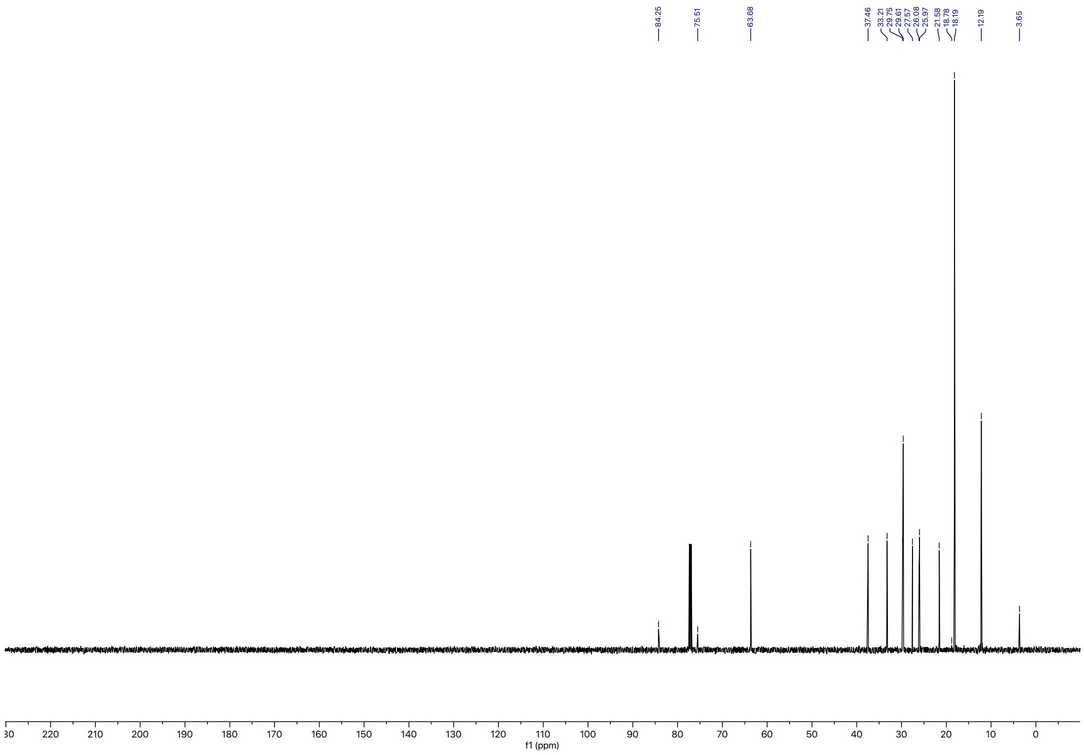

^{1}H NMR (600 MHz, $CDCl_3$) and ^{13}C NMR (150 MHz, $CDCl_3$) of **S19**

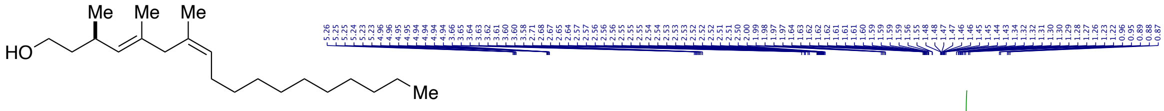

S19

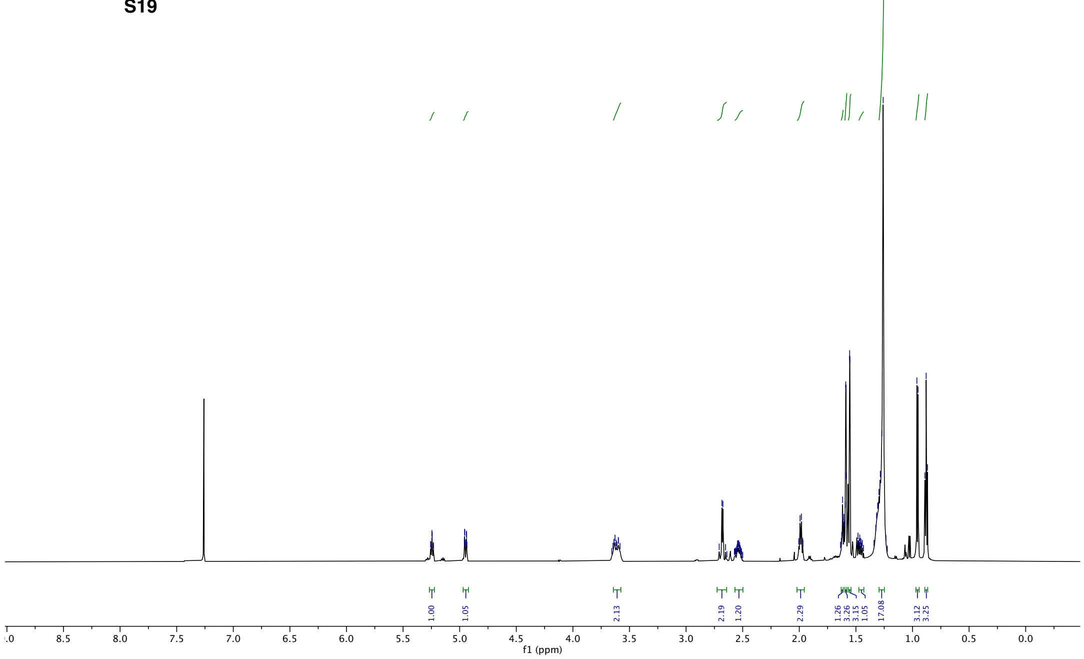

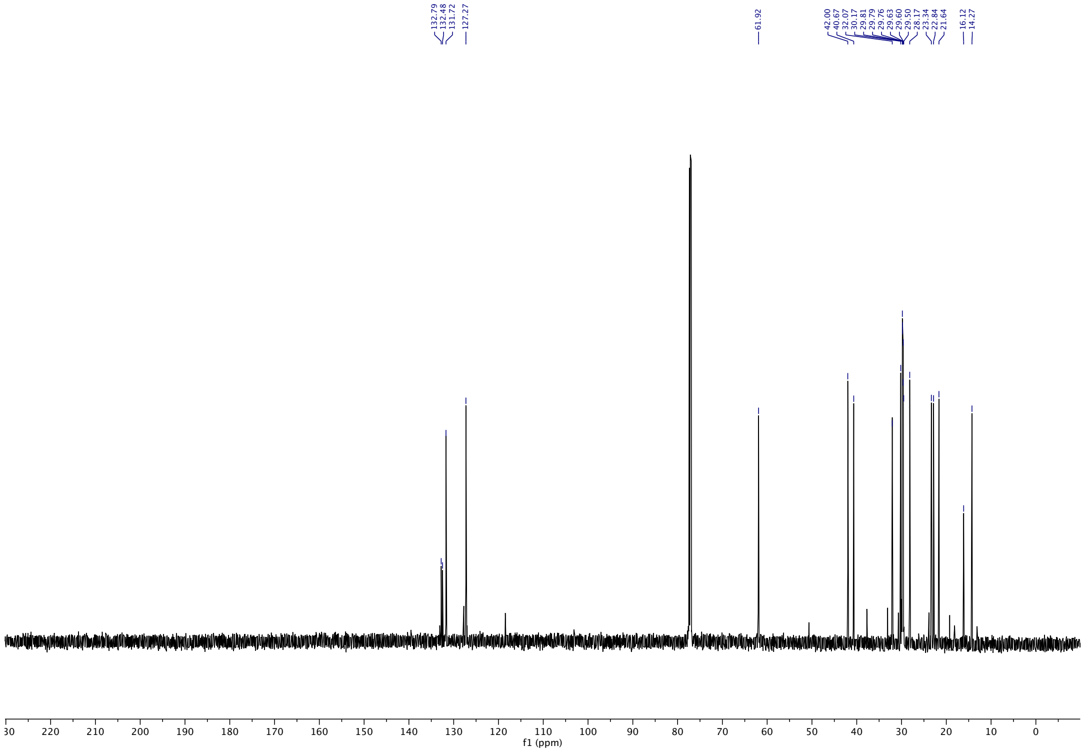

^{1}H NMR (600 MHz, $CDCl_3$) and ^{13}C NMR (150 MHz, $CDCl_3$) of **S20**

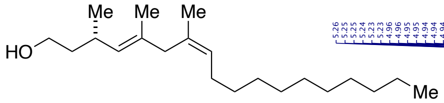

S20

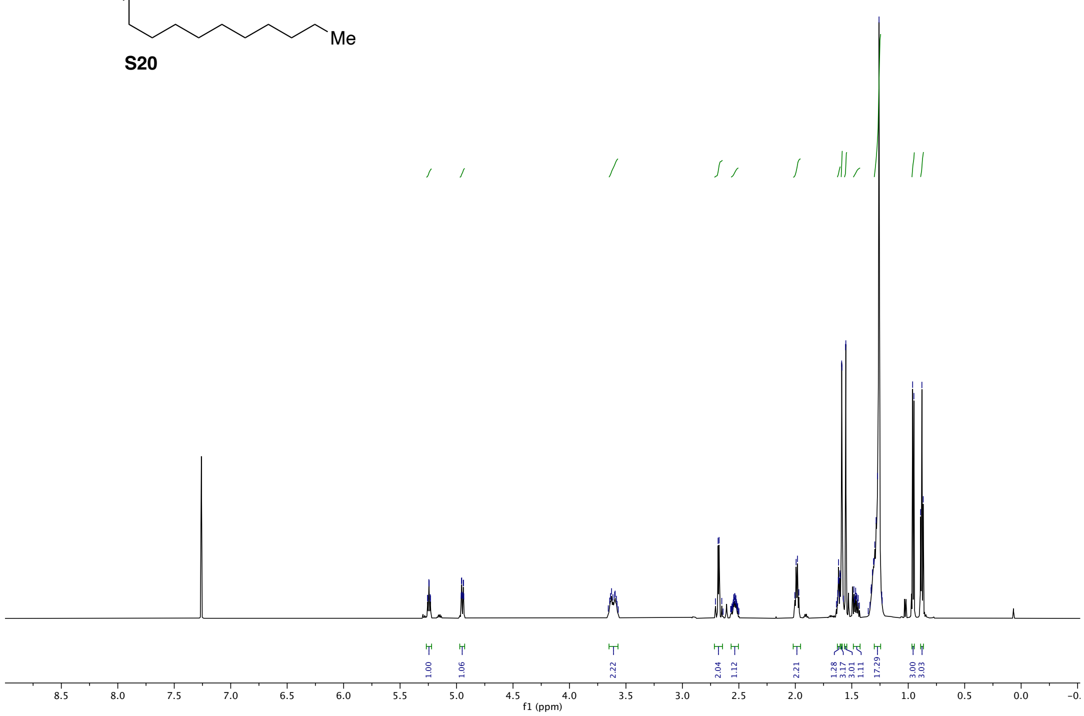

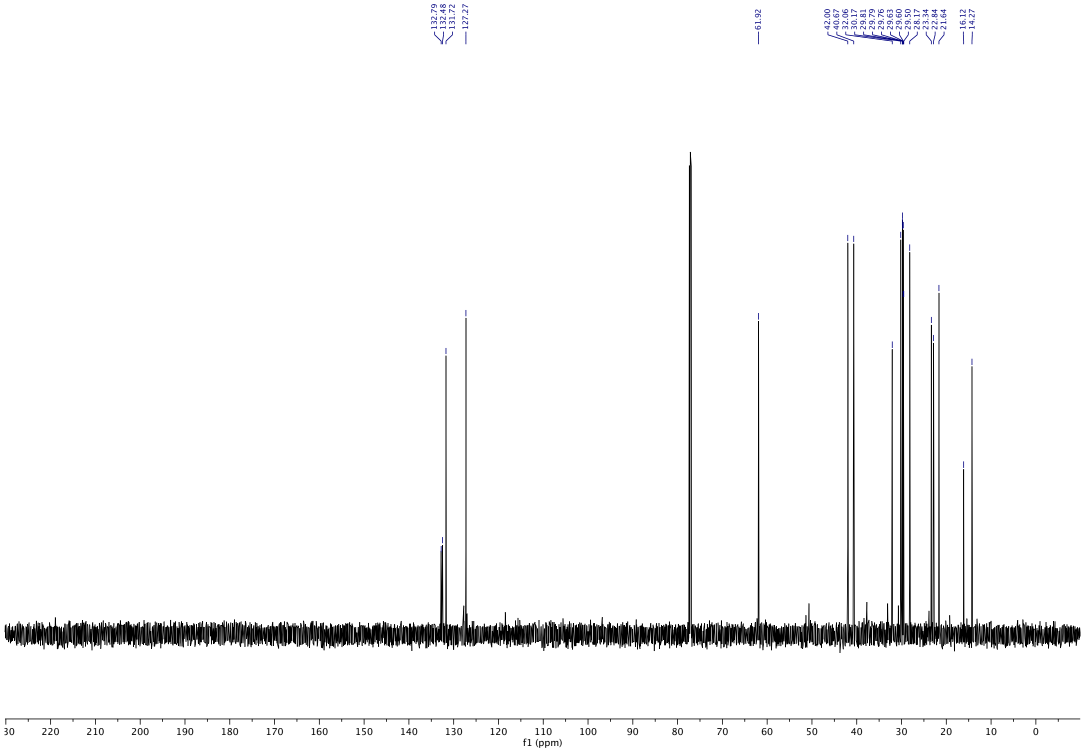

^{1}H NMR (500 MHz, $CDCl_3$) and ^{13}C NMR (150 MHz, $CDCl_3$) of **S21**

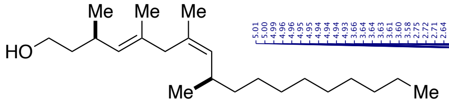

S21

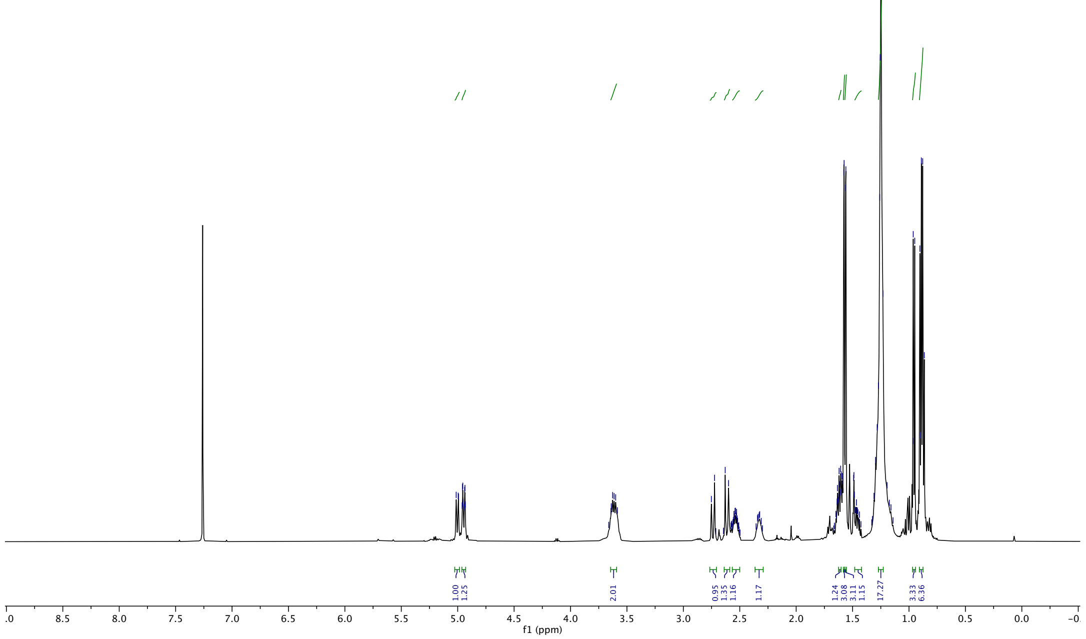

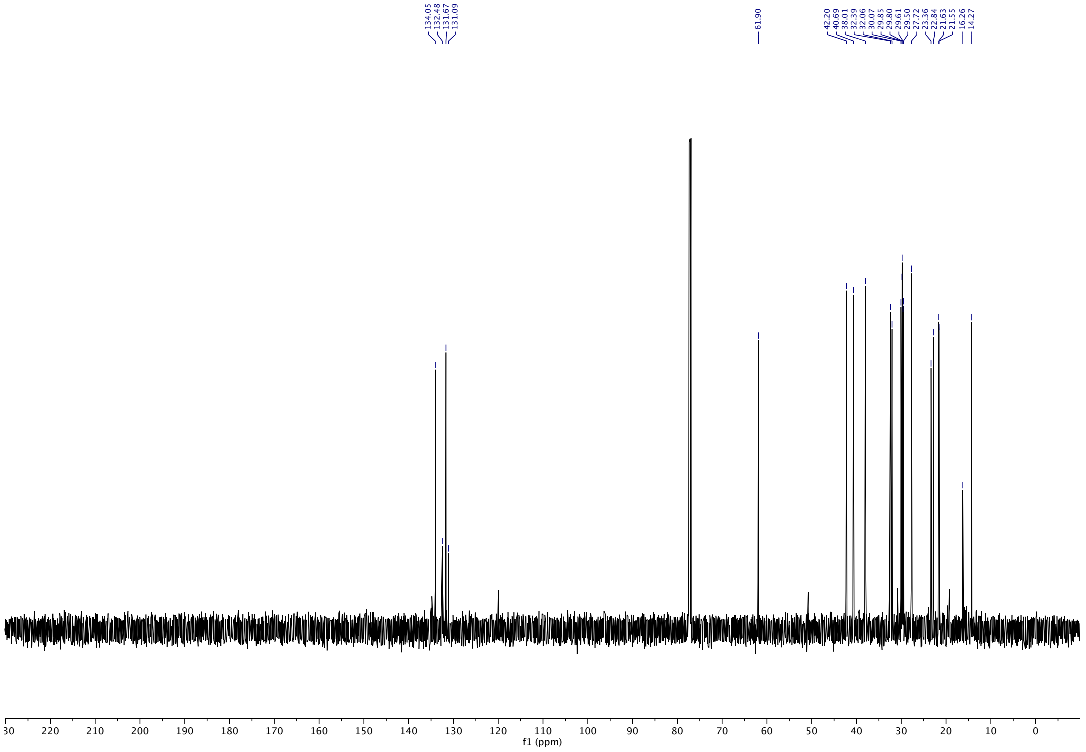

^{1}H NMR (600 MHz, $CDCl_3$) and ^{13}C NMR (150 MHz, $CDCl_3$) of **S22**

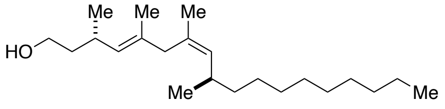

S22

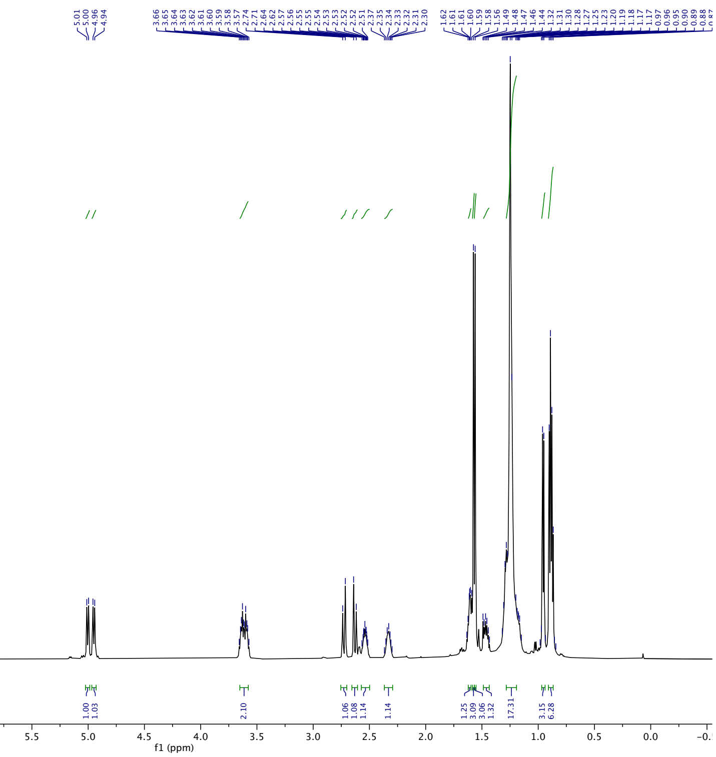

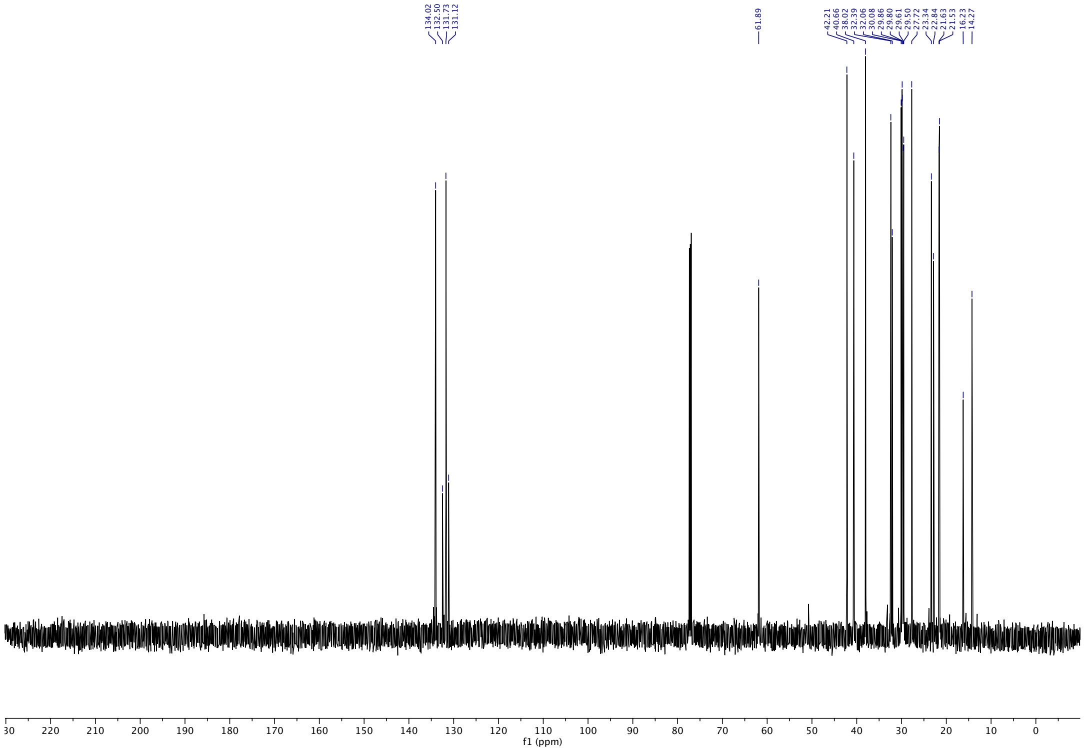

^{1}H NMR (600 MHz, $CDCl_3$) and ^{13}C NMR (150 MHz, $CDCl_3$) of **21**

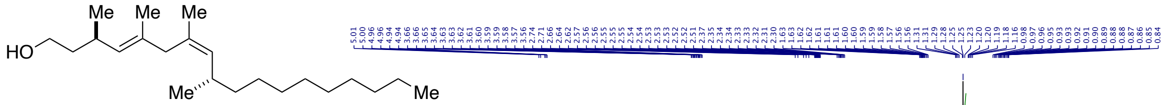

21

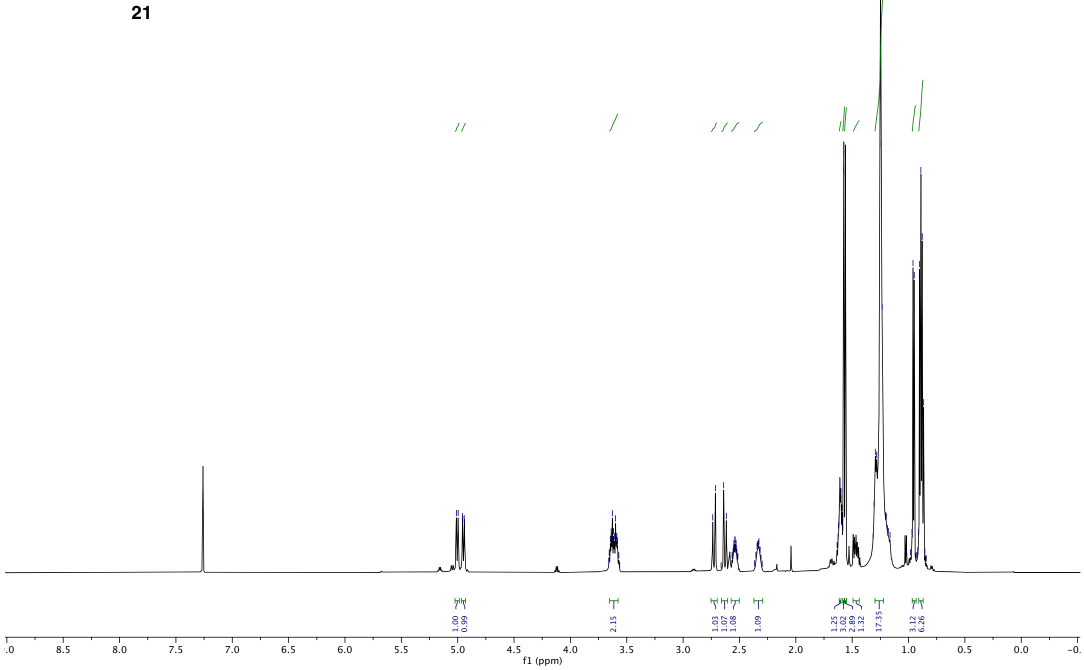

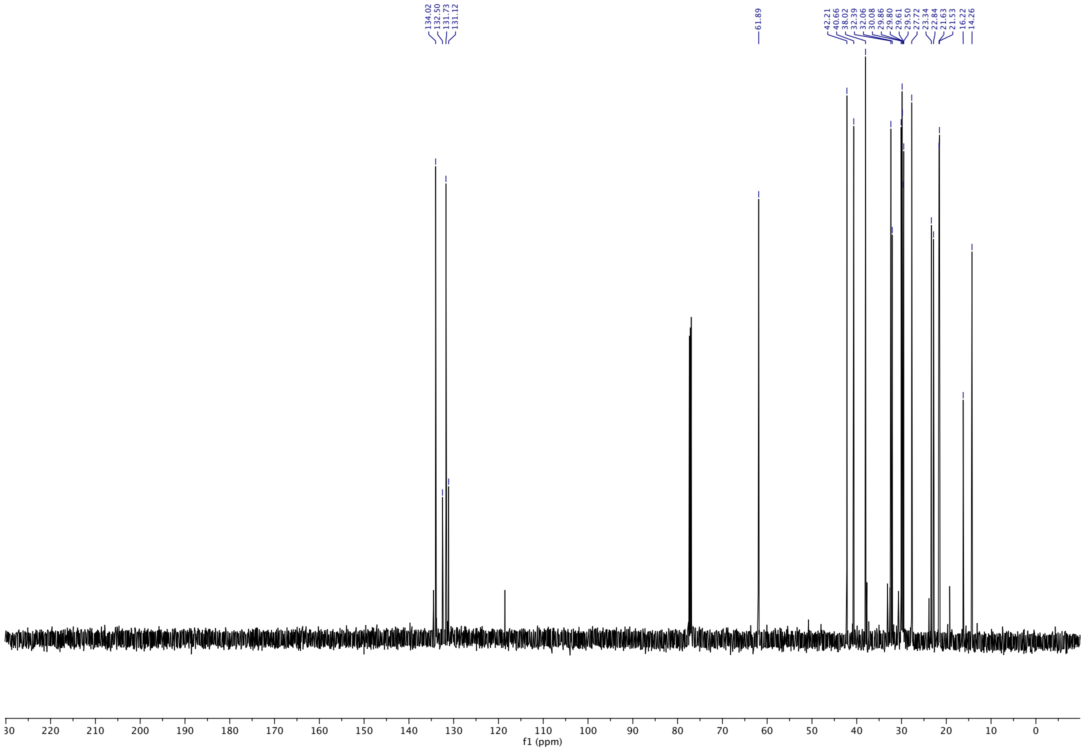

^{1}H NMR (500 MHz, $CDCl_3$) and ^{13}C NMR (150 MHz, $CDCl_3$) of **S23**

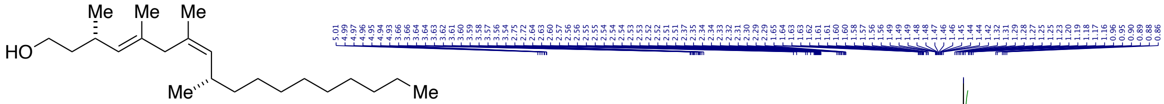

S23

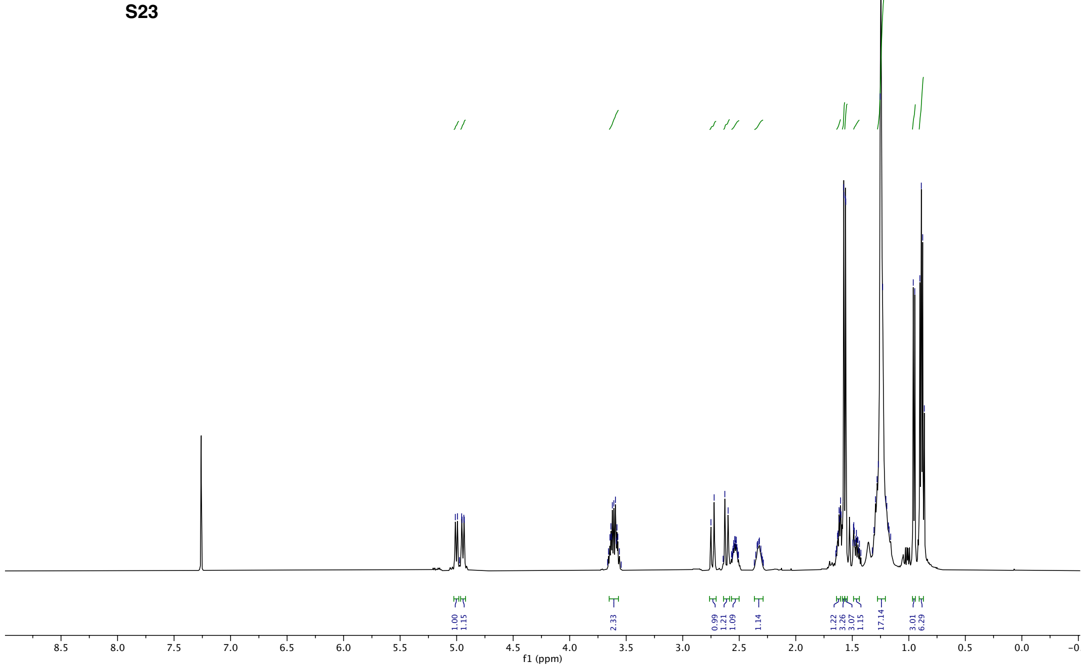

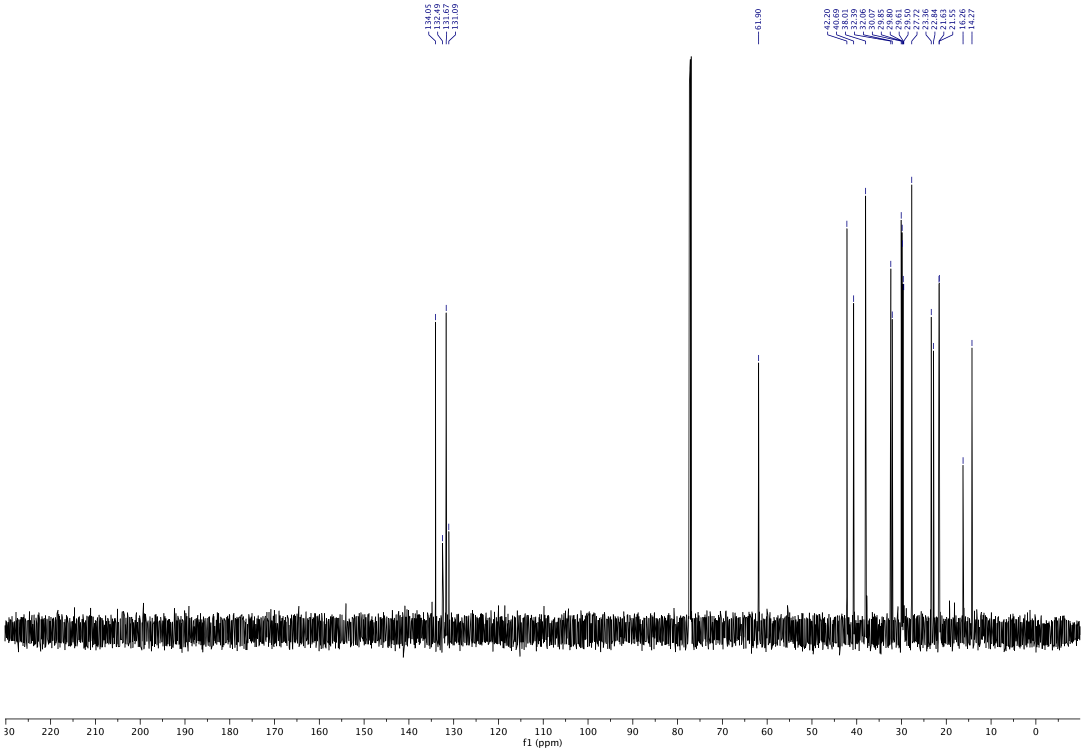

^{1}H NMR (600 MHz, $CDCl_3$) and ^{13}C NMR (150 MHz, $CDCl_3$) of **S24**

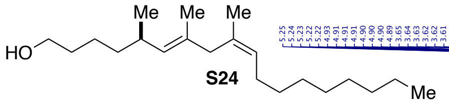

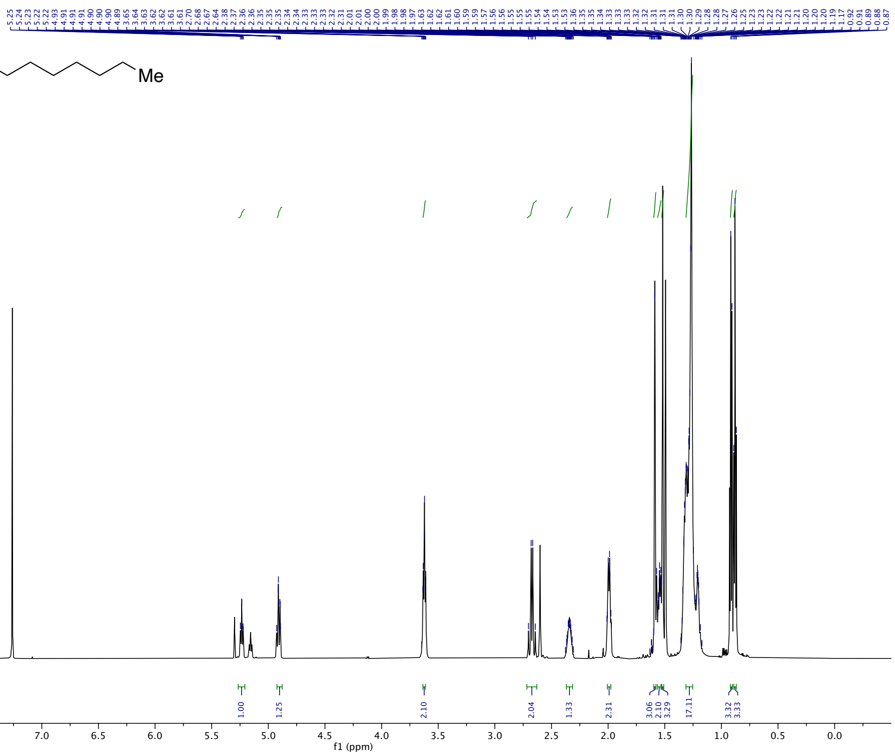

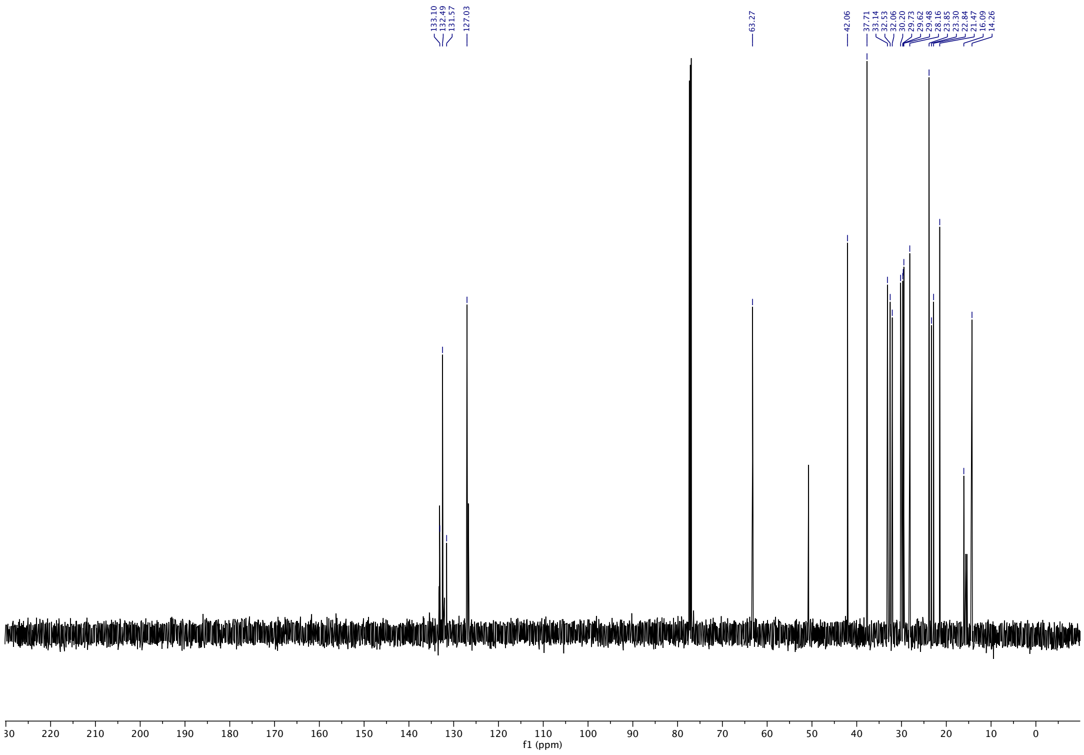

^{1}H NMR (600 MHz, $CDCl_3$) and ^{13}C NMR (150 MHz, $CDCl_3$) of **S25**

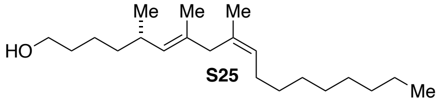

S25

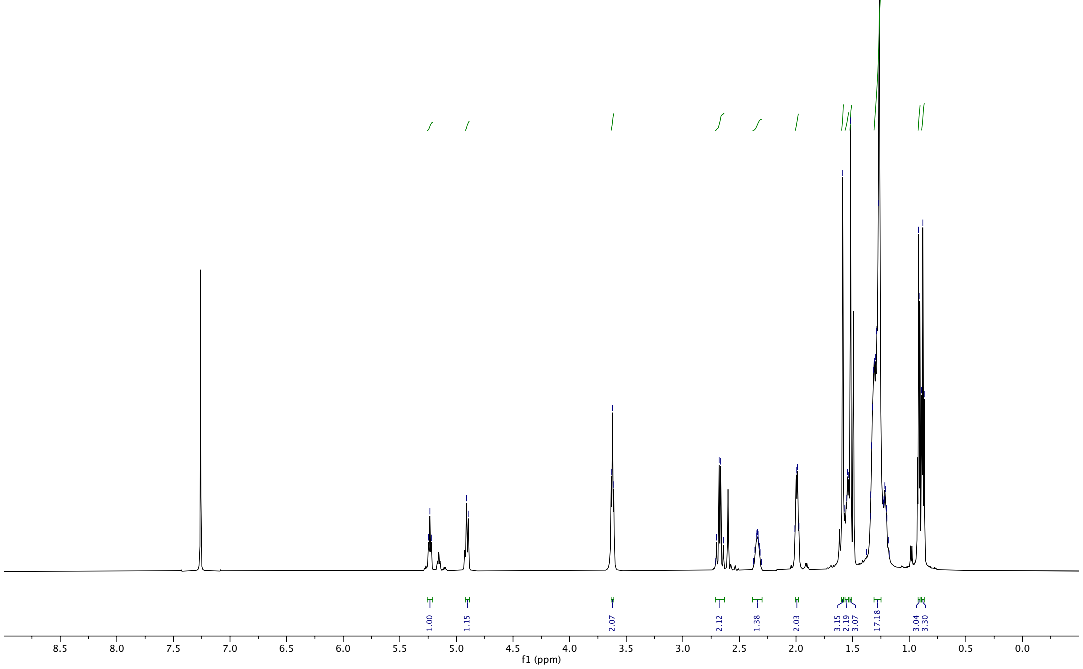

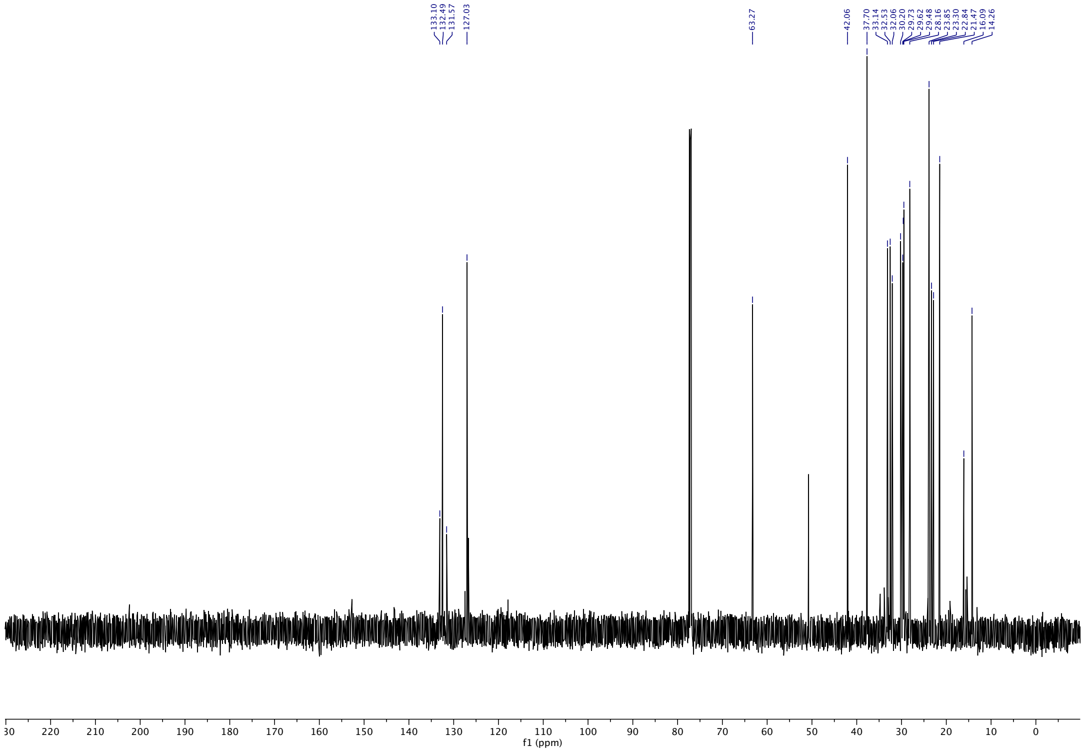

^{1}H NMR (600 MHz, $CDCl_3$) and ^{13}C NMR (150 MHz, $CDCl_3$) of **S26**

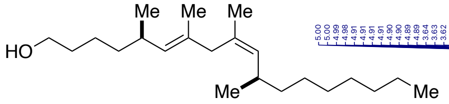

S26

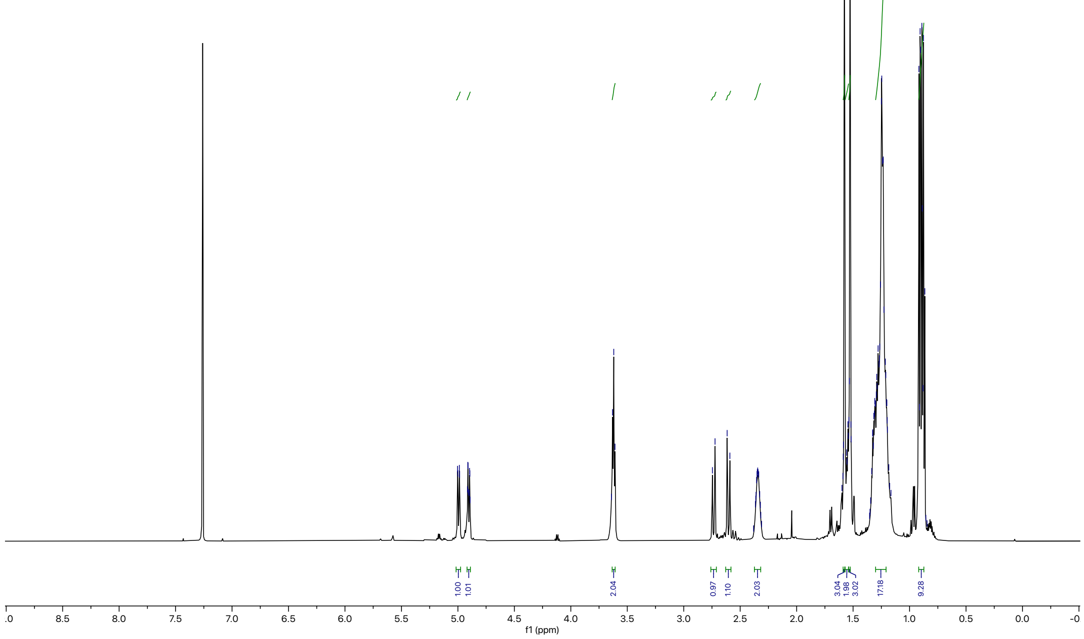

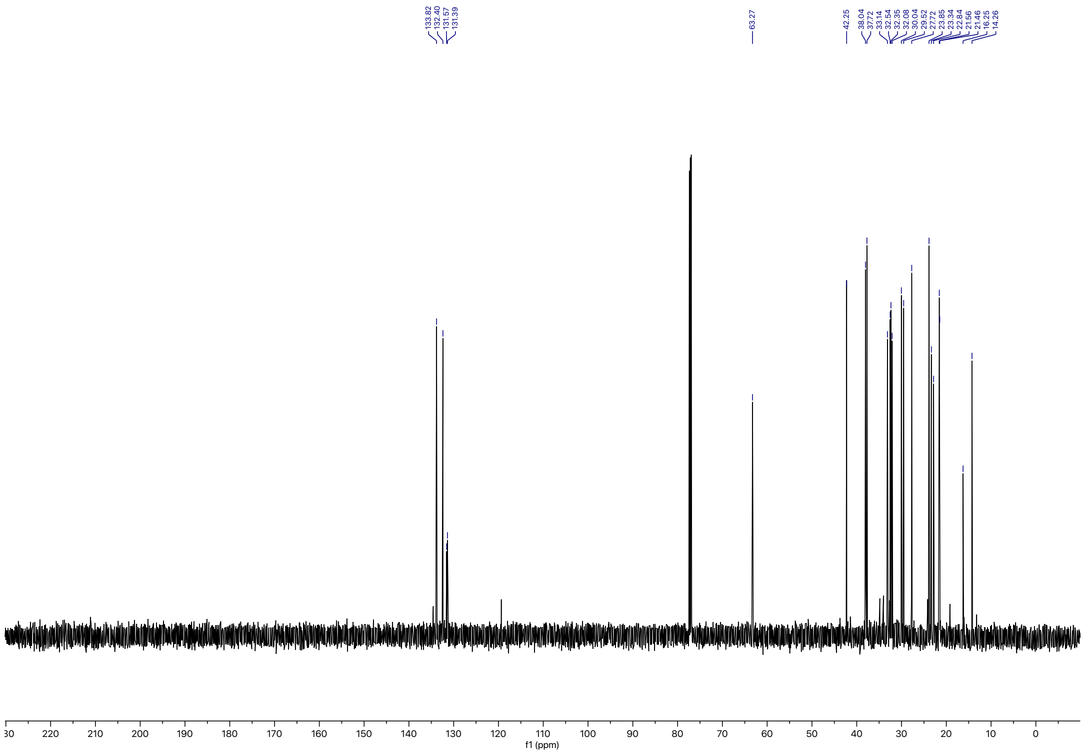

^{1}H NMR (600 MHz, $CDCl_3$) and ^{13}C NMR (150 MHz, $CDCl_3$) of **S27**

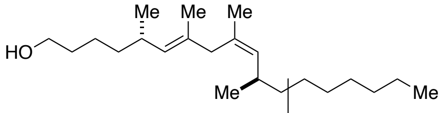

S27

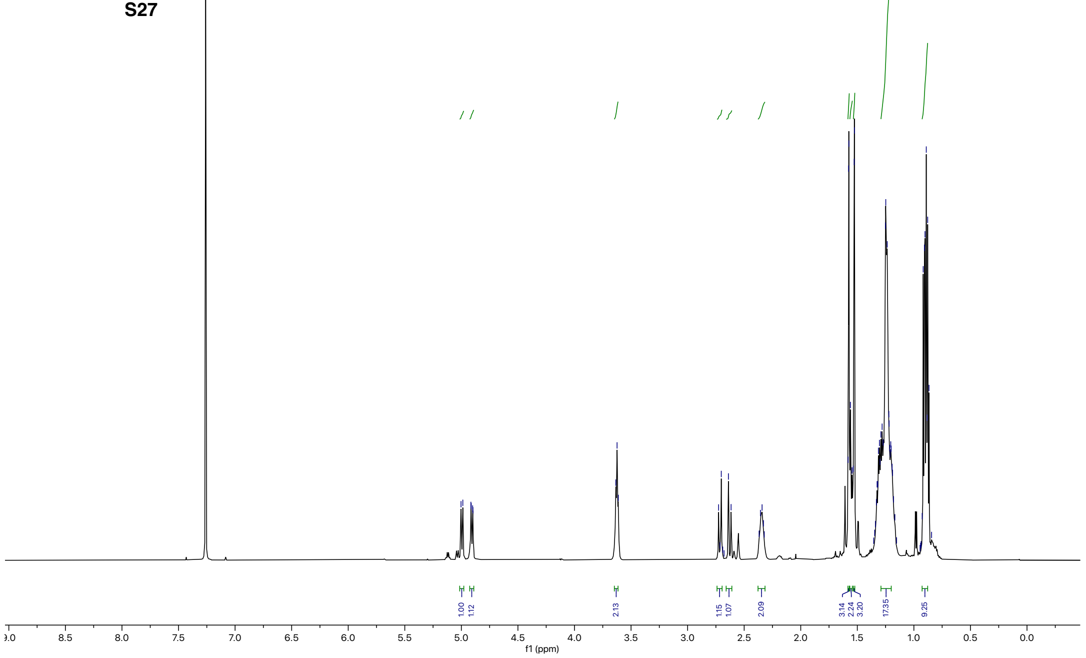

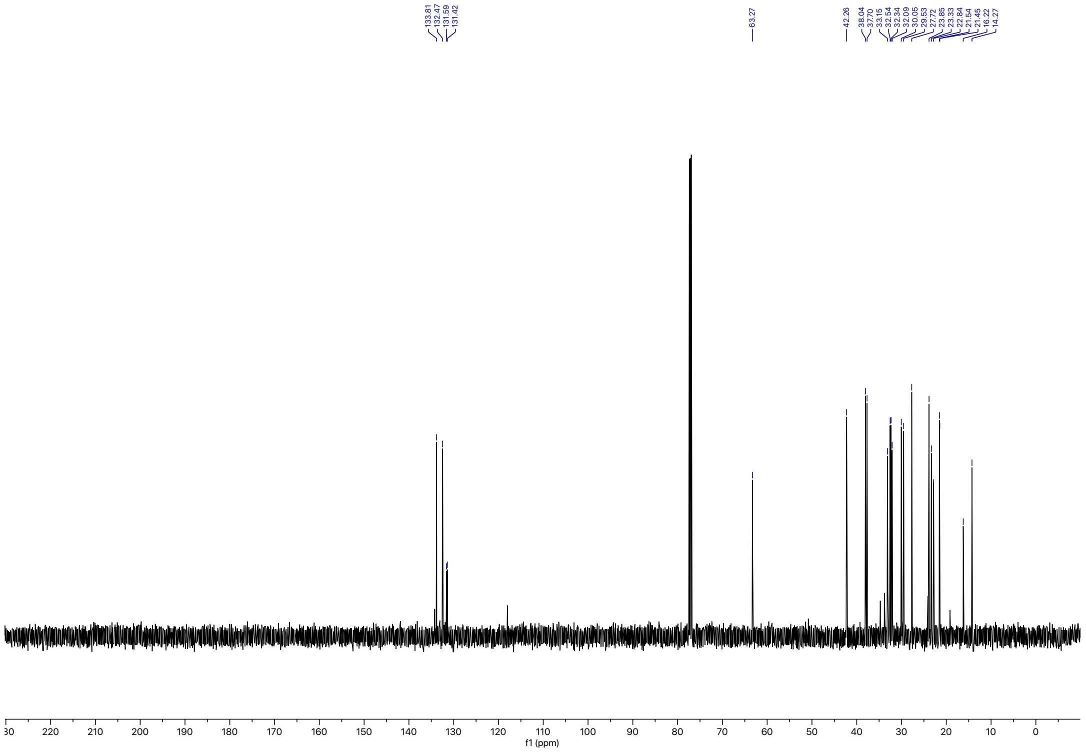

^{1}H NMR (600 MHz, $CDCl_3$) and ^{13}C NMR (150 MHz, $CDCl_3$) of **S28**

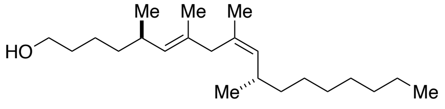

S28

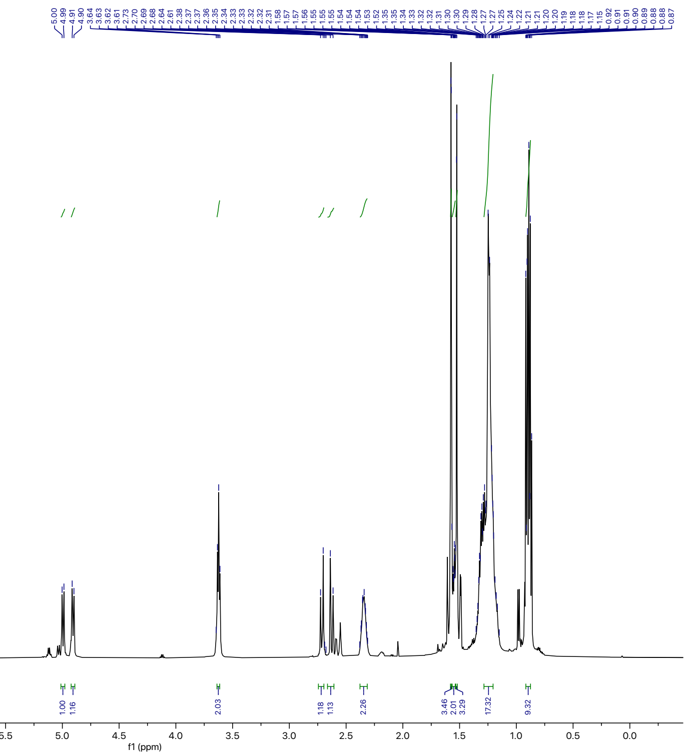

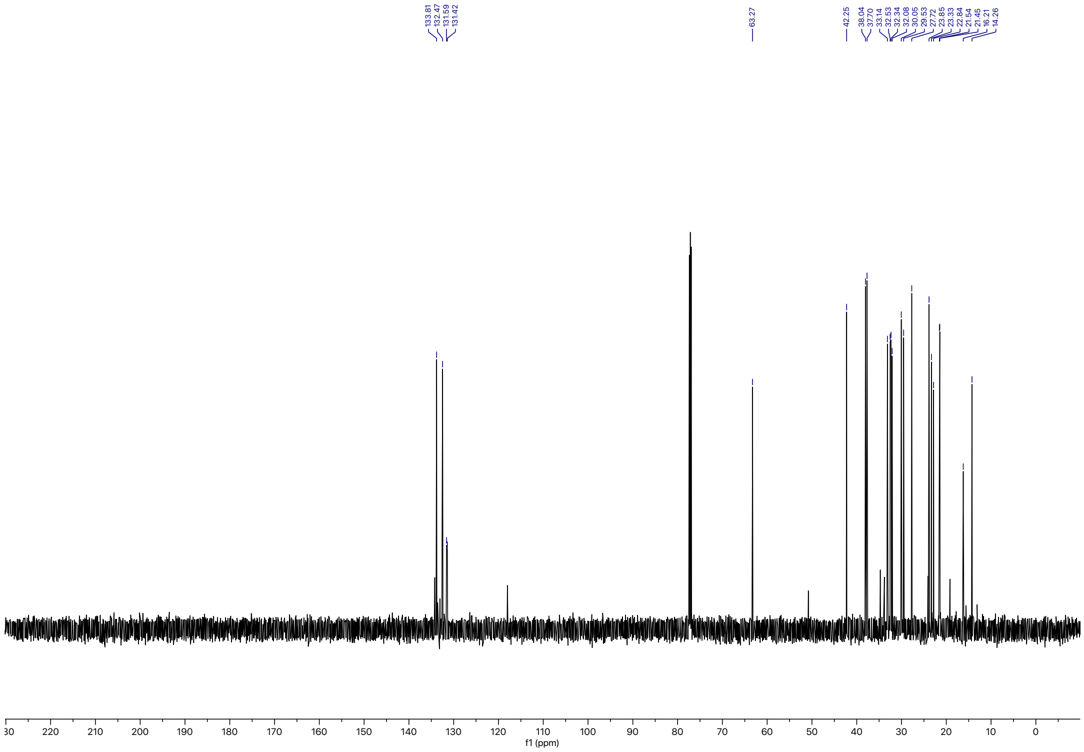

^{1}H NMR (600 MHz, $CDCl_3$) and ^{13}C NMR (150 MHz, $CDCl_3$) of **S29**

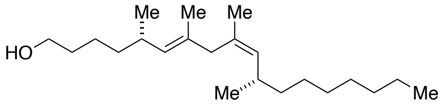

S29

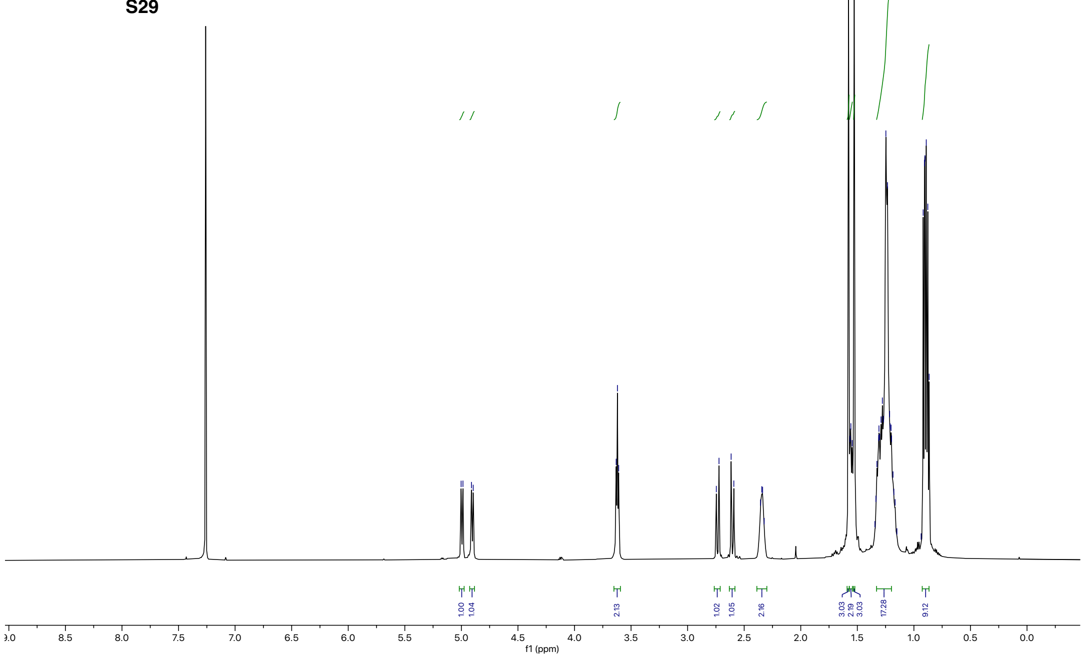

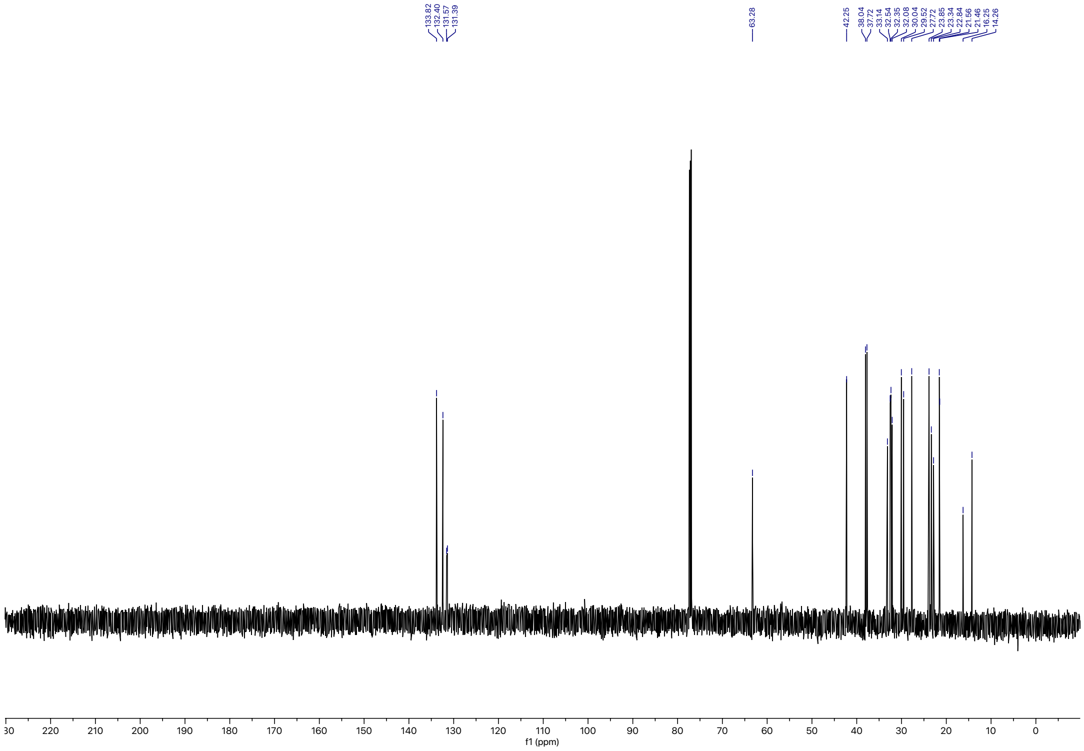

^{1}H NMR (600 MHz, $CDCl_3$) and ^{13}C NMR (150 MHz, $CDCl_3$) of **S30**

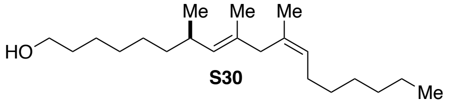

S30

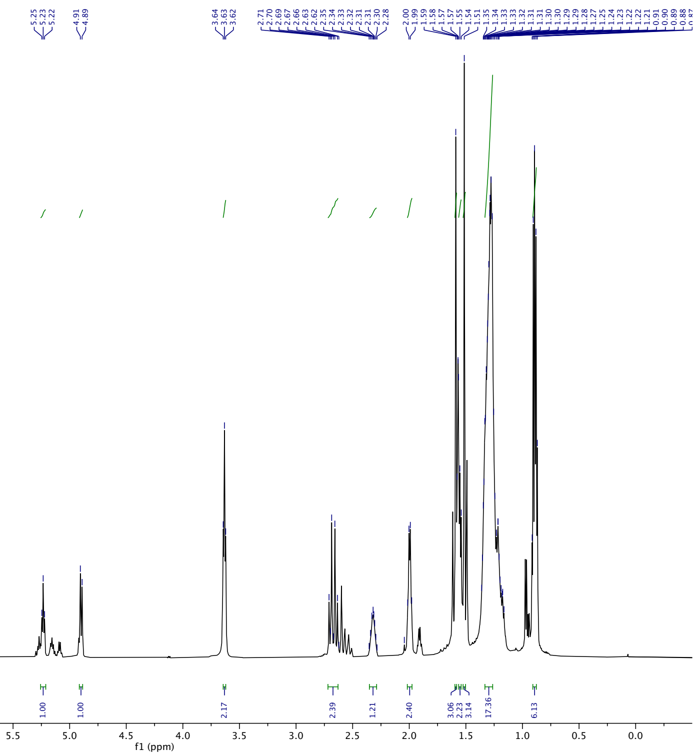

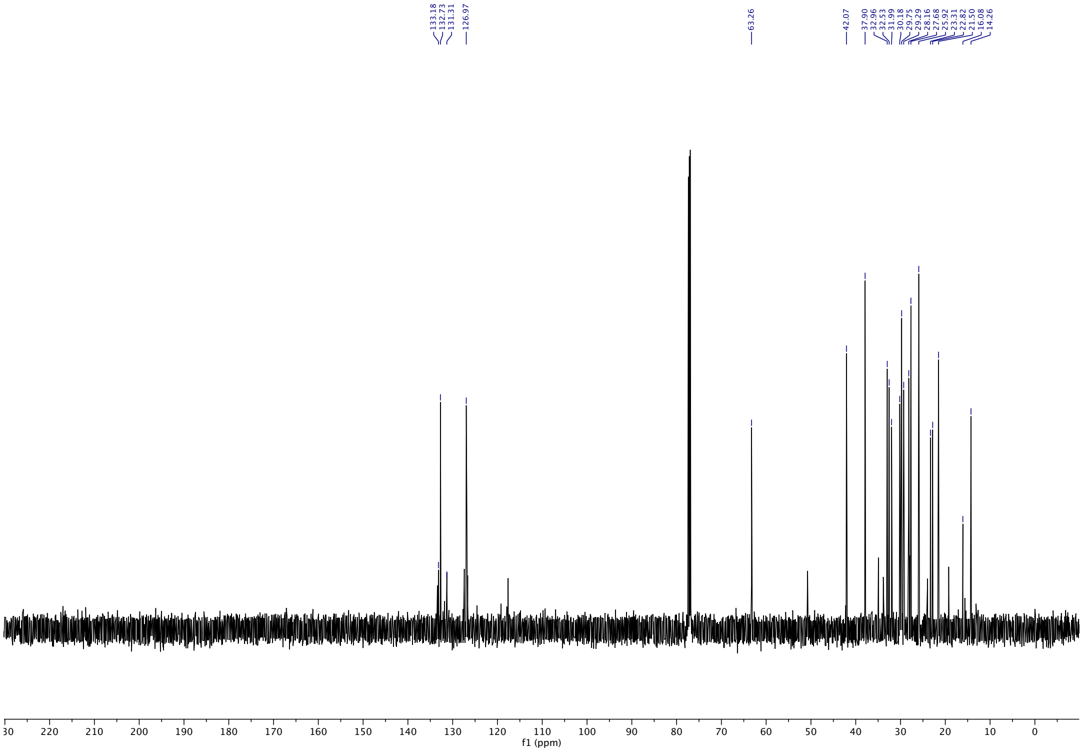

^{1}H NMR (600 MHz, $CDCl_3$) and ^{13}C NMR (150 MHz, $CDCl_3$) of **S31**

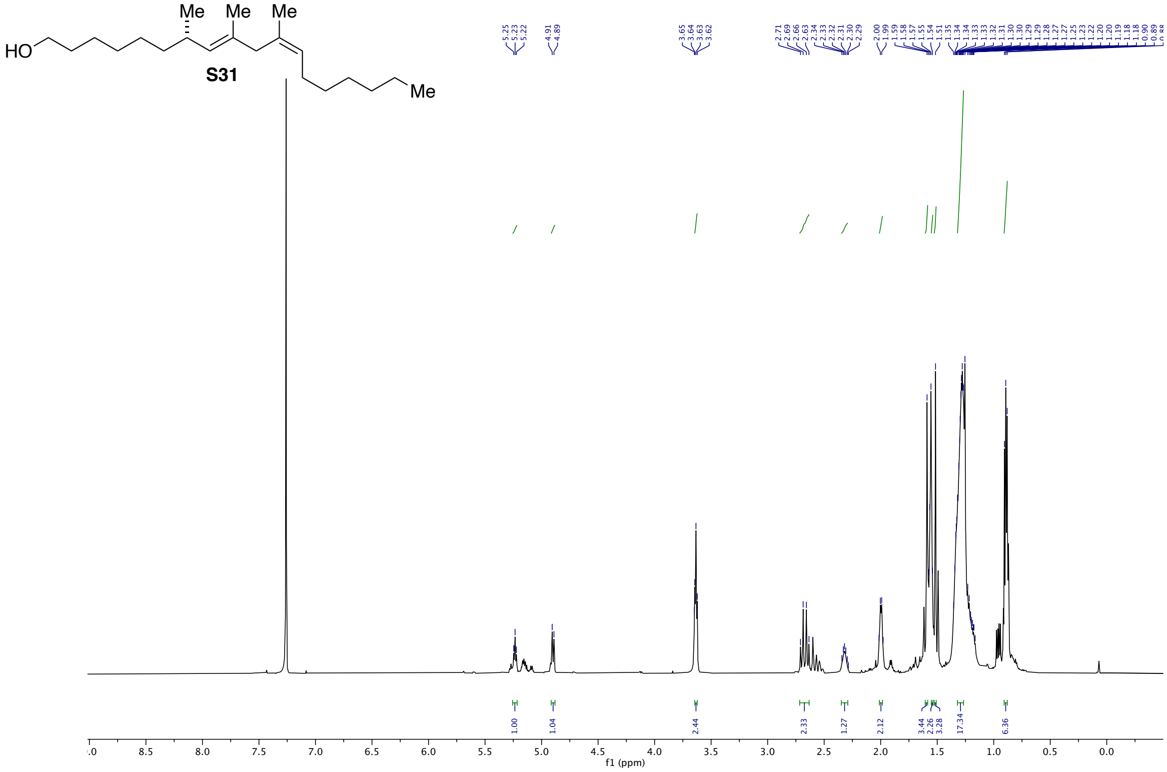

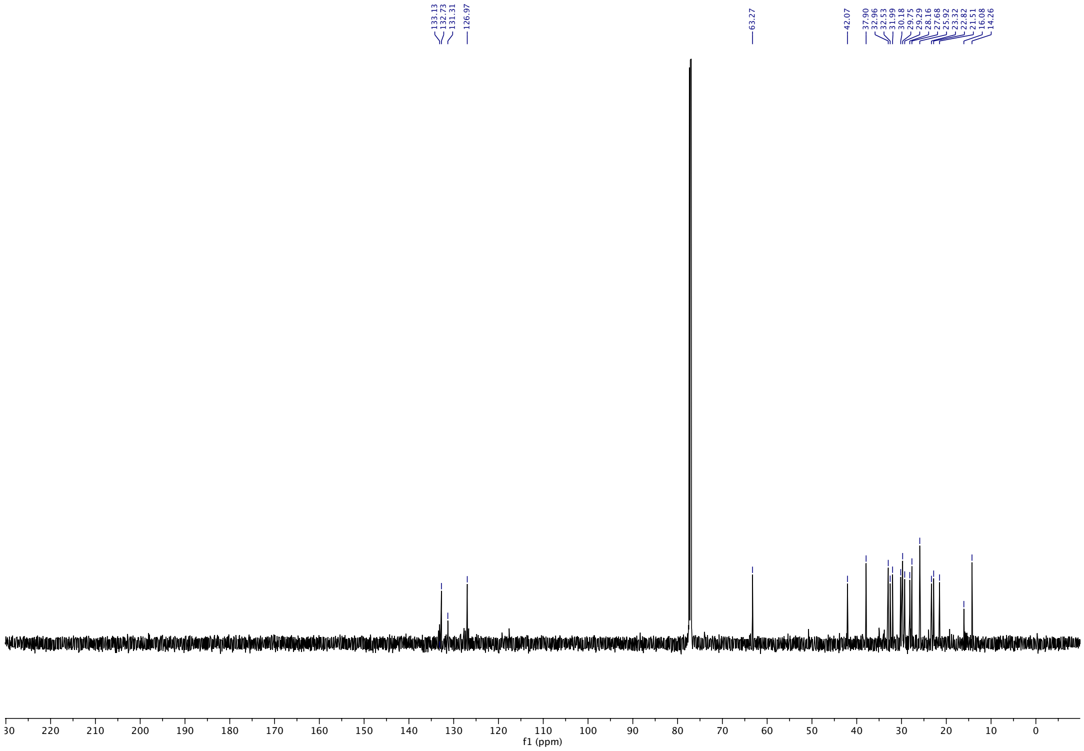

^{1}H NMR (600 MHz, $CDCl_3$) and ^{13}C NMR (150 MHz, $CDCl_3$) of **S32**

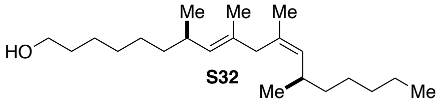

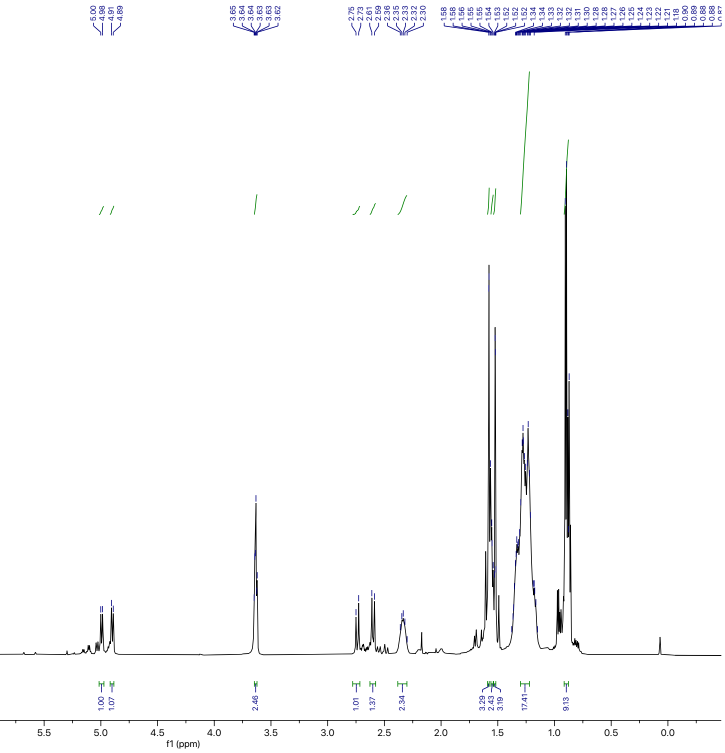

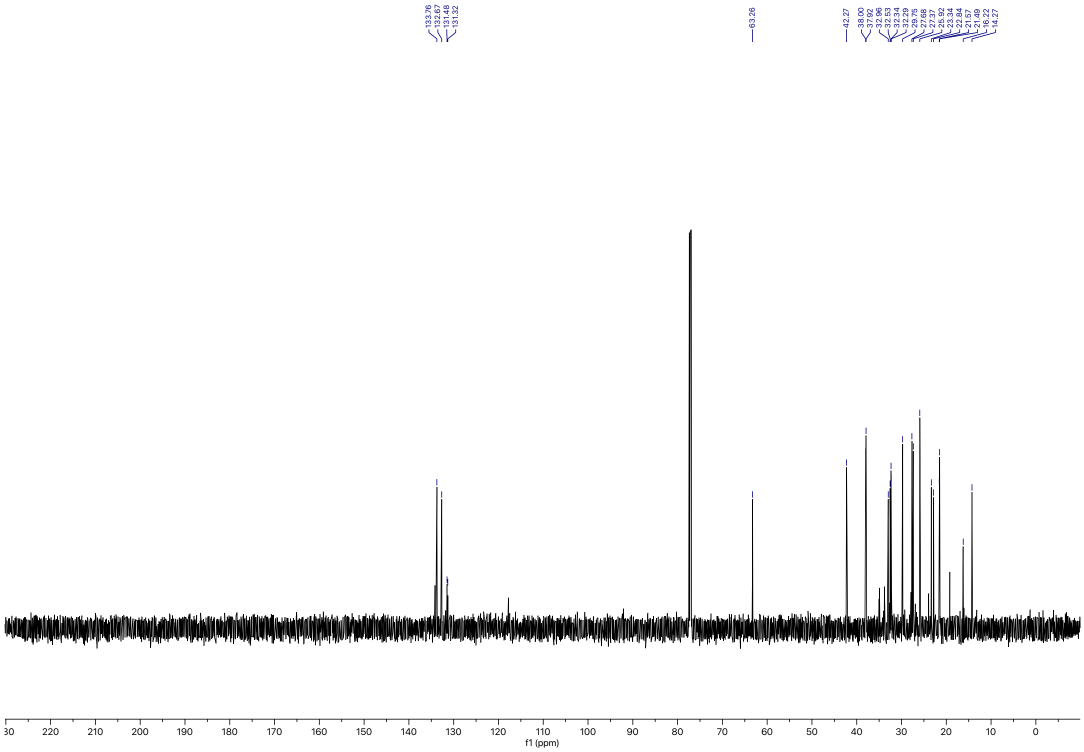

^{1}H NMR (600 MHz, $CDCl_3$) and ^{13}C NMR (150 MHz, $CDCl_3$) of **22**

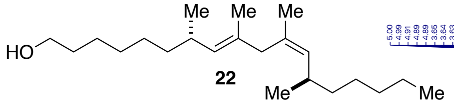

22

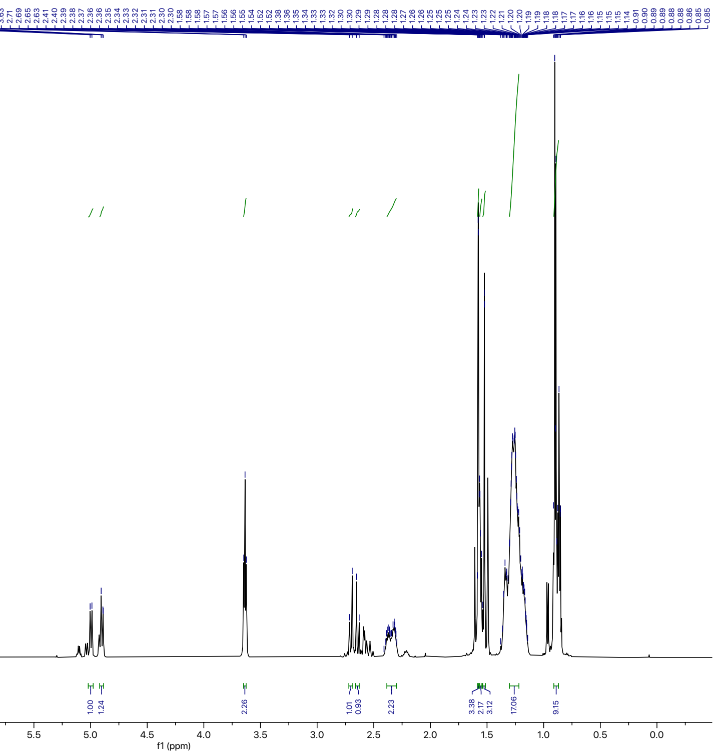

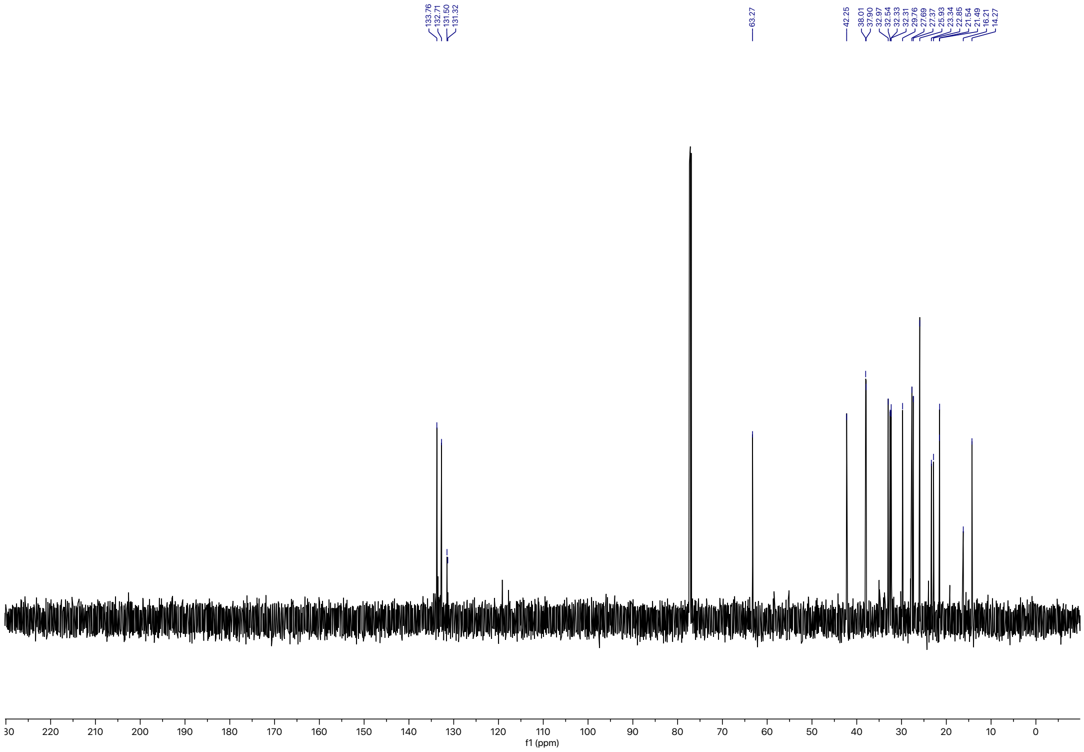

^1H NMR (600 MHz, $CDCl_3$) and ^{13}C NMR (150 MHz, $CDCl_3$) of **S33**

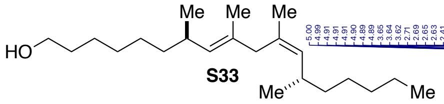

S33

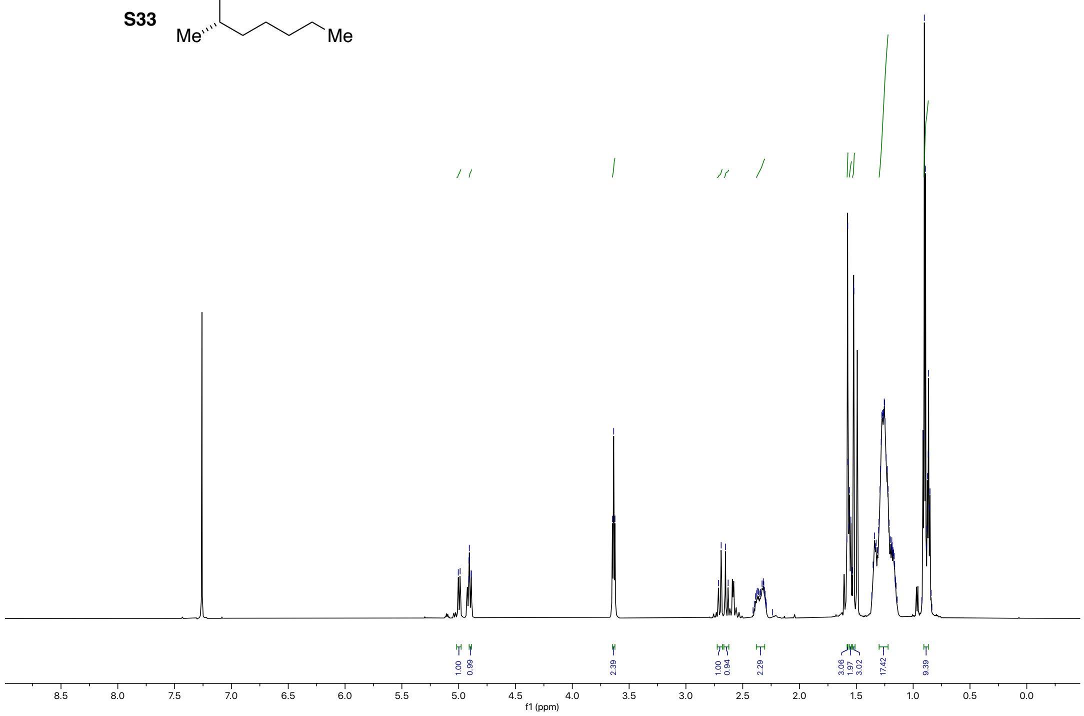

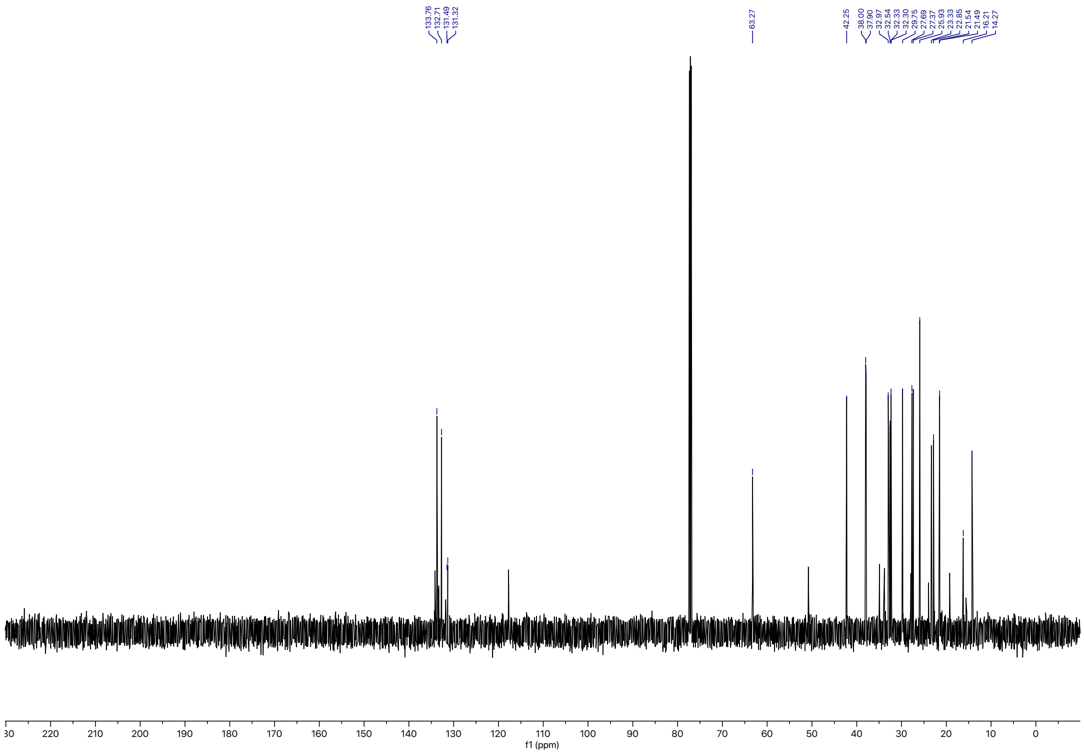

^{1}H NMR (600 MHz, $CDCl_3$) and ^{13}C NMR (150 MHz, $CDCl_3$) of **S34**

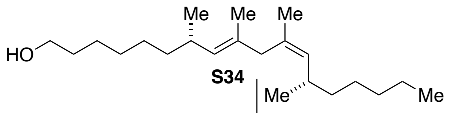

S34

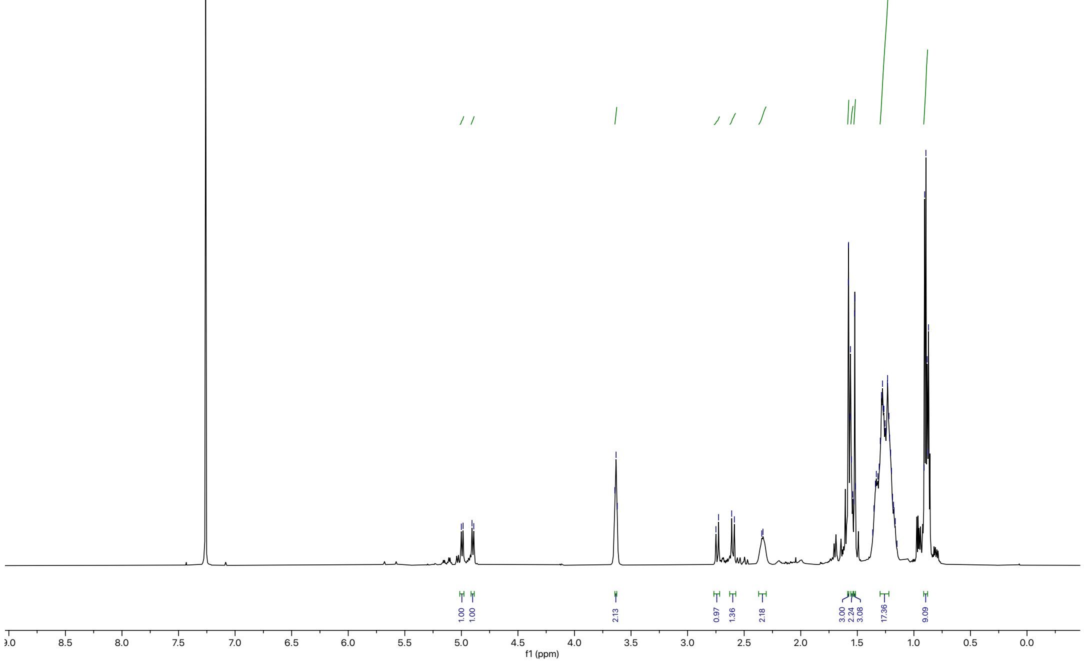

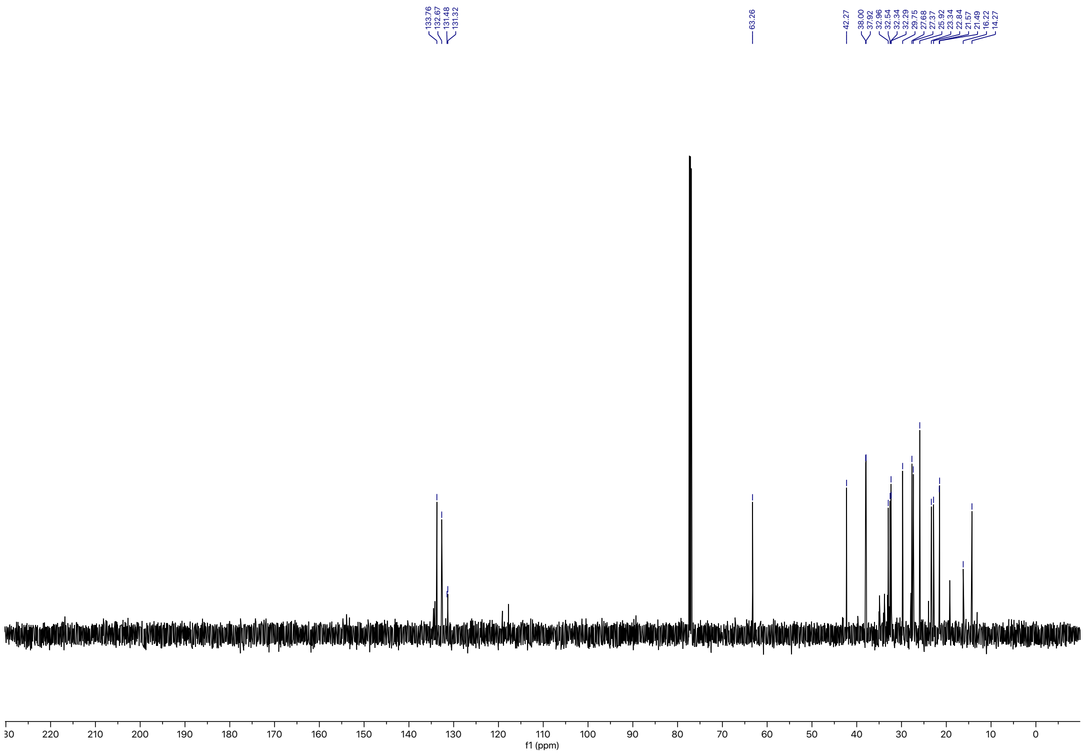

^{1}H NMR (600 MHz, $CDCl_3$) and ^{13}C NMR (150 MHz, $CDCl_3$) of **S35**

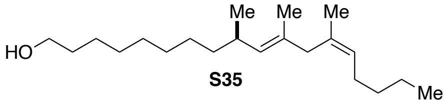

S35

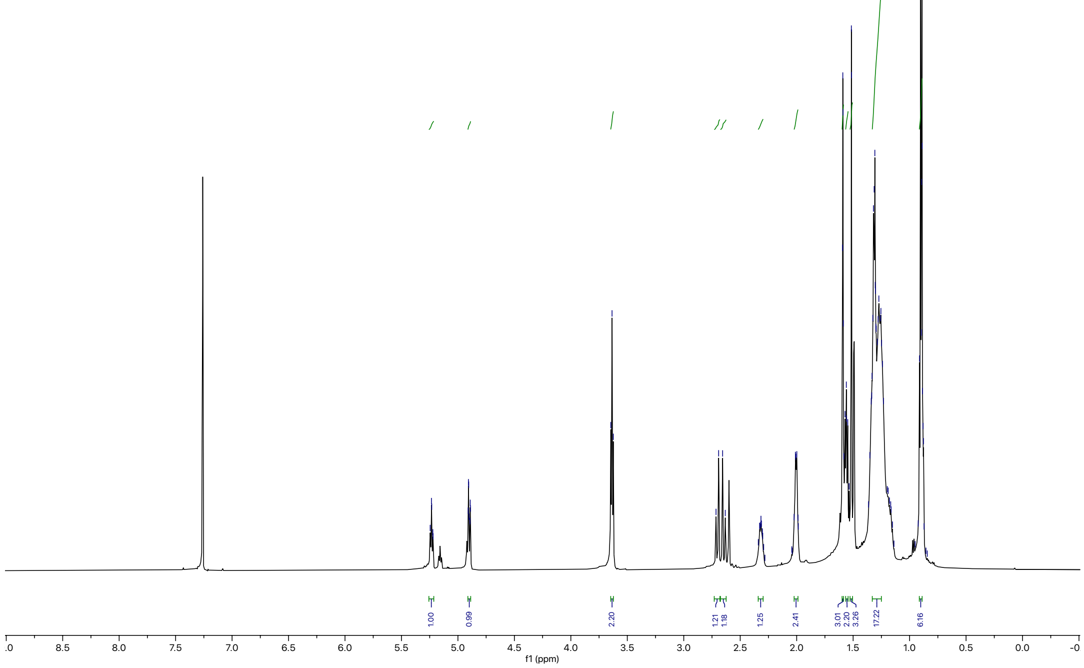

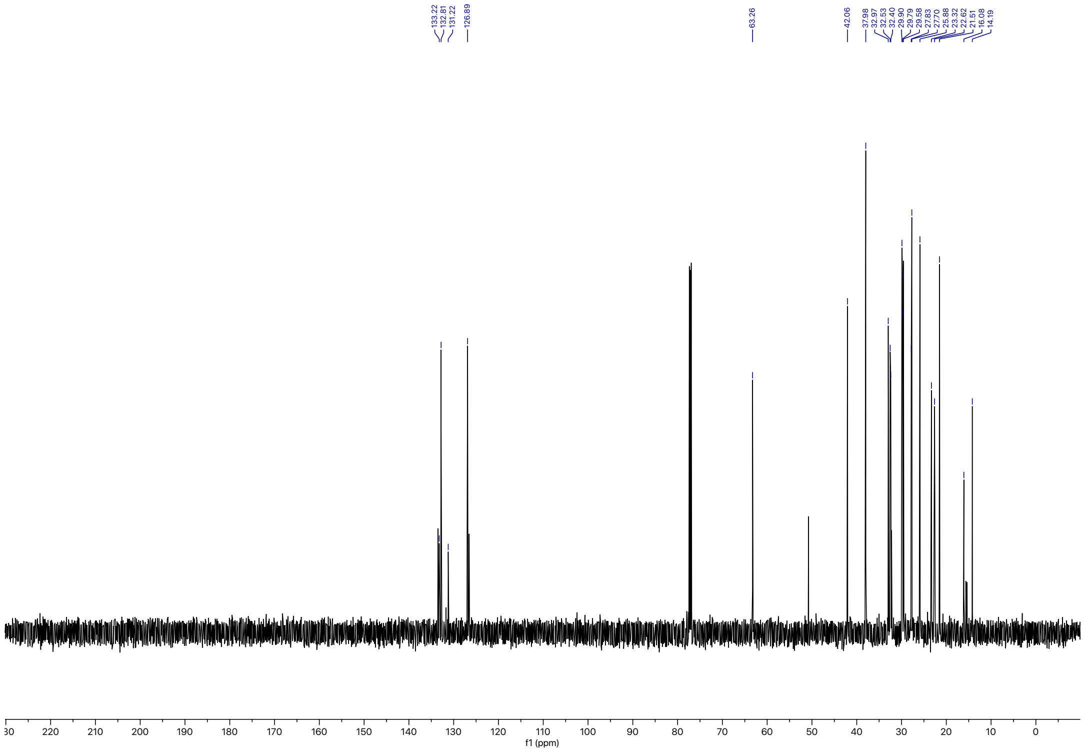

^{1}H NMR (600 MHz, $CDCl_3$) and ^{13}C NMR (150 MHz, $CDCl_3$) of **23**

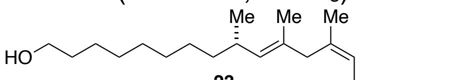

23

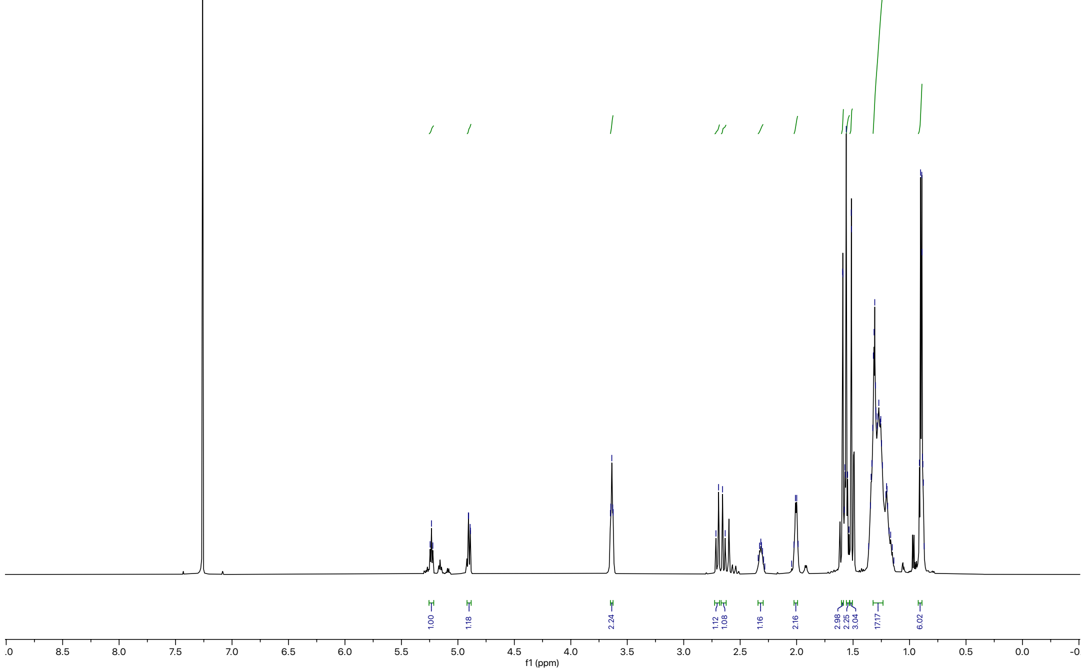

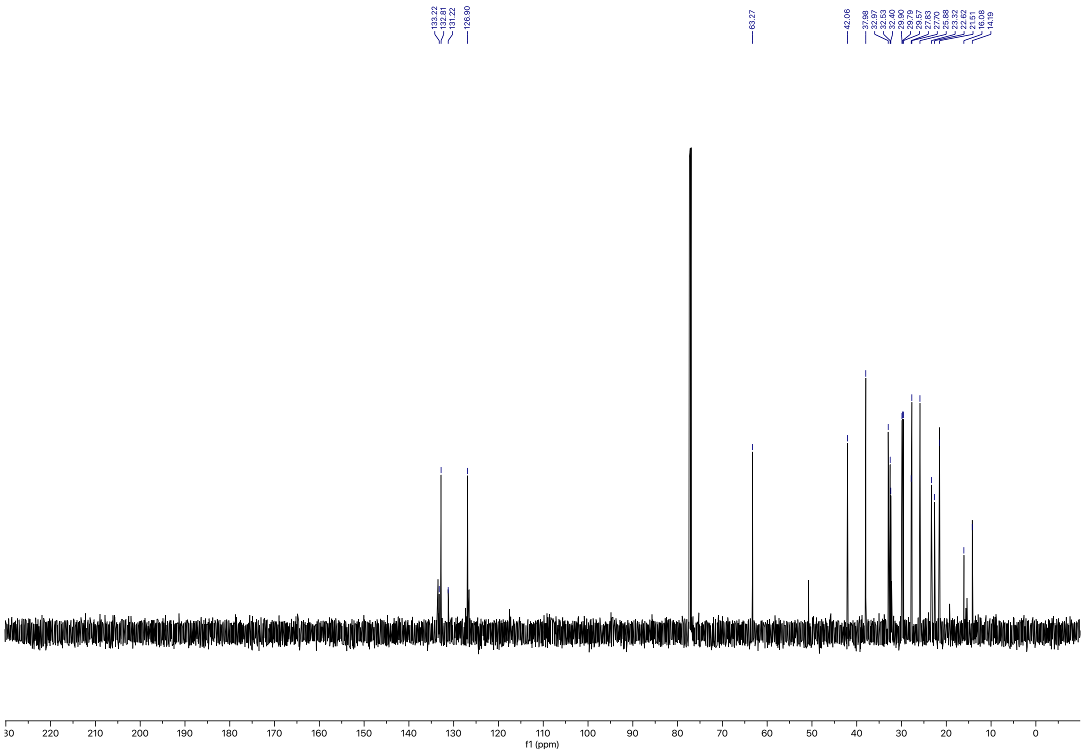

¹H NMR (600 MHz, CDCl$_3$) and ¹³C NMR (150 MHz, CDCl$_3$) of **S36**

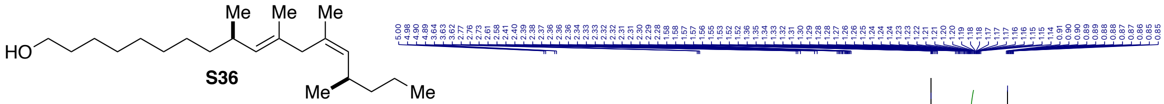

S36

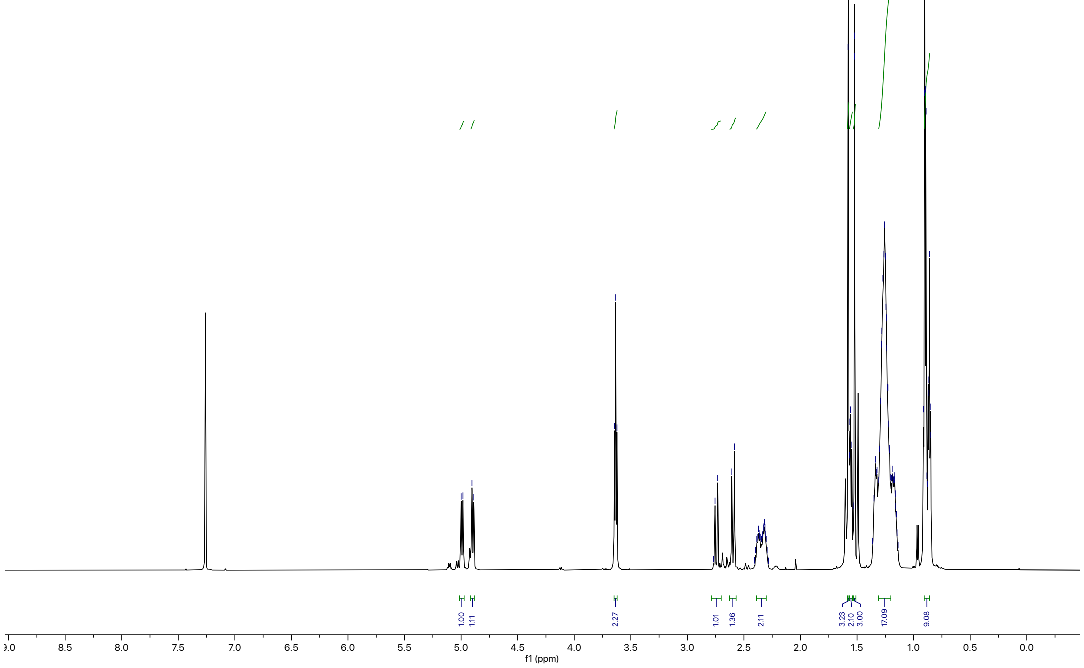

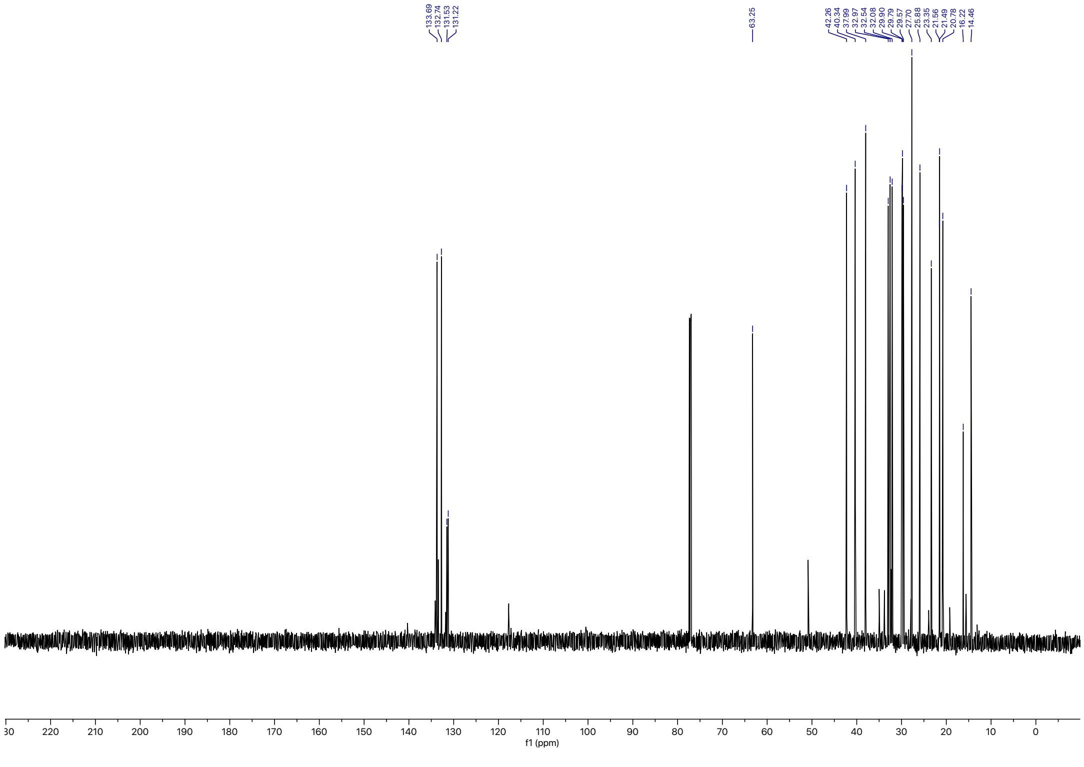

^{1}H NMR (600 MHz, $CDCl_3$) and ^{13}C NMR (150 MHz, $CDCl_3$) of **S37**

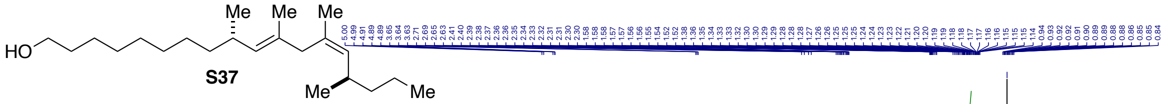

S37

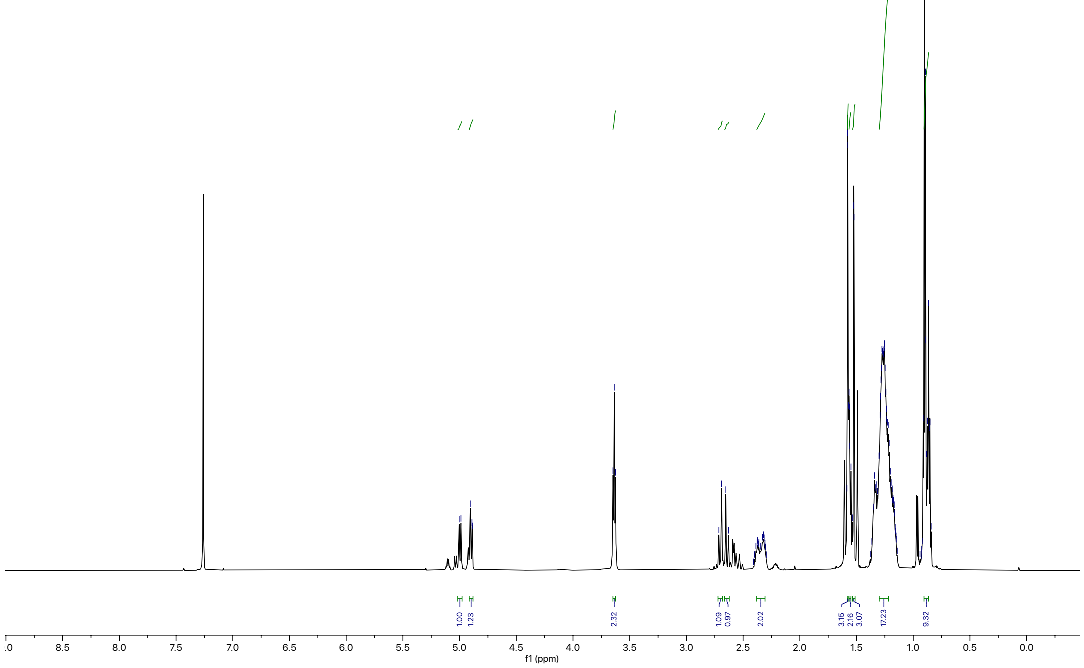

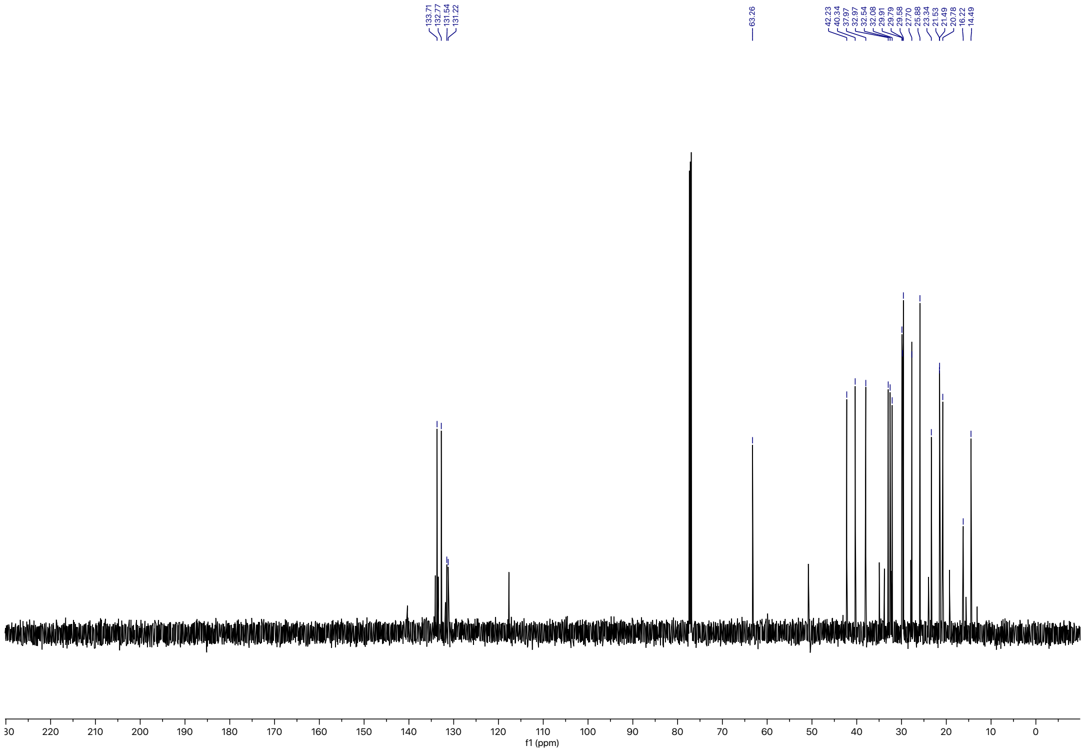

^{1}H NMR (600 MHz, $CDCl_3$) and ^{13}C NMR (150 MHz, $CDCl_3$) of **S38**

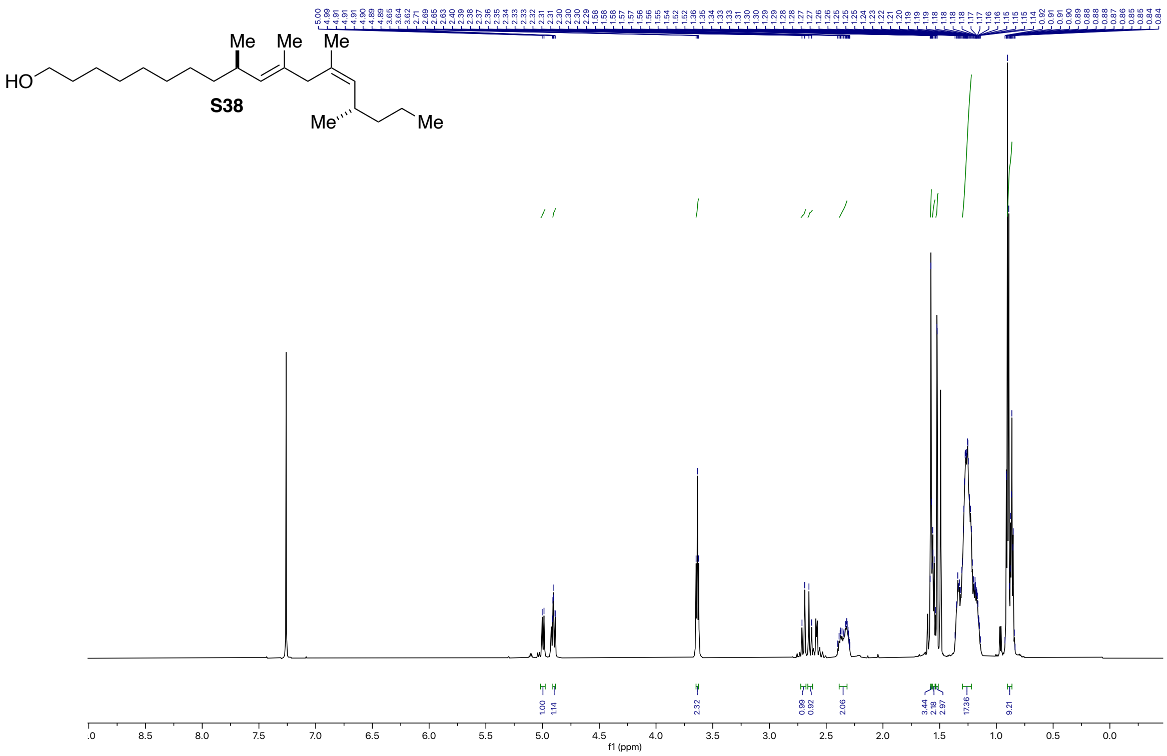

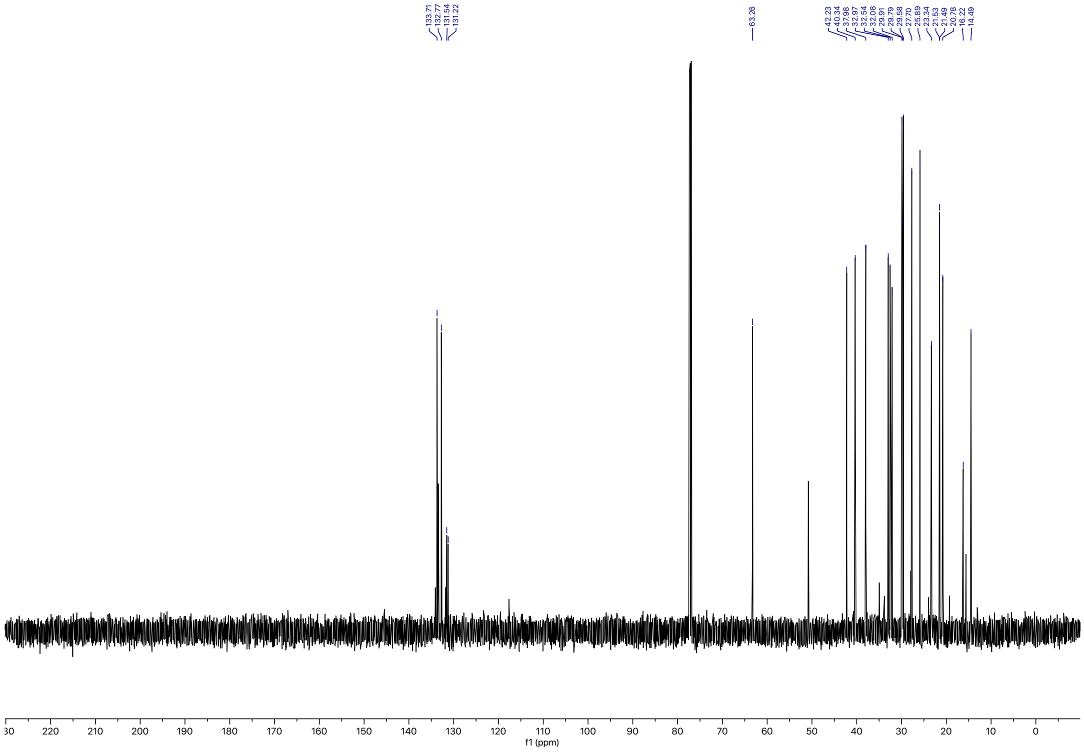

^{1}H NMR (600 MHz, $CDCl_3$) and ^{13}C NMR (150 MHz, $CDCl_3$) of **S39**

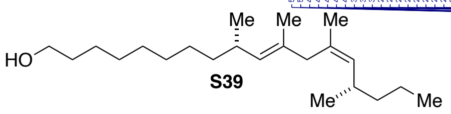

S39

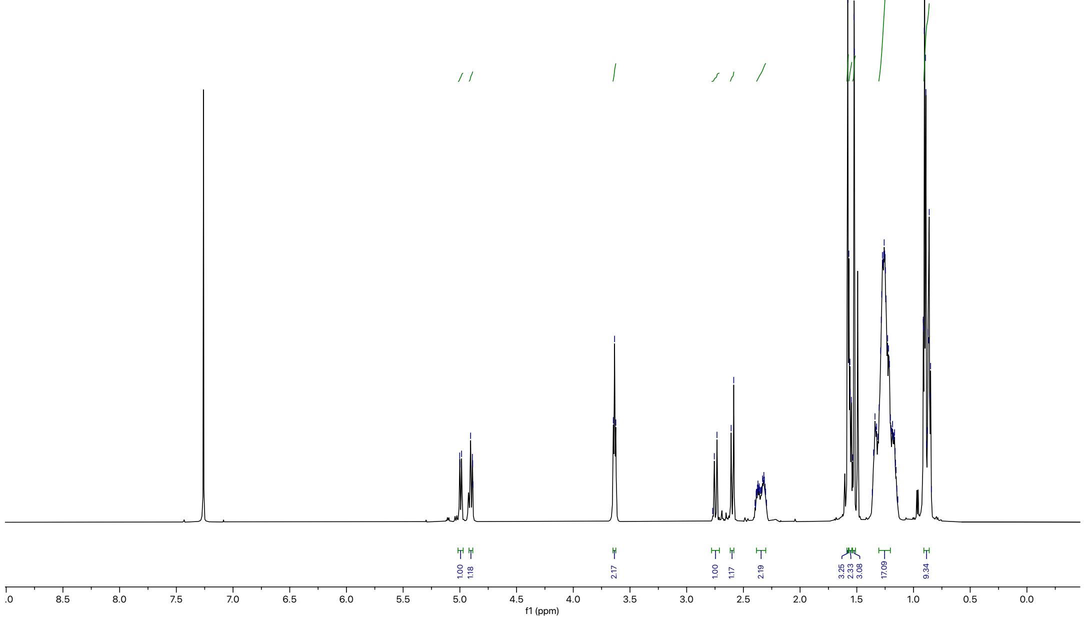

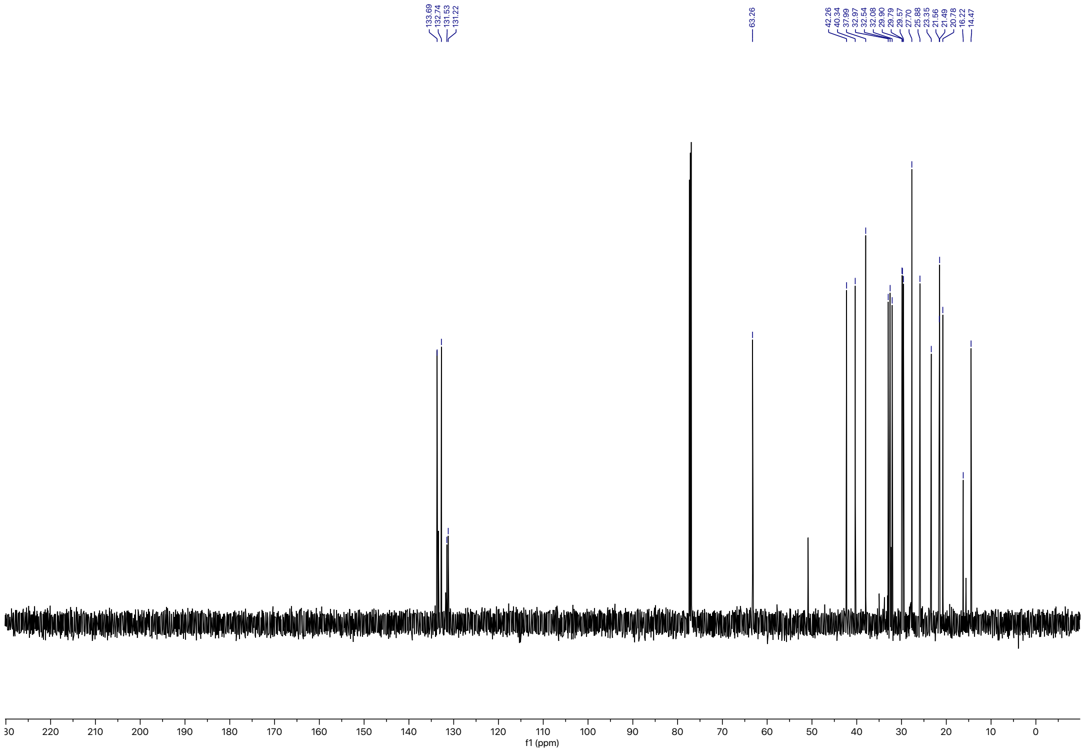

^{1}H NMR (600 MHz, $CDCl_3$) and ^{13}C NMR (150 MHz, $CDCl_3$) of **1-A***

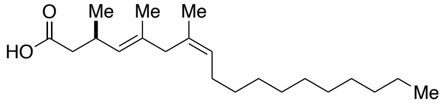

1-A

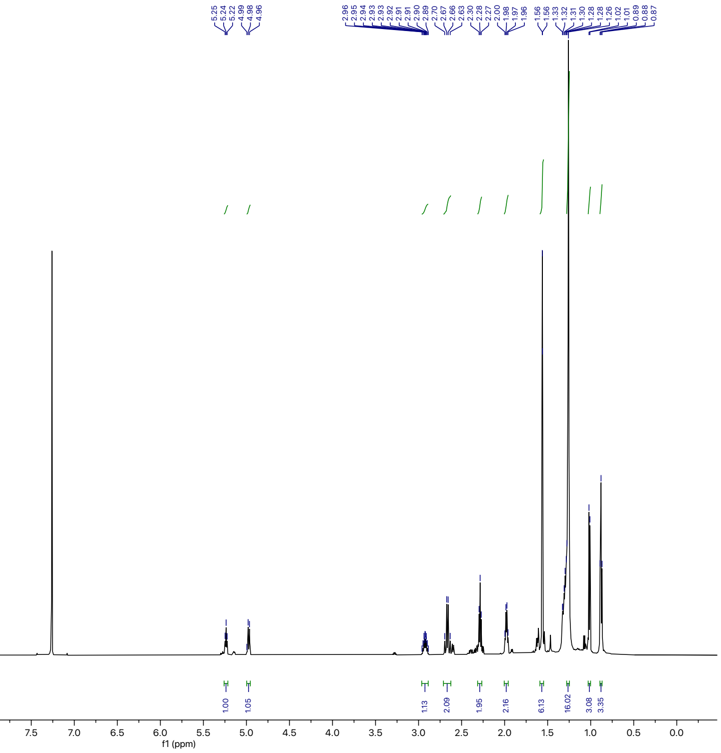

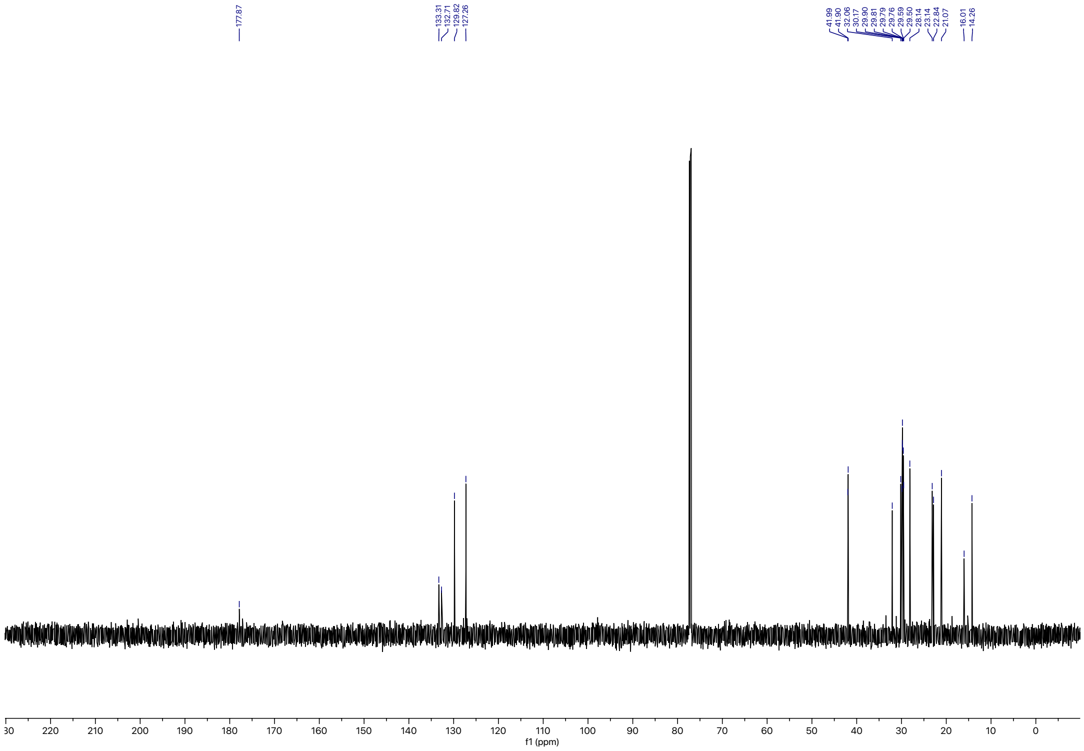

---

* All carboxylic acid NMRs are of the authentic samples submitted for biological testing.

^{1}H NMR (600 MHz, $CDCl_3$) and ^{13}C NMR (150 MHz, $CDCl_3$) of **1-B**

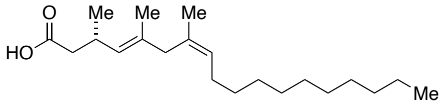

1-B

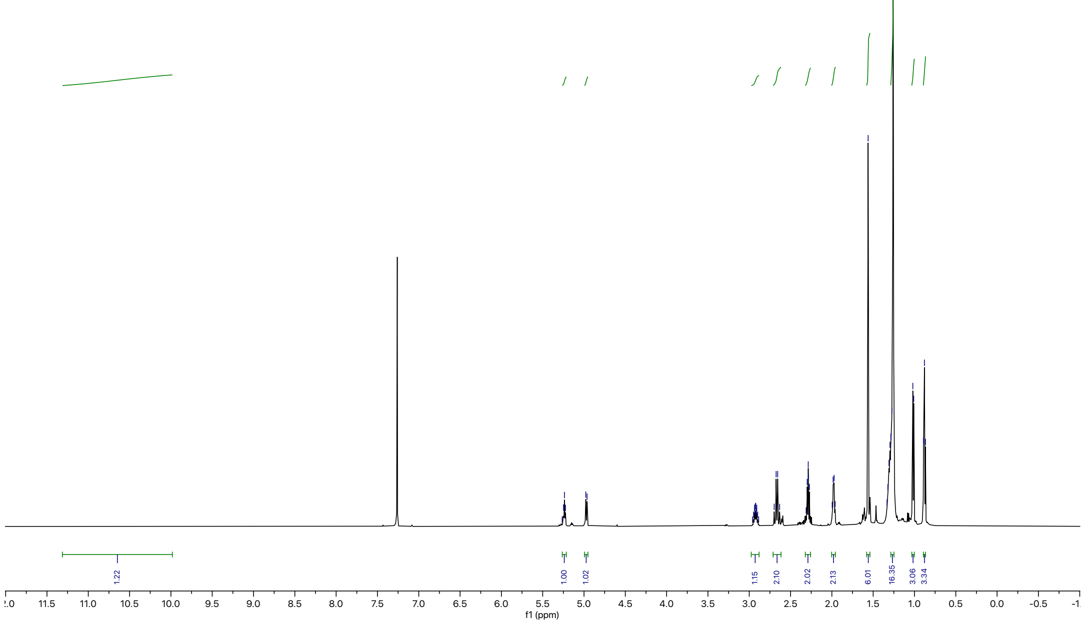

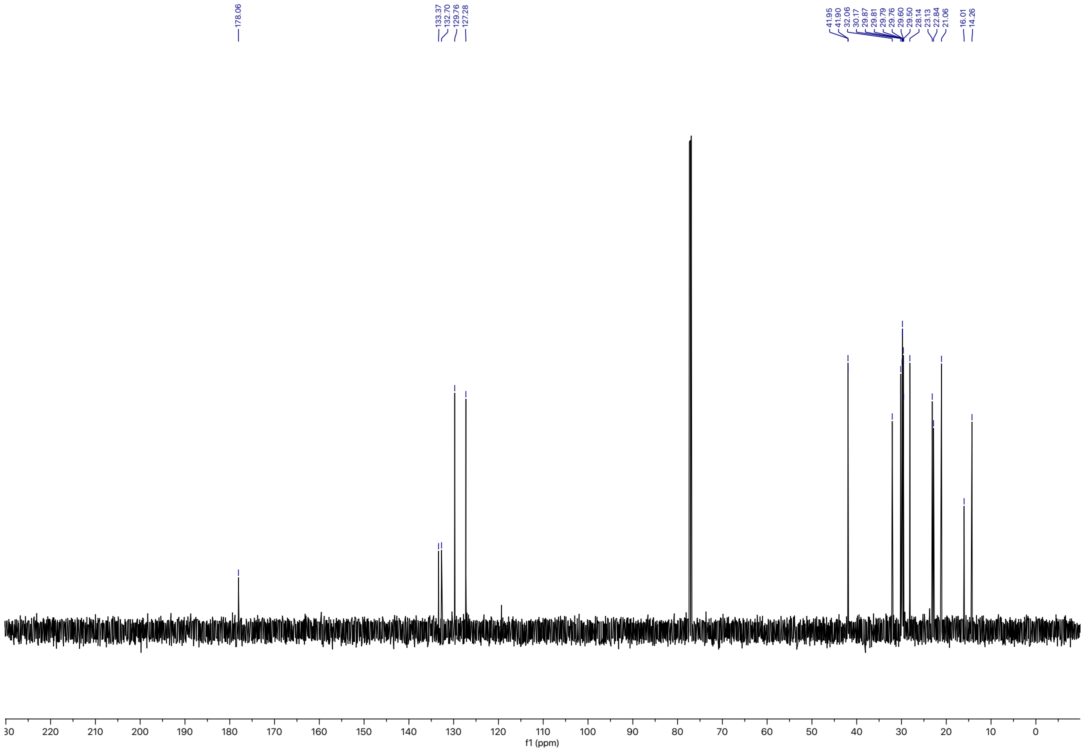

^{1}H NMR (600 MHz, $CDCl_3$) and ^{13}C NMR (150 MHz, $CDCl_3$) of **1-C**

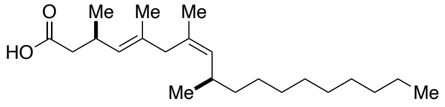

1-C

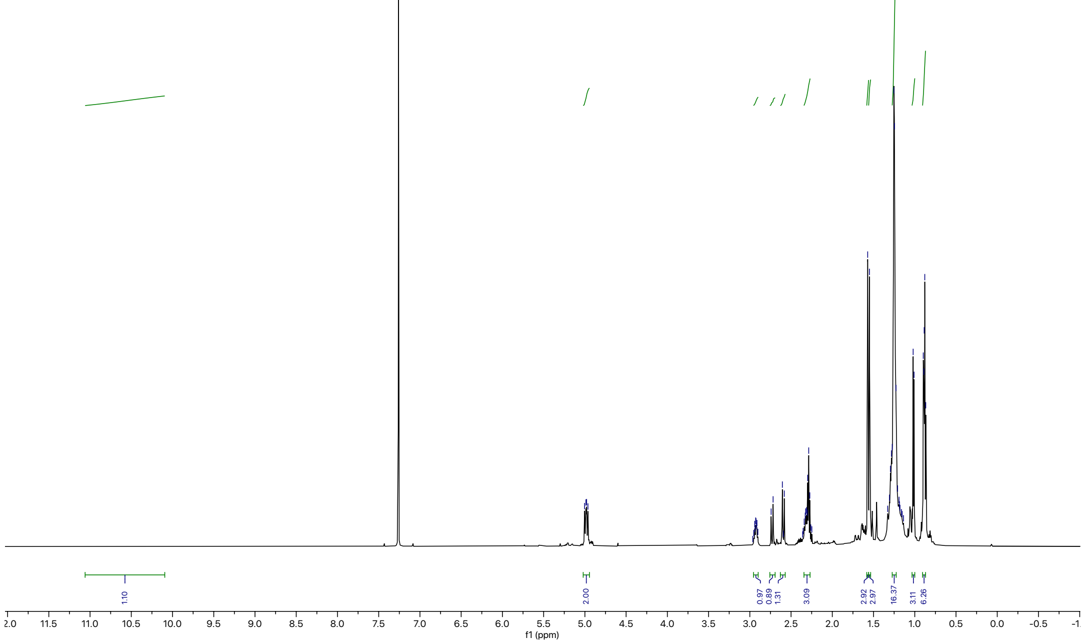

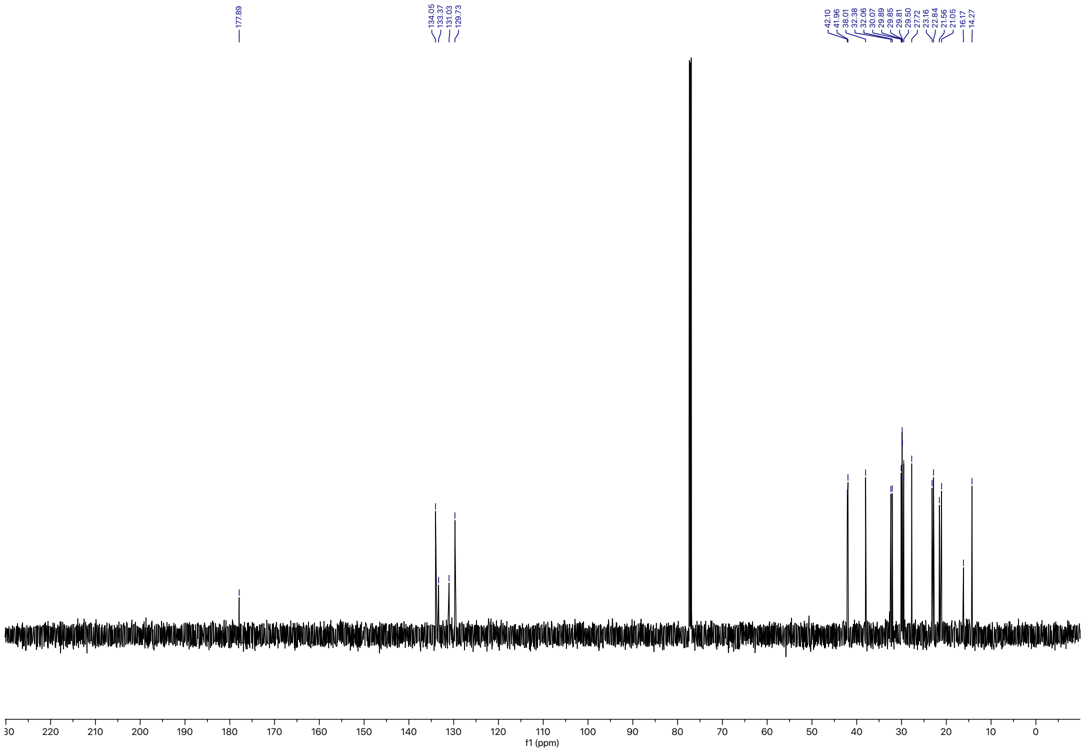

^{1}H NMR (600 MHz, $CDCl_3$) and ^{13}C NMR (150 MHz, $CDCl_3$) of **1-D** (^{13}C signal at 29.8 ppm not selectable via NMR software)

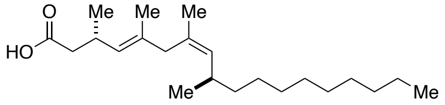

1-D

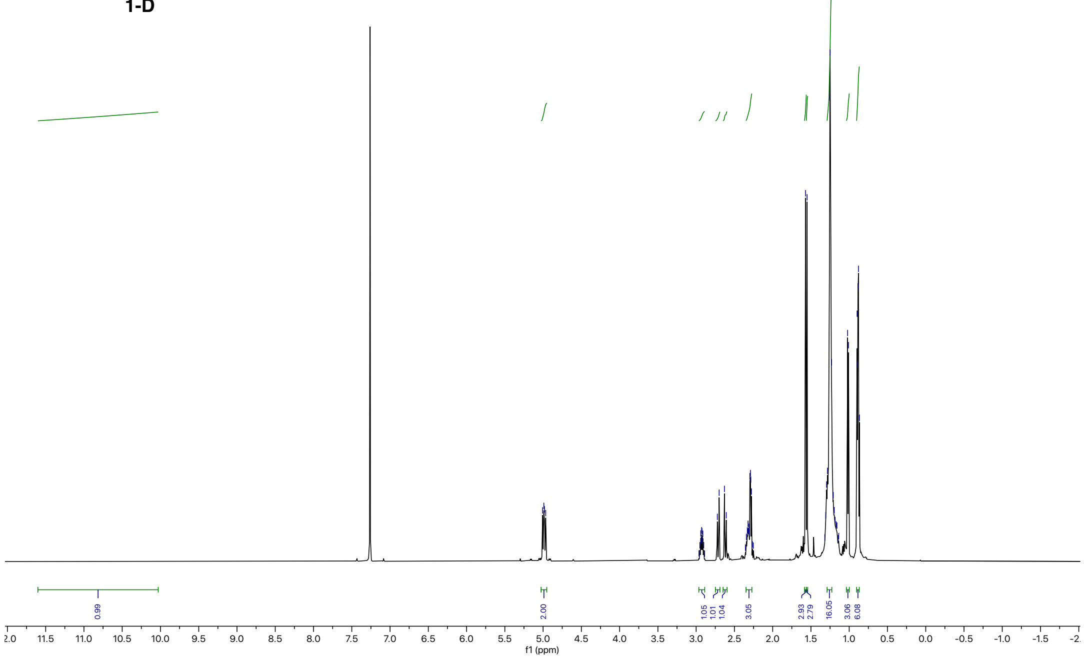

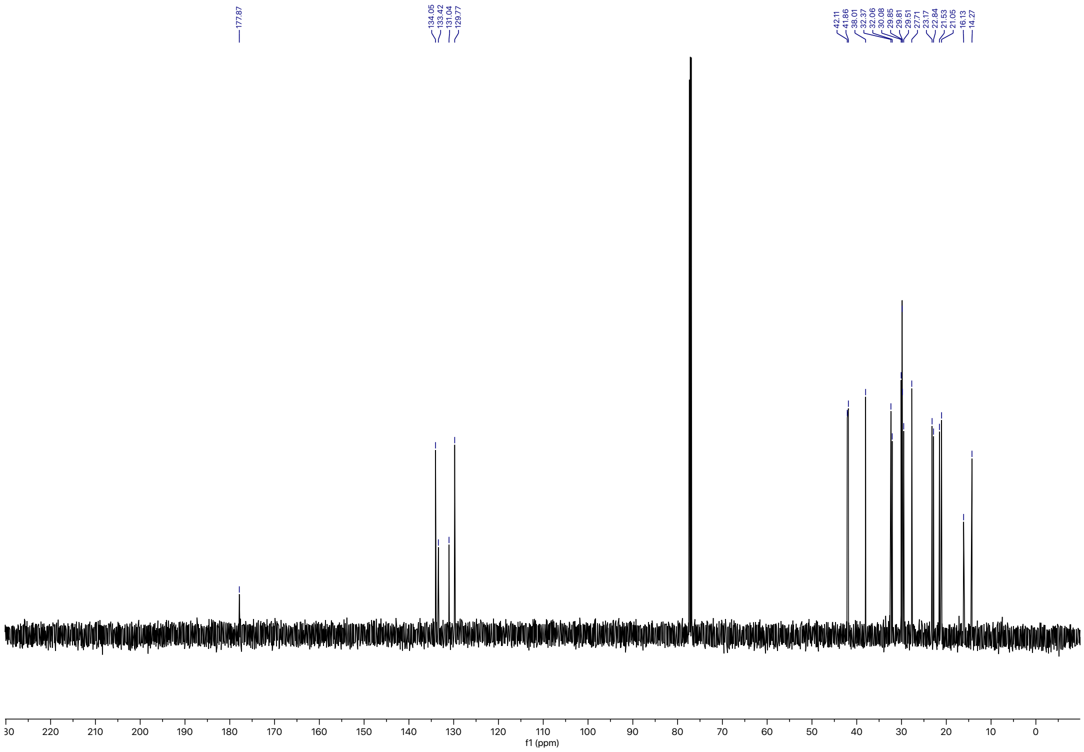

^{1}H NMR (600 MHz, $CDCl_3$) and ^{13}C NMR (150 MHz, $CDCl_3$) of **1-E**

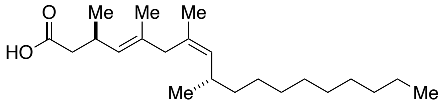

1-E

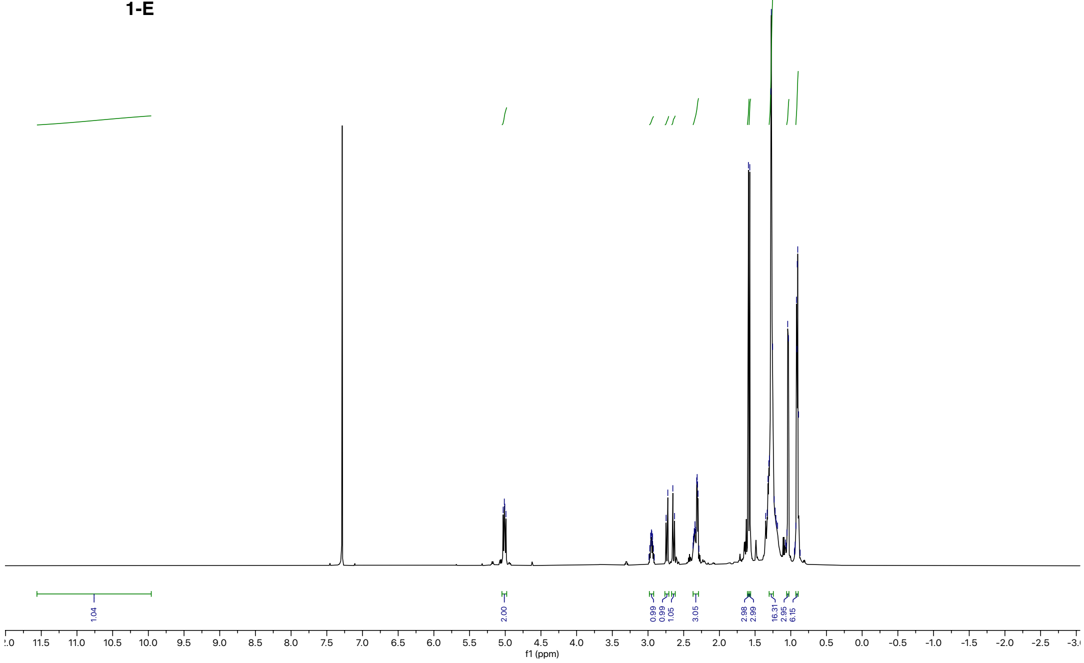

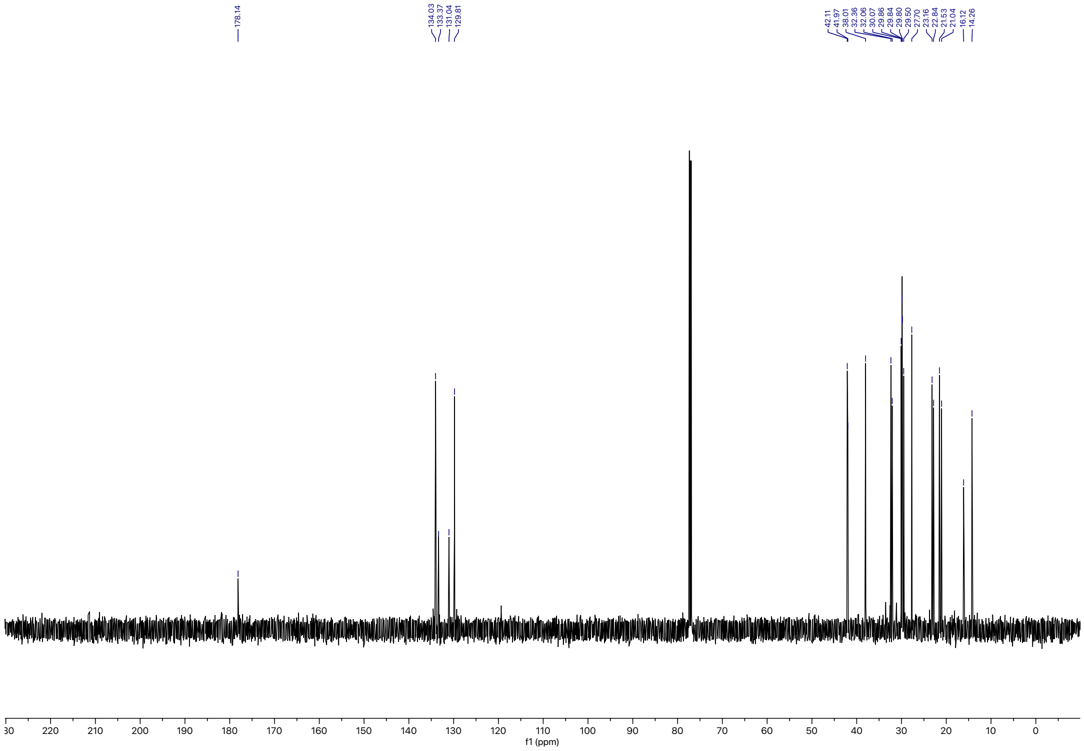

^1H NMR (600 MHz, $CDCl_3$) and ^{13}C NMR (150 MHz, $CDCl_3$) of **1-F** (^{13}C signal at 42.0 ppm not selectable via NMR software)

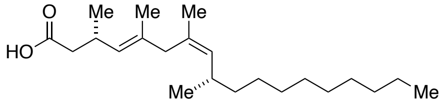

1-F

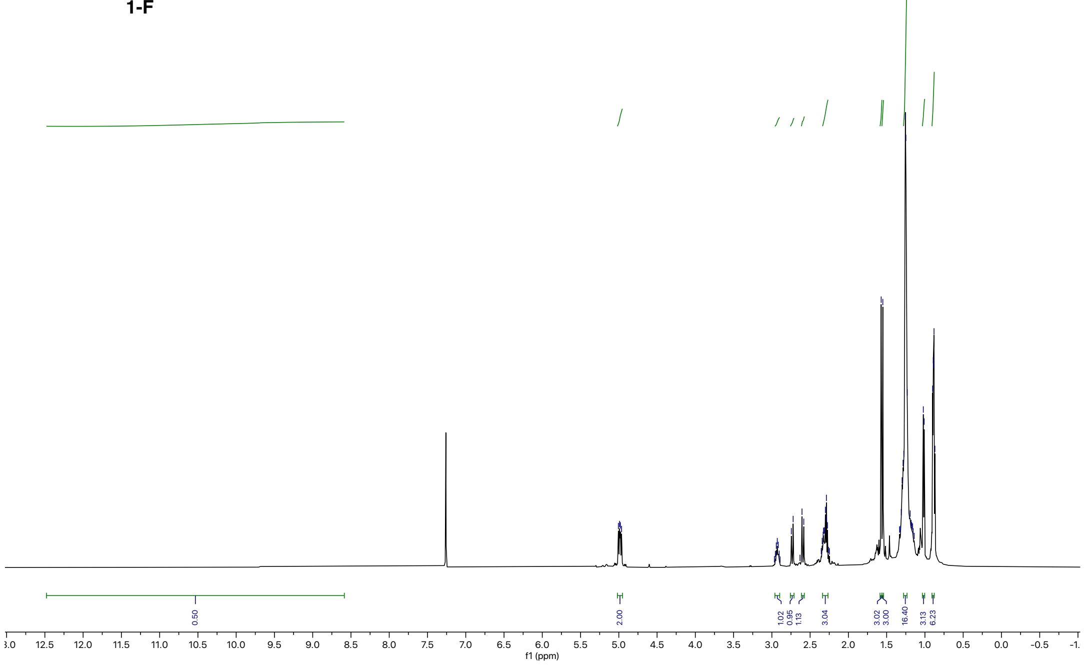

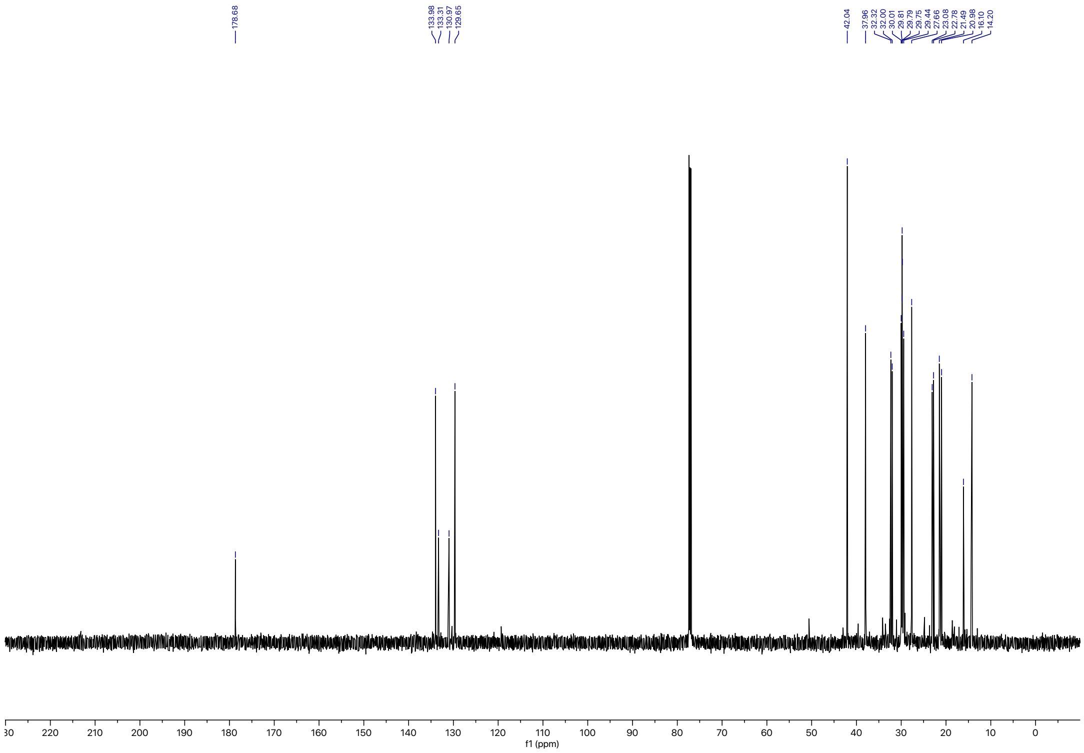

^{1}H NMR (600 MHz, $CDCl_3$) and ^{13}C NMR (150 MHz, $CDCl_3$) of **2-A**

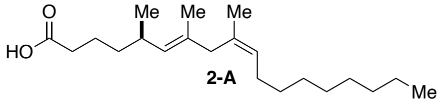

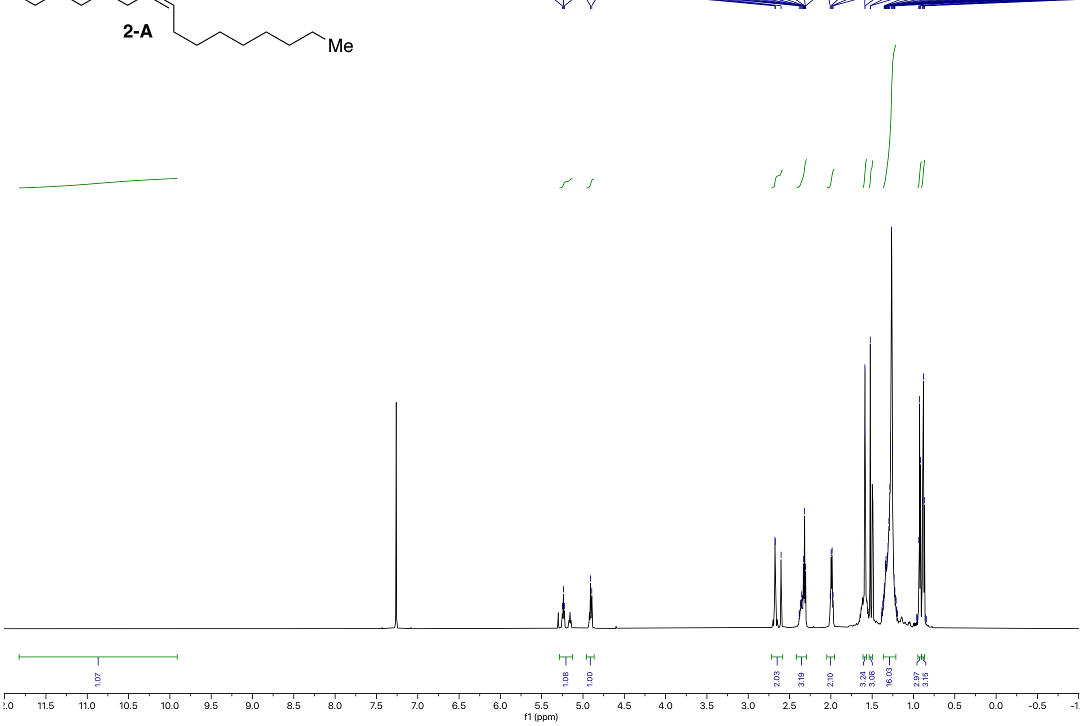

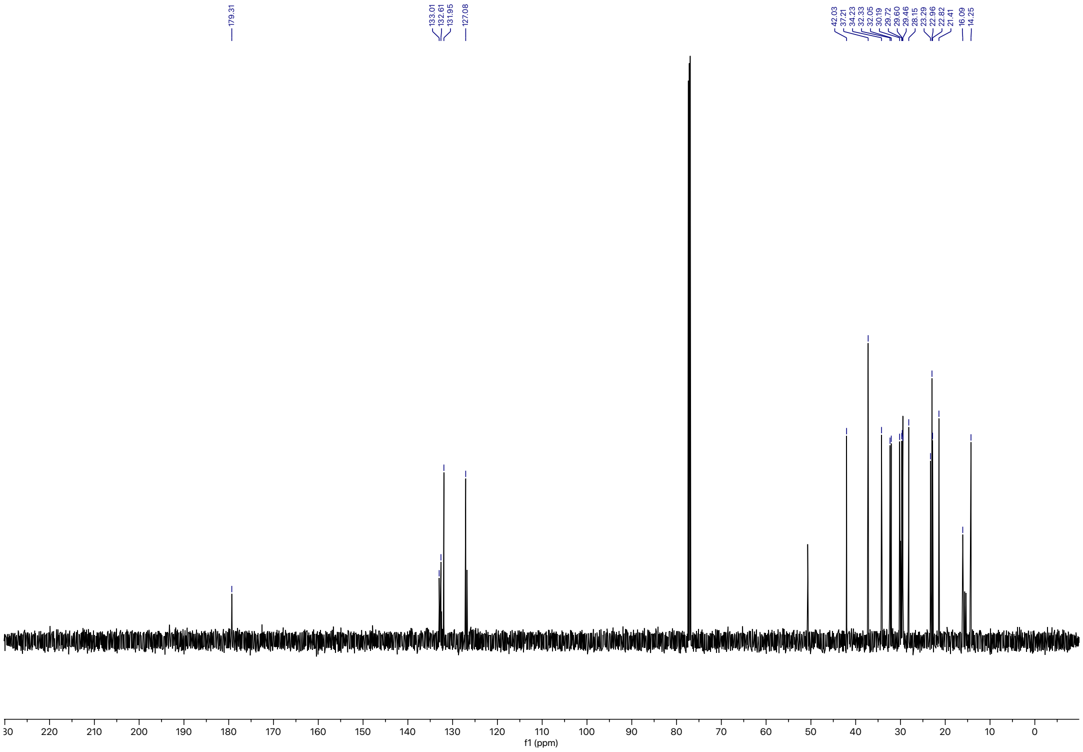

^1H NMR (600 MHz, $CDCl_3$) and ^{13}C NMR (150 MHz, $CDCl_3$) of **2-B**

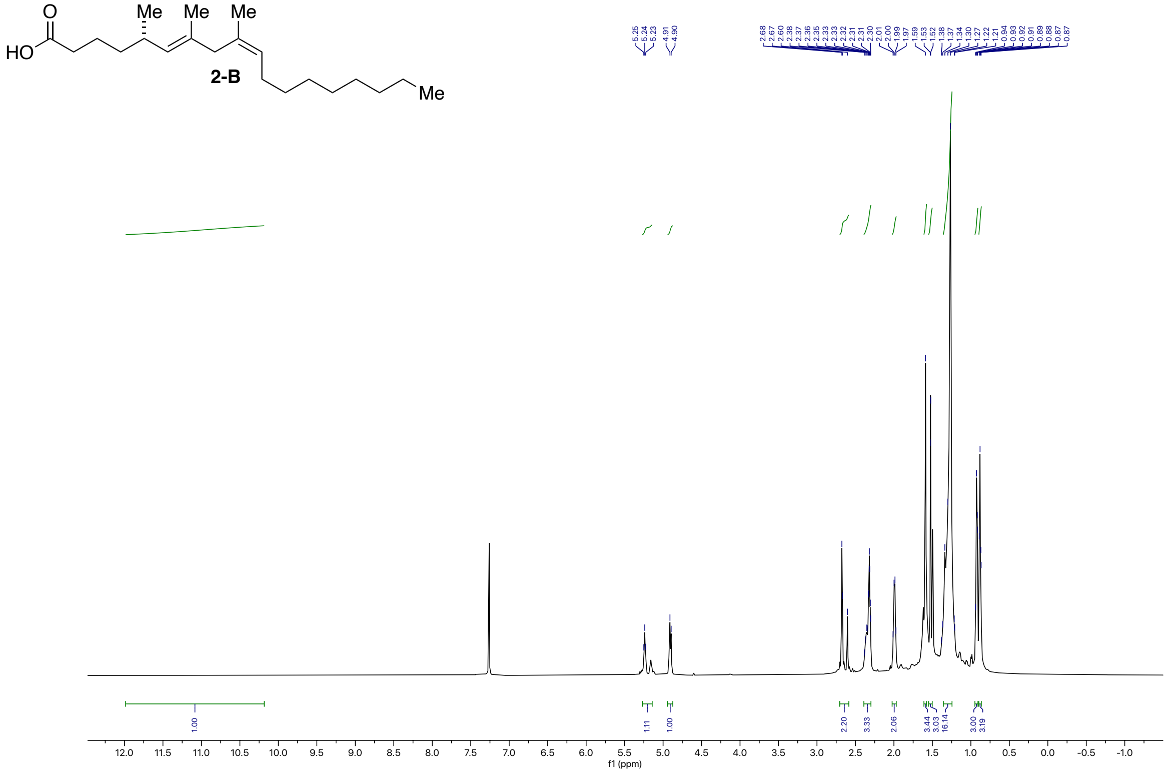

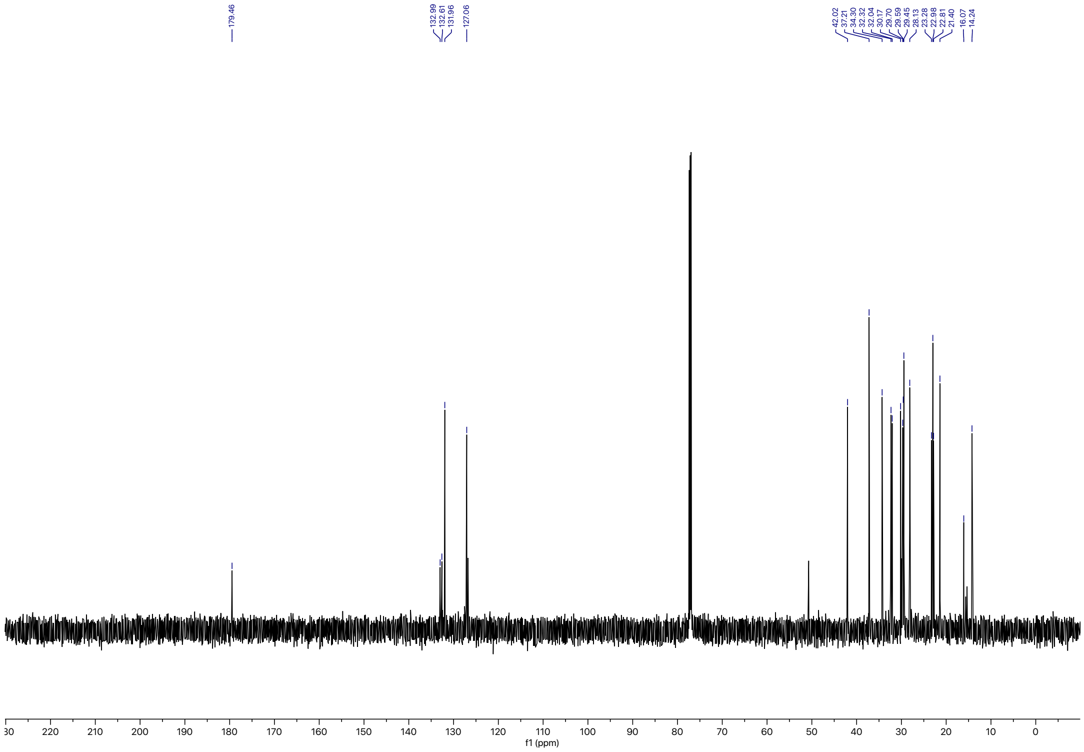

^{1}H NMR (600 MHz, $CDCl_3$) and ^{13}C NMR (150 MHz, $CDCl_3$) of **2-C** (^{13}C signal at 32.3 ppm not selectable via NMR software)

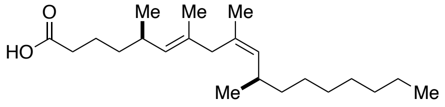

2-C

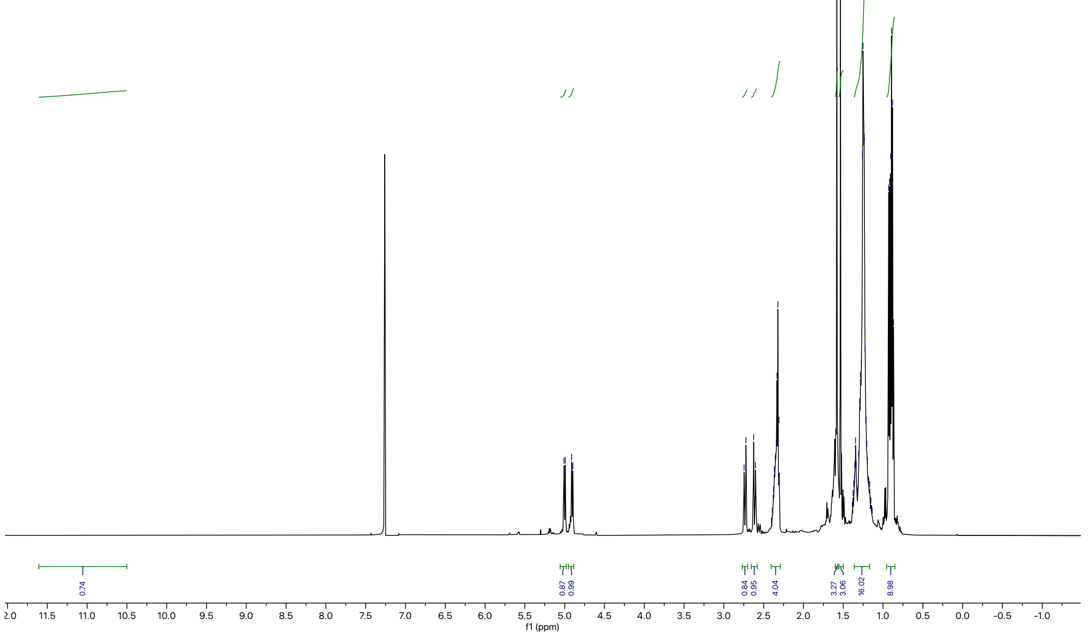

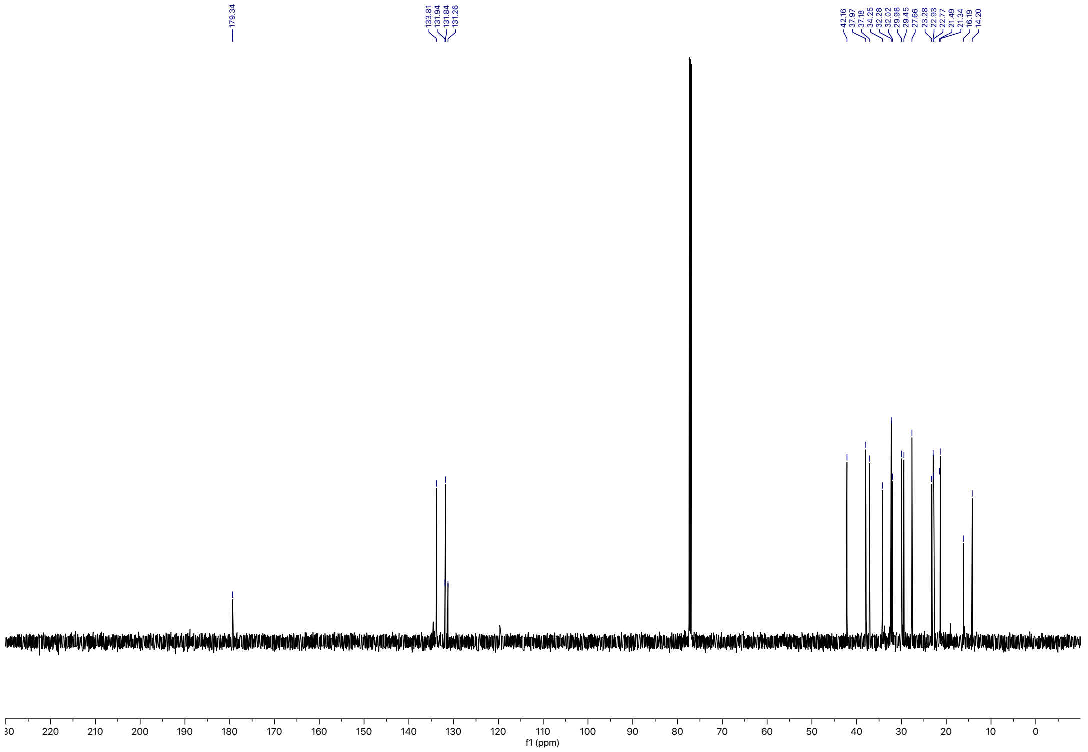

¹H NMR (600 MHz, $CDCl_3$) and ¹³C NMR (150 MHz, $CDCl_3$) of **2-D** (¹³C signal at 32.3 ppm not selectable via NMR software)

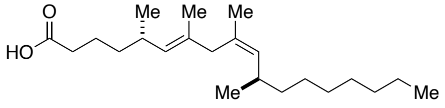

2-D

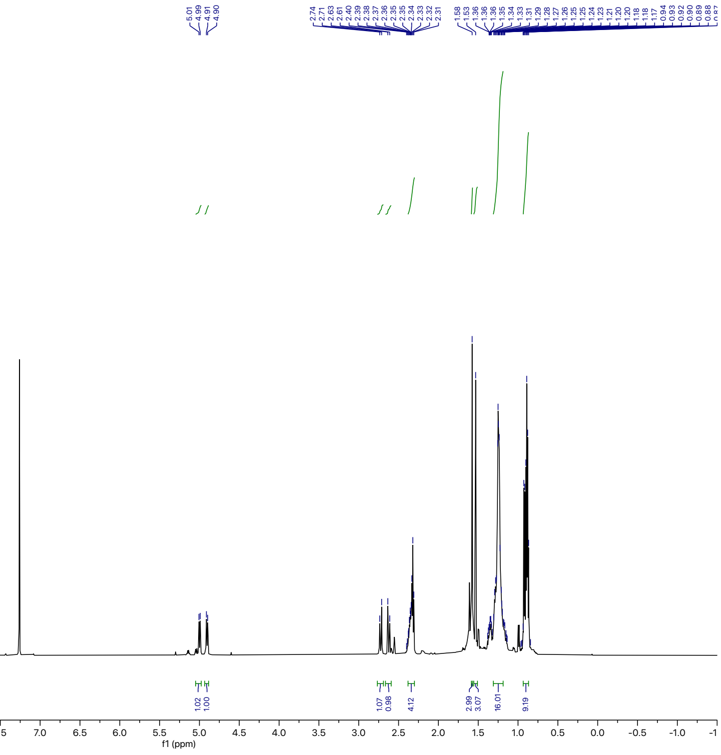

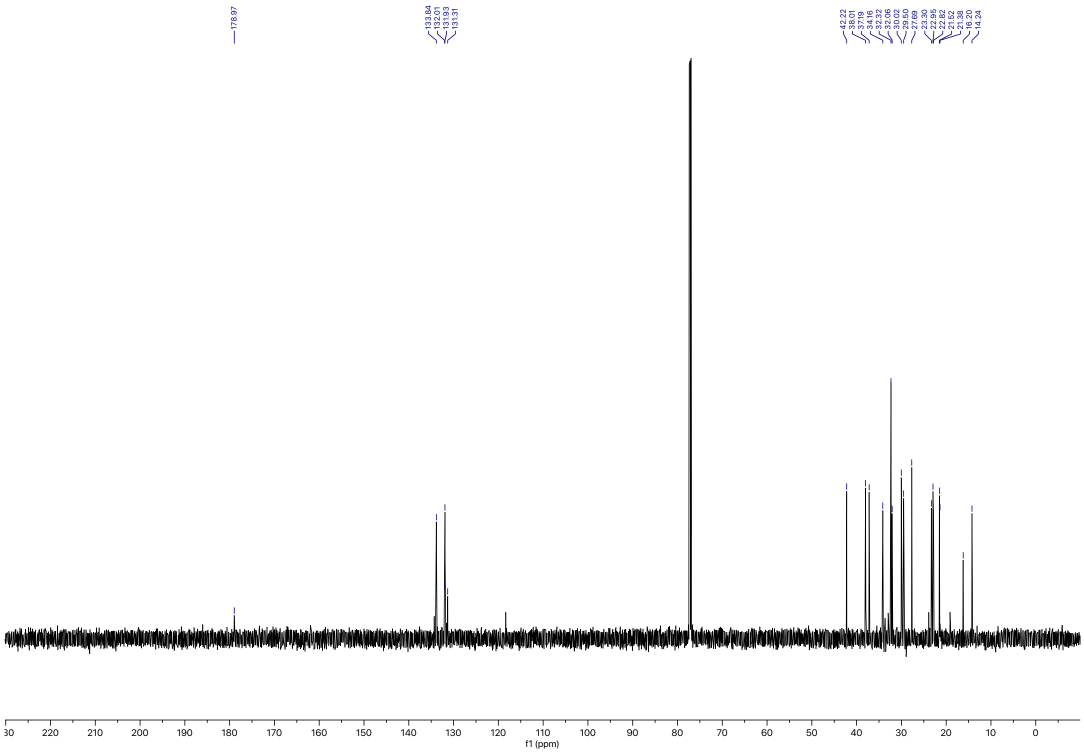

¹H NMR (600 MHz, $CDCl_3$) and ¹³C NMR (150 MHz, $CDCl_3$) of **2-E** (¹³C signal at 32.3 ppm not selectable via NMR software)

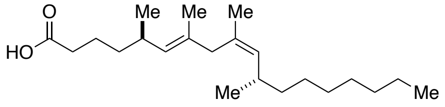

2-E

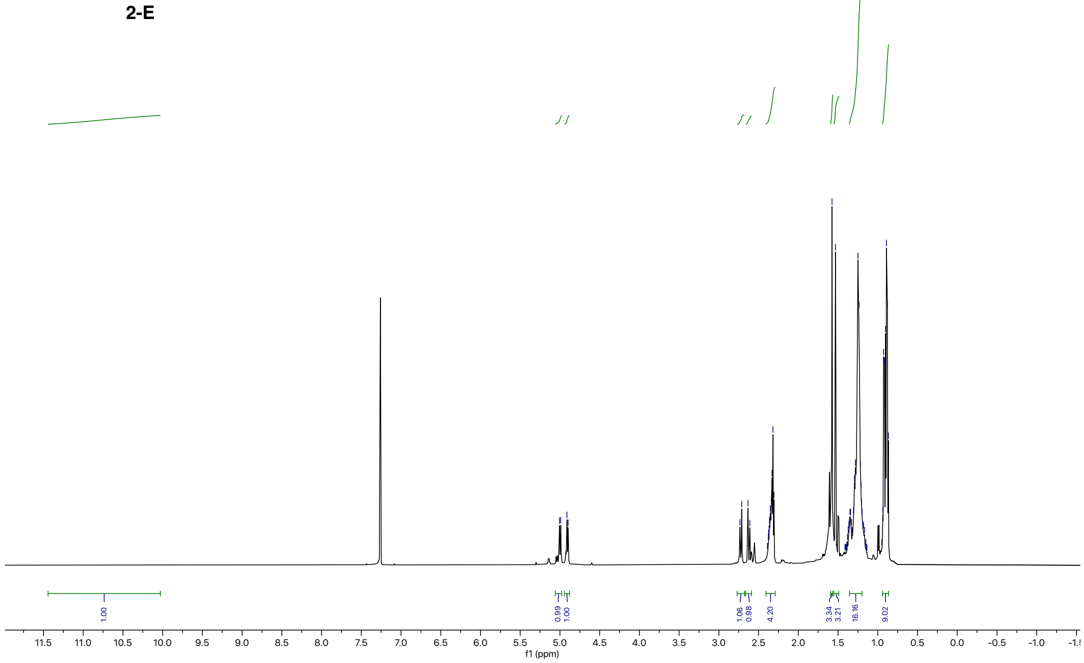

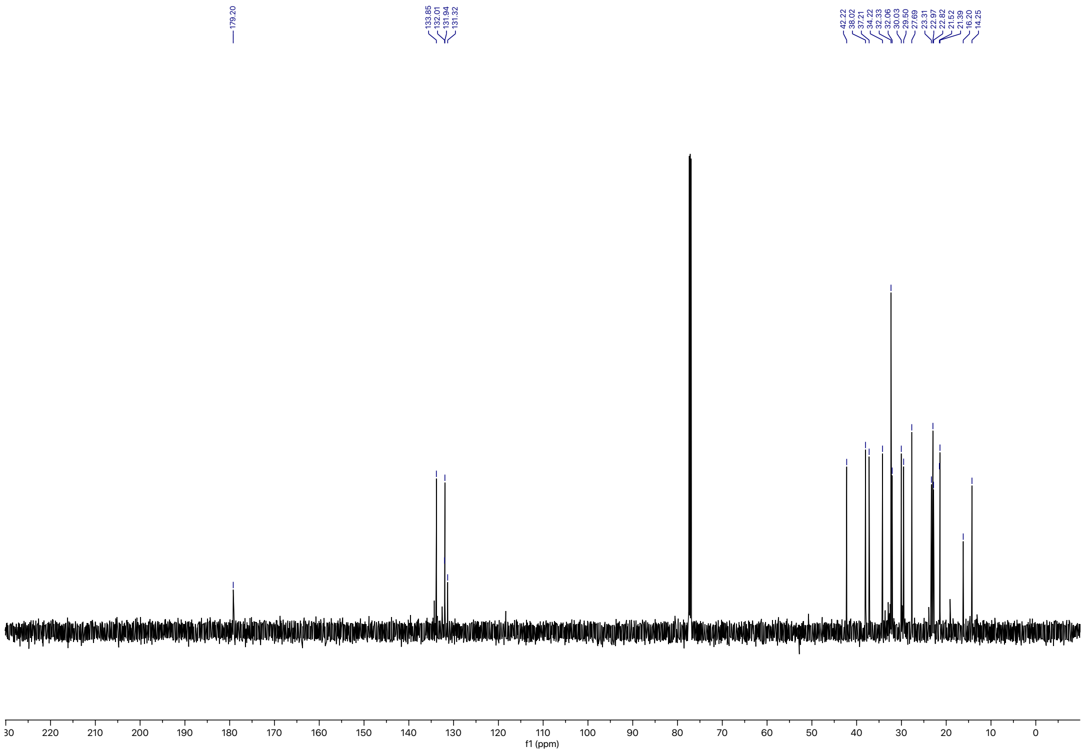

^{1}H NMR (600 MHz, $CDCl_3$) and ^{13}C NMR (150 MHz, $CDCl_3$) of **2-F**

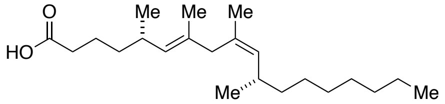

2-F

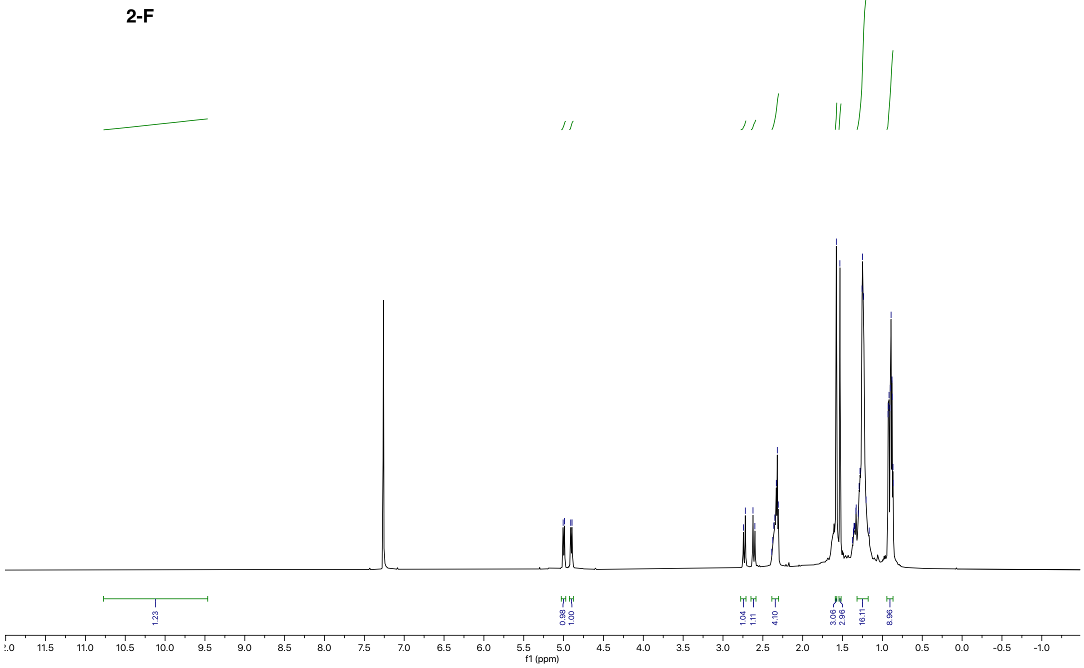

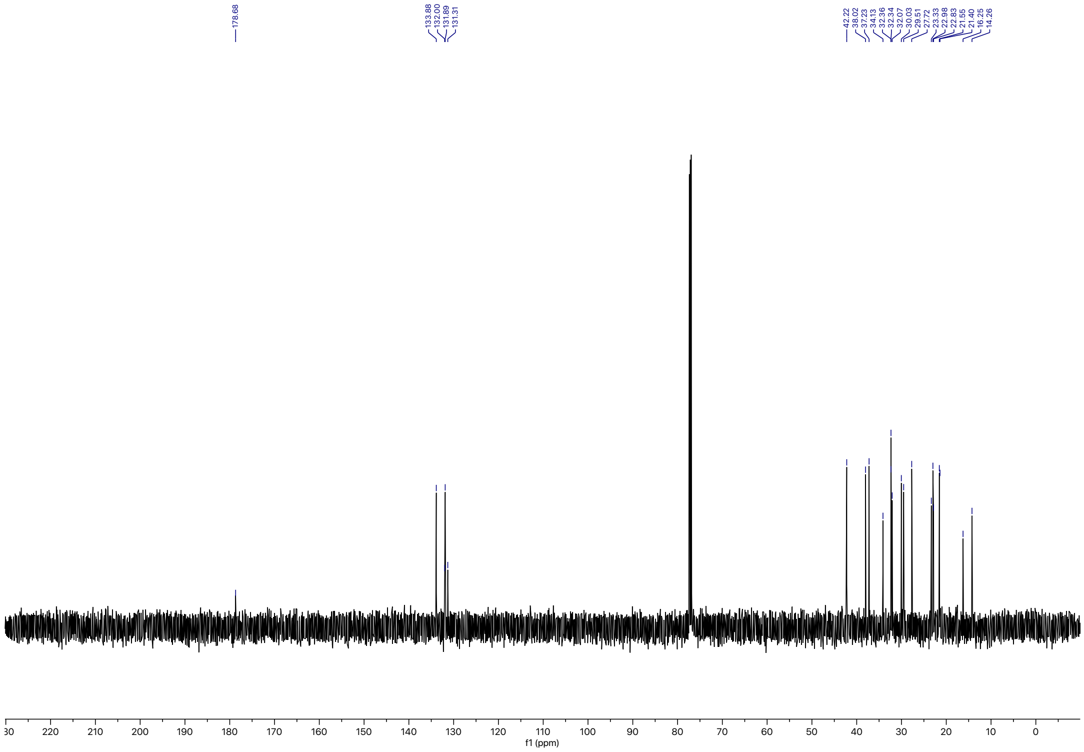

^{1}H NMR (600 MHz, $CDCl_3$) and ^{13}C NMR (150 MHz, $CDCl_3$) of **3-A**

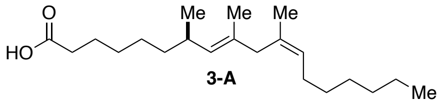

3-A

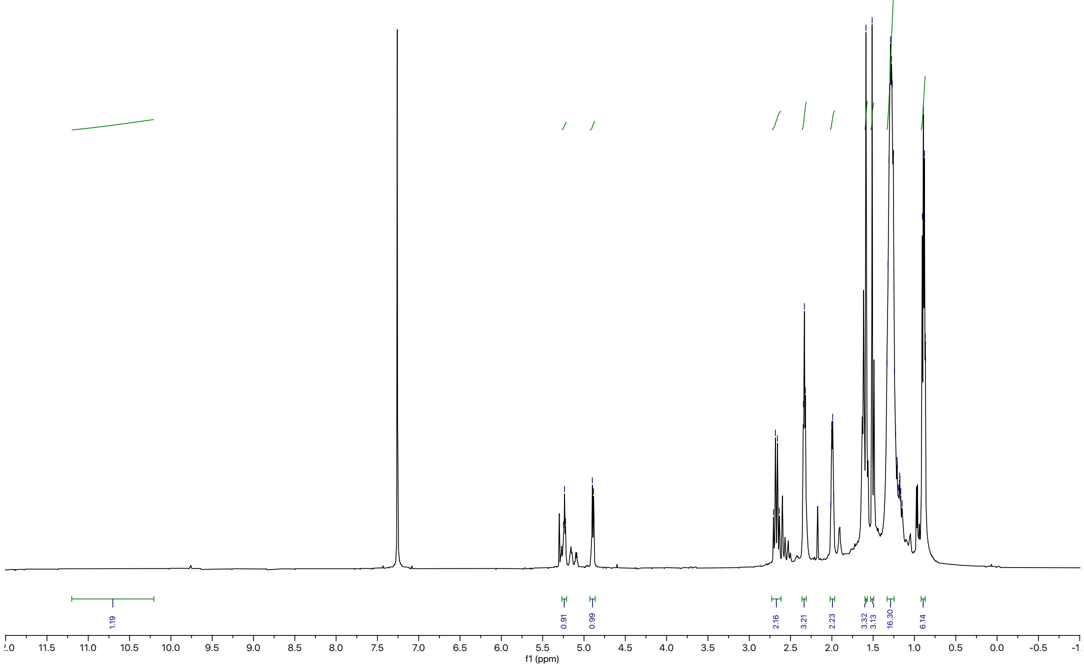

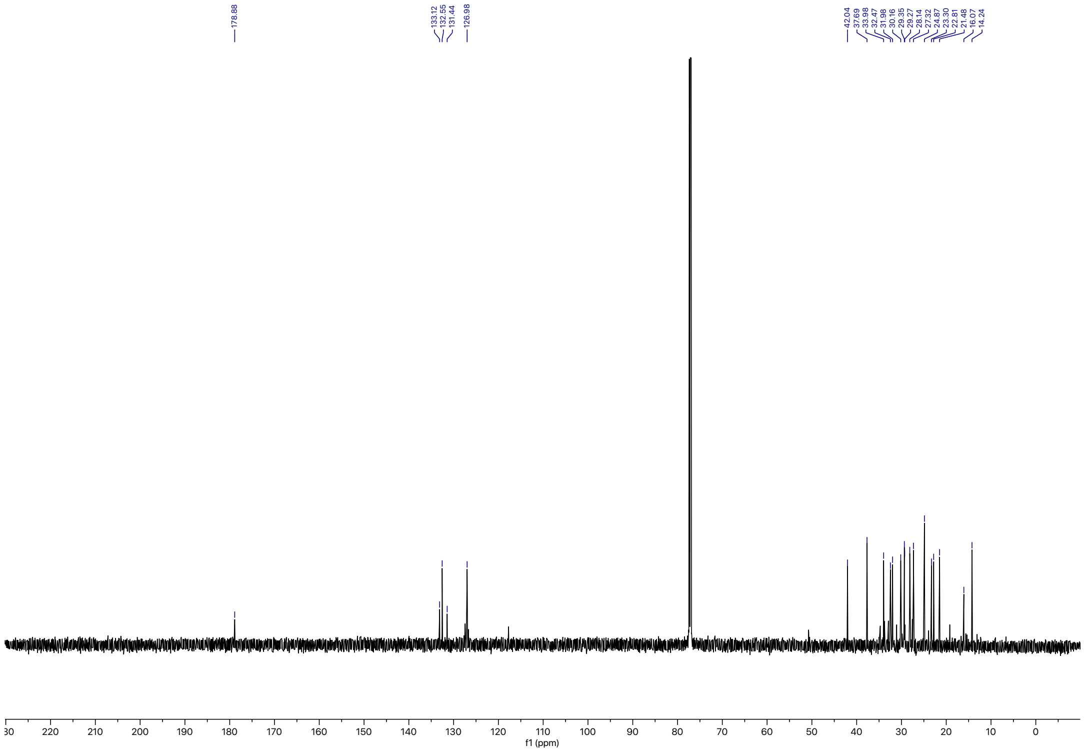

^{1}H NMR (600 MHz, $CDCl_3$) and ^{13}C NMR (150 MHz, $CDCl_3$) of **3-B**

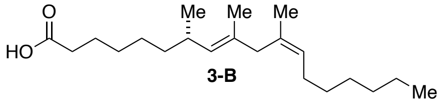

3-B

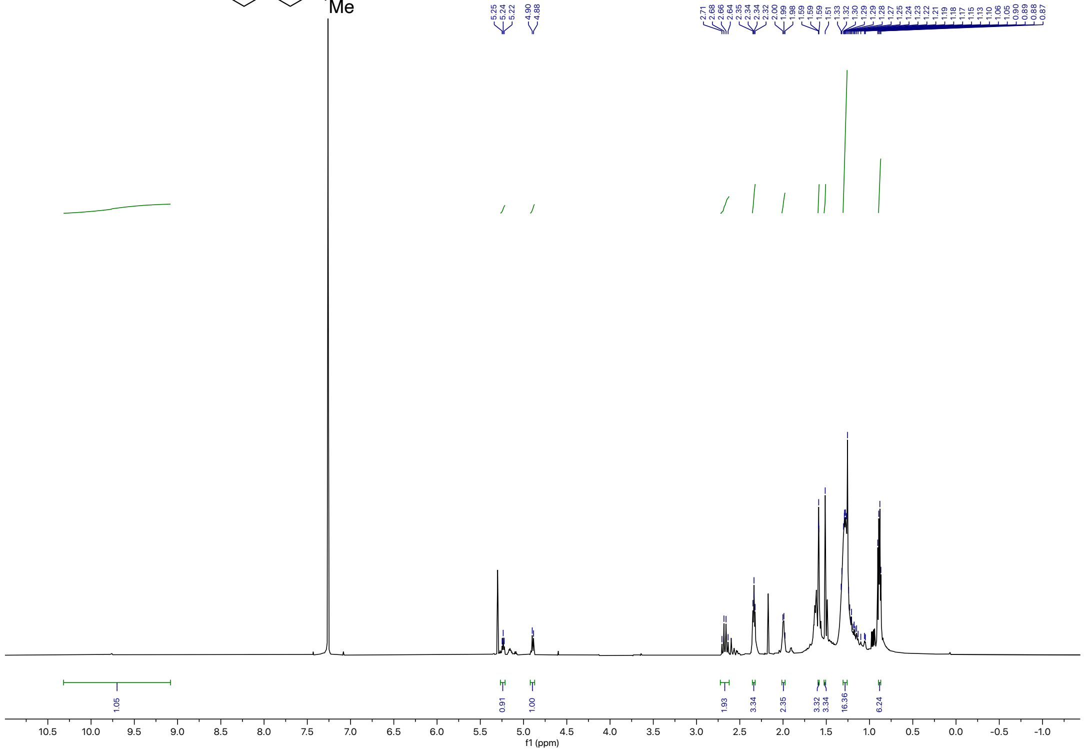

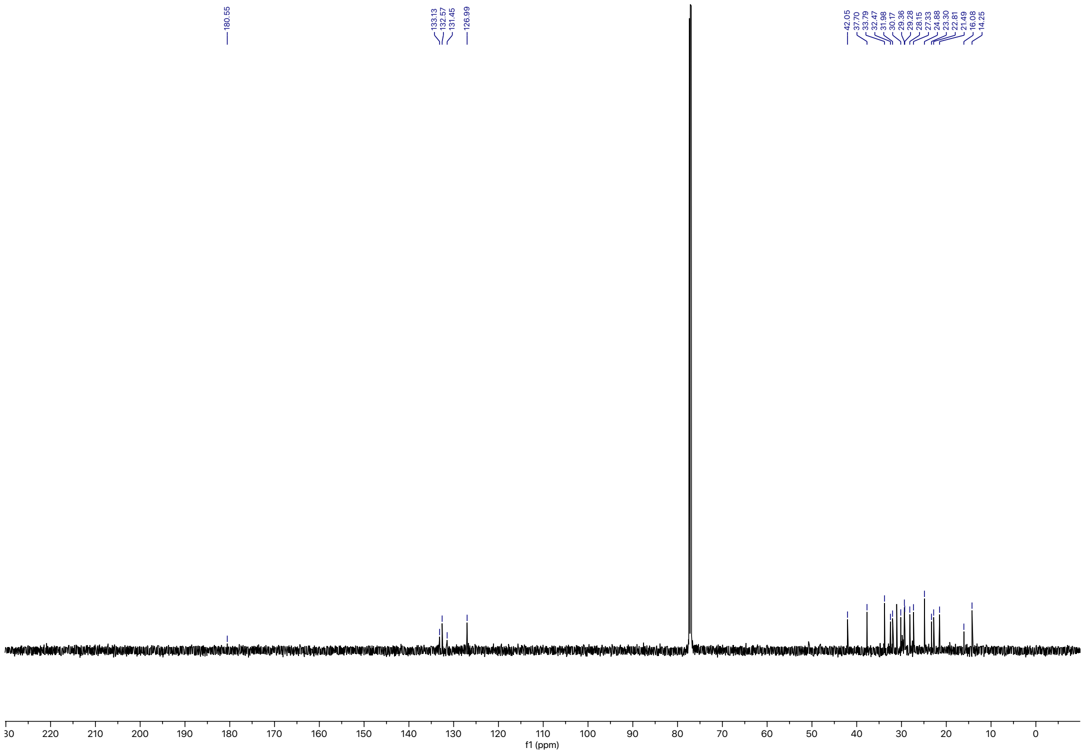

^{1}H NMR (600 MHz, $CDCl_3$) and ^{13}C NMR (150 MHz, $CDCl_3$) of **3-C**

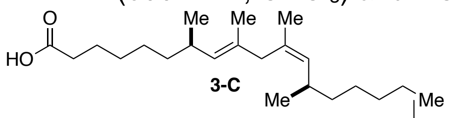

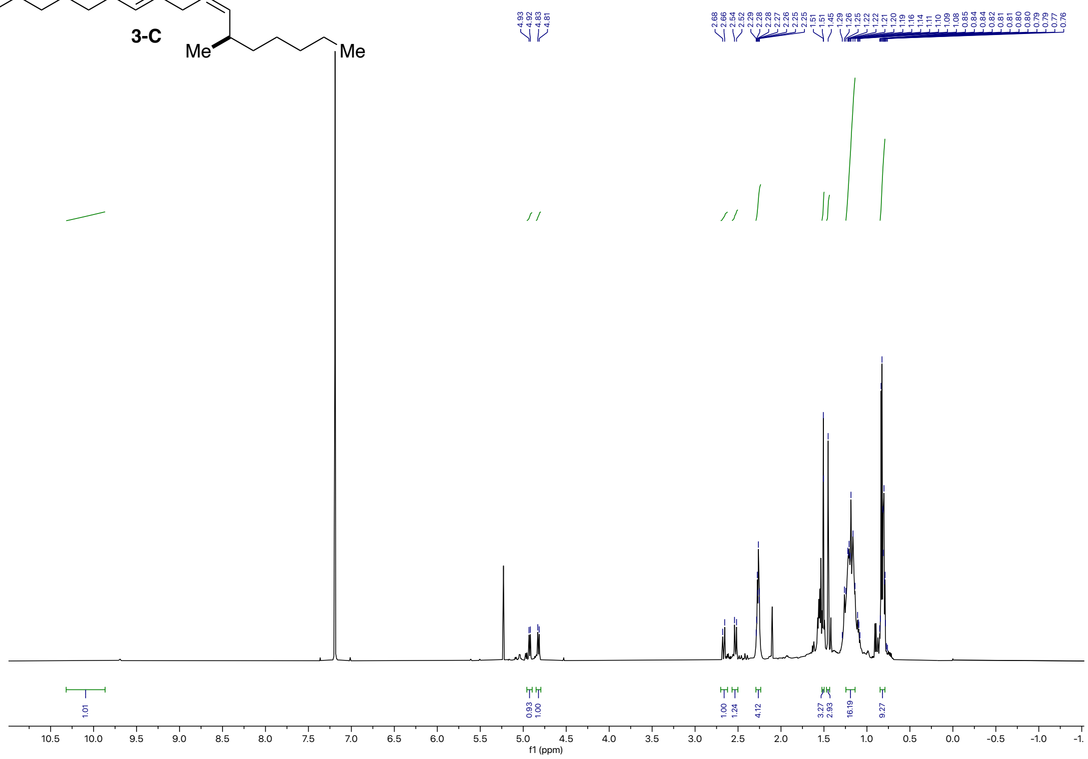

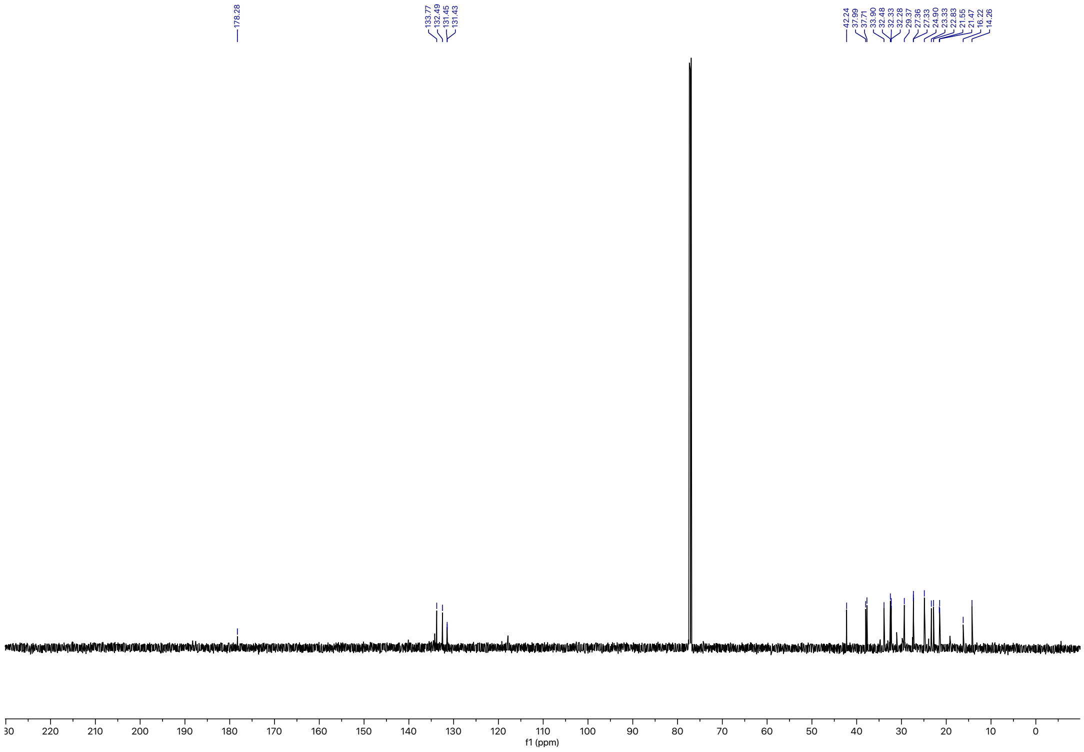

^{1}H NMR (600 MHz, $CDCl_3$) and ^{13}C NMR (150 MHz, $CDCl_3$) of **3-D** (^{13}C signal at 131.4 ppm not selectable via NMR software)

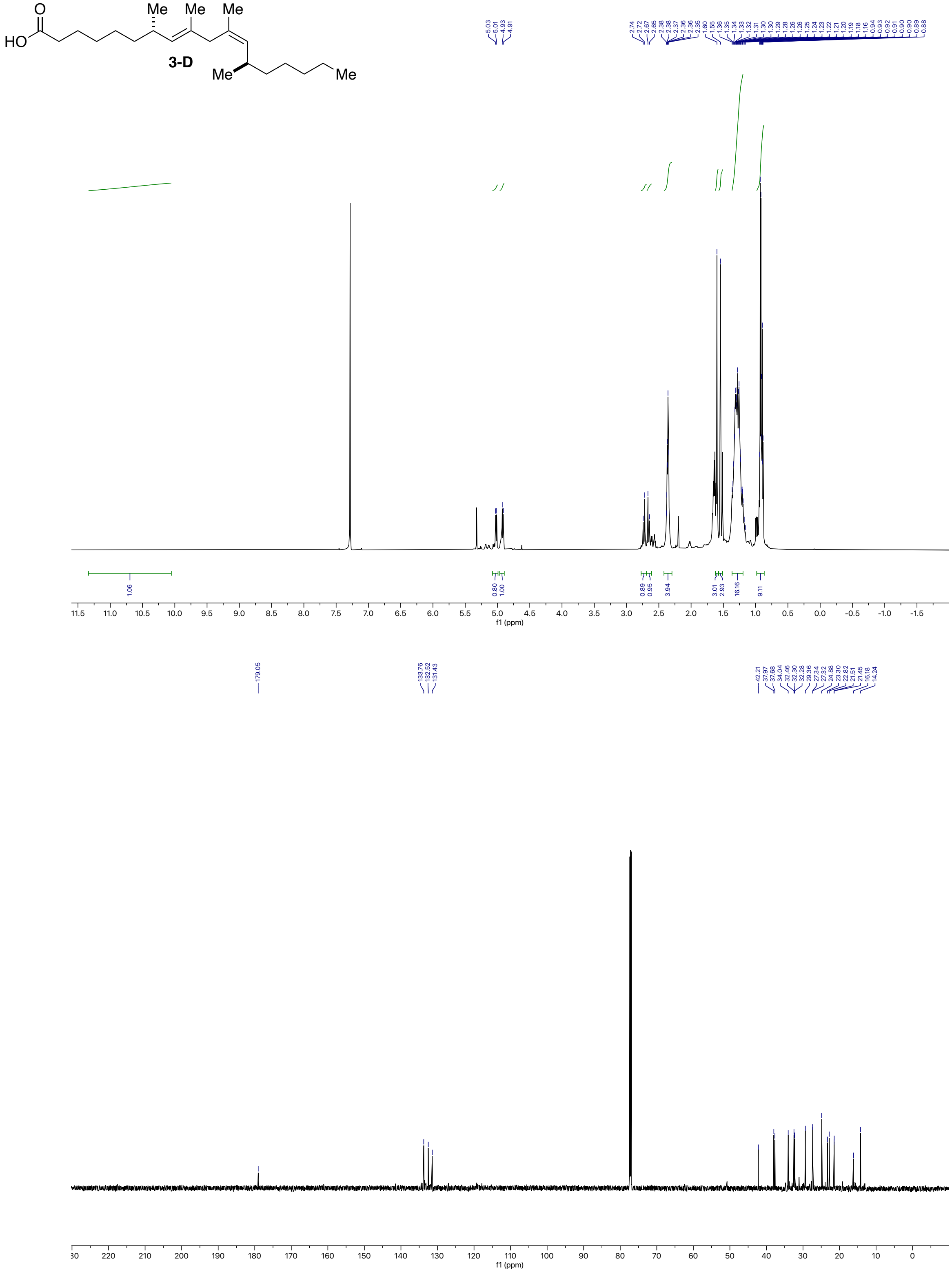

^1H NMR (600 MHz, $CDCl_3$) and ^{13}C NMR (150 MHz, $CDCl_3$) of **3-E** (^{13}C signal at 131.5 ppm not selectable via NMR software)

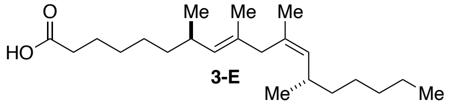

3-E

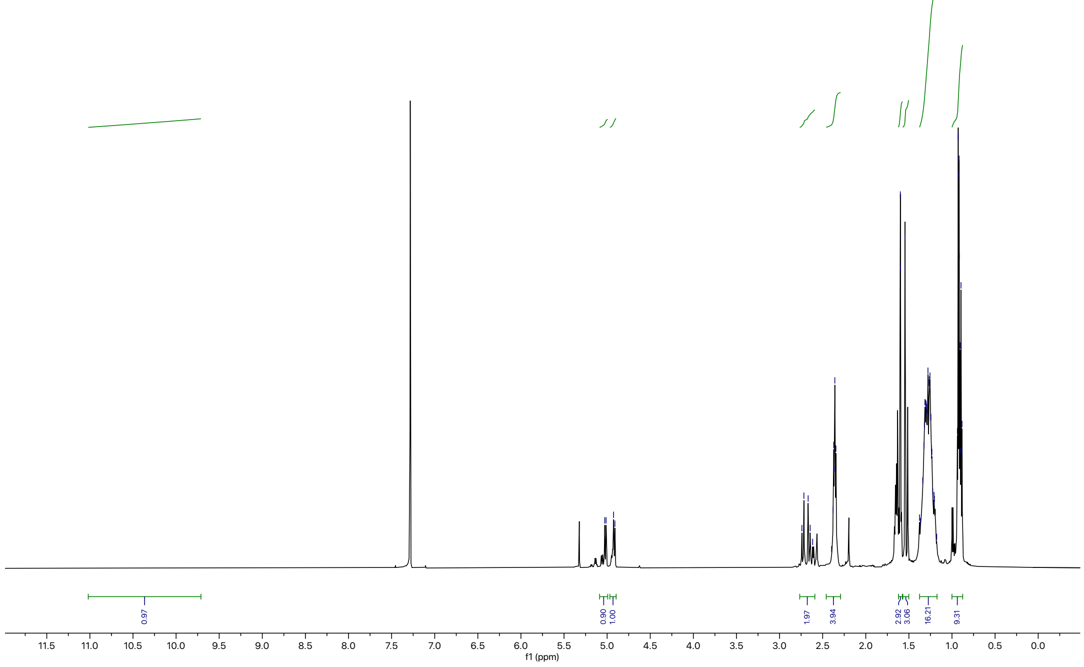

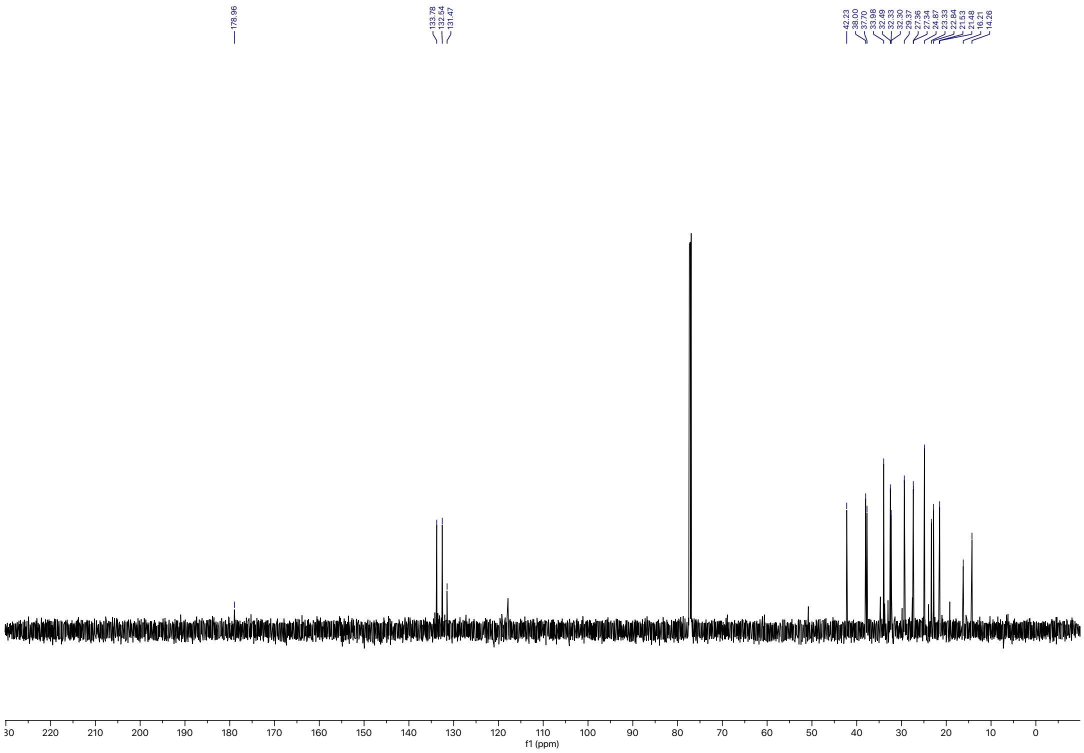

^{1}H NMR (600 MHz, $CDCl_3$) and ^{13}C NMR (150 MHz, $CDCl_3$) of **3-F**

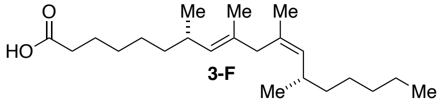

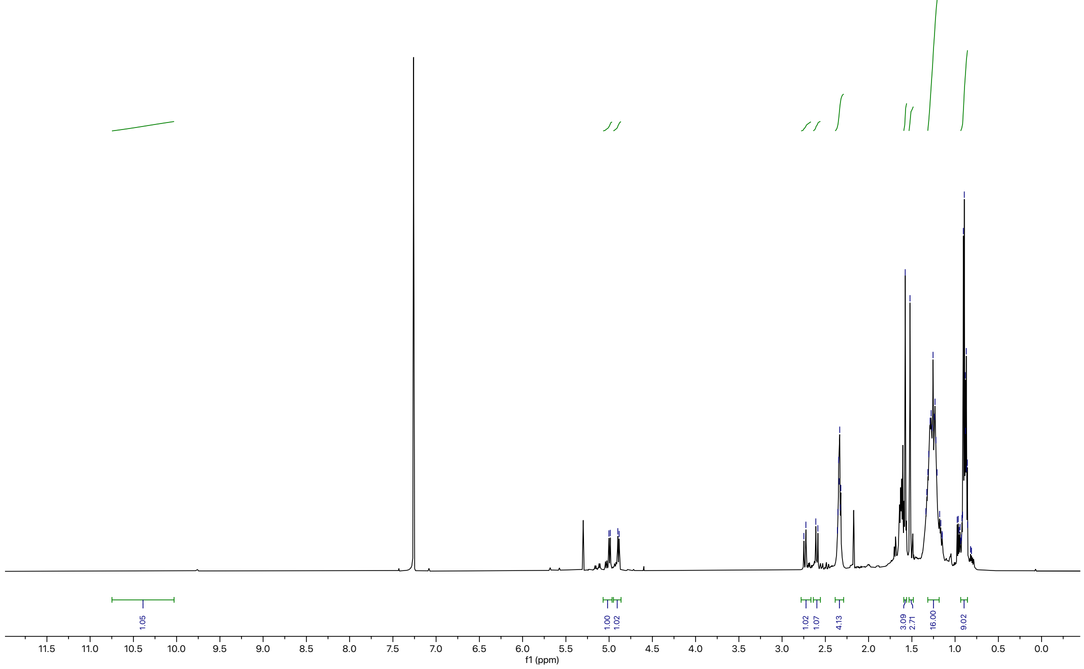

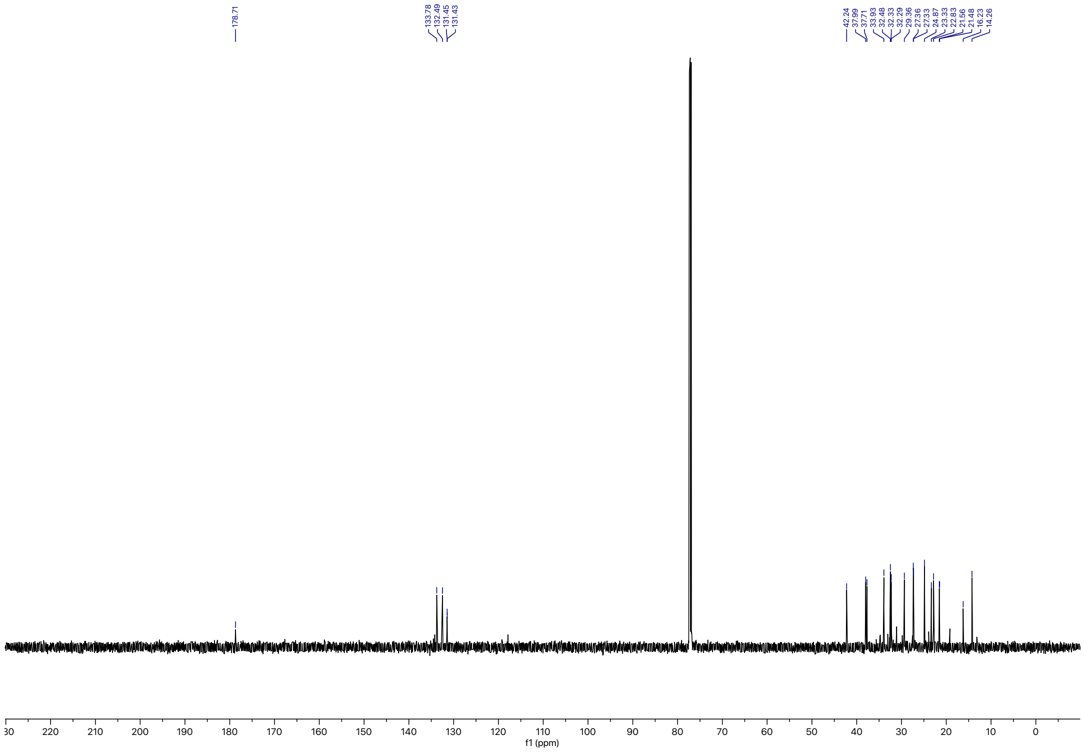

^{1}H NMR (600 MHz, $CDCl_3$) and ^{13}C NMR (150 MHz, $CDCl_3$) of **4-A**

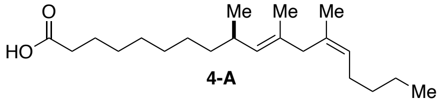

4-A

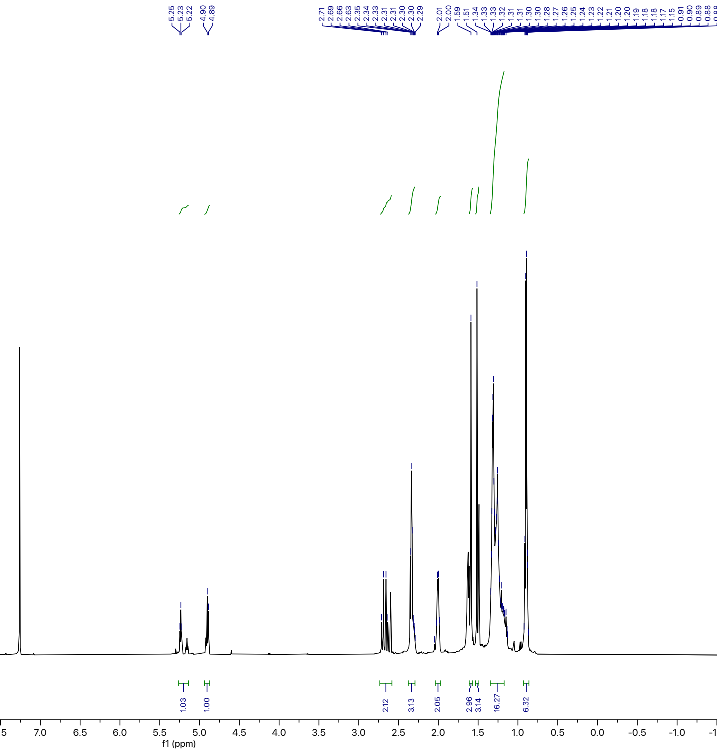

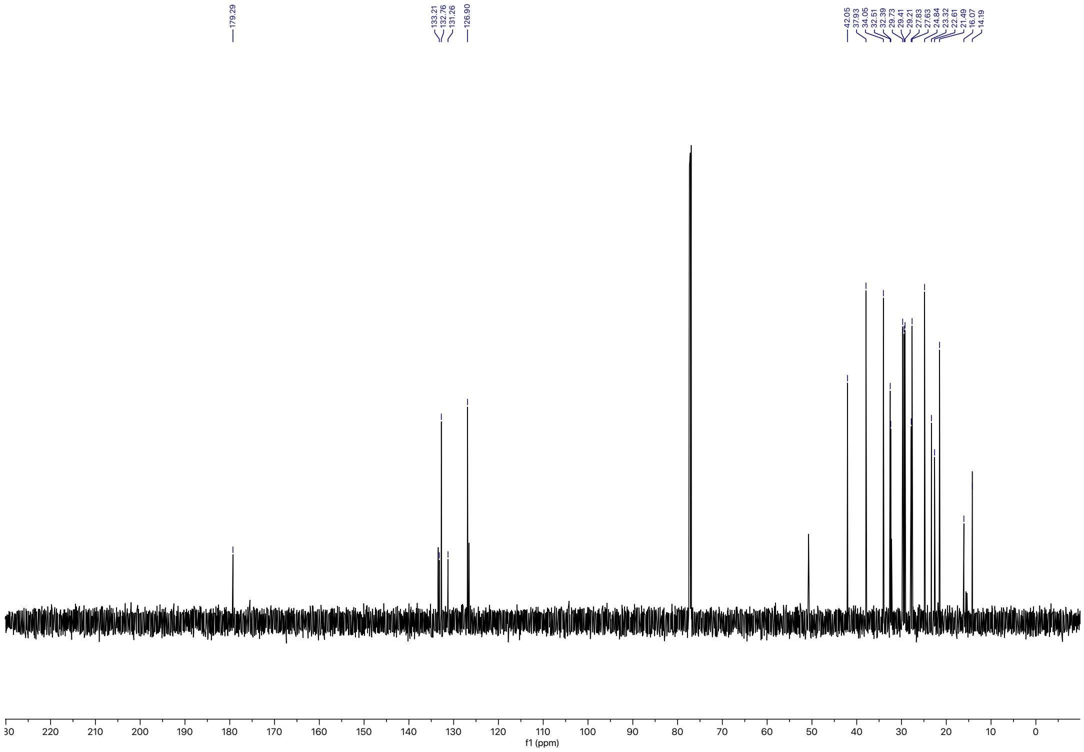

^{1}H NMR (600 MHz, $CDCl_3$) and ^{13}C NMR (150 MHz, $CDCl_3$) of **4-B**

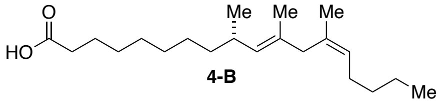

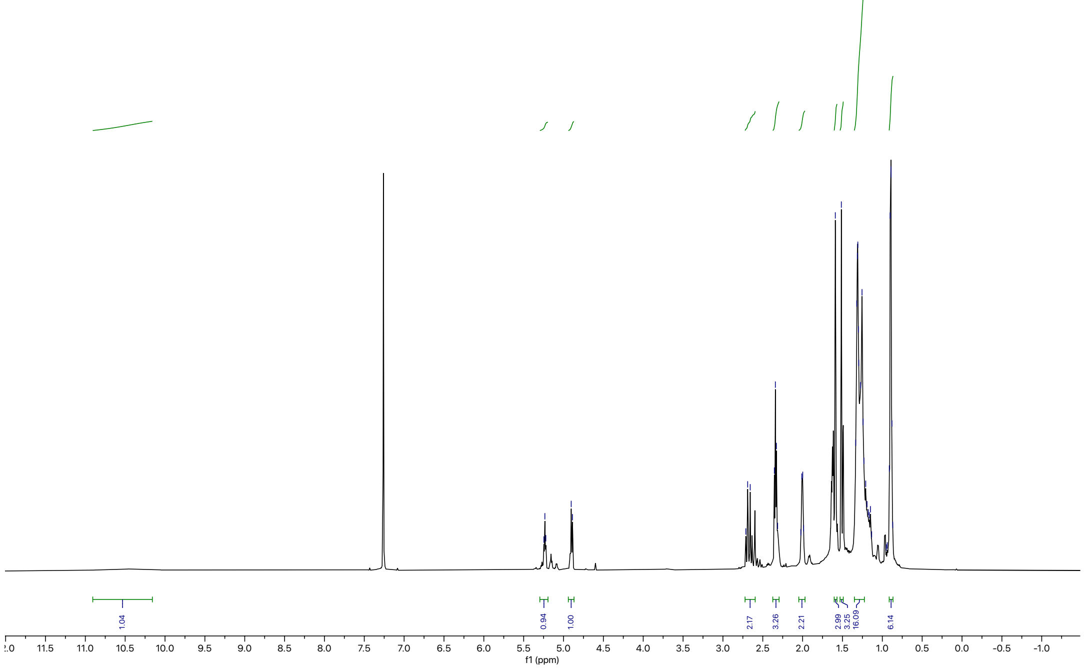

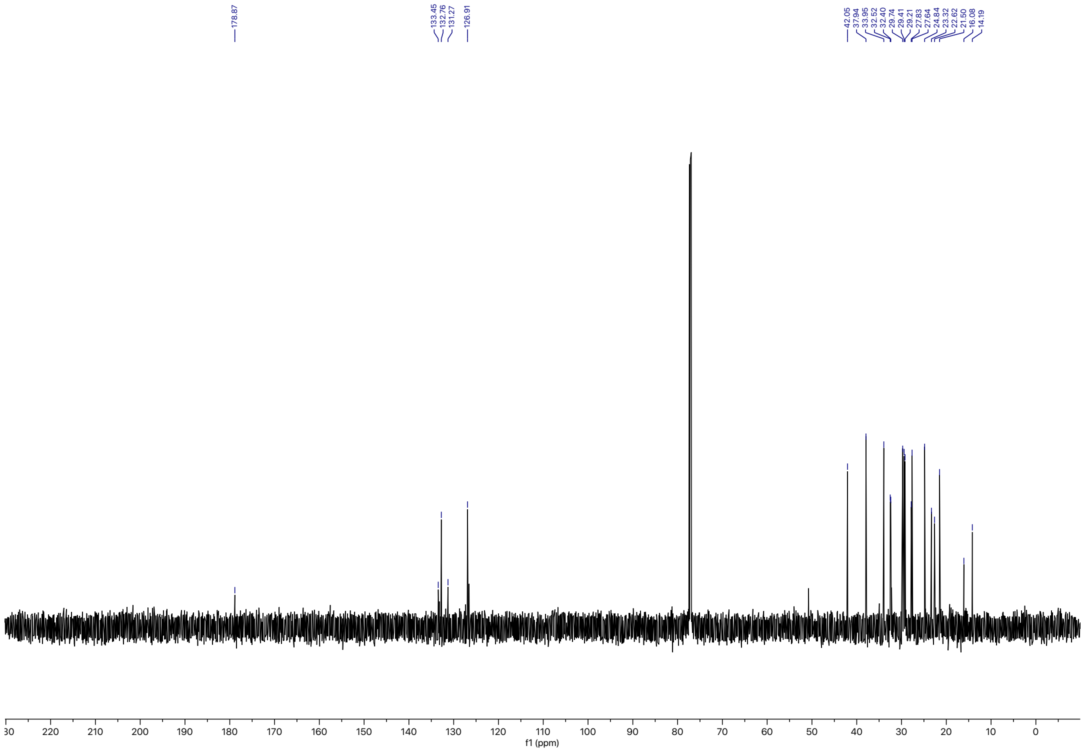

^{1}H NMR (600 MHz, $CDCl_3$) and ^{13}C NMR (150 MHz, $CDCl_3$) of **4-C**

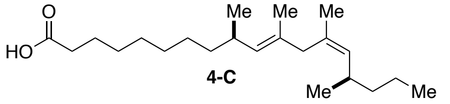

4-C

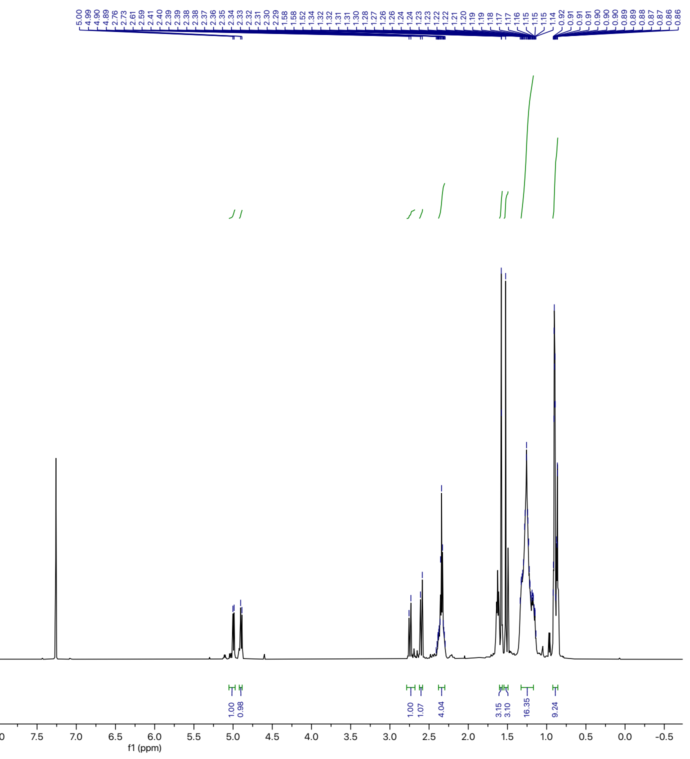

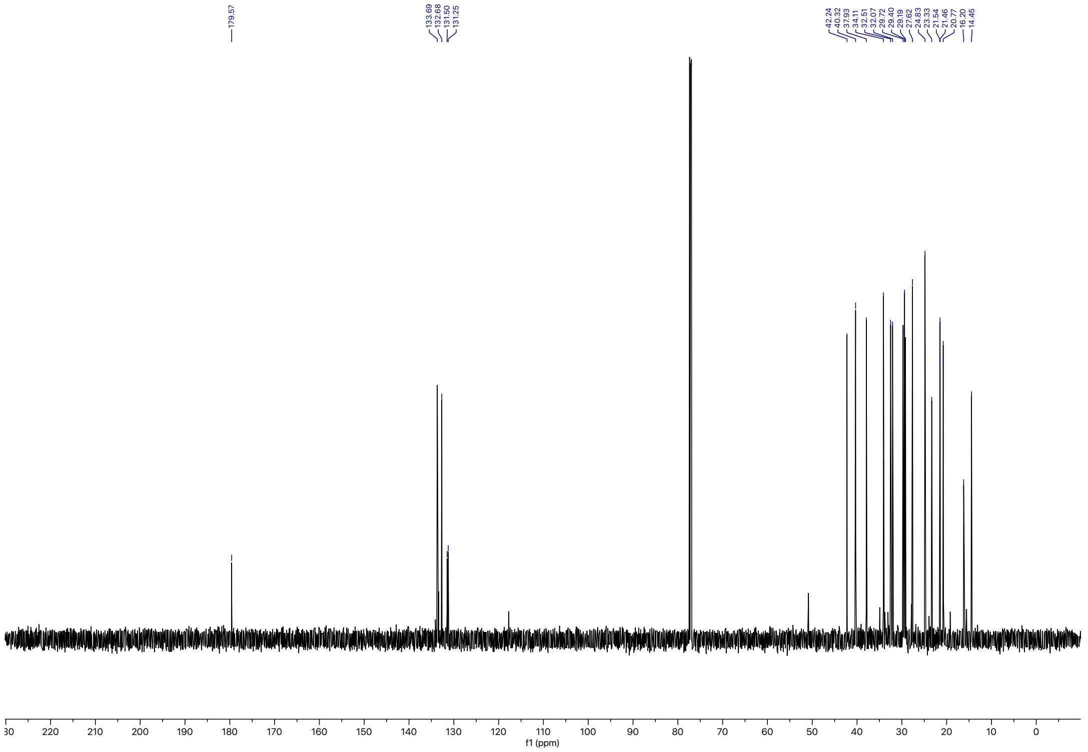

^{1}H NMR (600 MHz, $CDCl_3$) and ^{13}C NMR (150 MHz, $CDCl_3$) of **4-D**

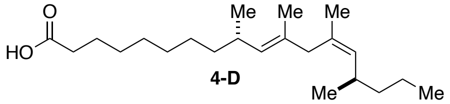

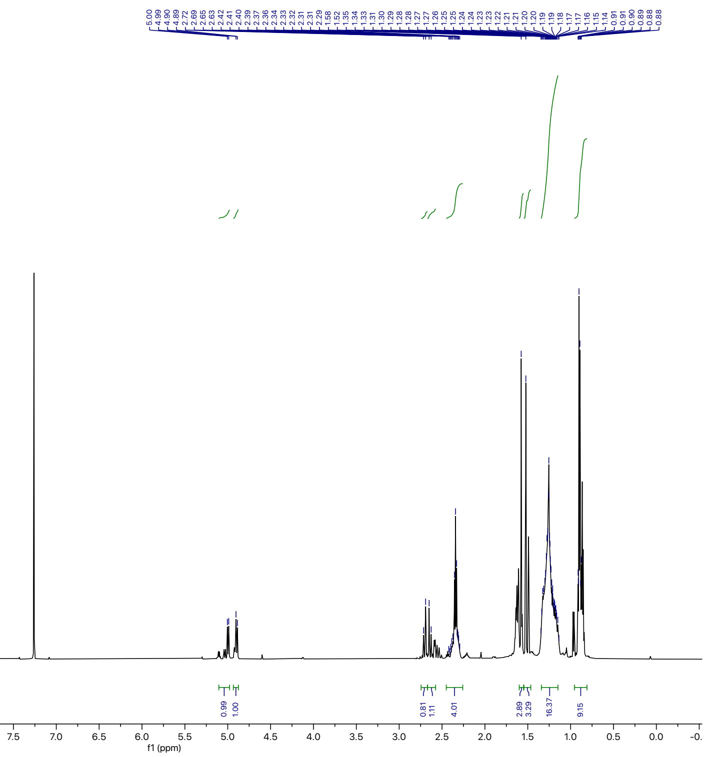

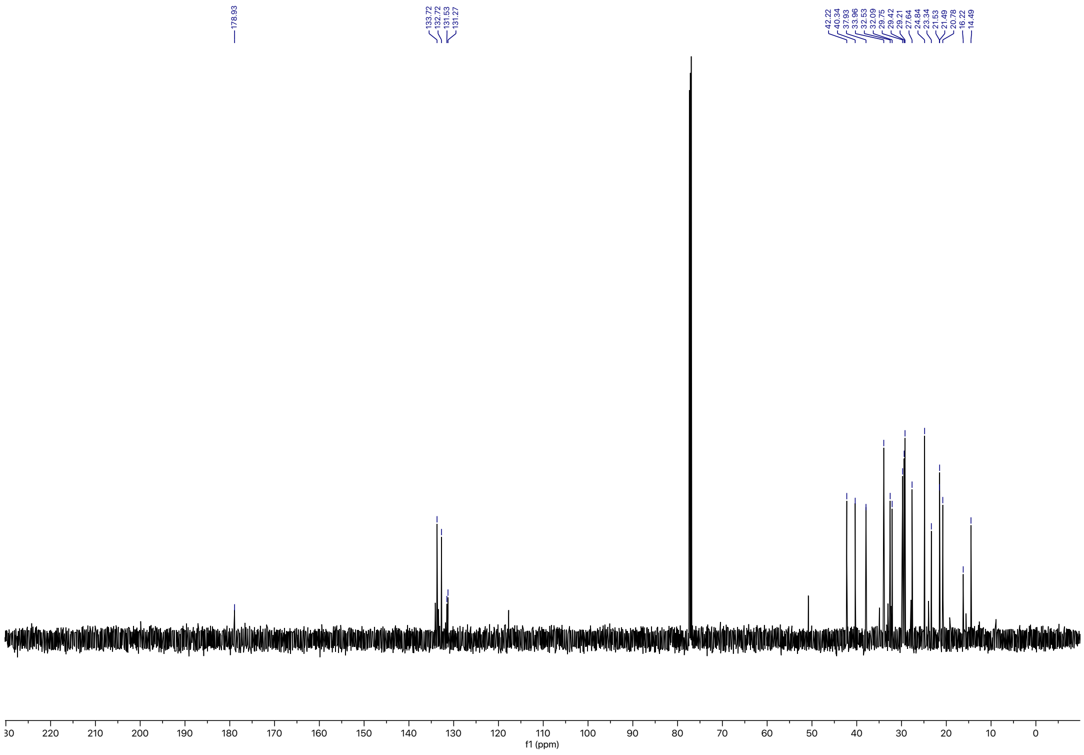

^{1}H NMR (600 MHz, $CDCl_3$) and ^{13}C NMR (150 MHz, $CDCl_3$) of **4-E**

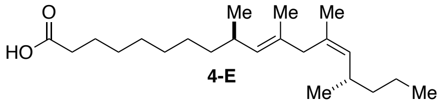

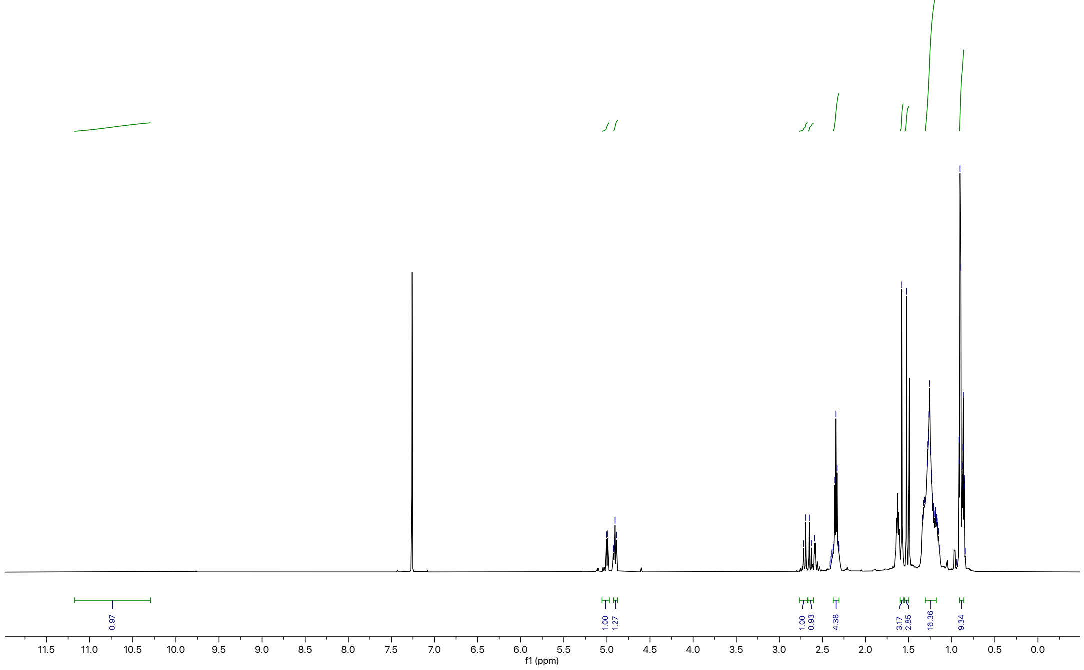

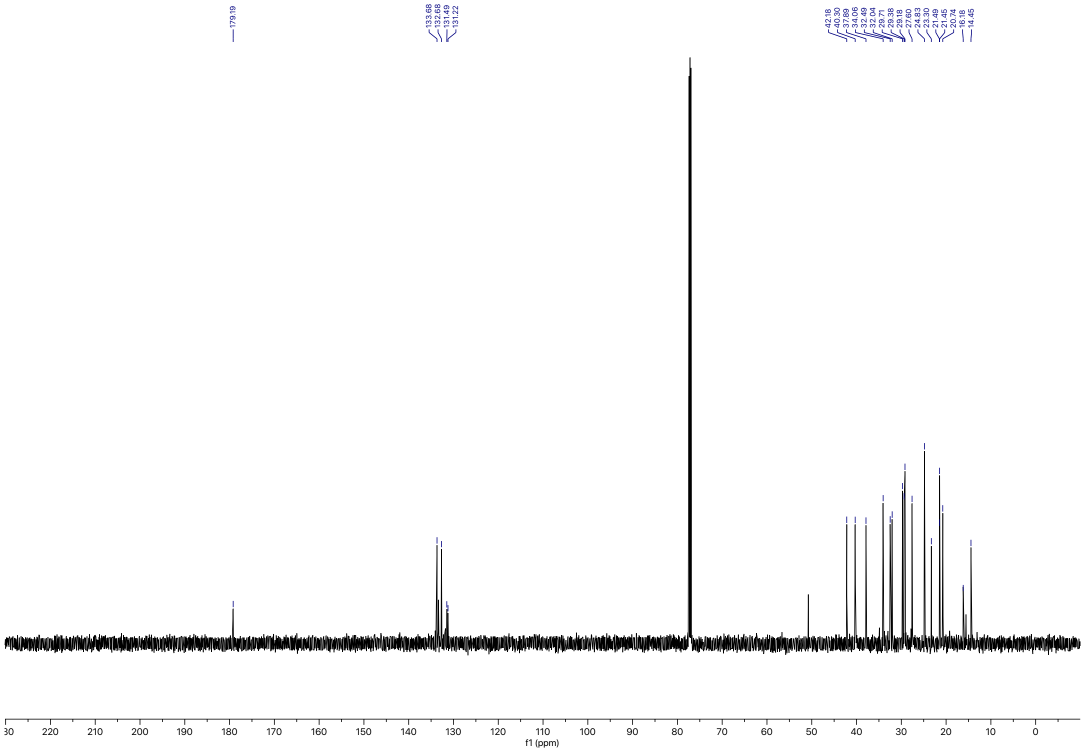

^{1}H NMR (600 MHz, $CDCl_3$) and ^{13}C NMR (150 MHz, $CDCl_3$) of **4-F**

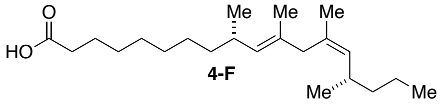

4-F

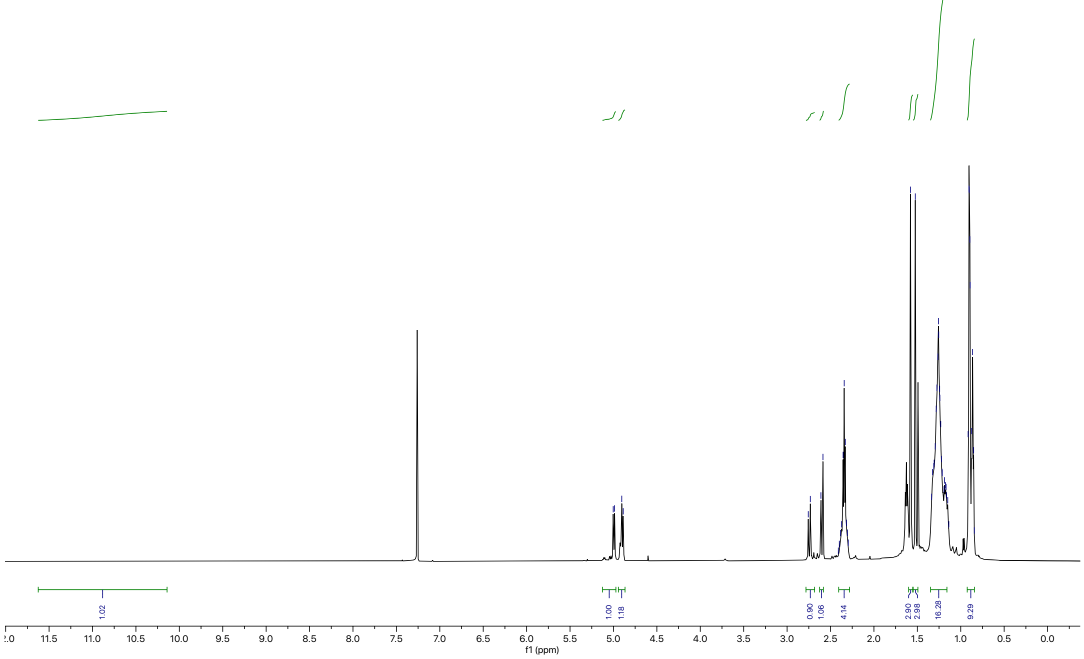

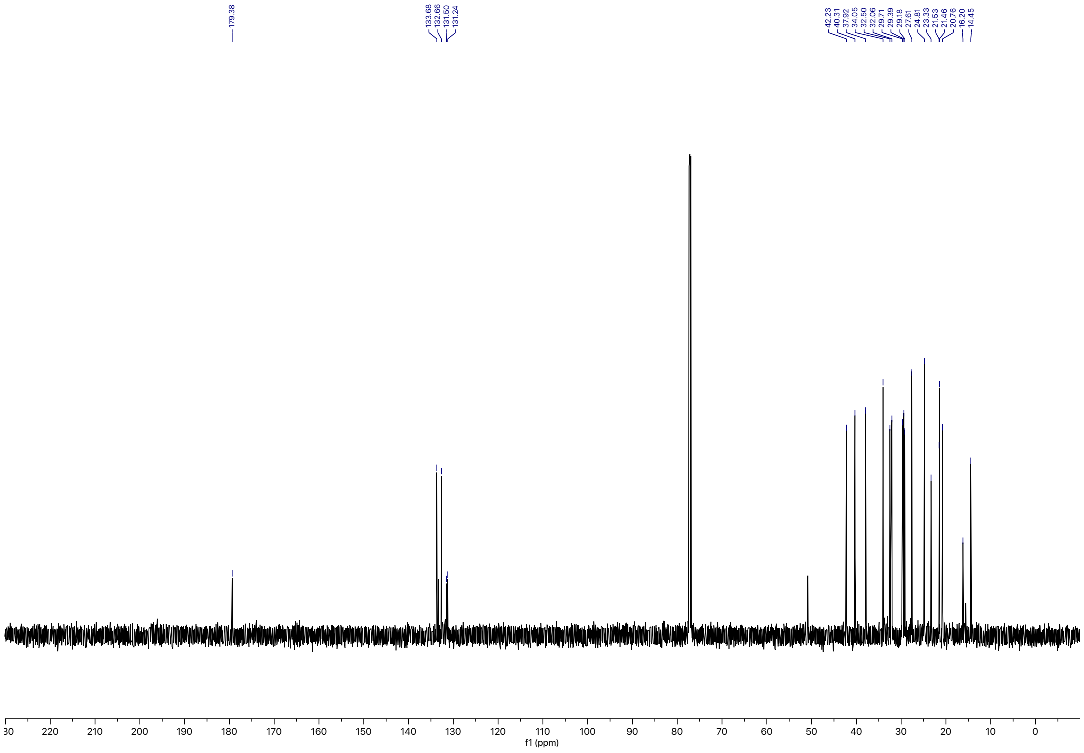

Supplement: Supplementary file 1 — oc3c01155_si_002.pdf [file oc3c01155_si_002.pdf]
